# Supplementary material for: Microevolutionary dynamics show tropical valleys are deeper for montane birds of the Atlantic Forest
Source: Nat Commun. 2021 Nov 1;12:6269. doi: 10.1038/s41467-021-26537-9 (PMC8560783; doi:10.1038/s41467-021-26537-9)
Supplement: Supplementary file 1 — Supplementary Information [file 41467_2021_26537_MOESM1_ESM.pdf]

Supplementary Information

Methods full description

Genomic Sampling

To obtain genetic samples, we conducted multiple field expeditions, sampling the main mountain regions of the Southern Atlantic Forest. A total of 145 specimens were collected and deposited at the Laboratorio de Genetica e Evolucao Molecular de Aves (LGEMA) and Museu de Zoologia da Universidade de São Paulo (MZUSP). Additional samples were requested from tissue collections and natural history museums (Supplementary Table 12). A total of 227 tissue samples were obtained for 21 species with an average of 11.2 samples per species (Supplementary Table 12). To obtain population genetic summary statistics and estimate demographic parameters, we used a double digest restriction site associated DNA technique (ddRAD; citation). Genomic DNA was extracted from pectoral muscle using the QIAGEN DNeasy tissue and Blood kit (Valencia, CA) and quantified in the Qubit 2.0 fluorometer. Library preparation and Illumina sequencing were outsourced to the University of Wisconsin Biotechnology Center (UWBC, Madison, WI) using *Pst*I and *Msp*I restriction enzymes. The sequence barcode was applied to a single cut site (TGCA). Library sequencing was conducted using three lanes of Illumina HiSeq2500 on a single-end 150bp mode. Sample demultiplexing, de novo assembly, and SNP calling for each species was performed in ipyrad v0.9.18 [1]. After demultiplexing all samples tagged with uniquely designed barcodes, each species was analyzed separately. The maximum number of bases per read with Phred quality score < 20 was set to 3. The minimum read depth for base calling was set to 6. We used a cluster threshold of 90% of similarity for the *de novo* assembly and dropped all fragments < 50 bp. Our filtering scheme allowed only bi-allelic sites and removed loci with excessive (> 5) heterozygous sites. Finally, only loci with no more than 25% missing data, and with sites with a proportion of heterozygous individuals < 0.75 were retained. To reduce the possibility of linked SNPs only one variant per locus was retained for downstream analyses.

Species distribution models and occupied environmental space

To obtain species occurrences, we data mined and filtered records from the Global Biodiversity Information Facility, GBIF.org (04/28/2020) GBIF Occurrence Download (DOI 10.15468/dl.8383sp). The initial search yielded a total of 53,853 records and a mean of 2,564 records per species. Points outside the species known geographic and elevational distribution were filtered out. We reduced sampling biases and homogenized the density of occurrences across space, by applying a spatial thinning using a 10 km distance in spThin v0.1.0.1 [2]. After filtering our final data set had 13,986 occurrence records (mean = 608.1 and SD= 371.3 per species; Supplementary Data 3). To test for the effect of elevational migration we conducted nonparametric Wilcoxon tests between occurrence records obtained in warm (Spring/Summer) versus cold (Fall/Winter) months. Given the lack of significant differences between records obtained in different periods of the year for most of the species (Supplementary Fig. 5) we used all occurrences in our species distribution models.

The extent of current and past climatic suitable areas across the Southern Atlantic Forest for our focal 21 species was accessed through species distribution models (SDMs). We used the 19 bioclimatic variables from the CHELSA database [3] with a spatial resolution of 30s. Current and past habitat suitability was estimated in Maxent v3.4.1 [4]. Given that the regularization method implemented in Maxent produces stable results with correlated variables, the 19 bioclimatic variables were used together [5]. Models were constrained to areas that are known to be occupied by setting a minimum convex polygon around the occurrence localities buffered by 0.5 degrees (~55 km; [6]. From this background area, 10,000 random points were sampled and used in the training process of the models. Occurrence data per species was partitioned into five equal portions using the random k-fold cross-validations for model training and testing [7]. To avoid model overfitting, we evaluated distinct feature classes and regularization multipliers using the ENMeval v0.3.0 [8] in R. We explored regularization multipliers between 1 to 4 with 0.5 increments and the following feature classes: 'Linear', 'Linear + Quadratic', 'Linear + Quadratic + Hinge'. A hierarchical combination of methods was implemented to select the best combination of regularization multipliers and feature classes, which allowed to reduce model overfitting while maximizing model accuracy [9]. First, models were classified by the value of the omission rate at the 10% omission threshold [7,10,11]. Second, we filtered models by the "maximum test AUC" (area under the receiver-operating characteristic – ROC – curve; Peterson et al., 2011). This approach allowed us to maximize the model's discriminatory ability between true

presence and the background. Lastly, we classified models by the corrected Akaike Information Criterion (AICc). Final models were projected to a wider geographic distribution than the initial background, between the latitude degrees -15 and -40 and longitude degrees -39 and -65. To estimate historical environmental suitability we past projected our best model for the current climatic configuration per species to four distinct time slices that ranged across contrasting climatic periods: 1) Pleistocene: Last Interglacial (ca. 130 ka), 2) Pleistocene: Last Glacial Maximum (ca. 21 ka), 3) mid-Holocene: Northgrippian (8.326-4.2 ka), and 4) late-Holocene: Meghalayan (4.2-0.3 ka). All four paleoclimatic data were retrieved from http://www.paleoclim.org/ with a 2.5 minutes spatial resolution [12]. To remove extrapolation from the projected SDMs generated by combinations of climatic variables not represented by the training dataset, we used a multivariate environmental similarity surface (MESS) analysis [13]. The MESS analysis indicates the locations with non-analogous habitats in relation to the training points [13,14]. Only pixels with values > 0 were retained, removing any extrapolation from the models. All present and past projections were also converted to binary presence-absence using the 10th percentile training presence threshold for each taxon [10]. Differences in occupied environments between tropical and subtropical mountain regions were tested with a multidimensional ordination of the climate occupied by populations occurring in these two regions. Values of the CHELSA bioclimatic variables at 30 seconds resolution were extracted from occurrence localities "raster" [15] in R. Variables were normalized and submitted to a PCA with "prcomp" in R. Scores of the first four components, representing on average 96,6% of the total variation (average across all species) were used to compute a per-species multidimensional volume of the occupied climatic space using "hypervolume" [16] in R. We performed a total of 1000 replicates of the algorithm for each species. Finally, we calculated the Euclidean geographic distance among species within a polygon matching the distribution of the Atlantic Forest proposed by IBGE (ftp://geoftp.ibge.gov.br/mapas/tematicos/mapas_murais/biomas.pdf).

Testing predictors for the spatial variation in population differentiation

We assessed the individual and the combined effects of landscape resistance layers on genetic distance using ResistanceGA v4.0 [17] in R. To evaluate the relative support for different resistance layers, ResistanceGA fits a linear mixed effect model using a maximum likelihood population effect parametrization to account for spatial autocorrelation within pairwise distance

matrices. Nei's genetic distance was used as the response variable and the scaled and transformed landscape layers were used as predictor variables including all possible combinations of resistance matrices. Given that geographic distance is embedded in resistance layers, we did not include its interaction with other variables, exploring its relative importance alone. All species were sampled for at least five localities. Model selection was performed using AIC values from the linear mixed effect models. Optimization of the model proceeded until no further improvement on AIC was observed after 25 consecutive iterations. We applied a monomolecular transformation and the "nlm" option to finetune parameter-estimates in the final optimization, with other options set as default. After model optimization, we conducted 1,000 bootstrap replicates. This approach subsampled the pairwise predictor and response distance matrix (without replacement) and refit the MLPE model for each replicate. The frequency that a model is selected as the best among replicates was used to assess the level of support for each surface. Model optimization and bootstrapping were performed using the "all_comb" function. Given that our sampling scheme has in general a single individual per locality, the use of the genetic distance between individuals was more adequate than F_{ST} and related estimates, which can be biased when the number of samples per locality is low [18].

The maximum likelihood population effect model (MLPE) accounted for the correlated nature of pairwise distance matrices, species-specific variation in genetic differentiation, and taxonomy (represented by the family of each species) as random effects. Here we used the optimized resistance matrices obtained in ResistanceGA as predictor variables. We additionally included the effects of species-specific traits as the Kipp's Index [19] as a proxy for individual dispersal ability, habitat preference described as the major environments occupied by the species (e.g., forest edge, bamboo stands, and cloud forest; [20]), and the divergence time between populations, representing the minimum time species occurred in both regions.

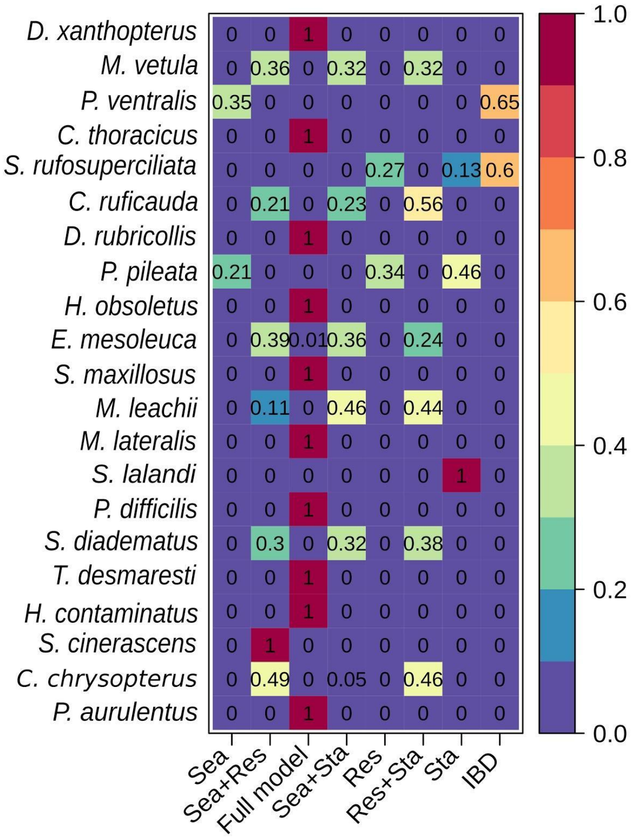

Supplementary Fig. 1: Heat map summarizing AIC relative contribution of single and combined landscape resistance layers on genetic dissimilarity for 21 species of birds restricted to the Atlantic Forest sky islands. Sea - Seasonality; Res - Environmental resistance; Sta - Historical stability; IBD - Isolation by distance.

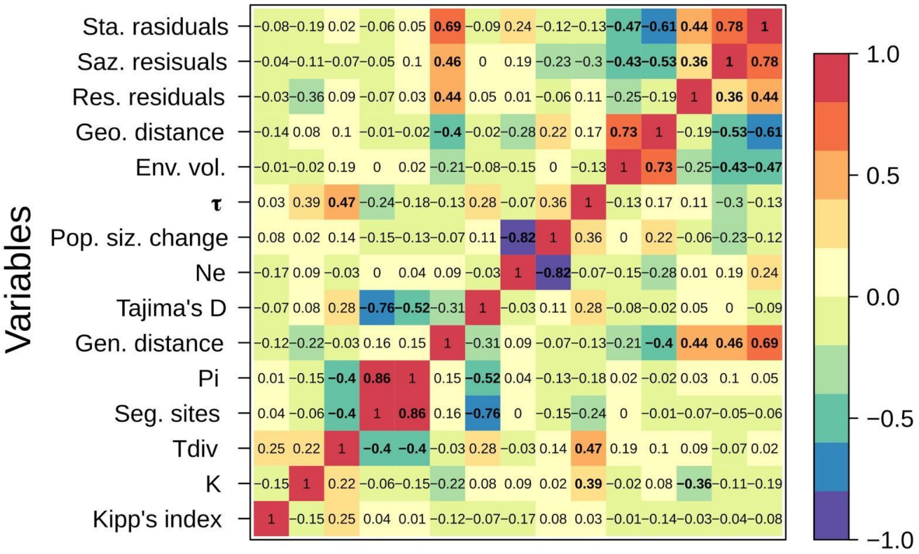

Supplementary Fig. 2: Spearman's correlation (two-sided) index between variables analyzed with Phylogenetic Generalized Least Squares (PGLS). The numbers in the cells represent the correlation index. Values in bold have p-values < 0.05. K - Number of inferred populations; Tdiv - Divergence time between populations in the two mountain regions; Seg. sites - Average number of segregating sites; Pi - Average nucleotide diversity; Gen. distance - The normalized average pairwise genetic distance between individuals; Ne - Effective population size; Pop. siz. change - the proportion of the ancestral population size compared to the current size; **τ** - Time since population size change; Env. vol. - Multidimensional environment space; Geo. distance - Average geographic distance between individuals; Res. residuals - an average of the residuals of linear regression with environmental resistance in the function of geographic distance between individuals; Saz. residuals - an average of the residuals of linear regression with seasonality resistance in the function of geographic distance between individuals; Sta. residuals - an average of the residuals of linear regression with the stability resistance in the function of geographic distance between individuals.

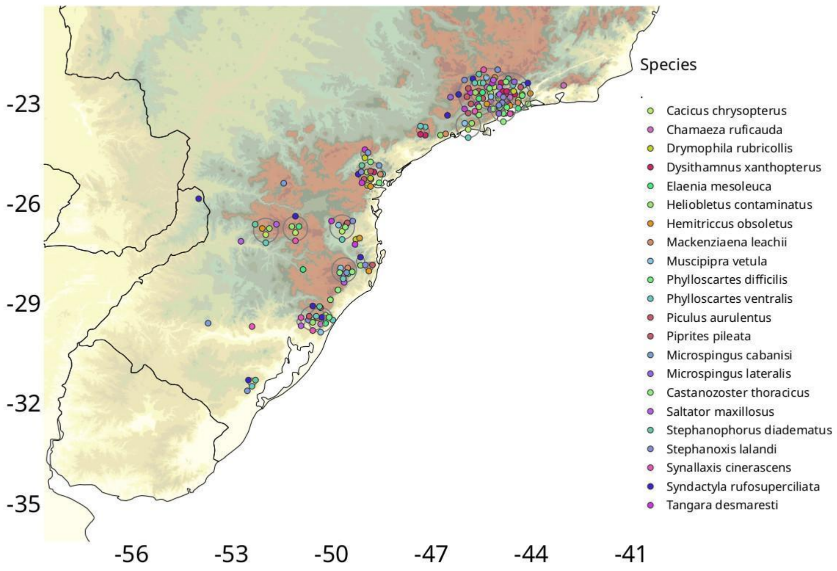

Supplementary Fig. 3: Sampled localities for our genomic approach for 21 species of birds restricted to the Atlantic Forest sky islands showing the elevation profile of the region in meters (m; Geospatial Information Authority of Japan).

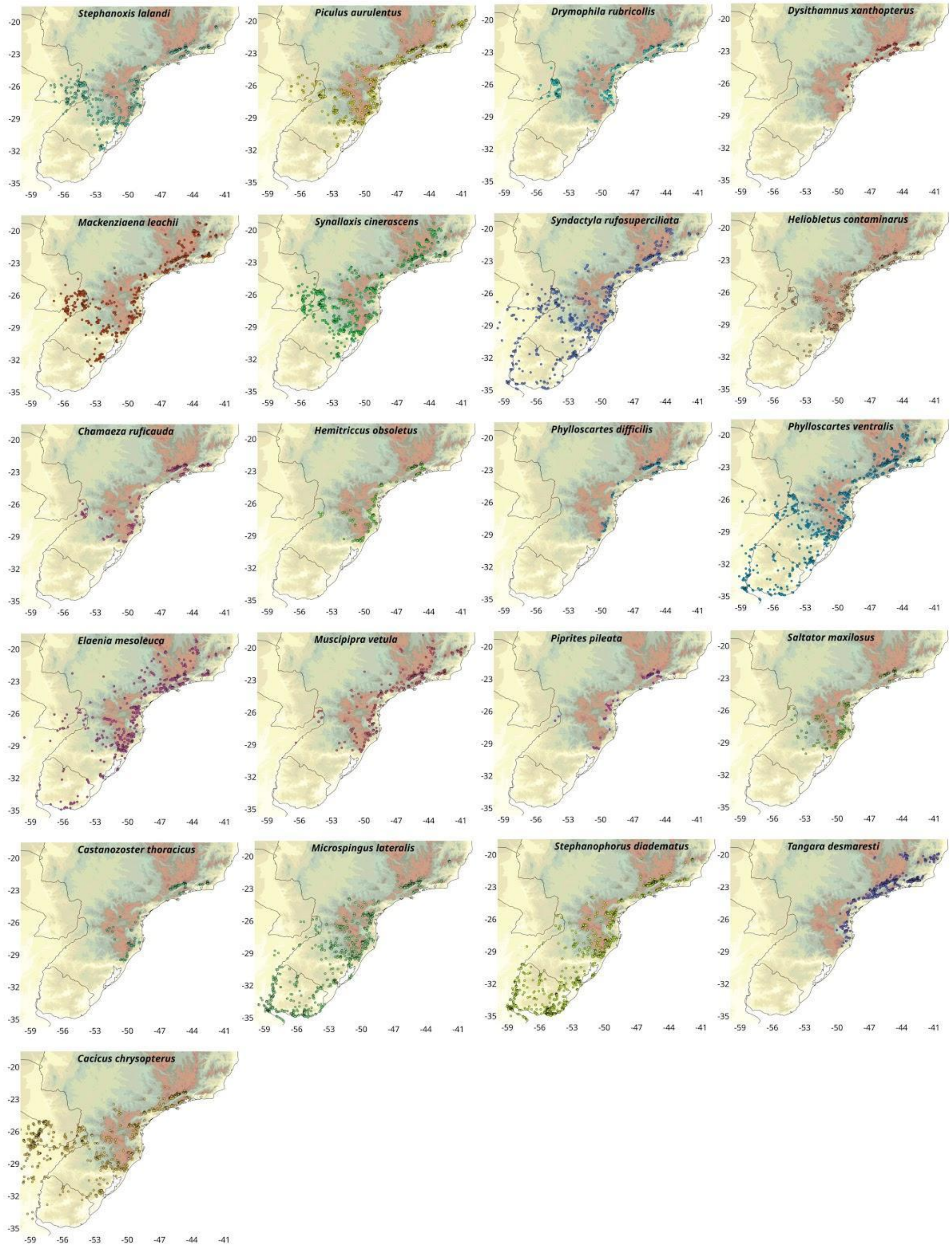

Supplementary Fig. 4: Occurrence records obtained for 21 species of birds restricted to the Atlantic Forest sky islands showing the elevation profile of the region in meters (m; Geospatial Information Authority of Japan).

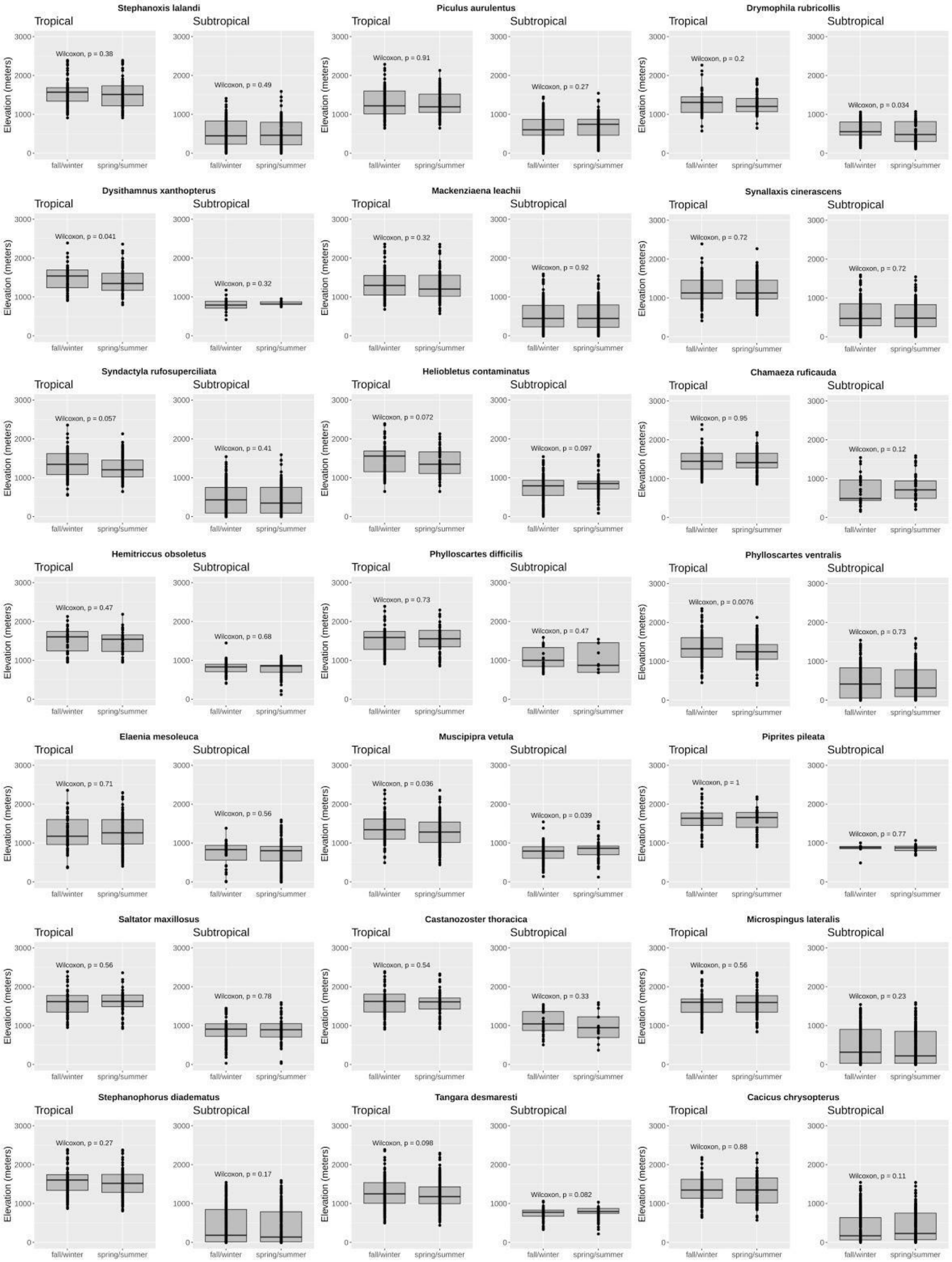

Stephanoxis lalandi
Tropical
Wilcoxon, p = 0.38
Subtropical
Wilcoxon, p = 0.49
Piculus aurulentus
Tropical
Wilcoxon, p = 0.91
Subtropical
Wilcoxon, p = 0.27
Drymophila rubricollis
Tropical
Wilcoxon, p = 0.2
Subtropical
Wilcoxon, p = 0.034
Dysithamnus xanthopterus
Tropical
Wilcoxon, p = 0.041
Subtropical
Wilcoxon, p = 0.32
Mackenziaena leachii
Tropical
Wilcoxon, p = 0.32
Subtropical
Wilcoxon, p = 0.92
Synallaxis cinerascens
Tropical
Wilcoxon, p = 0.72
Subtropical
Wilcoxon, p = 0.72
Syndactyla rufosuperciliata
Tropical
Wilcoxon, p = 0.057
Subtropical
Wilcoxon, p = 0.41
Heliobletus contaminatus
Tropical
Wilcoxon, p = 0.072
Subtropical
Wilcoxon, p = 0.097
Chamaeza ruficauda
Tropical
Wilcoxon, p = 0.95
Subtropical
Wilcoxon, p = 0.12
Hemitriccus obsoletus
Tropical
Wilcoxon, p = 0.47
Subtropical
Wilcoxon, p = 0.68
Phylloscartes difficilis
Tropical
Wilcoxon, p = 0.73
Subtropical
Wilcoxon, p = 0.47
Phylloscartes ventralis
Tropical
Wilcoxon, p = 0.0076
Subtropical
Wilcoxon, p = 0.73
Elaenia mesoleuca
Tropical
Wilcoxon, p = 0.71
Subtropical
Wilcoxon, p = 0.56
Muscipipra vetula
Tropical
Wilcoxon, p = 0.036
Subtropical
Wilcoxon, p = 0.039
Piprites pileata
Tropical
Wilcoxon, p = 1
Subtropical
Wilcoxon, p = 0.77
Saltator maxillosus
Tropical
Wilcoxon, p = 0.56
Subtropical
Wilcoxon, p = 0.78
Castanozoster thoracica
Tropical
Wilcoxon, p = 0.54
Subtropical
Wilcoxon, p = 0.33
Microspingus lateralis
Tropical
Wilcoxon, p = 0.56
Subtropical
Wilcoxon, p = 0.23
Stephanophorus diadematus
Tropical
Wilcoxon, p = 0.27
Subtropical
Wilcoxon, p = 0.17
Tangara desmaresti
Tropical
Wilcoxon, p = 0.098
Subtropical
Wilcoxon, p = 0.082
Cacicus chrysopterus
Tropical
Wilcoxon, p = 0.88
Subtropical
Wilcoxon, p = 0.11
Elevation (meters)
0
1000
2000
3000
fall/winter
spring/summer

Supplementary Fig. 5: Elevational distribution of 21 species of birds restricted to the Atlantic Forest sky islands. To test for the potential effect of elevational migration, occurrences were clustered by warm (Spring/Summer) and cold (Fall/Winter) months. n=13,986 occurrence records average across species; mean = 608.1 and SD= 371.3 per species. The boxplots are composed by the median (cetre), the first and third quartiles (lower and upper hinges), and the 1.5 * IQR from the hinge (where IQR is the interquartile range; upper and lower whiskers). P-values were obtained with a two-sided nonparametric Wilcoxon test. Source data is provided in Supplementary Data 3.

Supplementary Table 1: Mixed-effects model selection results testing the relative contribution of seasonality and local elevation on four dependent variables associated with species elevational distribution. *** p-value > 0.001 on a likelihood ratio test between the two models with the highest AIC weight.

Dependent variables	seasonality		Available elevation		Full model	
	AICc	weight	AICc	weight	AICc	weight
Occupied proportion of the elevational gradient	1010.6	1.42E-12	1046.6	2.13E-20	956.0 ***	1.00E+00
Elevational range	1763.7	2.23E-37	1693.0	5.06E-22	1594.9***	1.00E+00
Minimum elevation	1559.7***	7.56E-01	1692.3	1.18E-29	1561.9	2.44E-01
Maximum elevation	1767.133	3.56E-40	1585.9***	8.03E-01	1588.7	1.97E-01

Supplementary Table 2: Best models for four dependent variables tested to explore the relative effects of topography and seasonality on the elevation distribution of Atlantic forest montane species based on linear mixed effect models.

Dependent variable	Variables	Value	Std.Error	DF	t-value	p-value	R2c
The occupied proportion of the elevational gradient (full model)	intercept	50.4	1.57	105	32.10	< 0.001	0.57
	available elevation	14.5	1.98	105	7.30	< 0.001	
	seasonality	19.8	1.93	105	10.25	< 0.001	
	available elevation:seasonality	-0.1	1.07	105	-0.14	0.891	

Elevational range (full model)	intercept	706.2	23.17	105	30.47	< 0.001	0.78
	available elevation	455.6	26.91	105	16.93	< 0.001	
	seasonality	295.5	26.19	105	11.28	< 0.001	
	available elevation:seasonality	-15.1	14.55	105	-1.04	0.3019	
Minimum elevation (seasonality)	intercept	404.3	24.42	107	16.56	< 0.001	0.87
	seasonality	-317.9	12.39	107	-25.66	< 0.001	
Maximum elevation (available elevation)	intercept	1120.2	21.96	107	51.01	< 0.001	0.91
	available elevation	475.5	14.41	107	33.00	< 0.001	

Supplementary Table 3: Summary results of ResistanceGA analyses.

Species	Surface	k	AICc	R2m	LL	AIC weight
Piculus aurulentus	Full model	13	-667.54	0.60	255.77	>0.99
Cacicus chrysopterus	Seasonality + Resistance	9	-422.20	0.68	130.10	0.49
Synallaxis cinerascens	Full model	13	-389.18	0.71	162.09	>0.99
Heliobletus contaminatus	Full model	13	-538.91	0.29	221.79	>0.99
Tangara desmaresti	Full model	13	-814.70	0.06	329.35	>0.99
Stephanophorus diadematus	Resistance + Stability	9	-437.57	0.34	137.79	0.38
Phylloscartes difficilis	Full model	13	-501.19	0.70	202.93	>0.99
Stephanoxis lalandi	Stability	5	-853.64	1.00	435.57	>0.99
Microspingus lateralis	Full model	13	-723.03	0.44	283.52	>0.99
Mackenziaena leachii	Seasonality + Stability	9	-203.77	0.98	80.88	0.46
Saltator maxillosus	Full model	13	-377.53	0.42	156.26	>0.99
Elaenia mesoleuca	Seasonality + Resistance	9	-169.57	0.51	71.28	0.39
Hemitriccus obsoletus	Full model	13	-834.77	0.76	248.39	>0.99
Piprites pileata	Stability	5	-118.64	0.82	34.32	0.46
Drymophila rubricollis	Full model	13	-633.07	0.15	238.54	>0.99

Chamaeza ruficauda	Resistance + Stability	9	-390.85	0.86	114.42	0.56
Syndactyla rufosuperciliata	IBD	2	-1000.65	0.39	502.79	0.60
Castanozoster thoracica	Full model	13	-538.12	0.92	191.06	>0.99
Phylloscartes ventralis	IBD	2	-731.29	0.13	368.19	0.65
Muscipipra vetula	Seasonality + Resistance	9	-207.40	0.32	82.70	0.36
Dysithamnus xanthopterus	Full model	13	-334.57	0.62	134.78	>0.99

Supplementary Table 4: Best models for the maximum likelihood population effect analyses based on linear mixed effect models.

Groups	Variables	Value	Std.Error	DF	t-value	p-value
All samples – Full model	(Intercept)	0.72	0.05	1058.00	14.89	< 0.001
	IBD	0.17	0.02	1058.00	8.32	< 0.001
	Resistance	0.05	0.01	1058.00	7.22	< 0.001
	Seasonality	0.00	0.01	1058.00	-0.41	0.6813
	Stability	0.05	0.01	1058.00	6.99	< 0.001
	TDIV	0.04	0.02	12.00	1.86	0.0872
	Kipps index	-0.01	0.02	12.00	-0.58	0.5728
	Environment – Canopy	-0.14	0.06	12.00	-2.10	0.0575
	Environment – Edge	-0.12	0.07	12.00	-1.77	0.1019
	Environment – Midstory	-0.23	0.07	12.00	-3.06	0.0100
	Environment – Understory	-0.04	0.06	12.00	-0.66	0.5188
Tropical mountains - Full model	(Intercept)	0.59	0.10	249.00	5.81	< 0.001
	IBD	0.05	0.01	249.00	3.34	0.0010
	Resistance	0.04	0.01	249.00	4.96	< 0.001
	Seasonality	0.00	0.02	249.00	0.17	0.8672
	Stability	-0.03	0.02	249.00	-1.93	0.0544
	TDIV	0.02	0.04	12.00	0.44	0.6702
	Kipps index	-0.09	0.05	12.00	-1.91	0.0800

	Environment – Canopy	0.03	0.13	12.00	0.26	0.8015
	Environment – Edge	-0.36	0.15	12.00	-2.45	0.0305
	Environment – Midstory	0.22	0.17	12.00	1.30	0.2164
	Environment – Understory	-0.05	0.12	12.00	-0.38	0.7129
Subtropical mountains – IBD model	(Intercept)	0.39	0.07	222.00	5.32	< 0.001
	IBD	0.06	0.01	222.00	4.41	< 0.001
	TDIV	-0.01	0.03	12.00	-0.43	0.6738
	Kipps index	-0.02	0.03	12.00	-0.74	0.4741
	Environment – Canopy	-0.03	0.10	12.00	-0.32	0.7553
	Environment – Edge	0.07	0.10	12.00	0.70	0.4990
	Environment – Midstory	-0.13	0.10	12.00	-1.28	0.2260
	Environment – Understory	-0.15	0.08	12.00	-1.77	0.1017

Supplementary Table 5: Means and standard deviations of parameters estimated with a bidimensional stepping stone coalescent modeling (BSSM) approach with migrations scaled by the linear geographic distance between sampled localities (IBD-BSSM).

Species	Pop	Noise	SD Noise	Dispersion (km/generation)	SD Dispersion (km/generation)	Average Migration	SD Migration
Piculus aurulentus	Subtropical	0.9	0.08	157,389	2,630	0.86	0.01
Piculus aurulentus	Tropical	0.72	0.11	81,250	4,810	1.67	0.1
Cacicus chrysopterus	Subtropical	0.95	0.11	93,994	1,239	0.45	0.01
Cacicus chrysopterus	Tropical	0.9	0.14	79,495	2,210	1.01	0.03
Synallaxis cinerascens	Subtropical	0.54	0.12	90,098	543	0.44	0
Synallaxis cinerascens	Tropical	0.95	0.08	89,163	1,643	1.4	0.03
Heliobletus contaminatus	Subtropical	0.8	0.12	124,104	2,248	0.45	0.01
Heliobletus contaminatus	Tropical	0.95	0.04	80,450	805	0.84	0.01
Tangara desmaresti	Subtropical	0.82	0.09	124,799	4,767	0.76	0.03
Tangara desmaresti	Tropical	0.64	0.05	107,325	3,173	1.67	0.05

Stephanophorus diadematus	Subtropical	0.72	0.06	164,094	1,907	0.42	0
Stephanophorus diadematus	Tropical	1.02	0.4	123,435	6,761	3.59	0.2
Stephanoxis lalandi	Subtropical	0.99	0.13	112,357	1,591	0.61	0.01
Stephanoxis lalandi	Tropical	0.73	0.4	83,950	3,110	1.61	0.06
Microspingus lateralis	Subtropical	0.96	0.09	121,793	1,748	0.4	0.01
Microspingus lateralis	Tropical	0.9	0.04	76,948	2,272	1.19	0.04
Saltator maxillosus	Subtropical	0.8	0.45	107,216	1,348	0.59	0.01
Saltator maxillosus	Tropical	0.87	0.04	74,164	37,781	2.1	1.07
Hemitriccus obsoletus	Subtropical	0.82	0.06	102,177	1,111	0.42	0
Hemitriccus obsoletus	Tropical	0.63	0.03	35,776	1,485	0.58	0.02
Chamaeza ruficauda	Subtropical	1.04	0.15	65,618	2,703	0.51	0.02
Chamaeza ruficauda	Tropical	0.77	0.02	60,356	1,114	0.81	0.01
Syndactyla rufosuperciliata	Subtropical	0.96	0.11	164,345	5,791	0.5	0.02
Syndactyla rufosuperciliata	Tropical	0.68	0.07	126,858	3,044	1.33	0.03
Castanozoster thoracica	Subtropical	0.86	0.09	101,624	2,125	0.82	0.02
Castanozoster thoracica	Tropical	0.98	0.21	51,063	3,463	1.54	0.1
Phylloscartes ventralis	Subtropical	0.84	0.1	107,864	54,351	0.63	0.32
Phylloscartes ventralis	Tropical	0.82	0.04	101,799	958	1.35	0.01
Muscipipra vetula	Subtropical	0.92	0.15	96,137	1,889	0.45	0.01
Muscipipra vetula	Tropical	0.89	0.14	86,110	1,811	1.19	0.02
Dysithamnus xanthopterus	Subtropical	0.48	0.06	73,790	271	0.5	0
Dysithamnus xanthopterus	Tropical	0.4	0.06	85,367	335	1.44	0.01

Supplementary Table 6: Means and standard deviations for parameters estimated with a bidimensional stepping stone coalescent modeling (BSSM) approach with a single value assigned to all migration parameters (Island-BSSM).

Species	Pop	Noise	SD Noise	Dispersion (km/generation)	SD Dispersion (km/generation)	Average Migration	SD Migration

Piculus aurulentus	Subtropical	0.78	0.06	239,962	16,317	1.32	0.09
Piculus aurulentus	Tropical	0.76	0.05	84,266	7,813	1.74	0.16
Cacicus chrysopterus	Subtropical	0.73	0.12	229,711	8,662	1.09	0.04
Cacicus chrysopterus	Tropical	0.82	0.26	157,337	7,373	1.99	0.09
Synallaxis cinerascens	Subtropical	0.56	0.1	233,680	11,856	1.15	0.06
Synallaxis cinerascens	Tropical	0.68	0.08	66,720	8,828	1.05	0.14
Heliobletus contaminatus	Subtropical	0.8	0.09	302,009	16,072	1.1	0.06
Heliobletus contaminatus	Tropical	0.78	0.06	71,904	3,748	0.75	0.04
Tangara desmaresti	Subtropical	0.9	0.11	186,377	13,484	1.13	0.08
Tangara desmaresti	Tropical	0.38	0.12	102,100	1,934	1.59	0.03
Stephanophorus diadematus	Subtropical	0.5	0.14	196,865	8,287	0.5	0.02
Stephanophorus diadematus	Tropical	0.89	0.15	42,491	6,693	1.24	0.19
Stephanoxis lalandi	Subtropical	0.99	0.12	152,874	16,810	0.84	0.09
Stephanoxis lalandi	Tropical	0.62	0.01	102,804	5,033	1.97	0.1
Microspingus lateralis	Subtropical	0.92	0.49	193,757	12,734	0.63	0.04
Microspingus lateralis	Tropical	0.74	0.16	48,156	4,770	0.74	0.07
Saltator maxillosus	Subtropical	0.68	0.11	288,194	8,501	1.57	0.05
Saltator maxillosus	Tropical	0.53	0.1	49,174	1,779	1.39	0.05
Hemitriccus obsoletus	Subtropical	0.7	0.07	138,378	2,650	0.56	0.01
Hemitriccus obsoletus	Tropical	0.86	0.03	37,739	5,013	0.61	0.08
Chamaeza ruficauda	Subtropical	0.91	0.14	132,848	7,048	1.03	0.05
Chamaeza ruficauda	Tropical	0.86	0.11	38,310	2,090	0.52	0.03
Syndactyla rufosuperciliata	Subtropical	0.8	0.09	167,598	4,035	0.51	0.01
Syndactyla rufosuperciliata	Tropical	0.82	0.2	189,389	11,760	1.99	0.12
Castanozoster thoracica	Subtropical	0.78	0.23	216,172	12,146	1.74	0.1
Castanozoster thoracica	Tropical	0.64	0.16	54,932	5,455	1.66	0.16
Phylloscartes ventralis	Subtropical	0.8	0.09	327,814	26,216	1.91	0.15

Phylloscartes ventralis	Tropical	0.88	0.05	163,462	8,771	2.16	0.12
Muscipipra vetula	Subtropical	0.66	0.03	364,713	2,904	1.72	0.01
Muscipipra vetula	Tropical	0.66	0.08	131,393	4,141	1.81	0.06
Dysithamnus xanthopterus	Subtropical	0.72	0.22	91,837	3,381	0.62	0.02
Dysithamnus xanthopterus	Tropical	0.52	0.24	145,636	73,206	2.45	1.23

Supplementary Table 7: Estimated R-squared (R^2) for correlations between pseudo-observed and estimated effective population size (*Ne*), and effective dispersion rate (*De*) for 16 species of birds with populations occurring in the Tropical and Subtropical mountains of the Atlantic Forest. Coalescent simulations were performed under a bidimensional stepping stone model (BSSM). Results are shown for a model with migrations between localities scaled by the linear geographic distance between localities (IBD-BSSM) and for a model where migrations were not scaled by distances (Island-BSSM) - see methods for more details). Estimated values were obtained with a Neural Network approach.

Species	Parameter	IBD-BSSM Tropical	IBD-BSSM Subtropical	Island-BSSM Tropical	Island-BSSM Subtropical
Piculus aurulentus	Ne	0.95	0.94	0.95	0.96
	De	0.90	0.92	0.89	0.85
Cacicus chrysopterus	Ne	0.95	0.93	0.95	0.95
	De	0.91	0.93	0.79	0.78
Synallaxis cinerascens	Ne	0.95	0.94	0.96	0.95
	De	0.93	0.93	0.84	0.78
Heliobletus contaminatus	Ne	0.95	0.94	0.97	0.97
	De	0.92	0.96	0.85	0.88
Tangara desmaresti	Ne	0.98	0.97	0.97	0.97
	De	0.90	0.93	0.90	0.89
Stephanophorus diadematus	Ne	0.95	0.93	0.95	0.96
	De	0.85	0.93	0.81	0.74
Stephanoxis lalandi	Ne	0.97	0.96	0.97	0.97
	De	0.93	0.96	0.91	0.89
Microspingus	Ne	0.95	0.95	0.97	0.97

lateralis	*De*	0.94	0.95	0.91	0.88
Saltator maxillosus	Ne	0.94	0.94	0.96	0.95
	De	0.87	0.94	0.85	0.83
Hemitriccus obsoletus	Ne	0.98	0.95	0.97	0.97
	De	0.94	0.94	0.90	0.85
Chamaeza ruficauda	Ne	0.95	0.93	0.96	0.95
	De	0.91	0.92	0.80	0.77
Syndactyla rufosuperciliat a	Ne	0.98	0.96	0.98	0.97
	De	0.92	0.96	0.88	0.88
Castanozoster thoracica	Ne	0.94	0.95	0.96	0.96
	De	0.89	0.94	0.84	0.85
Phylloscartes ventralis	Ne	0.95	0.96	0.95	0.95
	De	0.92	0.96	0.82	0.88
Muscipipra vetula	Ne	0.93	0.90	0.94	0.93
	De	0.90	0.89	0.75	0.66
Dysithamnus xanthopterus	Ne	0.94	0.93	0.94	0.92
	De	0.85	0.86	0.71	0.65

Supplementary Table 8: Estimated values of Blomberg’s K for 15 continuous variables. P-values were obtained based on 1000 permutations.

Variable	Blomberg's K	p-value
Kipp’s index	0.0576	0.433
Number of populations (# of conStruct layers)	0.0613	0.357
Tdiv	0.0430	0.919
Seg. sites	0.0680	0.19
Pi	0.0722	0.157
Gen. distance	0.0688	0.178
Tajima’s D	0.0481	0.781

Ne	0.0690	0.187
Pop. siz. change	0.0446	0.891
**τ**	0.0415	0.959
Env. vol.	0.0537	0.58
Geo. distance	0.1169	0.008
Res. residuals	0.2533	0.001
Saz. residuals	0.0659	0.185
Sta. residuals	0.1267	0.004

Tdiv - divergence time between populations in the two mountain regions; Seg. sites - Average number of segregating sites; Pi - Average nucleotide diversity; Gen. distance - The normalized average pairwise genetic distance between individuals; Ne - Effective population size; Pop. siz. Change - the proportion of the ancestral population size compared to the current size; **τ** - Time since population size change; Env. vol. - Multidimensional environment space; Geo. distance - Average geographic distance between individuals; Res. residuals - an average of the residuals of linear regression with environmental resistance in the function of geographic distance between individuals; Saz. residuals - an average of the residuals of linear regression with seasonality resistance in the function of geographic distance between individuals; Sta. residuals - an average of the residuals of linear regression with the stability resistance in the function of geographic distance between individuals.

Supplementary Table 9: Layer (clusters) contribution to the explained variation for spatial and non-spatial ConStruct models for 21 species of birds with populations occurring in the tropical and subtropical mountains of the Atlantic Forest. Layers with a relative contribution >5% to the total covariance of the model were considered significant.

	Nonspatial			Spatial		
Species	Layer 1	Layer 2	K	Layer 1	Layer 2	K
Piculus aurulentus	0.48	0.52	2	0.64	0.36	2
Cacicus chrysopterus	0.94	0.06	2	0.07	0.93	2
Synallaxis cinerascens	0.66	0.34	2	0.45	0.55	2
Heliobletus contaminatus	0.97	0.03	1	0.00	1.00	1
Tangara desmaresti	1.00	0.00	1	0.00	1.00	1
Stephanophorus diadematus	0.99	0.01	1	1.00	0.00	1
Phylloscartes difficilis	0.63	0.37	2	0.99	0.01	1
Stephanoxis lalandi	0.36	0.64	2	0.63	0.37	2
Microspingus lateralis	0.51	0.49	2	0.31	0.69	2
Mackenziaena leachii	0.19	0.81	2	0.06	0.94	2
Saltator maxillosus	0.80	0.20	2	0.01	0.99	1
Elaenia mesoleuca	1.00	0.00	1	1.00	0.00	1

Hemitriccus obsoletus	0.49	0.51	2	0.63	0.37	2
Piprites pileata	0.49	0.51	2	1.00	0.00	1
Drymophila rubricollis	0.65	0.35	2	0.00	1.00	1
Chamaeza ruficauda	0.62	0.38	2	1.00	0.00	1
Syndactyla rufosuperciliata	0.73	0.27	2	0.00	1.00	1
Castanozoster thoracica	0.39	0.61	2	0.62	0.38	2
Phylloscartes ventralis	1.00	0.00	1	0.00	1.00	1
Muscipipra vetula	0.99	0.01	1	0.99	0.01	1
Dysithamnus xanthopterus	0.76	0.24	2	0.03	0.97	1

References

1. Eaton, D. A. R. & Overcast, I. ipyrad: Interactive assembly and analysis of RADseq datasets. *Bioinformatics* (2016).
2. Aiello-Lammens, M. E., Boria, R. A., Radosavljevic, A., Vilela, B. & Anderson, R. P. spThin: an R package for spatial thinning of species occurrence records for use in ecological niche models. *Ecography* **38**, 541–545 (2015).
3. Karger, D. N. *et al.* Climatologies at high resolution for the earth's land surface areas. *Sci Data* **4**, 170122 (2017).
4. Phillips, S. J. & Dudík, M. Modeling of species distributions with Maxent: new extensions and a comprehensive evaluation. *Ecography* **31**, 161–175 (2008).
5. Elith, J. *et al.* A statistical explanation of MaxEnt for ecologists. *Diversity and distributions* **17**, 43–57 (2011).
6. Peterson, A. T. *et al.* Ecological Niches and Geographic Distributions (MPB-49). (2011) doi:10.23943/princeton/9780691136868.001.0001.
7. Radosavljevic, A. & Anderson, R. P. Making better Maxent models of species distributions:

complexity, overfitting and evaluation. *J. Biogeogr.* **41**, 629–643 (2014).

8. Muscarella, R. *et al.* ENMeval: An R package for conducting spatially independent evaluations and estimating optimal model complexity for Maxent ecological niche models. *Methods Ecol. Evol.* **5**, 1198–1205 (2014).

9. Galante, P. J. *et al.* The challenge of modeling niches and distributions for data-poor species: a comprehensive approach to model complexity. *Ecography* **41**, 726–736 (2018).

10. Pearson, R. G. Species' distribution modeling for conservation educators and practitioners. *Synthesis. American Museum of Natural History* **50**, 54–89 (2007).

11. Shcheglovitova, M. & Anderson, R. P. Estimating optimal complexity for ecological niche models: A jackknife approach for species with small sample sizes. *Ecol. Modell.* **269**, 9–17 (2013).

12. Brown, J. L., Hill, D. J., Dolan, A. M., Carnaval, A. C. & Haywood, A. M. PaleoClim, high spatial resolution paleoclimate surfaces for global land areas. *Sci Data* **5**, 180254 (2018).

13. Elith, J., Kearney, M. & Phillips, S. The art of modelling range-shifting species. *Methods Ecol. Evol.* **1**, 330–342 (2010).

14. Elith, J. & Leathwick, J. R. Species Distribution Models: Ecological Explanation and Prediction Across Space and Time. *Annual Review of Ecology, Evolution, and Systematics* vol. 40 677–697 (2009).

15. Hijmans, R. J. & van Etten, J. Raster: raster: Geographic data analysis and modeling. *R package version* 2–3 (2013).

16. Blonder, B., Lamanna, C., Violle, C. & Enquist, B. J. The n-dimensional hypervolume. *Glob. Ecol. Biogeogr.* **23**, 595–609 (2014).

17. Peterman, W. E. ResistanceGA : An R package for the optimization of resistance surfaces

using genetic algorithms. *Methods Ecol. Evol.* **9**, 1638–1647 (2018).

18. Meirmans, P. G. & Hedrick, P. W. Assessing population structure: FST and related measures. *Mol. Ecol. Resour.* **11**, 5–18 (2011).
19. Kipp, F. A. Der Handflügel-Index als flugbiologisches Maß. *Vogelwarte* **20**, 77–86 (1959).
20. Stotz, D. F., Fitzpatrick, J. W., Parker, T. A., III & Moskovits, D. K. Neotropical birds: ecology and conservation. (1996).
