# Supplementary material for: Microevolutionary dynamics show tropical valleys are deeper for montane birds of the Atlantic Forest
Source: Nat Commun. 2021 Nov 1;12:6269. doi: 10.1038/s41467-021-26537-9 (PMC8560783; doi:10.1038/s41467-021-26537-9)
Supplement: Supplementary file 3 — Description of Additional Supplementary Files [file 41467_2021_26537_MOESM3_ESM.pdf]

Description of Additional Supplementary Files

Supplementary Data 1: Description of genetic samples for 21 species of birds occurring across the Atlantic Forest montane regions (Available as supplementary data and in Github: https://doi.org/10.5281/zenodo.5510615 (https://github.com/GregoryThom/)). FURB - Coleção Zoológica da Universidade Regional de Blumenau; LGEMA - Laboratório de Genética e Evolução Molecular de Aves, MPEG - Museu Paraense Emílio Goeldi, MCT-PUCRS - Museu de Ciências e Tecnologia da Pontifícia Universidade Católica do Rio Grande do Sul; MZUSP - Museu de Zoologia da Universidade de São Paulo.

Supplementary Data 2: Demographic parameter estimation based on a two population (Tropical and Subtropical) model for 21 species of birds restricted to the Atlantic Forest sky-islands. (Available as supplementary data and in Github: https://doi.org/10.5281/zenodo.5510615 (https://github.com/GregoryThom/))

Supplementary Data 3: Filtered occurrences retrieved from Global Biodiversity Information Facility, GBIF.org (04/28/2020; GBIF Occurrence Download DOI 10.15468/dl.8383sp; Available as supplementary data and in Github: https://doi.org/10.5281/zenodo.5510615 (https://github.com/GregoryThom/)).

Supplementary Data 4: Species distribution models (SDMs) for the current climate configurations and combined stability maps (Available as supplementary data and in Github: https://doi.org/10.5281/zenodo.5510615 (https://github.com/GregoryThom/))

Supplementary Data 5: Principal Component Analysis of observed and simulated summary statistics. Coalescent simulations were performed under a bidimensional stepping stone model; 0 - migrations were not scaled by distances; 1 migration between localities was scaled by the linear spatial distances (Available as supplementary data and in Github: https://doi.org/10.5281/zenodo.5510615 (https://github.com/GregoryThom/))
