# Supplementary figures and images for: Microevolutionary dynamics show tropical valleys are deeper for montane birds of the Atlantic Forest
Source: Nat Commun. 2021 Nov 1;12:6269. doi: 10.1038/s41467-021-26537-9 (PMC8560783; doi:10.1038/s41467-021-26537-9)

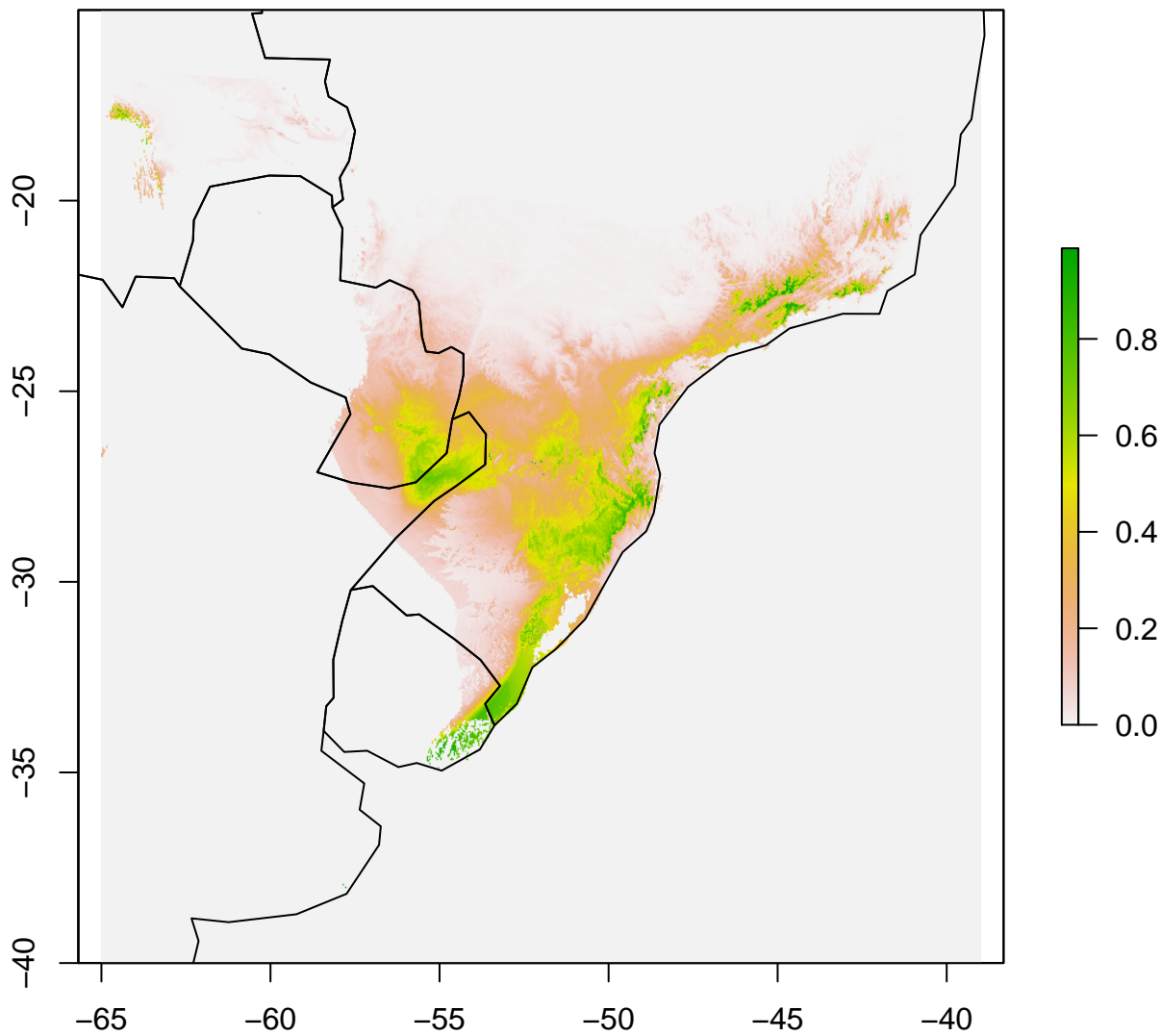

Supplement: Supplementary file 7 — Supplementary Data 4 [file 41467_2021_26537_MOESM7_ESM.gz › Dataset S1/lalandi_projection_MESS]

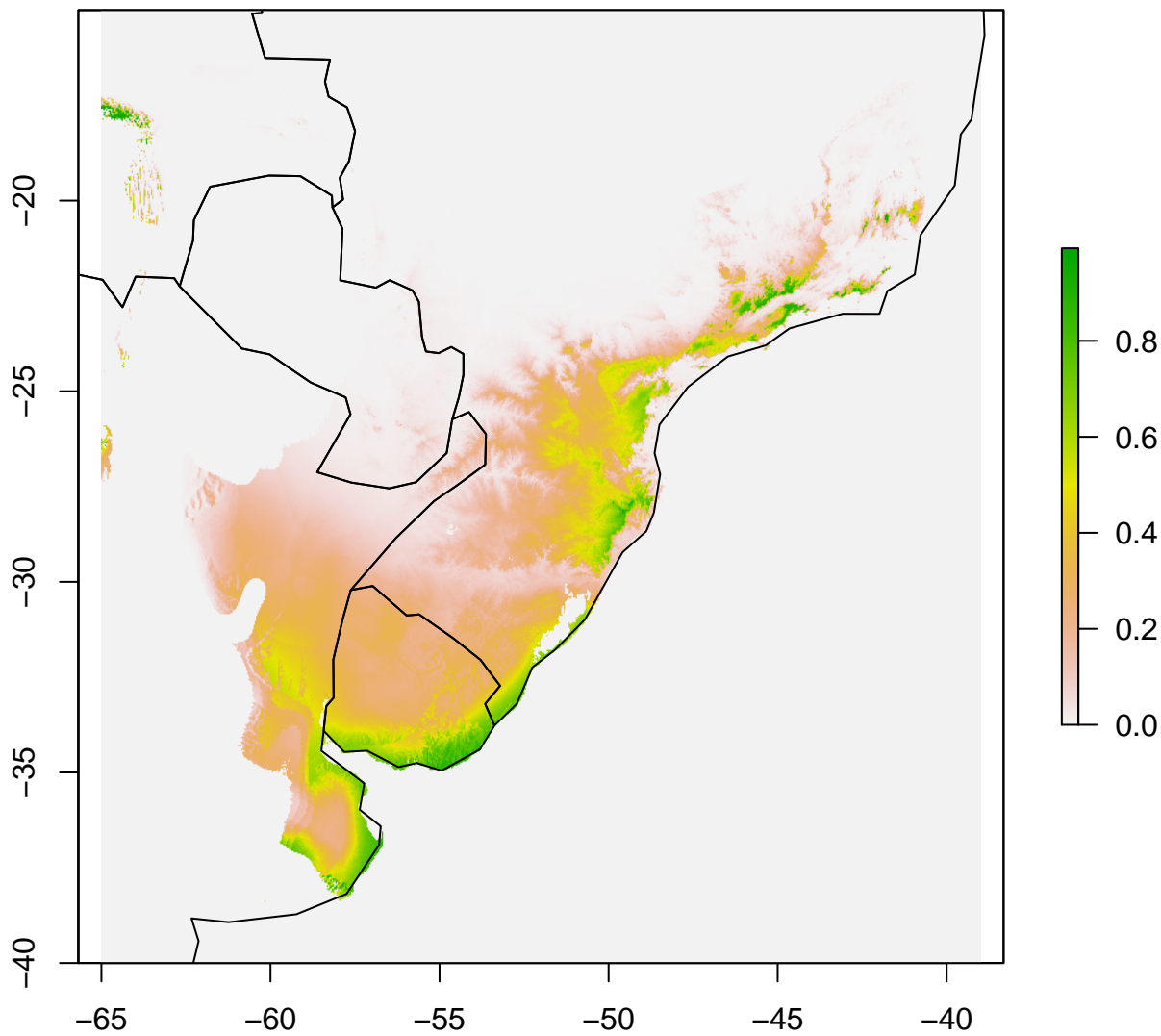

Supplement: Supplementary file 7 — Supplementary Data 4 [file 41467_2021_26537_MOESM7_ESM.gz › Dataset S1/diadematus_projection_MESS]

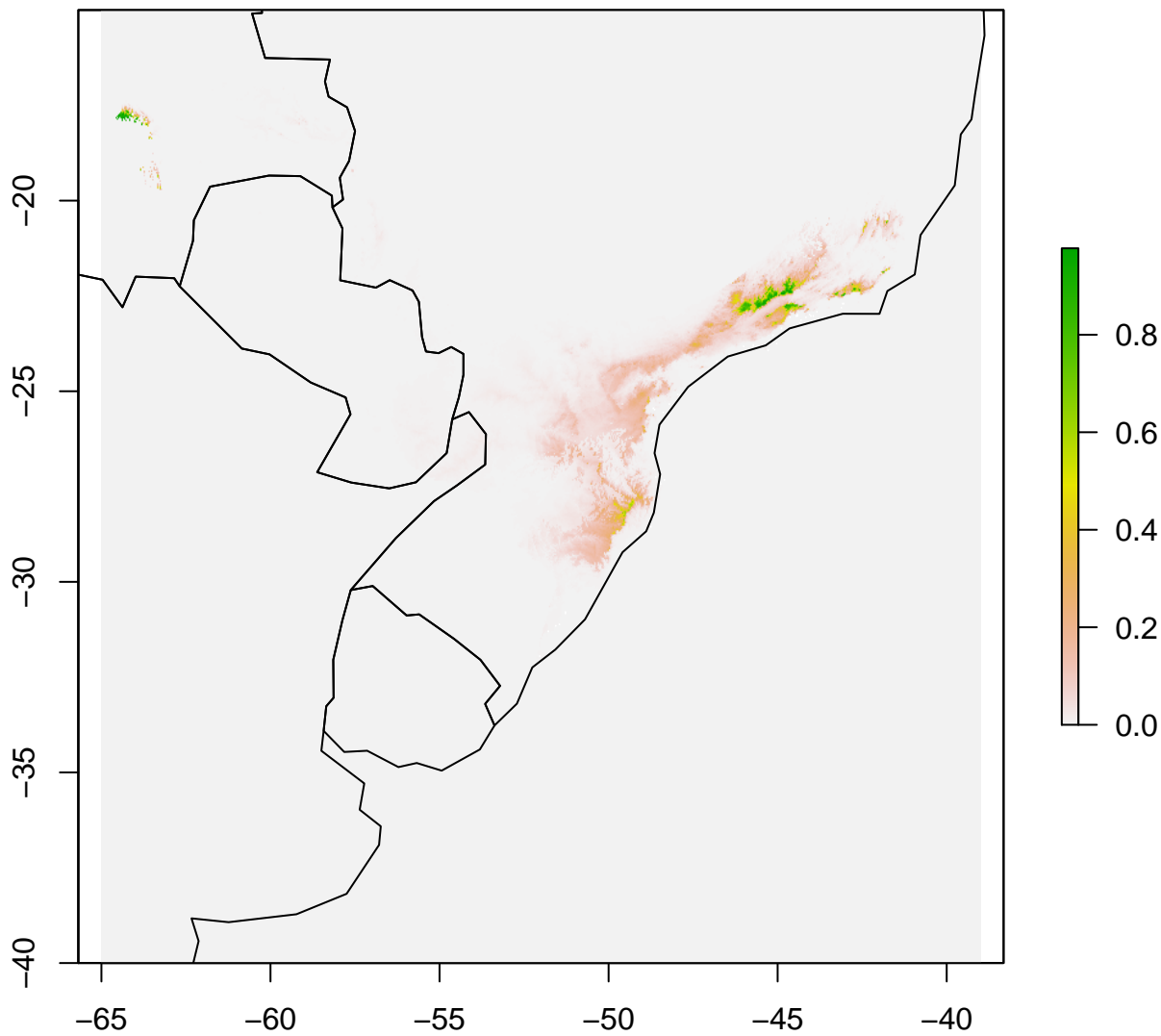

Supplement: Supplementary file 7 — Supplementary Data 4 [file 41467_2021_26537_MOESM7_ESM.gz › Dataset S1/pileata_projection_MESS]

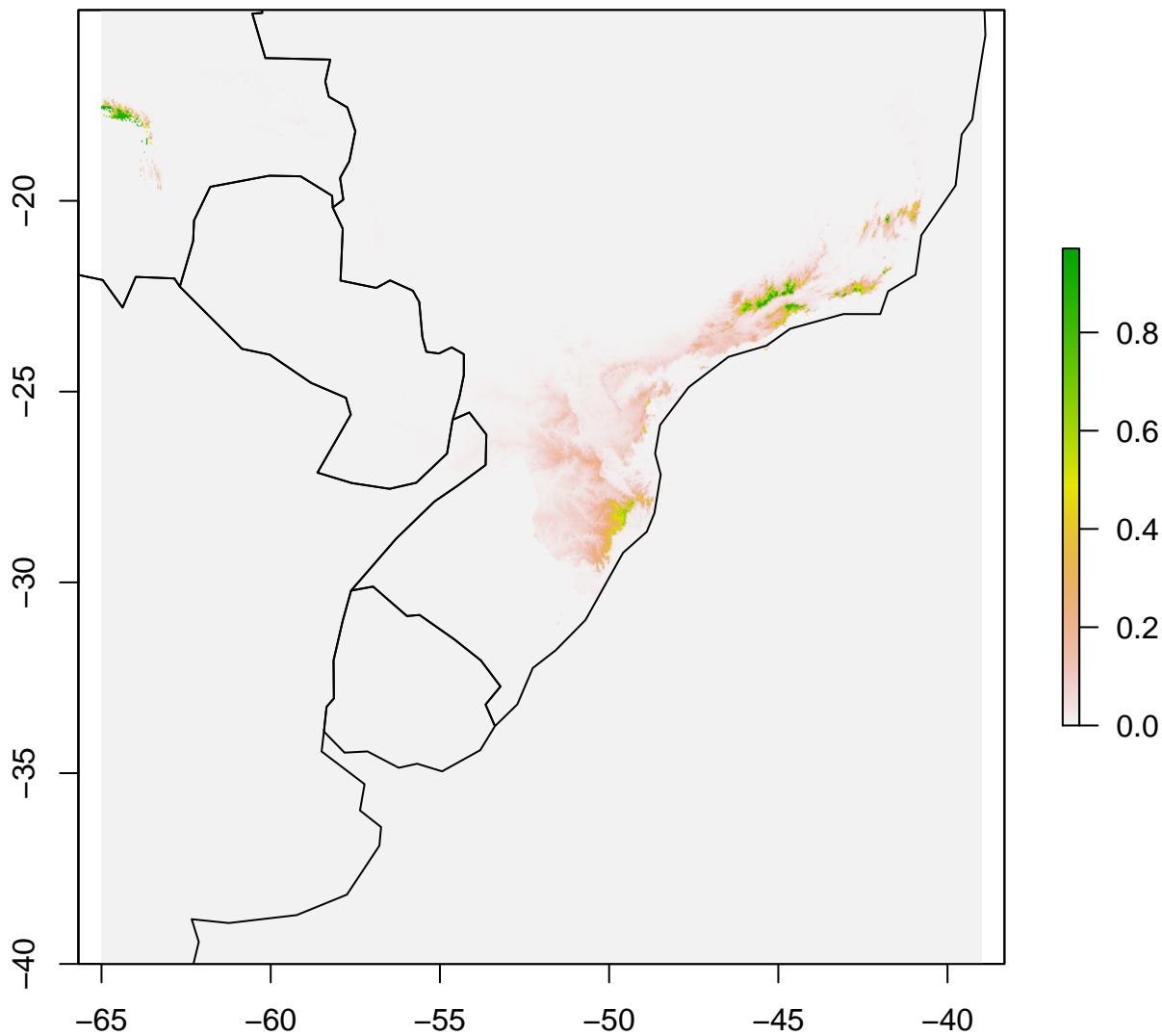

Supplement: Supplementary file 7 — Supplementary Data 4 [file 41467_2021_26537_MOESM7_ESM.gz › Dataset S1/thoracica_projection_MESS]

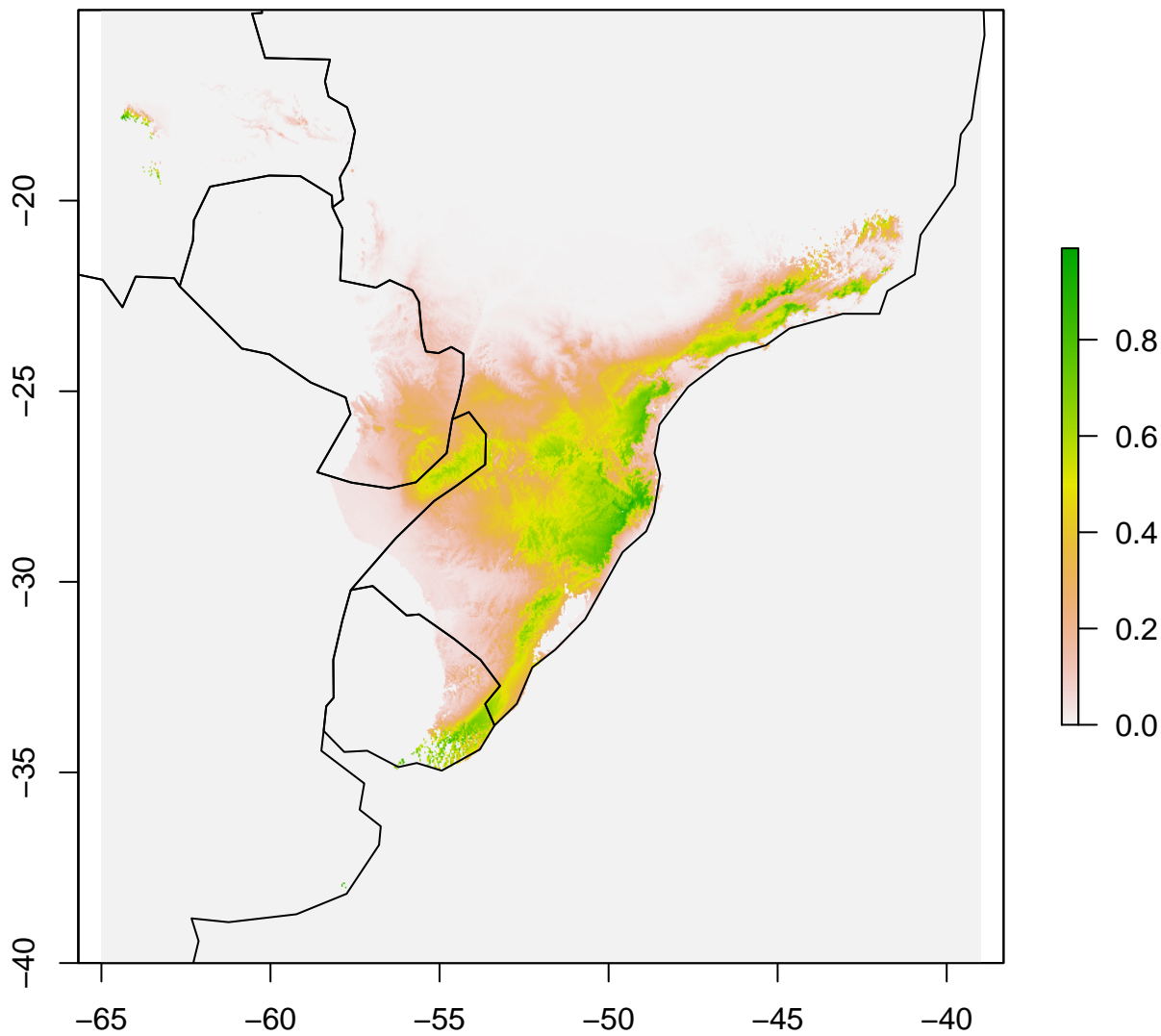

Supplement: Supplementary file 7 — Supplementary Data 4 [file 41467_2021_26537_MOESM7_ESM.gz › Dataset S1/falcinellus_projection_MESS]

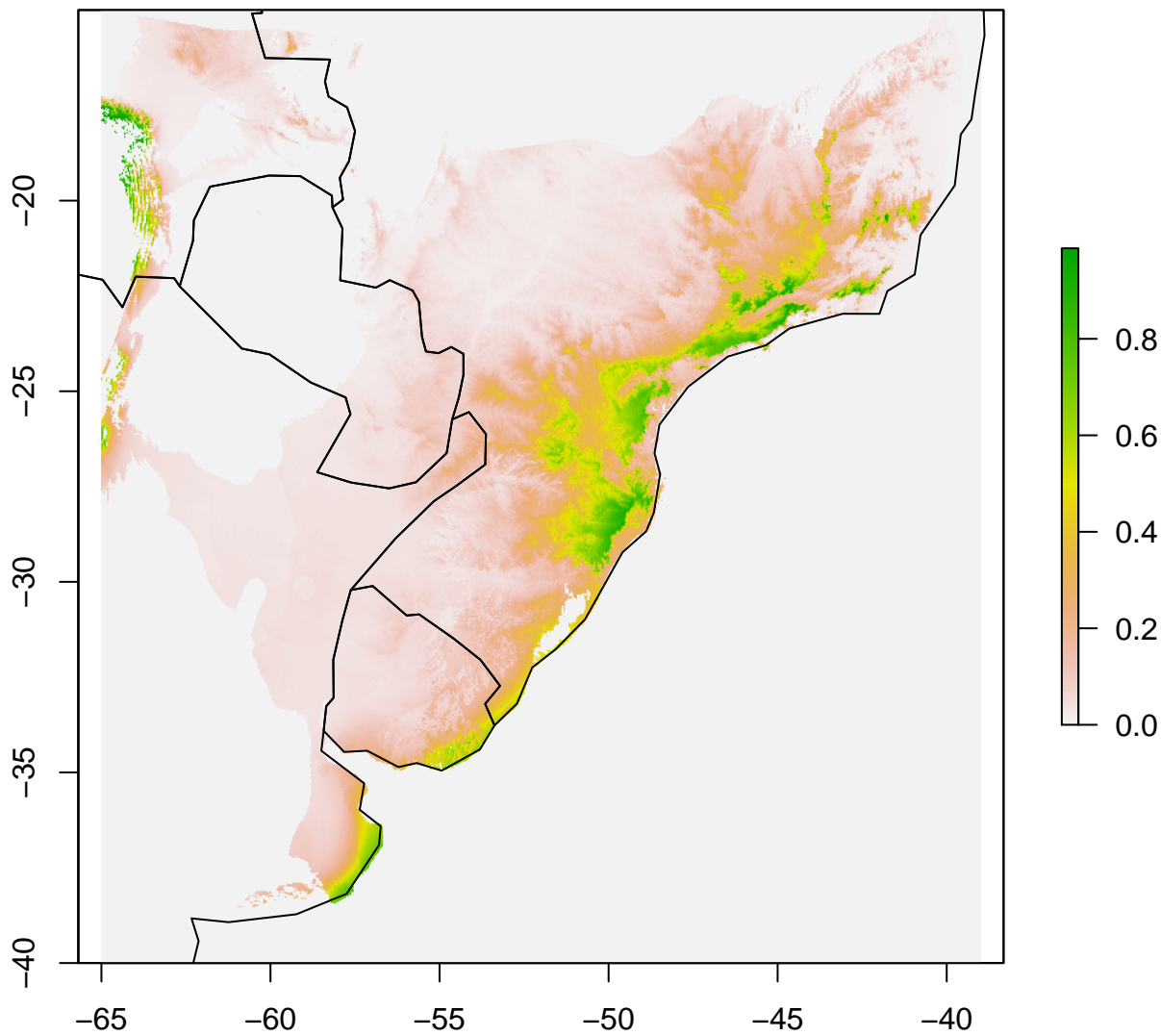

Supplement: Supplementary file 7 — Supplementary Data 4 [file 41467_2021_26537_MOESM7_ESM.gz › Dataset S1/mesoleuca_projection_MESS]

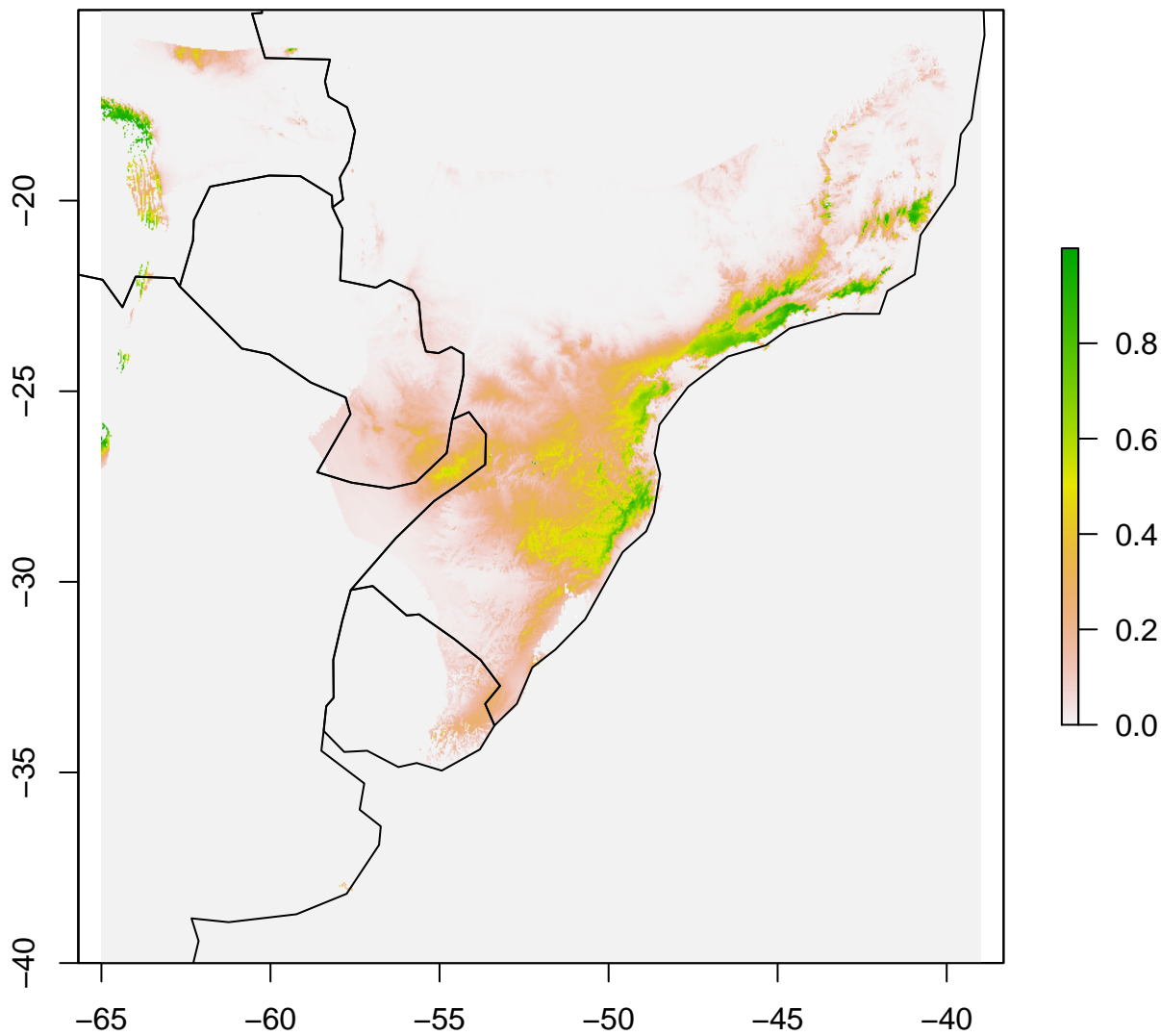

Supplement: Supplementary file 7 — Supplementary Data 4 [file 41467_2021_26537_MOESM7_ESM.gz › Dataset S1/aurulentus_projection_MESS]

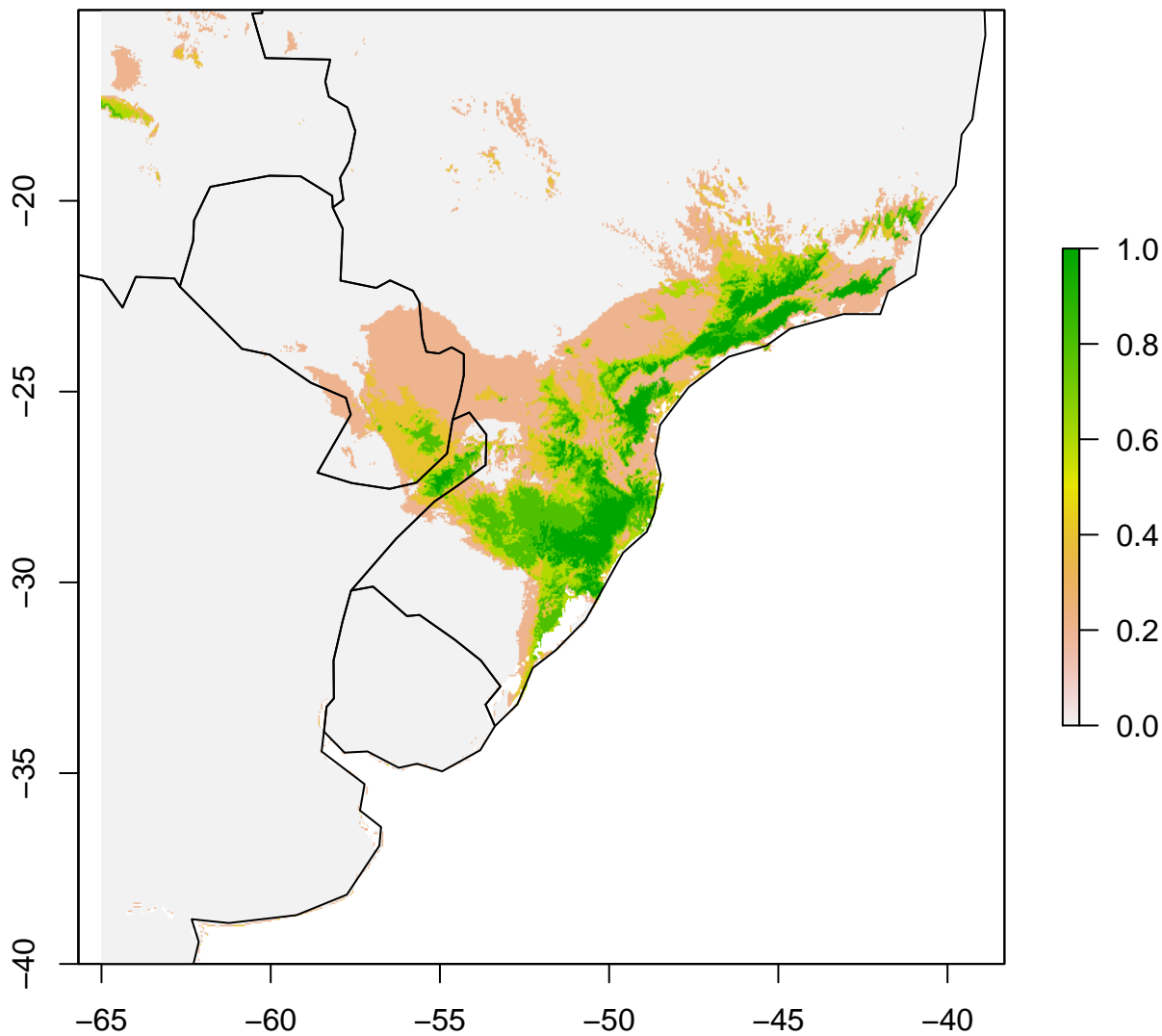

Supplement: Supplementary file 7 — Supplementary Data 4 [file 41467_2021_26537_MOESM7_ESM.gz › Dataset S1/ruficauda_stability_map.pdf]

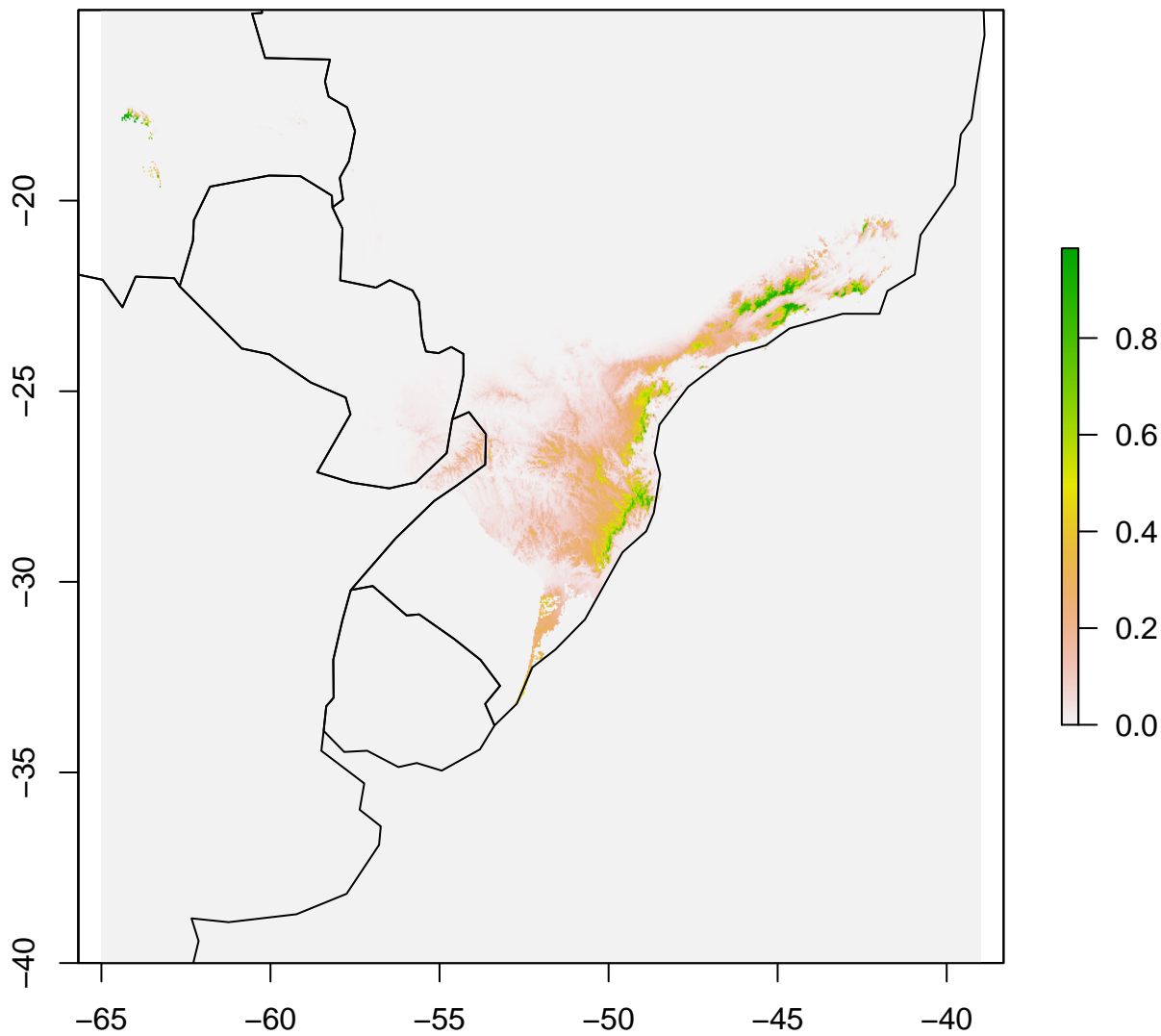

Supplement: Supplementary file 7 — Supplementary Data 4 [file 41467_2021_26537_MOESM7_ESM.gz › Dataset S1/obsoletus_projection_MESS]

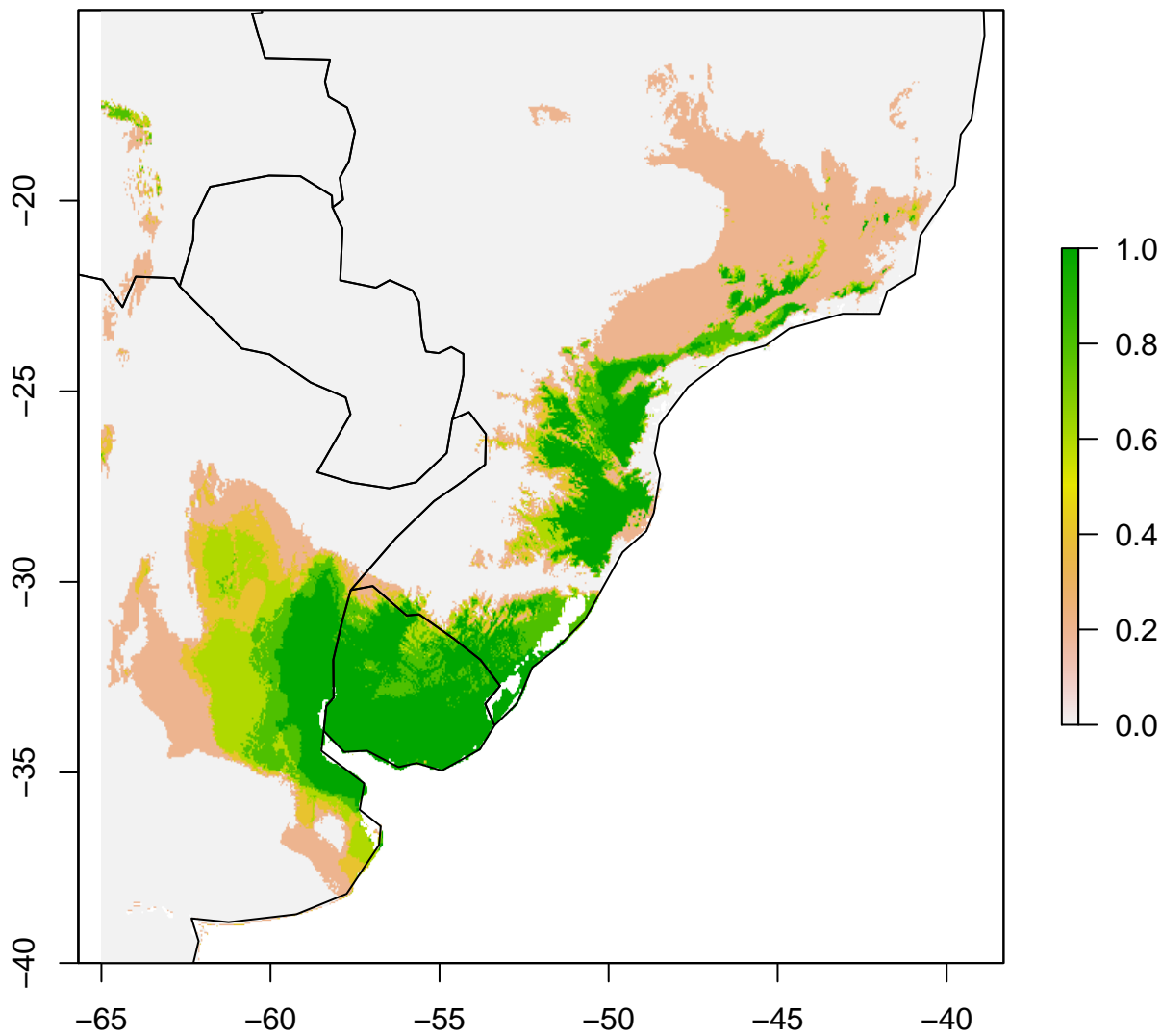

Supplement: Supplementary file 7 — Supplementary Data 4 [file 41467_2021_26537_MOESM7_ESM.gz › Dataset S1/diadematus_stability_map.pdf]

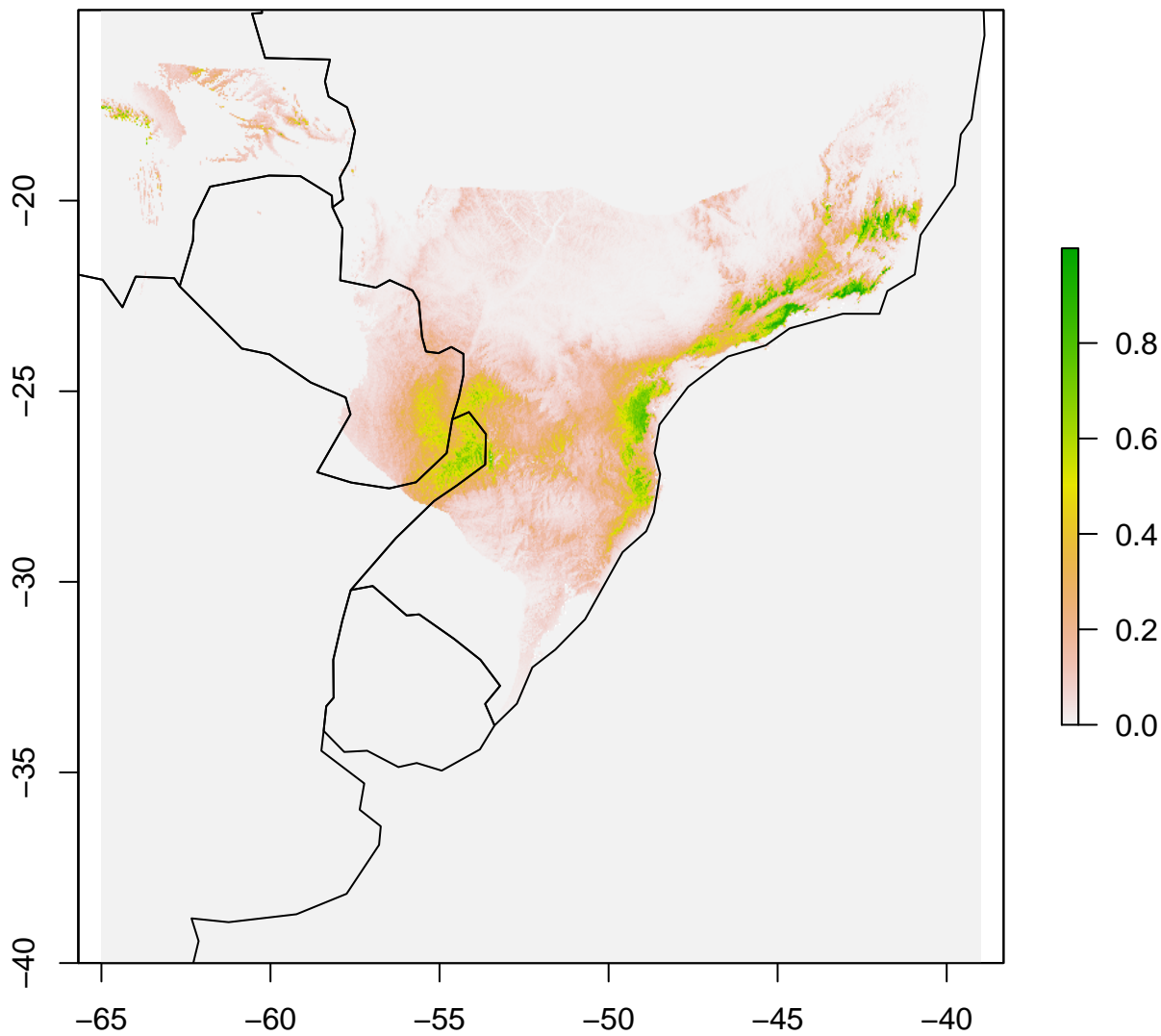

Supplement: Supplementary file 7 — Supplementary Data 4 [file 41467_2021_26537_MOESM7_ESM.gz › Dataset S1/rubricollis_projection_MESS]

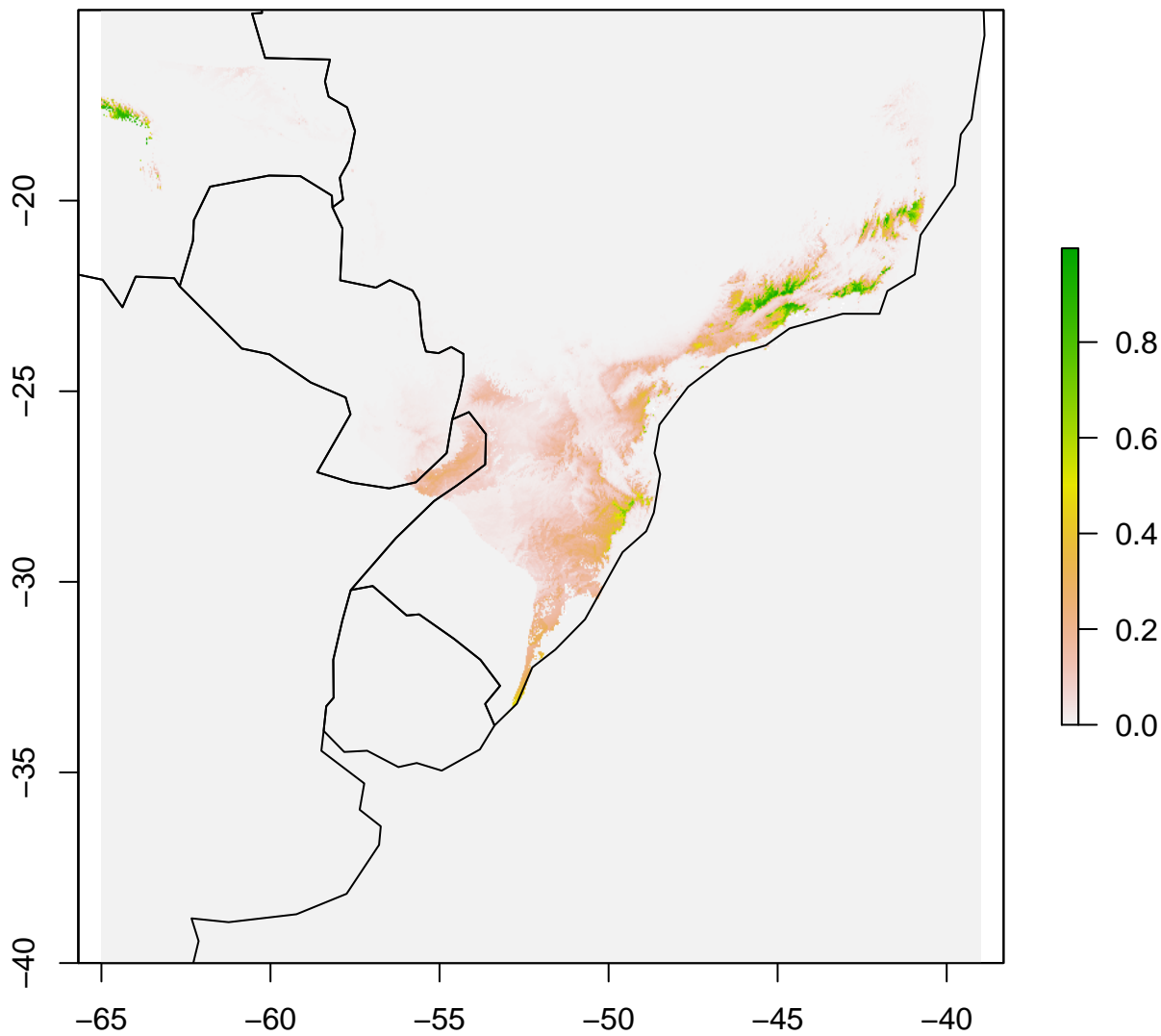

Supplement: Supplementary file 7 — Supplementary Data 4 [file 41467_2021_26537_MOESM7_ESM.gz › Dataset S1/ruficauda_projection_MESS]

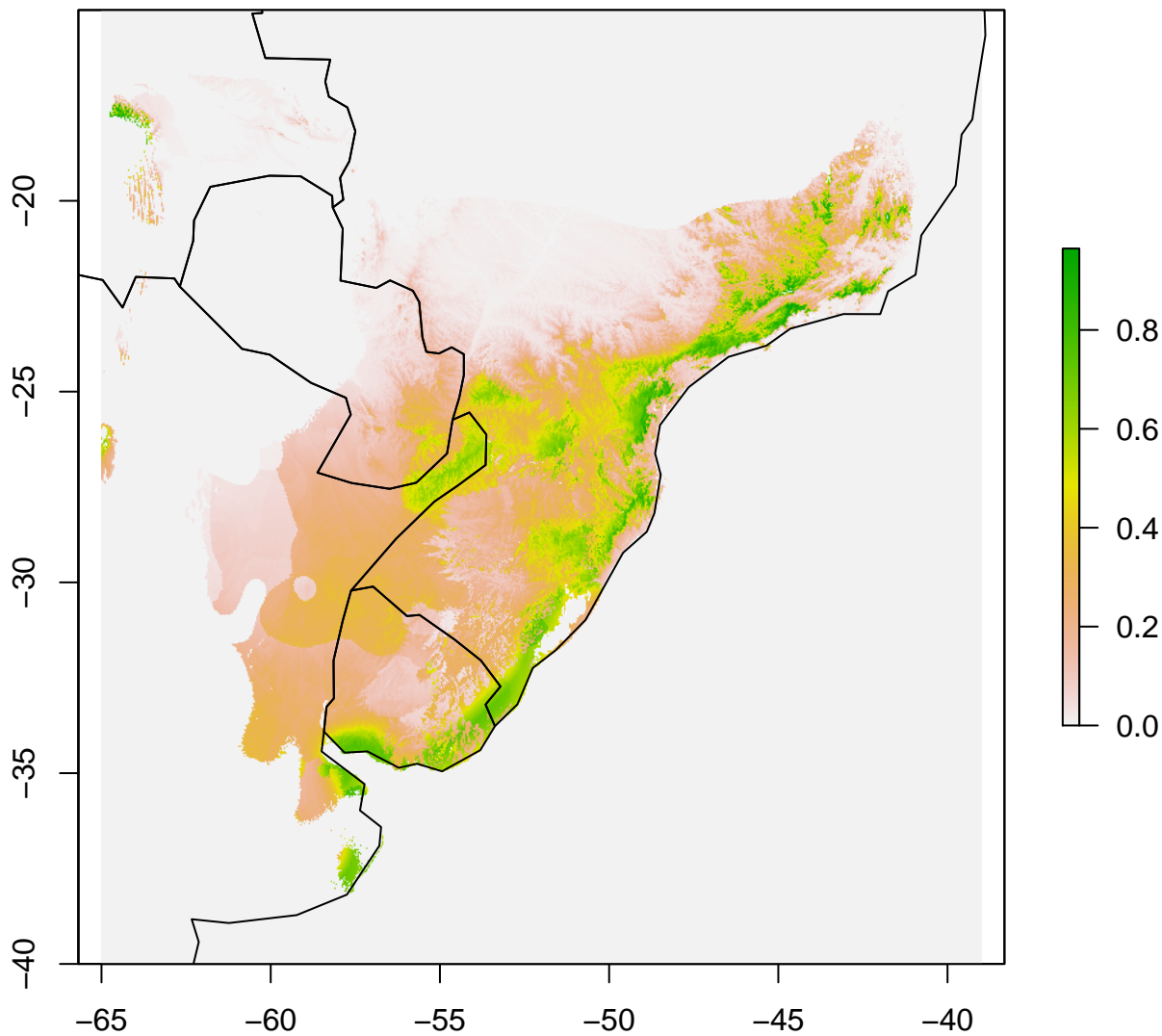

Supplement: Supplementary file 7 — Supplementary Data 4 [file 41467_2021_26537_MOESM7_ESM.gz › Dataset S1/rufosuperciliata_projection_MESS]

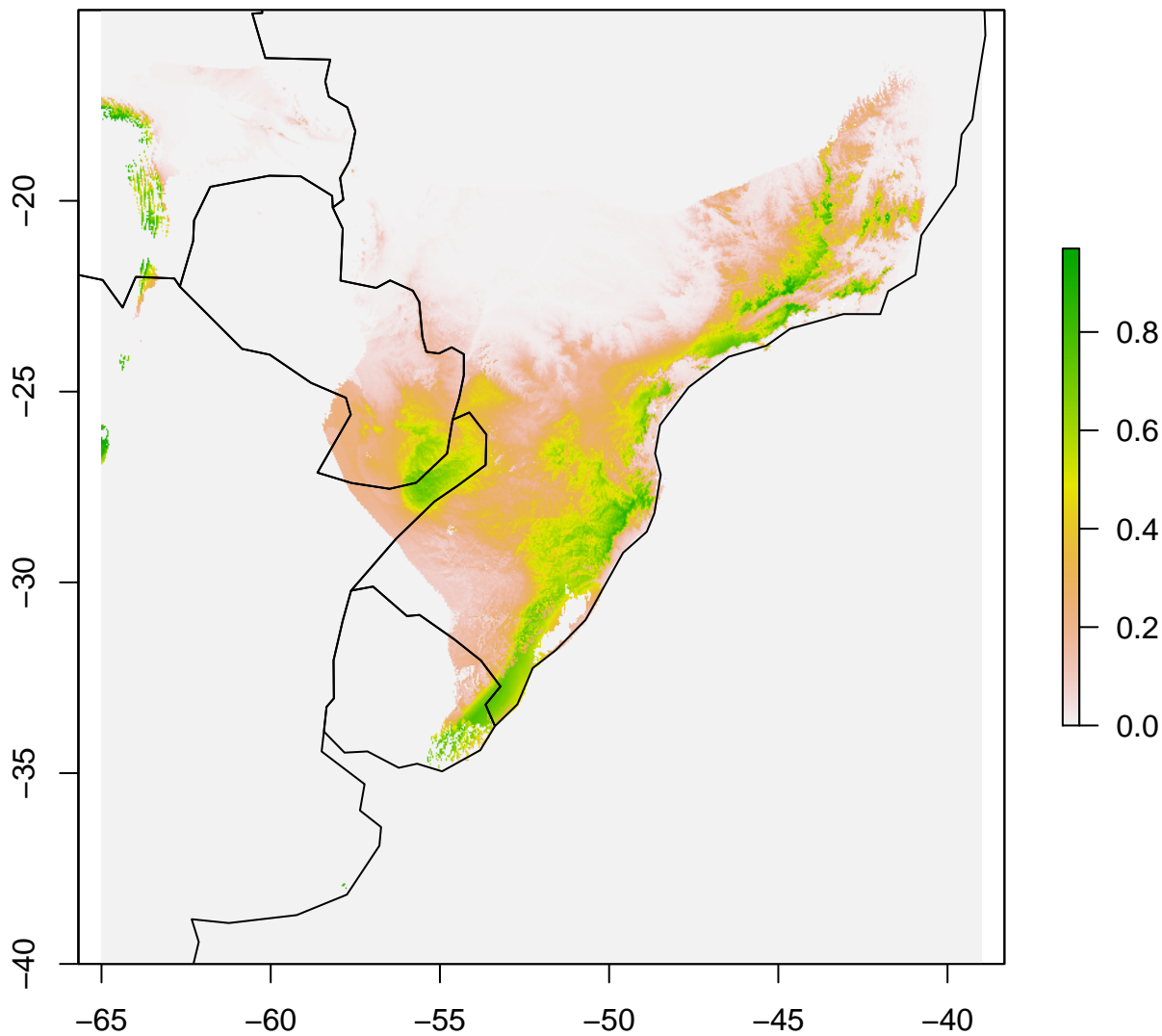

Supplement: Supplementary file 7 — Supplementary Data 4 [file 41467_2021_26537_MOESM7_ESM.gz › Dataset S1/leachii_projection_MESS]

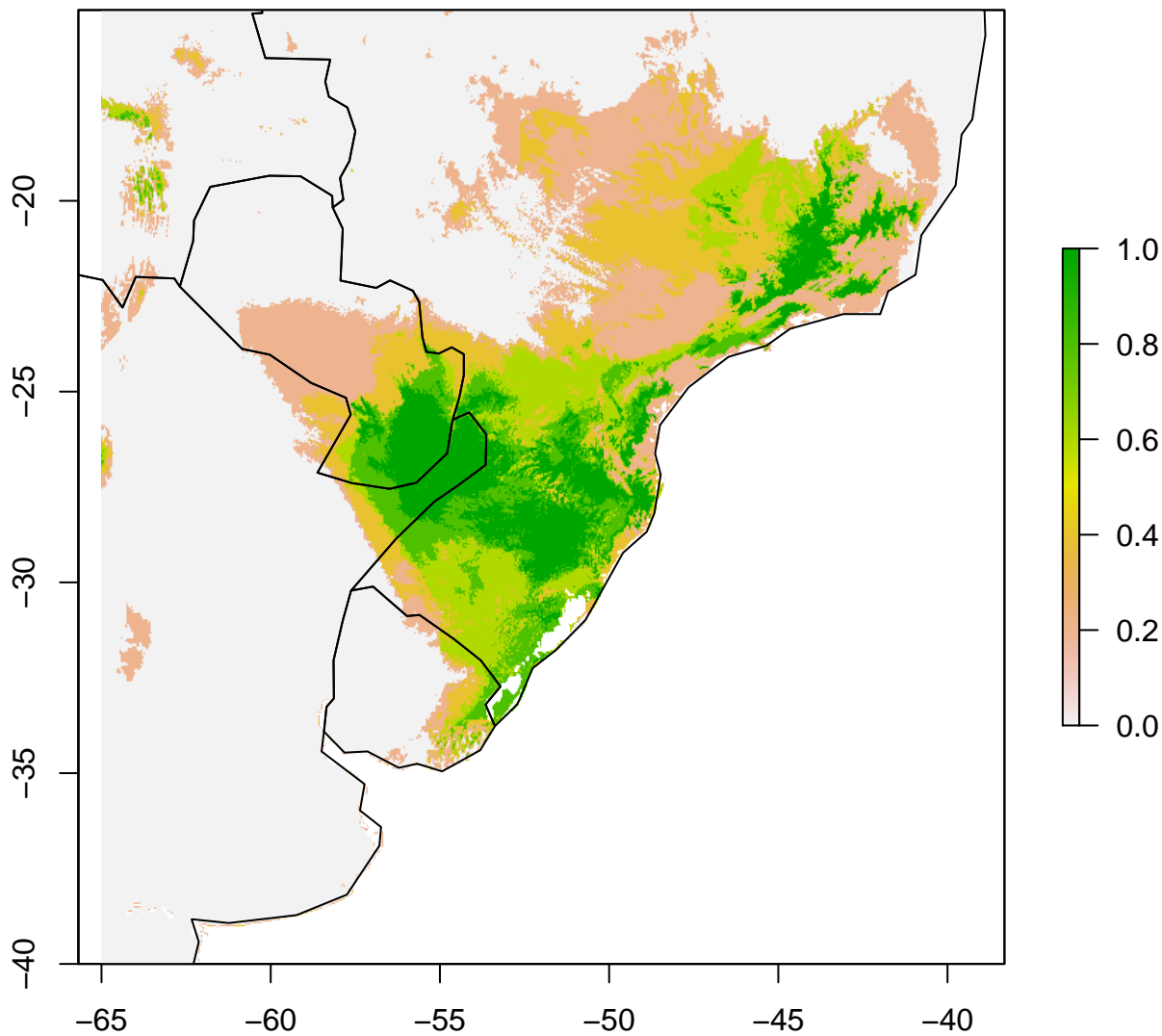

Supplement: Supplementary file 7 — Supplementary Data 4 [file 41467_2021_26537_MOESM7_ESM.gz › Dataset S1/leachii_stability_map.pdf]

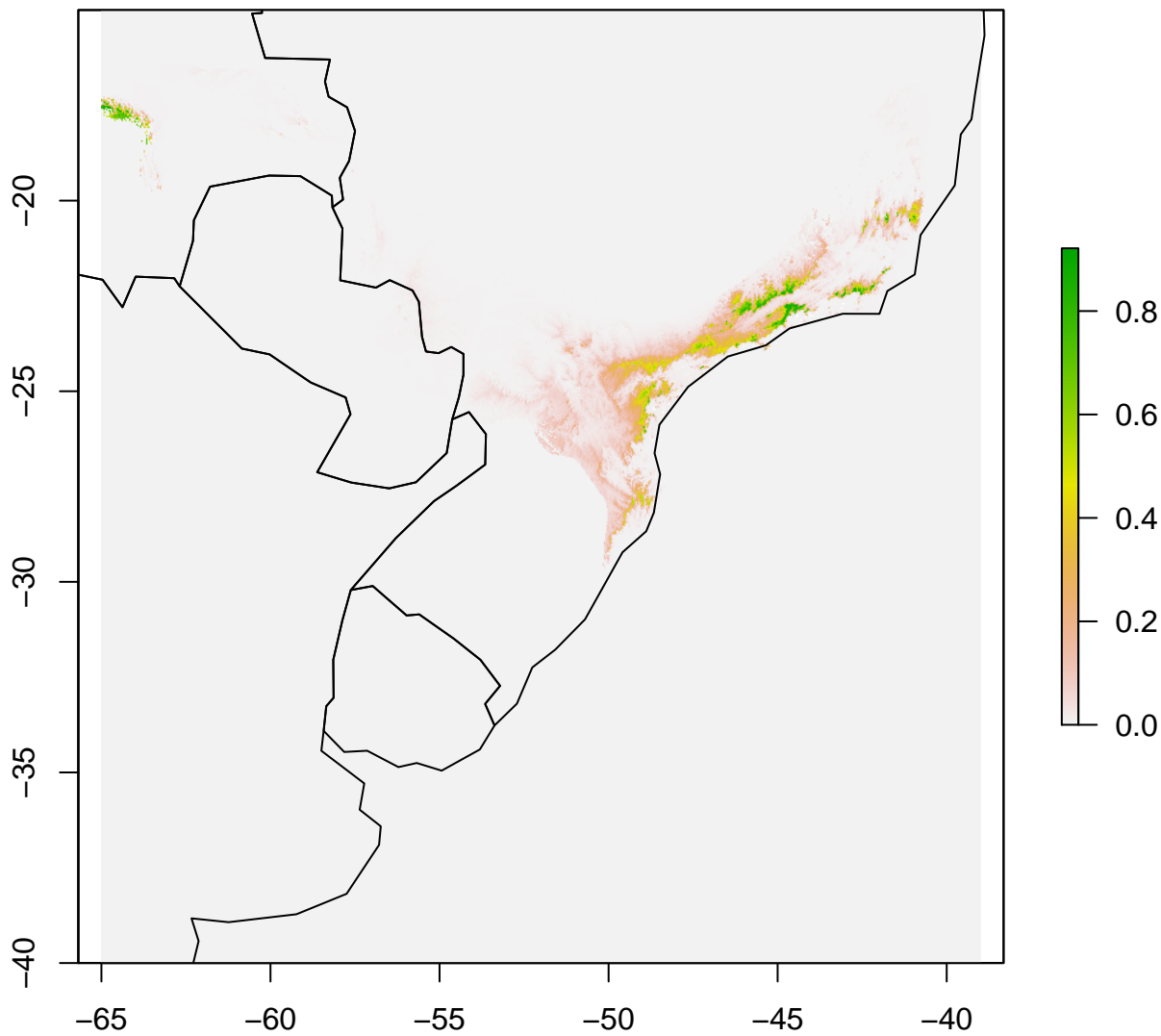

Supplement: Supplementary file 7 — Supplementary Data 4 [file 41467_2021_26537_MOESM7_ESM.gz › Dataset S1/xanthopterus_projection_MESS]

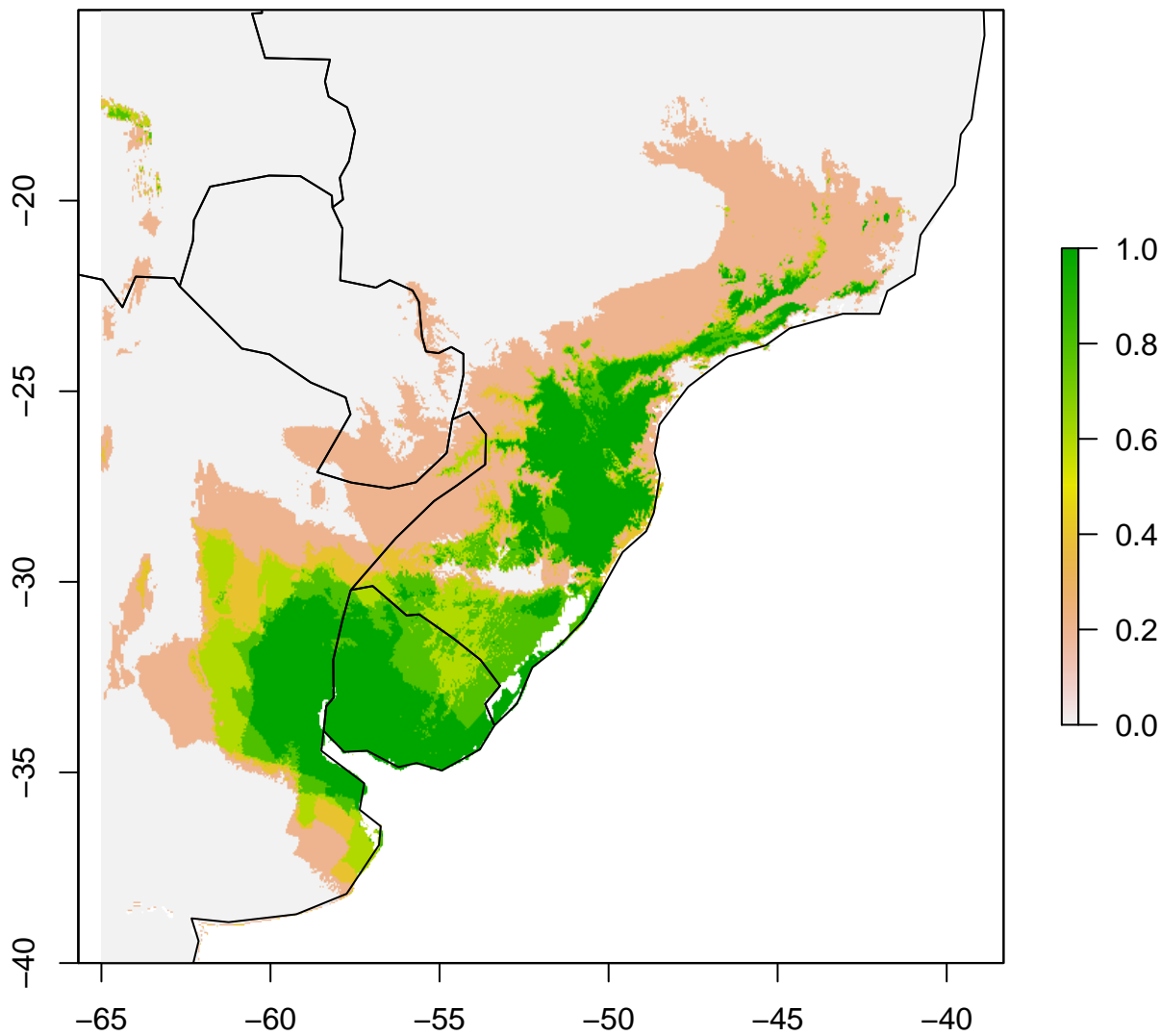

Supplement: Supplementary file 7 — Supplementary Data 4 [file 41467_2021_26537_MOESM7_ESM.gz › Dataset S1/lateralis_stability_map.pdf]

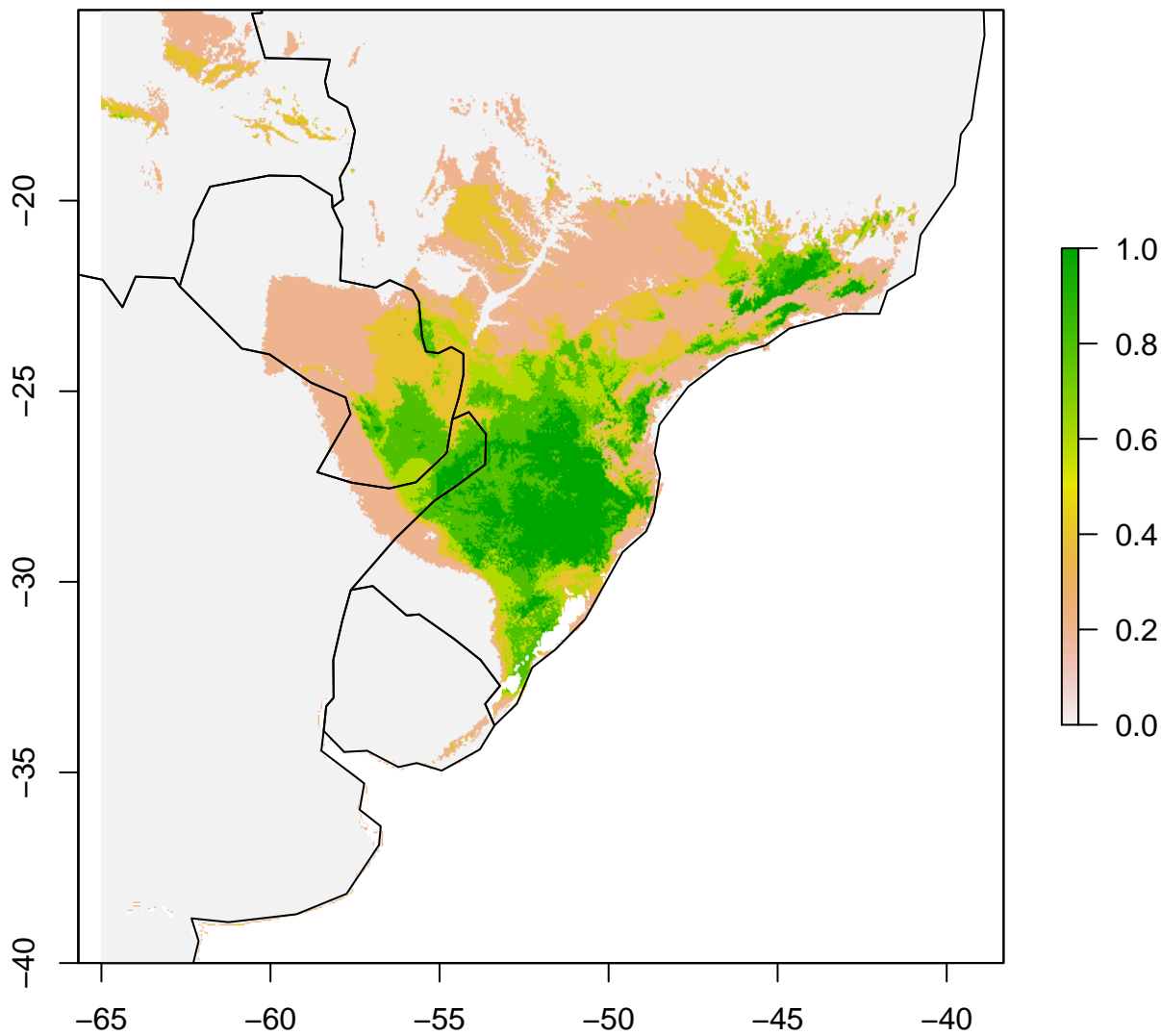

Supplement: Supplementary file 7 — Supplementary Data 4 [file 41467_2021_26537_MOESM7_ESM.gz › Dataset S1/maxillosus_stability_map.pdf]

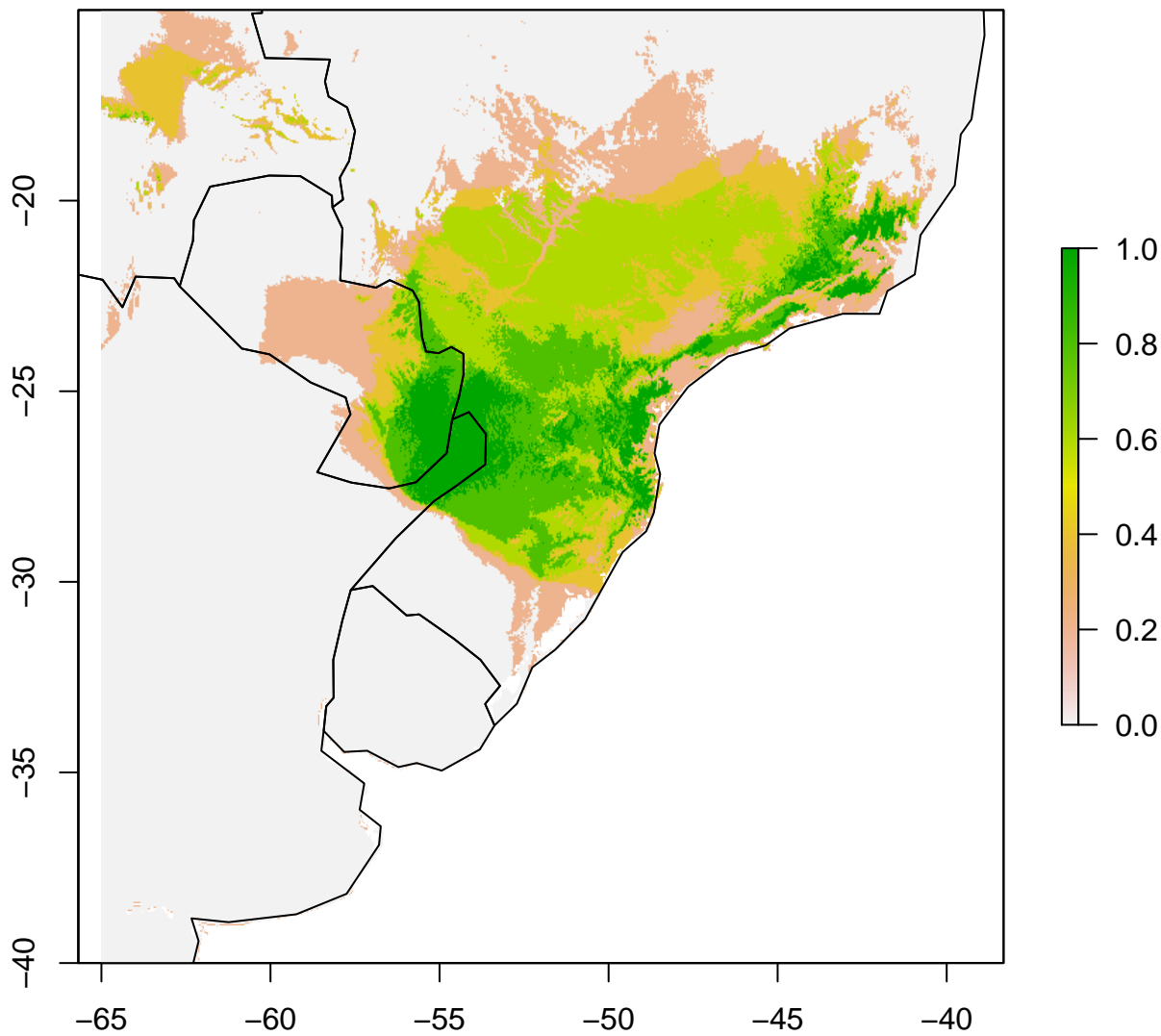

Supplement: Supplementary file 7 — Supplementary Data 4 [file 41467_2021_26537_MOESM7_ESM.gz › Dataset S1/rubricollis_stability_map.pdf]

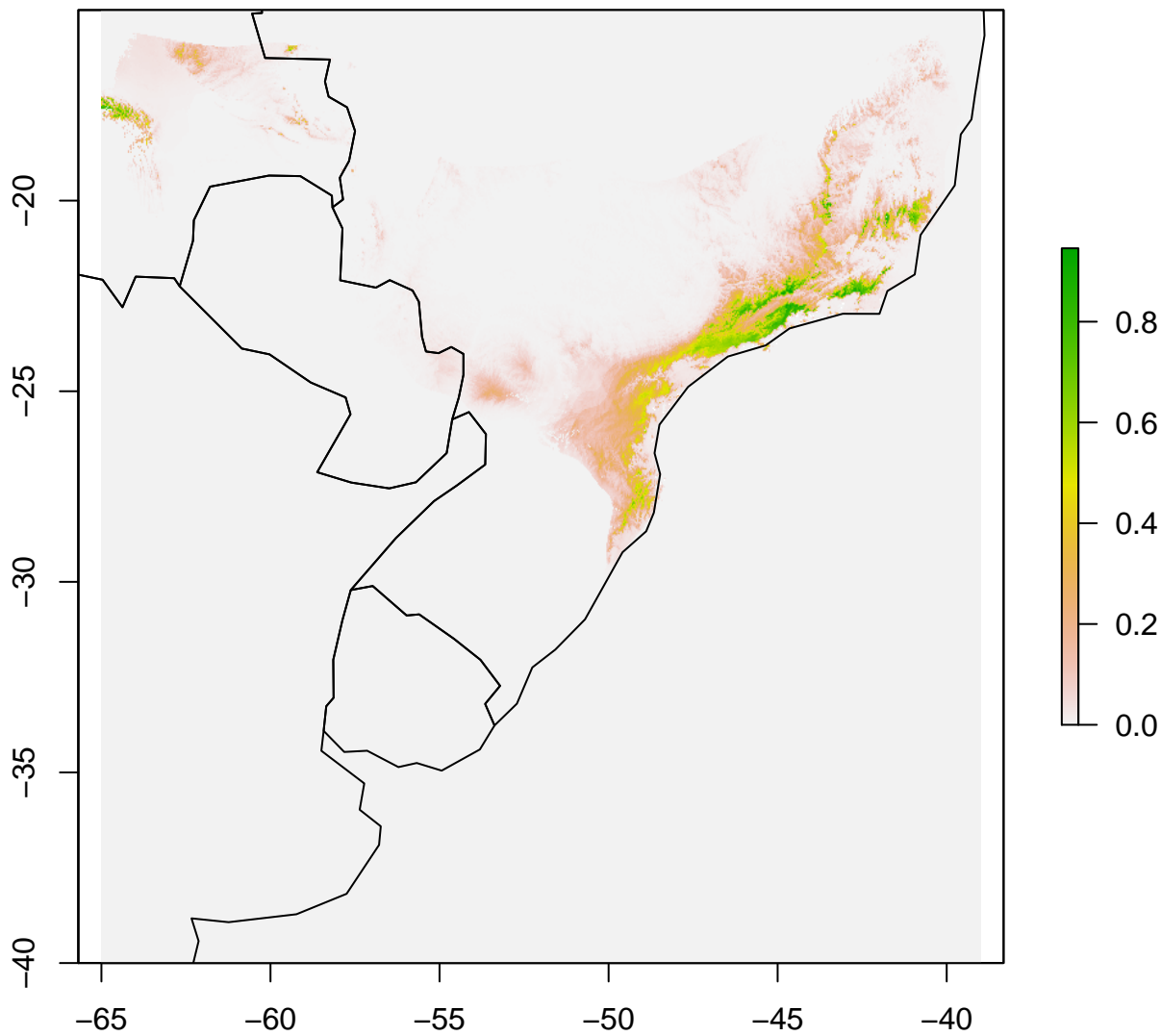

Supplement: Supplementary file 7 — Supplementary Data 4 [file 41467_2021_26537_MOESM7_ESM.gz › Dataset S1/desmaresti_projection_MESS]

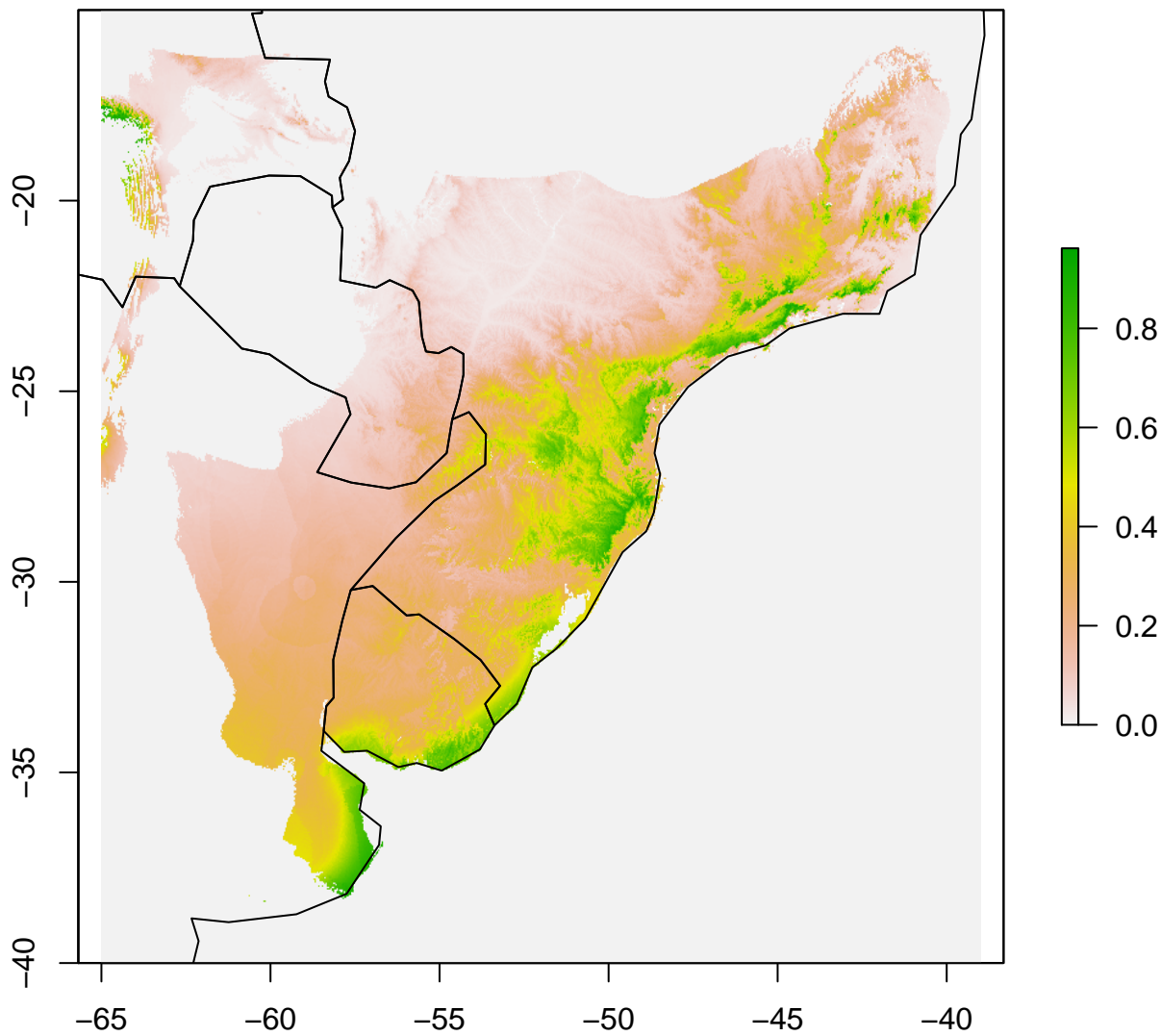

Supplement: Supplementary file 7 — Supplementary Data 4 [file 41467_2021_26537_MOESM7_ESM.gz › Dataset S1/ventralis_projection_MESS]

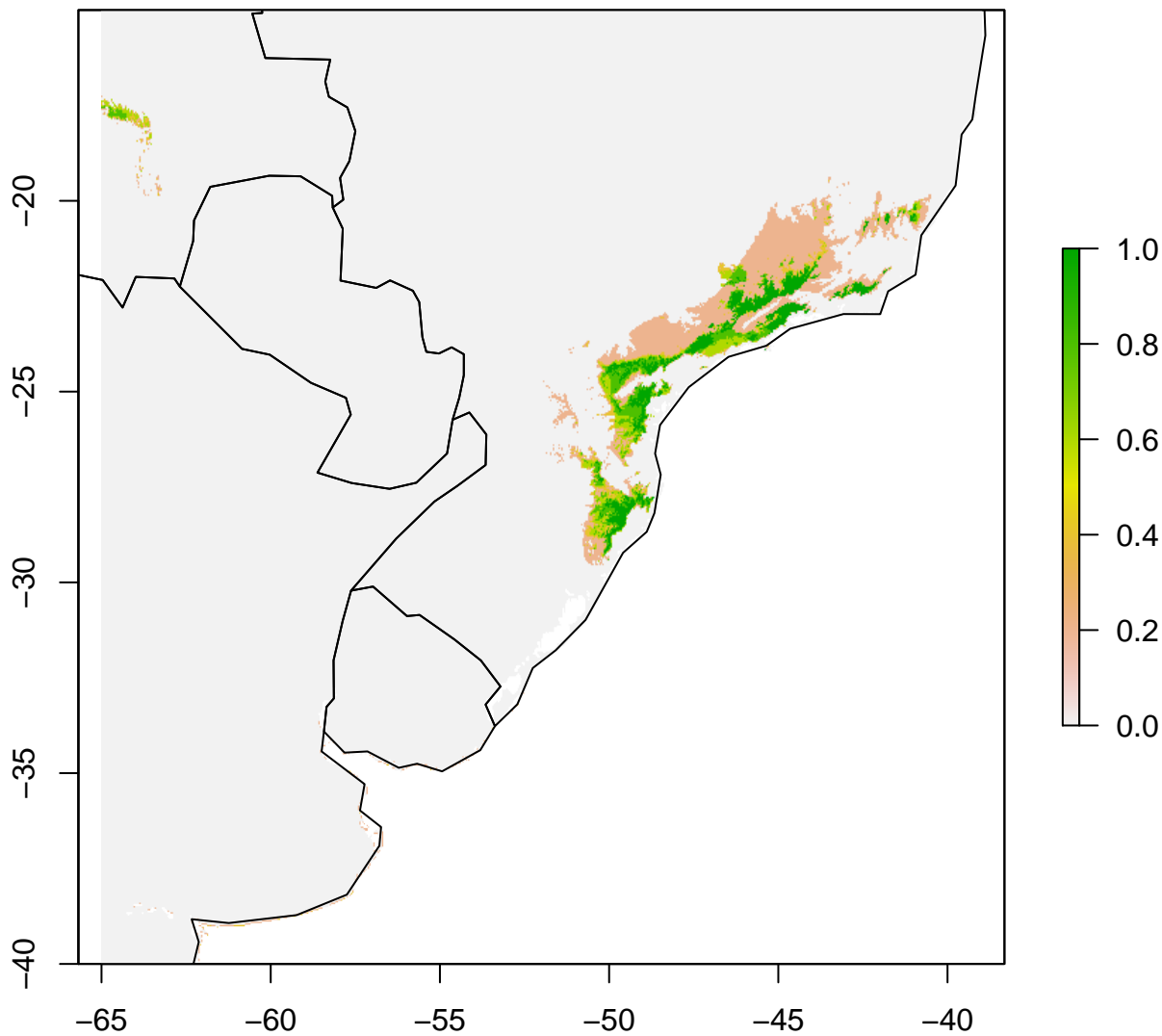

Supplement: Supplementary file 7 — Supplementary Data 4 [file 41467_2021_26537_MOESM7_ESM.gz › Dataset S1/difficilis_stability_map.pdf]

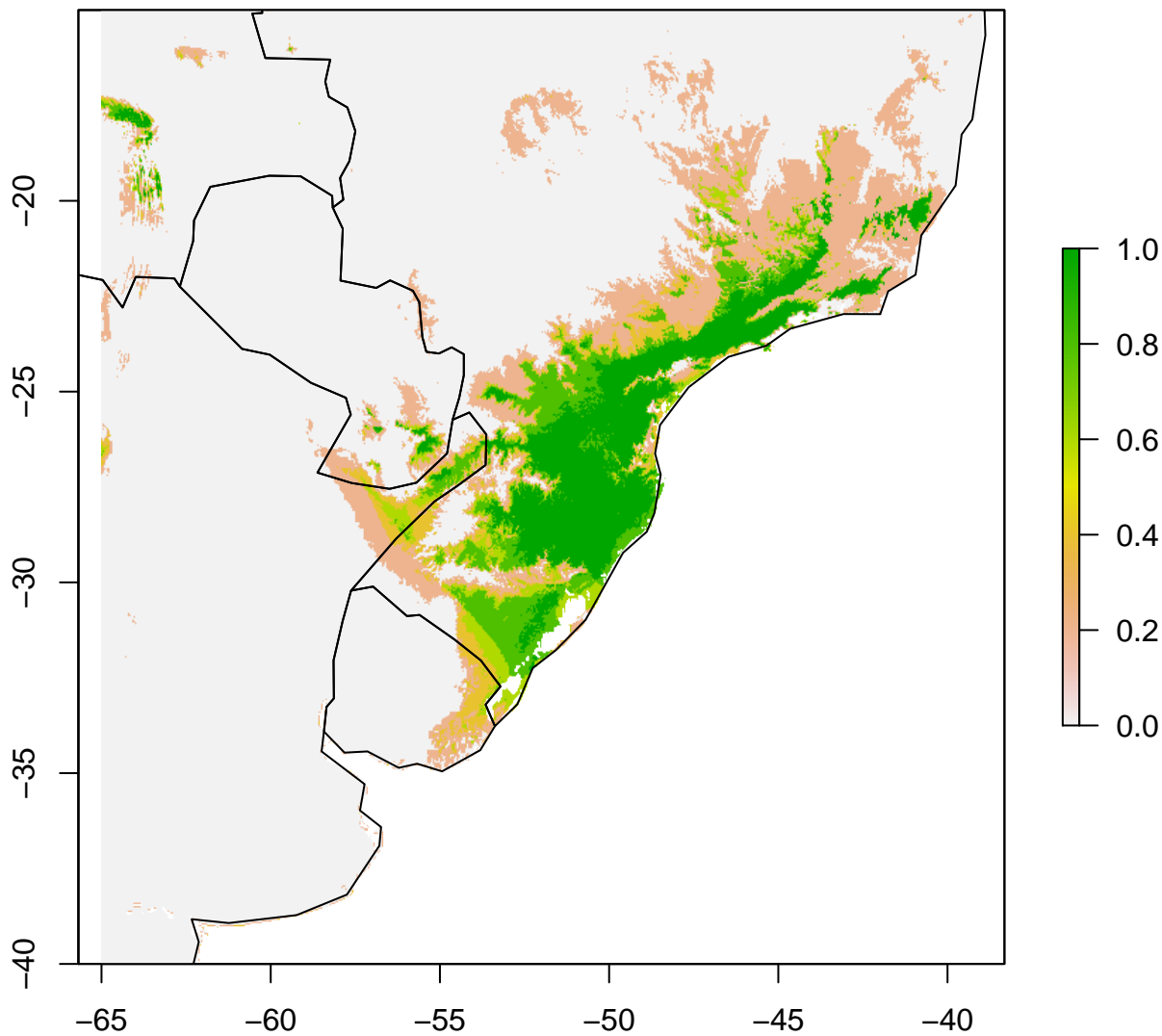

Supplement: Supplementary file 7 — Supplementary Data 4 [file 41467_2021_26537_MOESM7_ESM.gz › Dataset S1/aurulentus_stability_map.pdf]

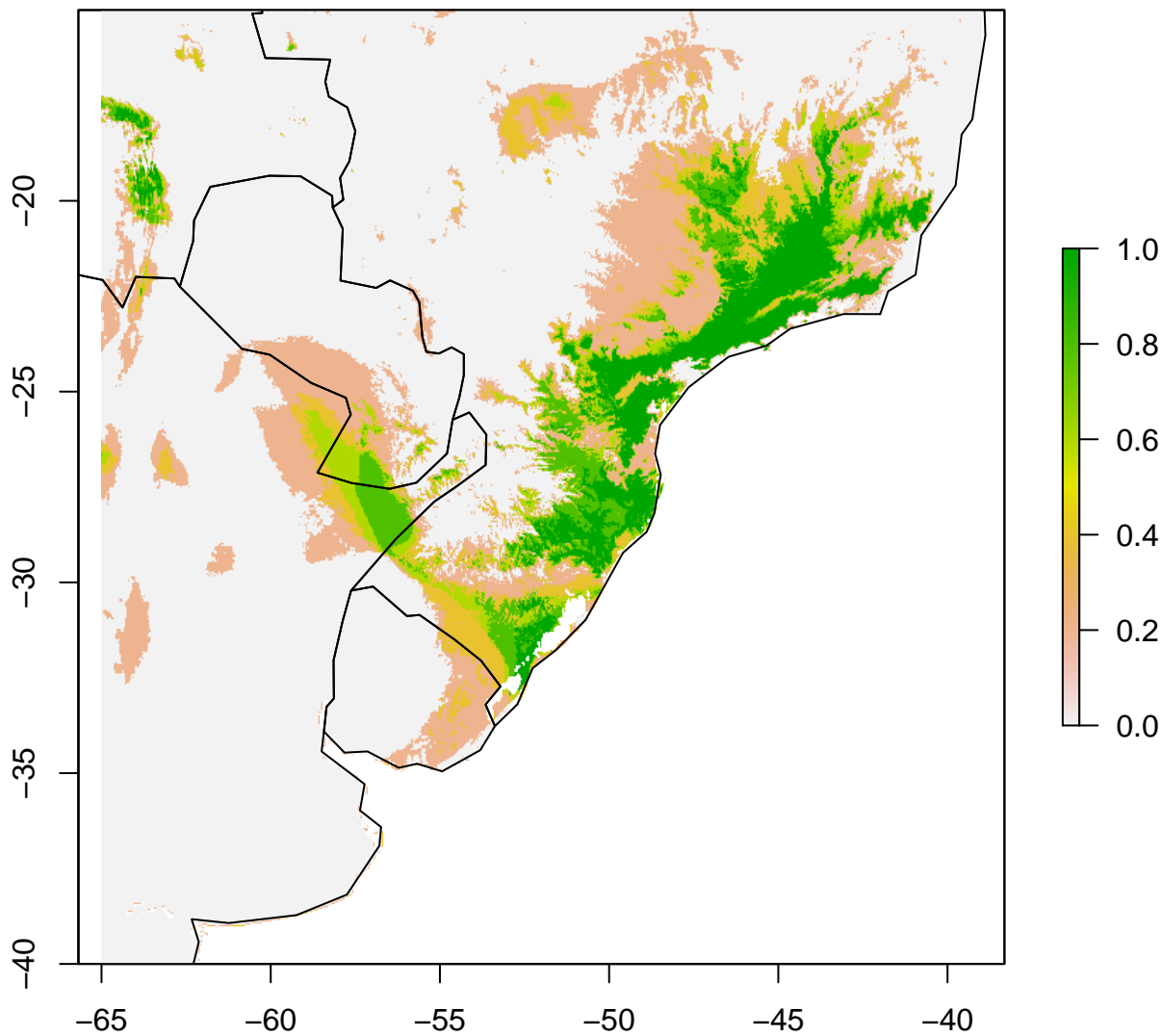

Supplement: Supplementary file 7 — Supplementary Data 4 [file 41467_2021_26537_MOESM7_ESM.gz › Dataset S1/vetula_stability_map.pdf]

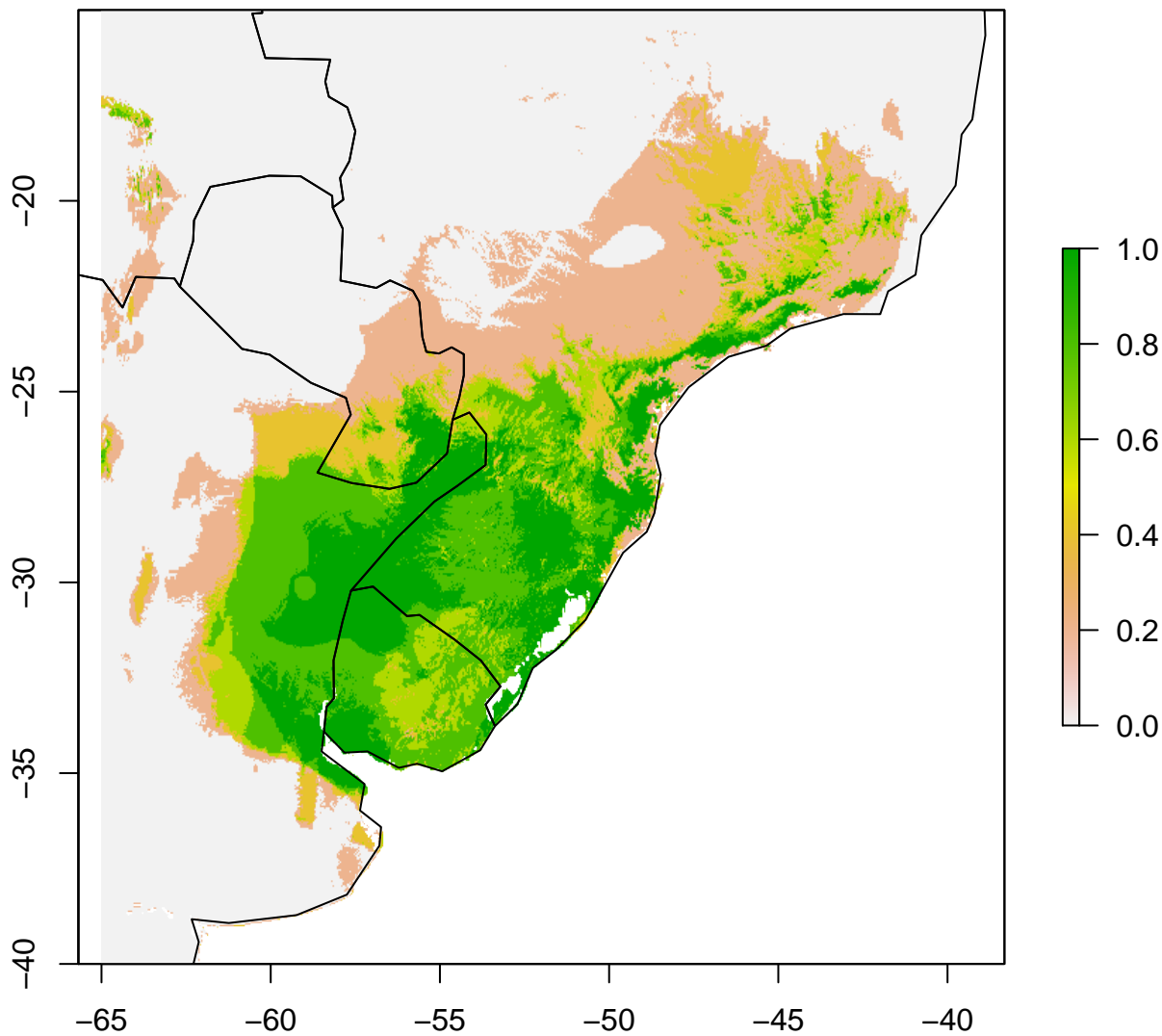

Supplement: Supplementary file 7 — Supplementary Data 4 [file 41467_2021_26537_MOESM7_ESM.gz › Dataset S1/rufosuperciliata_stability_map.pdf]

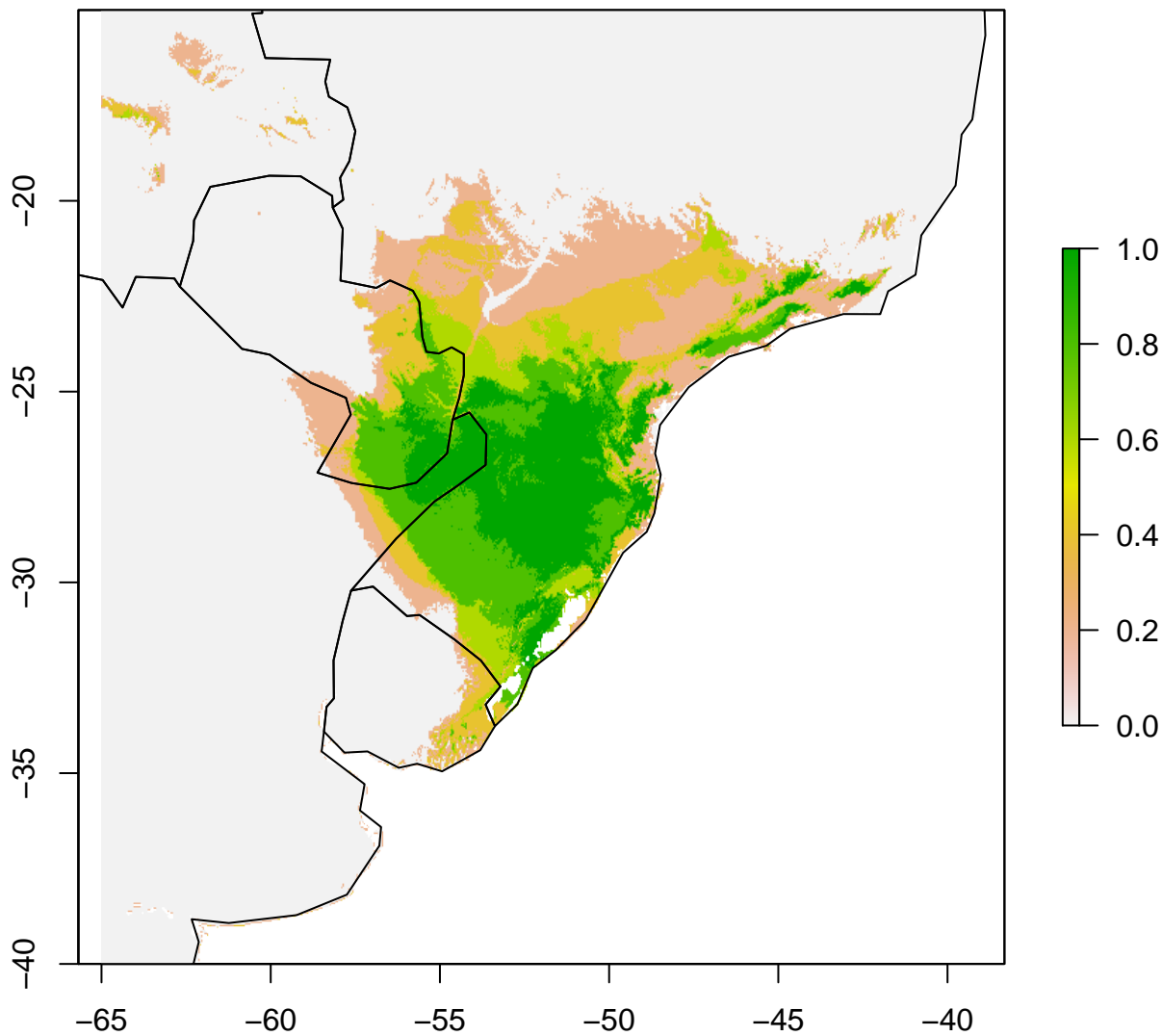

Supplement: Supplementary file 7 — Supplementary Data 4 [file 41467_2021_26537_MOESM7_ESM.gz › Dataset S1/falcinellus_stability_map.pdf]

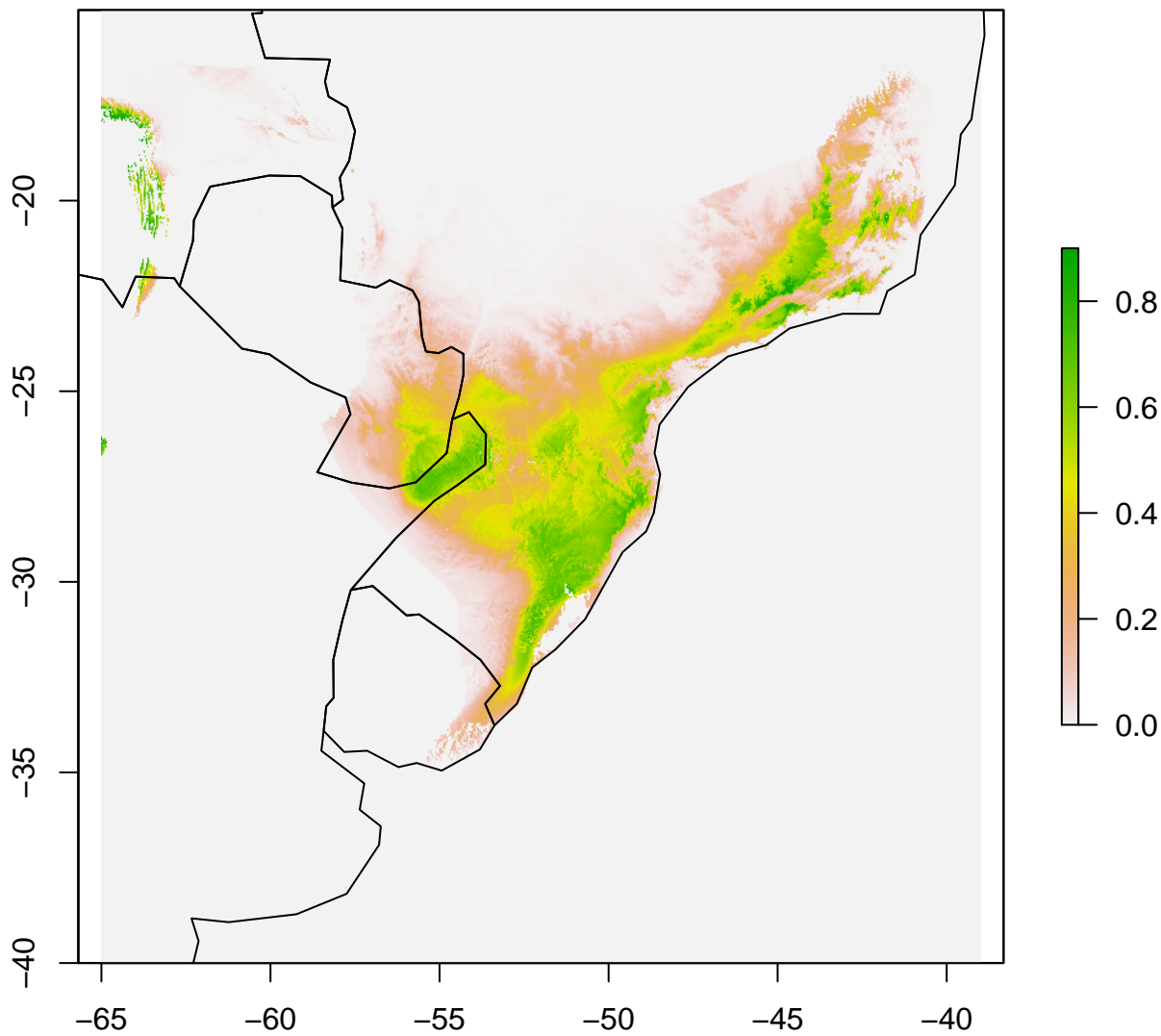

Supplement: Supplementary file 7 — Supplementary Data 4 [file 41467_2021_26537_MOESM7_ESM.gz › Dataset S1/cinerascens_projection_MESS]

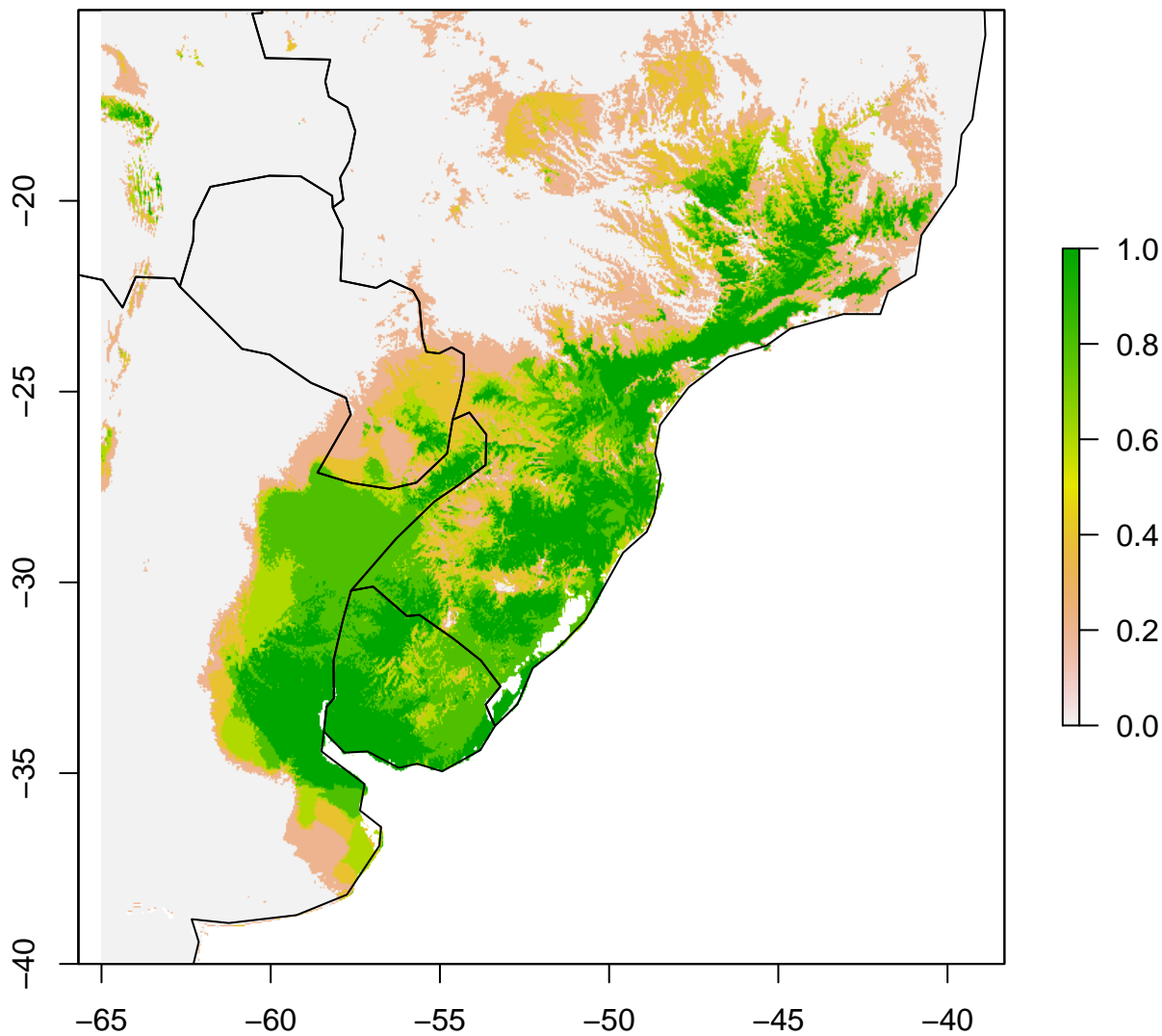

Supplement: Supplementary file 7 — Supplementary Data 4 [file 41467_2021_26537_MOESM7_ESM.gz › Dataset S1/ventralis_stability_map.pdf]

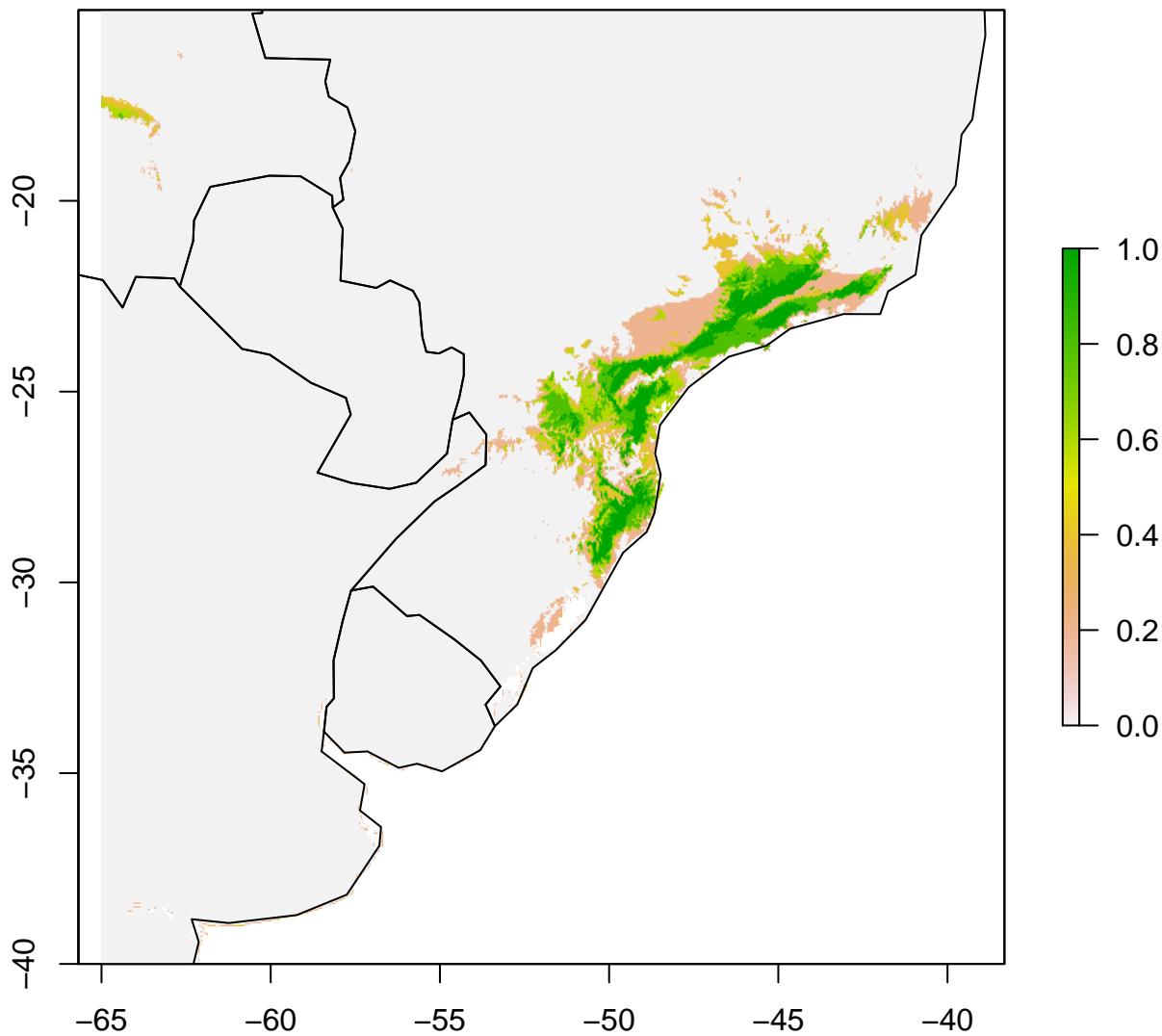

Supplement: Supplementary file 7 — Supplementary Data 4 [file 41467_2021_26537_MOESM7_ESM.gz › Dataset S1/pileata_stability_map.pdf]

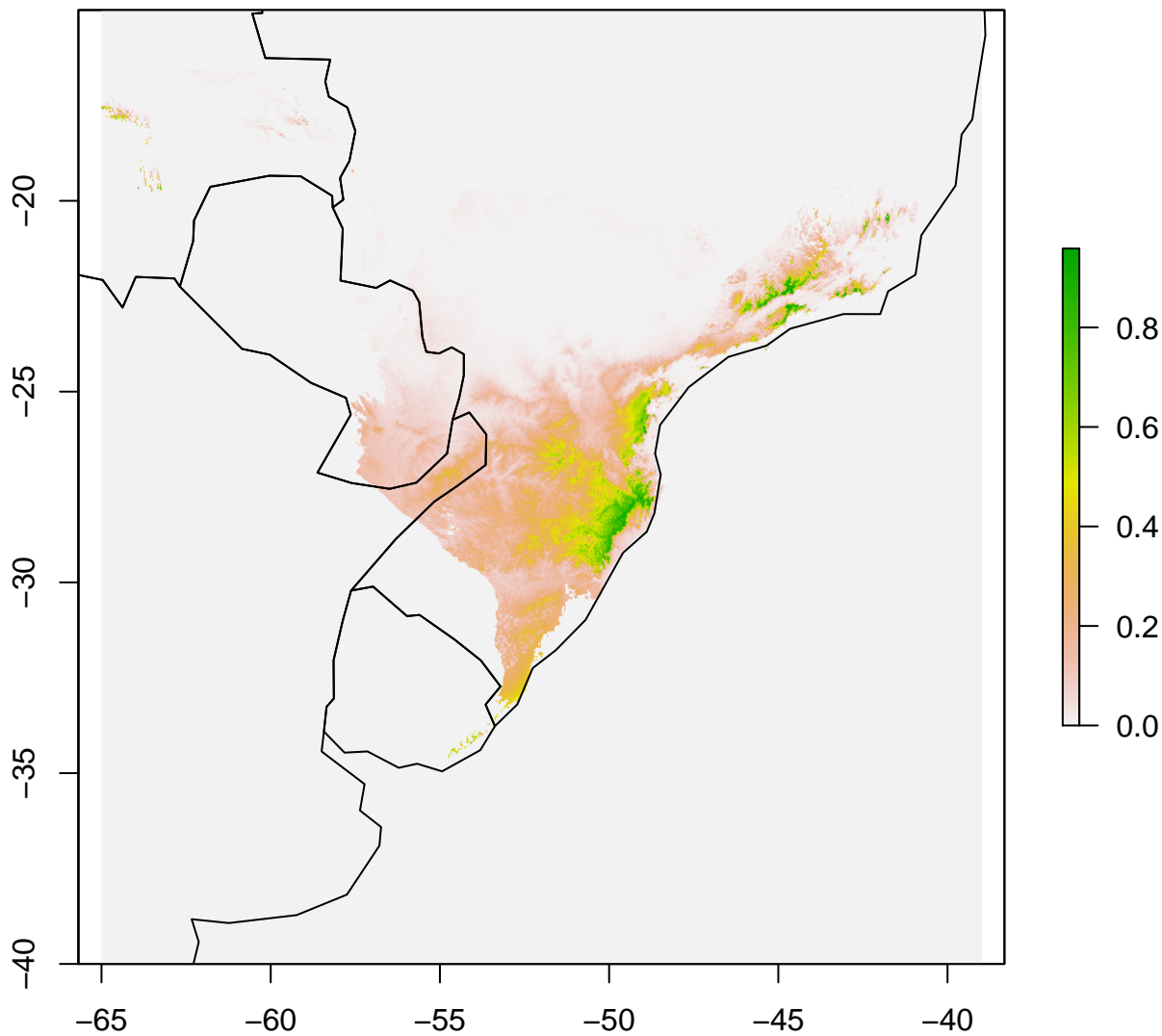

Supplement: Supplementary file 7 — Supplementary Data 4 [file 41467_2021_26537_MOESM7_ESM.gz › Dataset S1/maxillosus_projection_MESS]

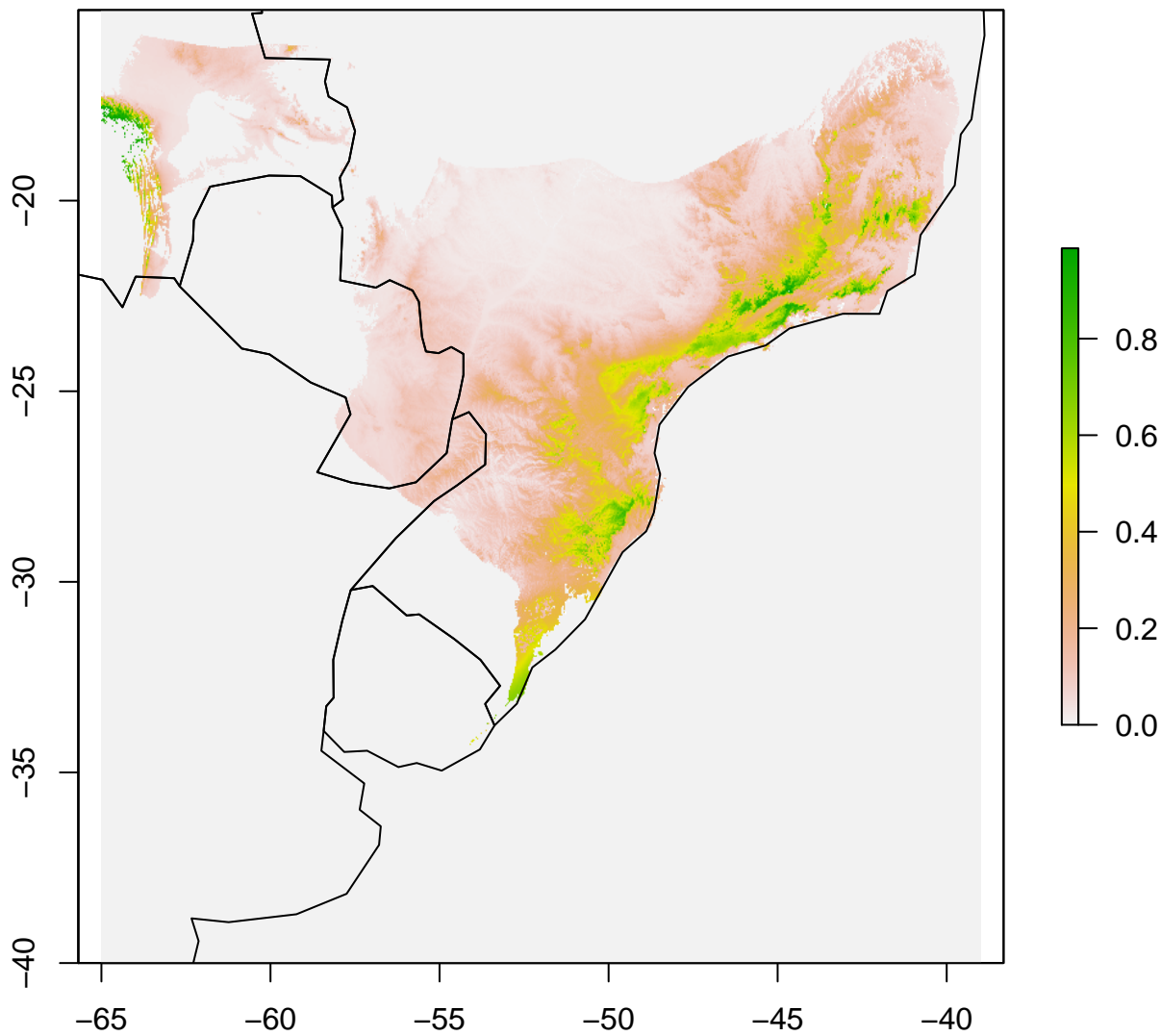

Supplement: Supplementary file 7 — Supplementary Data 4 [file 41467_2021_26537_MOESM7_ESM.gz › Dataset S1/vetula_projection_MESS]

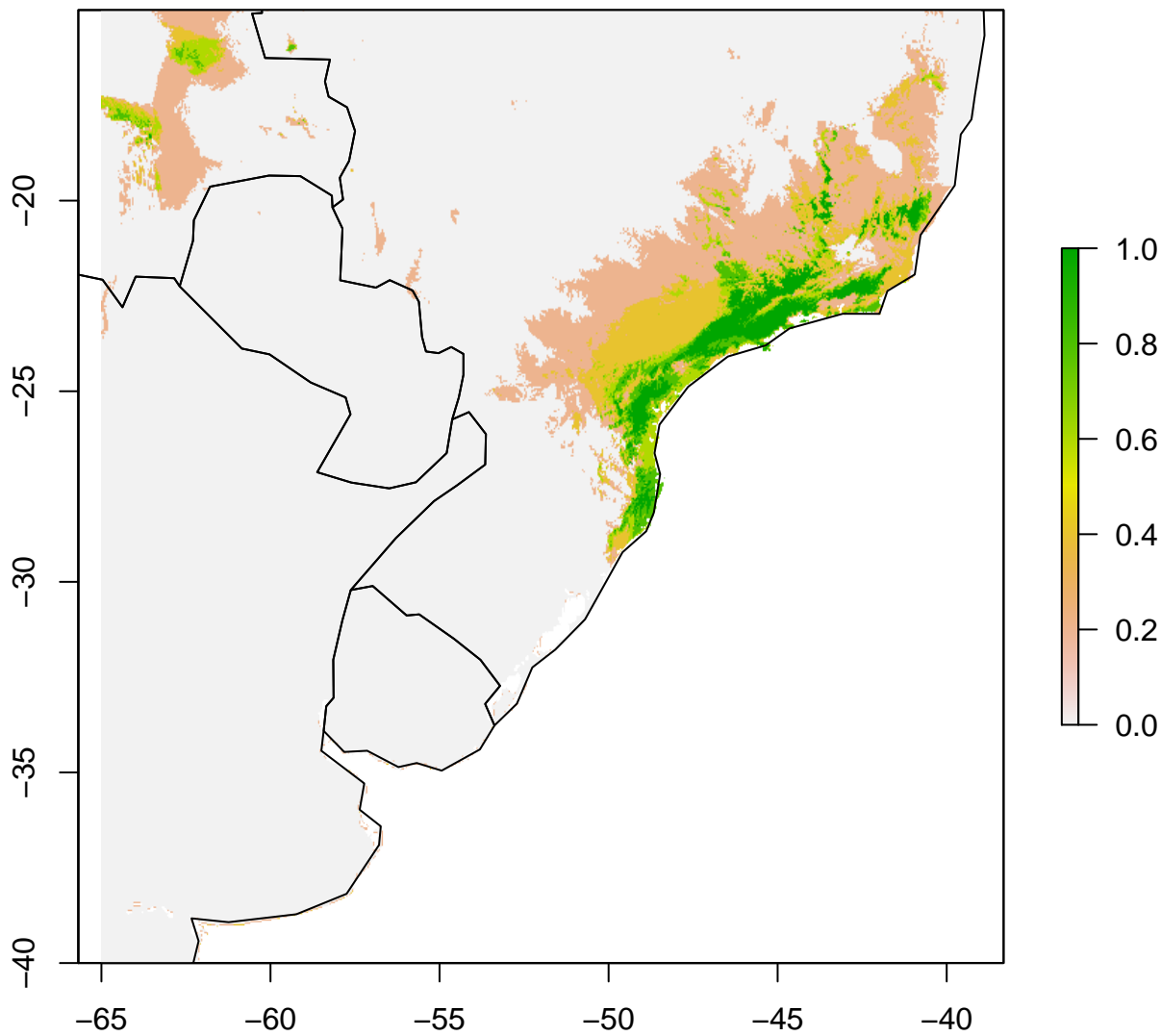

Supplement: Supplementary file 7 — Supplementary Data 4 [file 41467_2021_26537_MOESM7_ESM.gz › Dataset S1/desmaresti_stability_map.pdf]

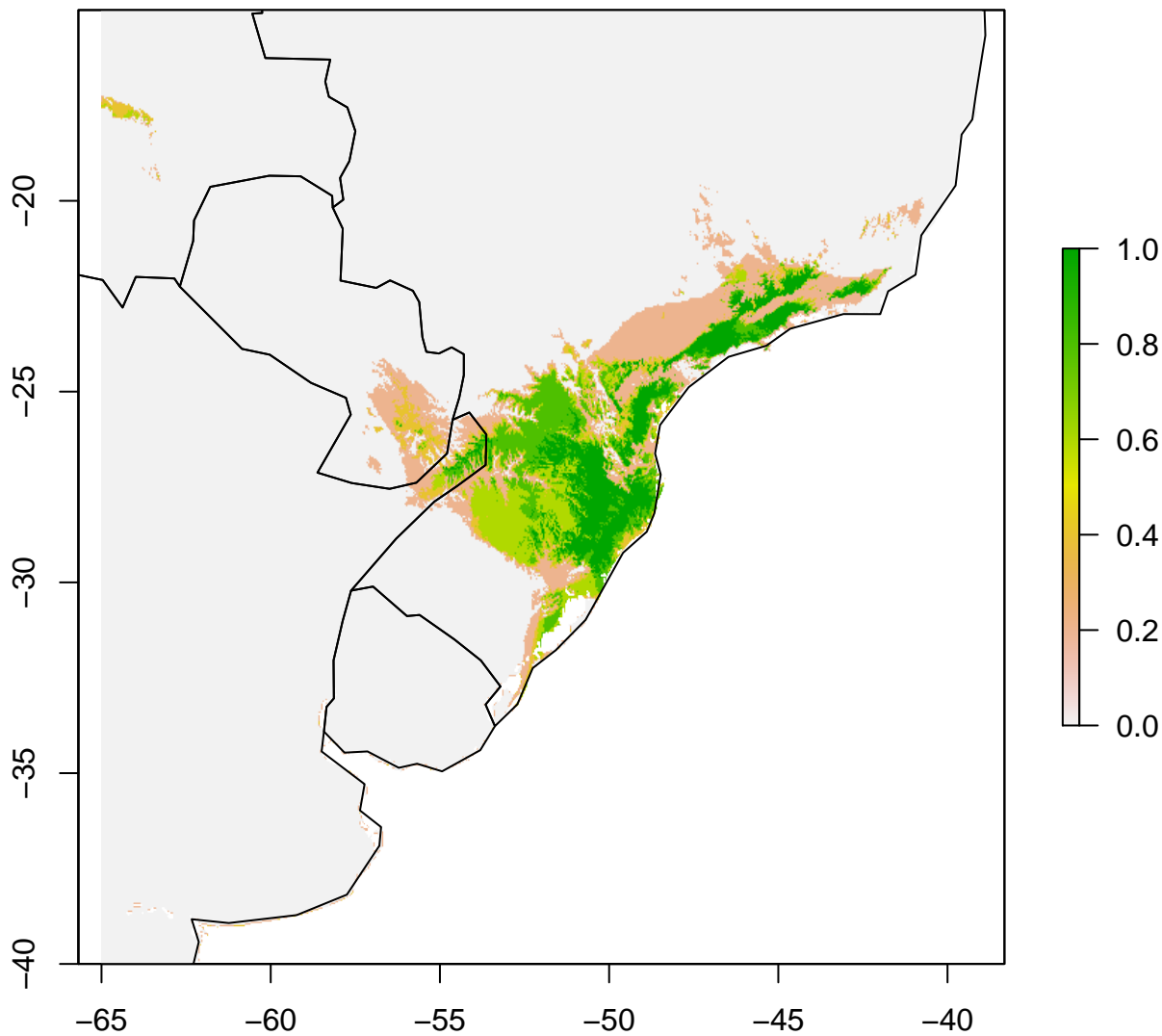

Supplement: Supplementary file 7 — Supplementary Data 4 [file 41467_2021_26537_MOESM7_ESM.gz › Dataset S1/obsoletus_stability_map.pdf]

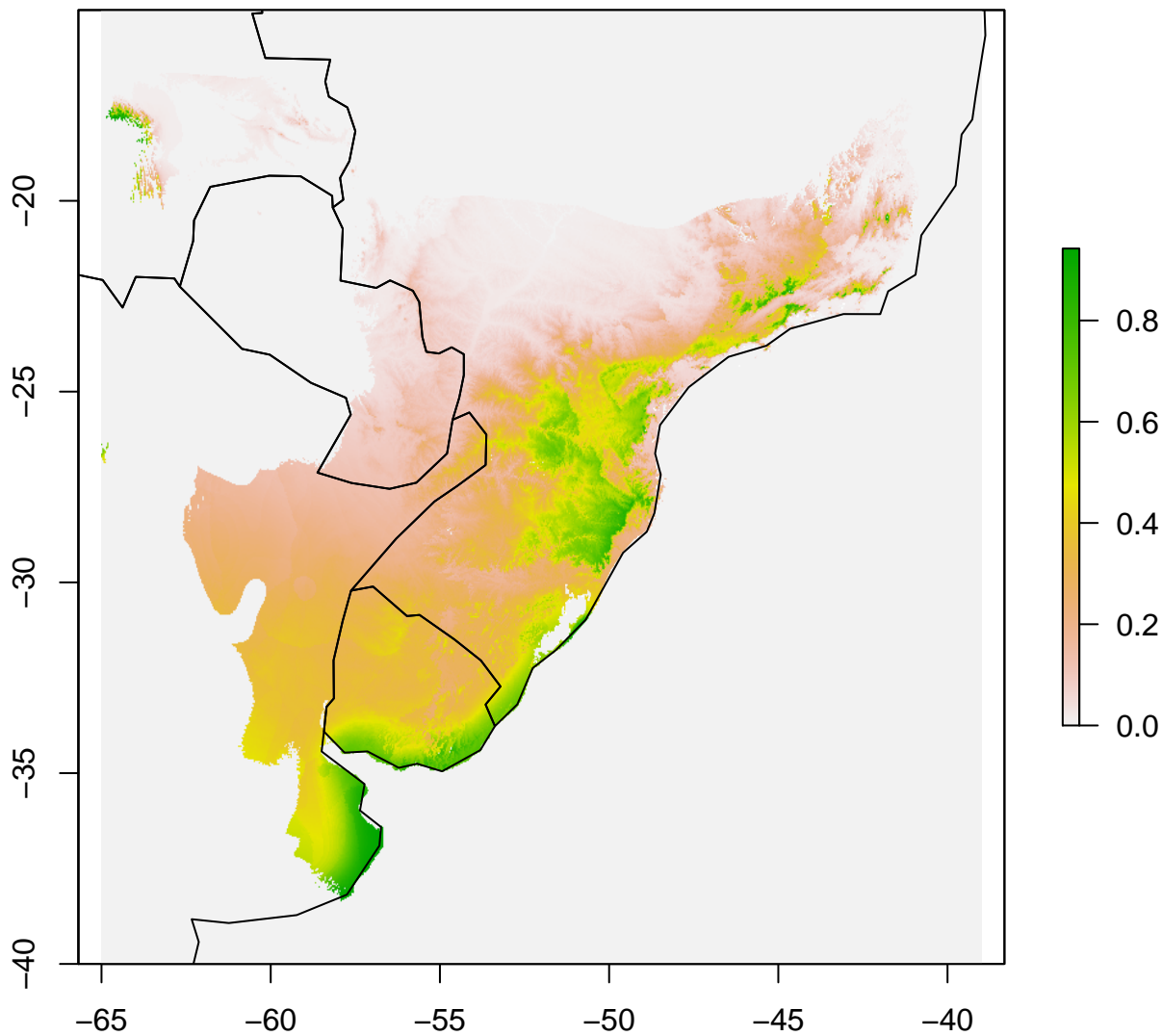

Supplement: Supplementary file 7 — Supplementary Data 4 [file 41467_2021_26537_MOESM7_ESM.gz › Dataset S1/lateralis_projection_MESS]

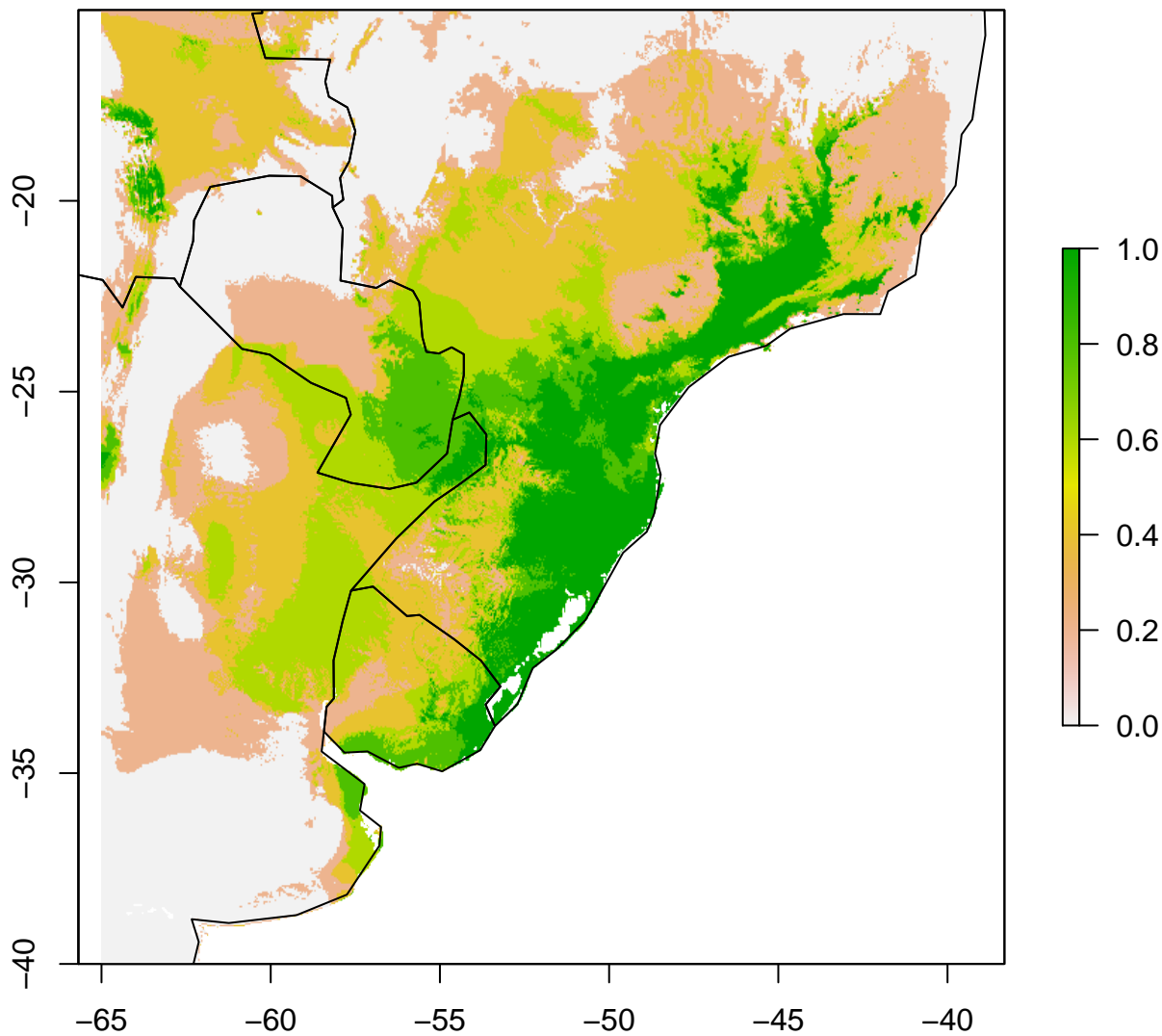

Supplement: Supplementary file 7 — Supplementary Data 4 [file 41467_2021_26537_MOESM7_ESM.gz › Dataset S1/mesoleuca_stability_map.pdf]

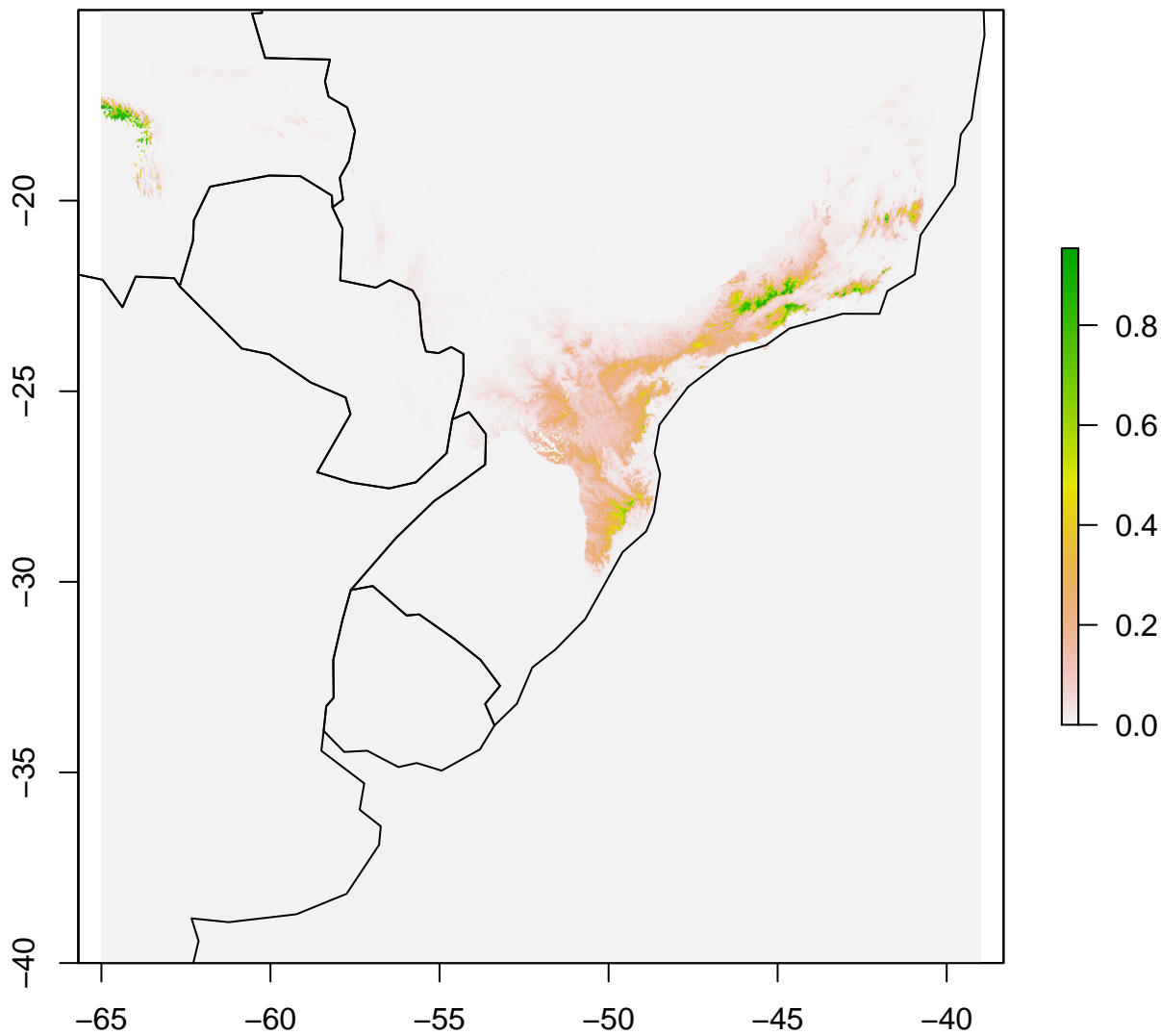

Supplement: Supplementary file 7 — Supplementary Data 4 [file 41467_2021_26537_MOESM7_ESM.gz › Dataset S1/difficilis_projection_MESS]

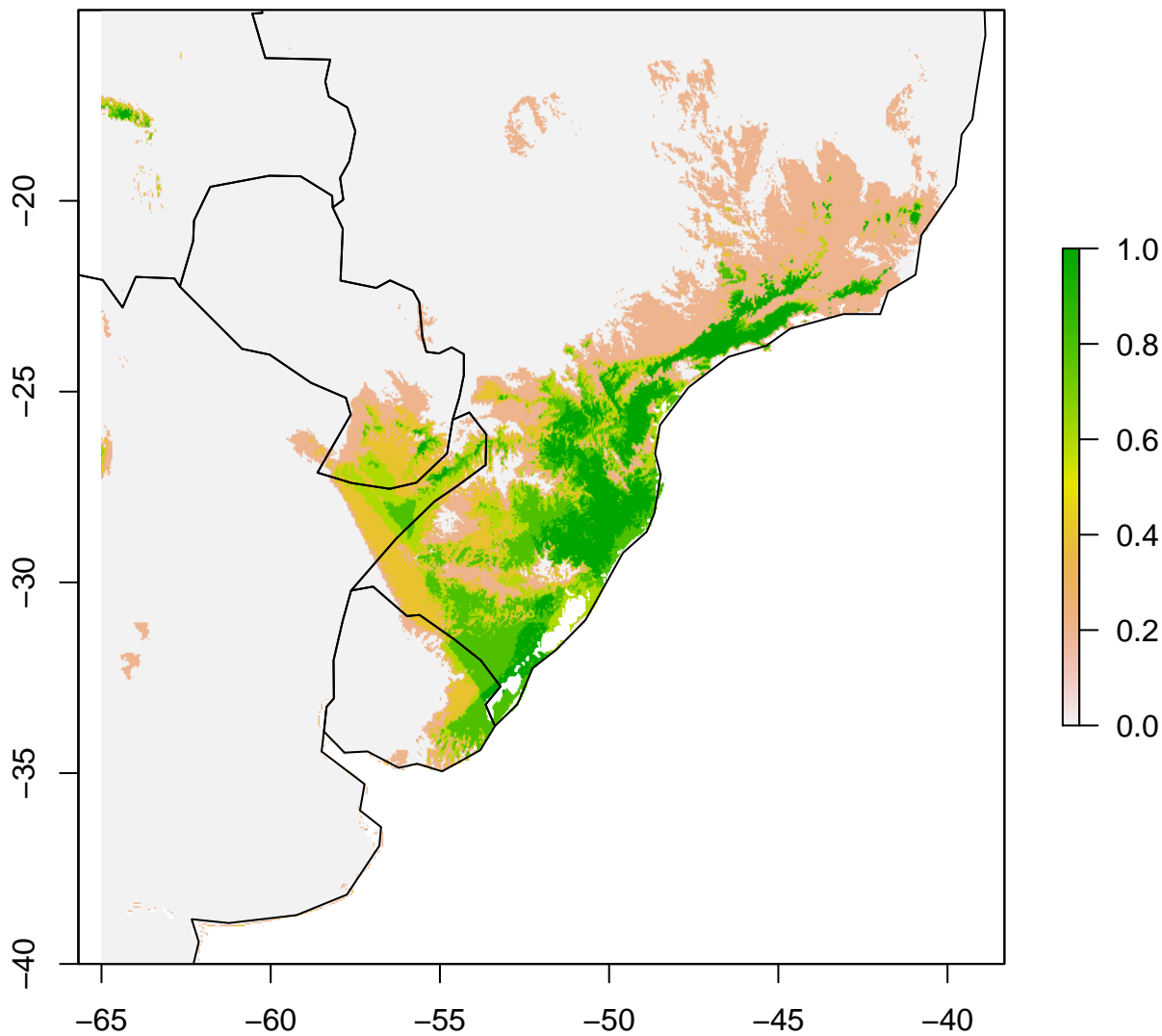

Supplement: Supplementary file 7 — Supplementary Data 4 [file 41467_2021_26537_MOESM7_ESM.gz › Dataset S1/contaminatus_stability_map.pdf]

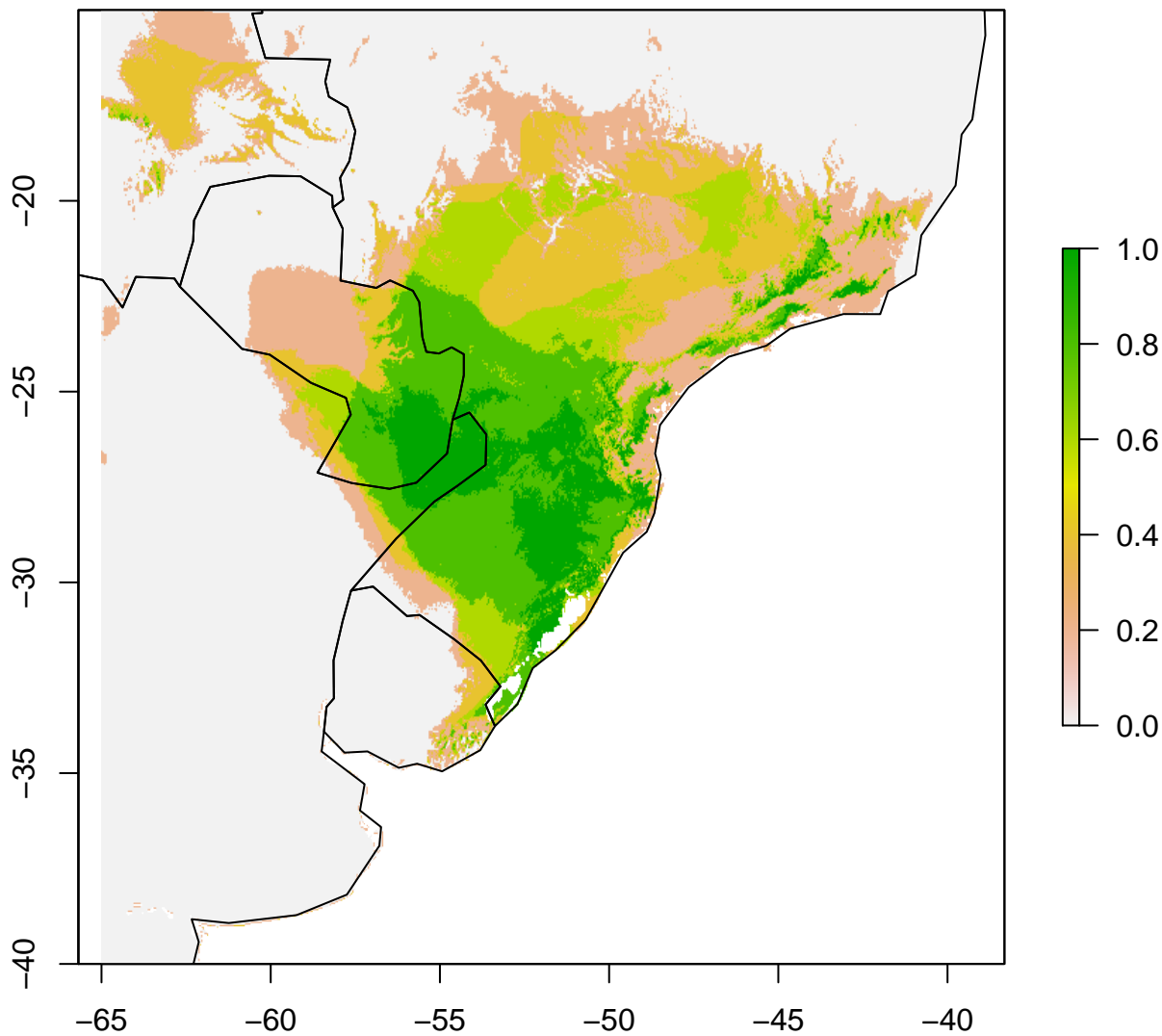

Supplement: Supplementary file 7 — Supplementary Data 4 [file 41467_2021_26537_MOESM7_ESM.gz › Dataset S1/lalandi_stability_map.pdf]

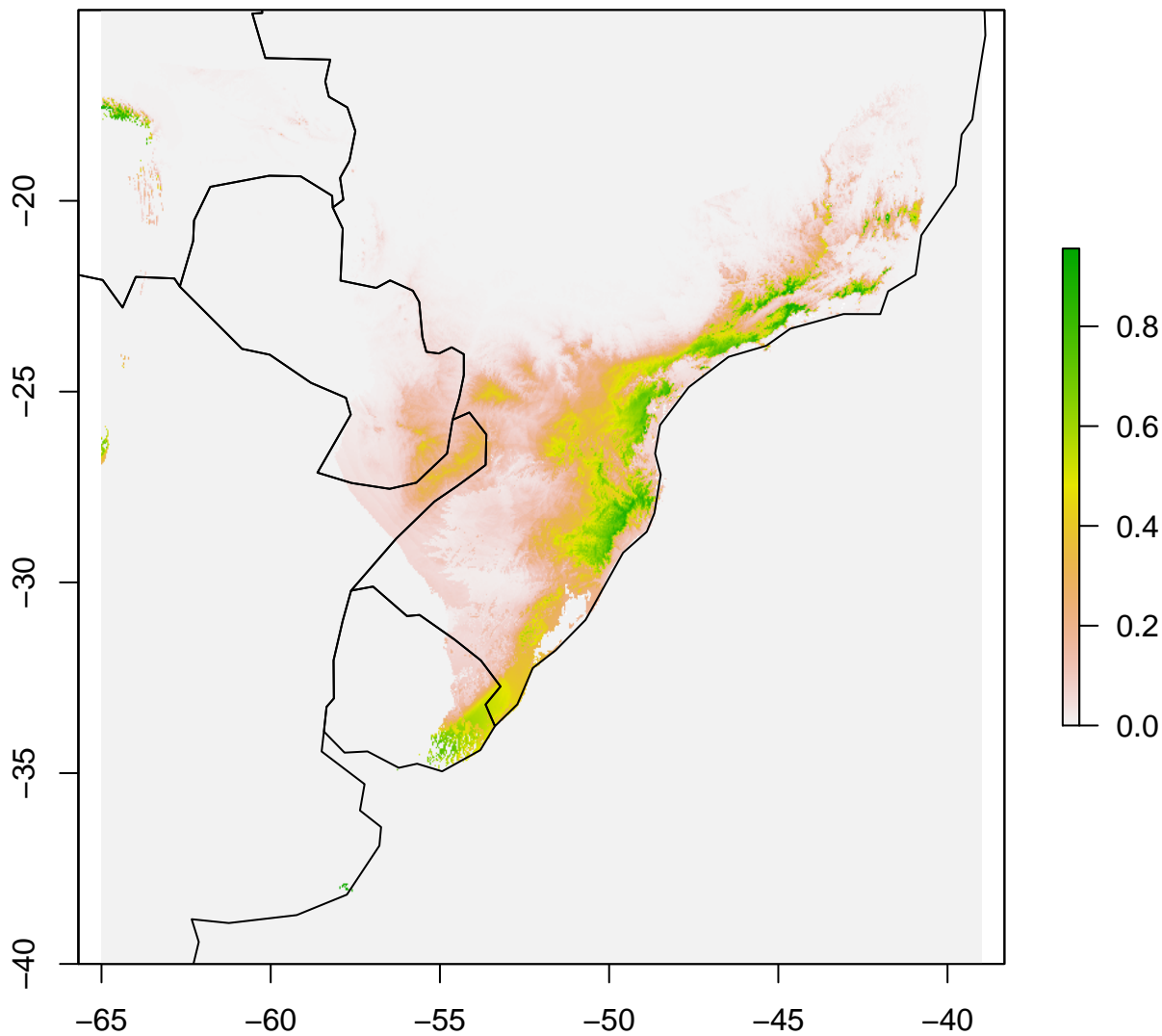

Supplement: Supplementary file 7 — Supplementary Data 4 [file 41467_2021_26537_MOESM7_ESM.gz › Dataset S1/contaminatus_projection_MESS]

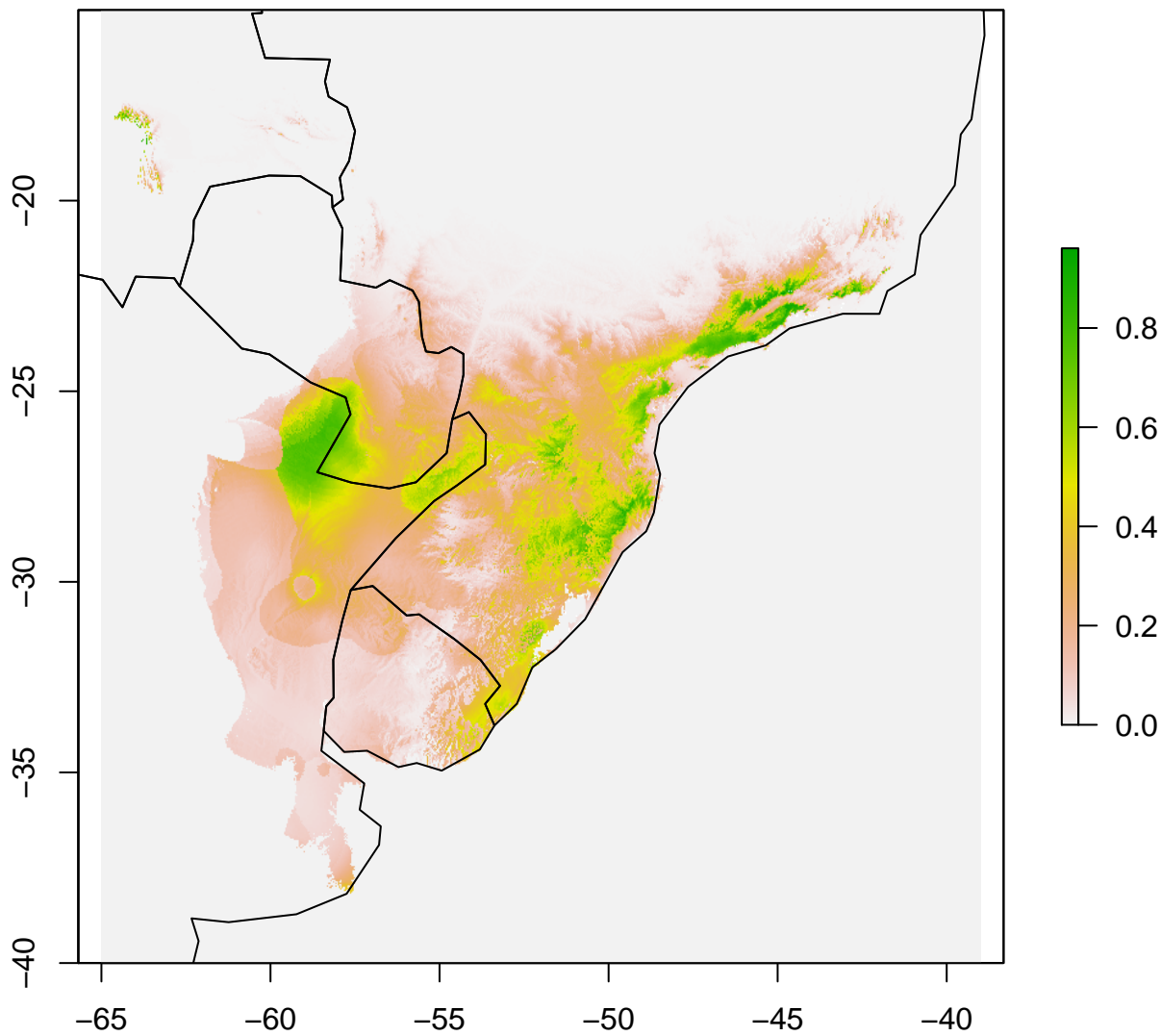

Supplement: Supplementary file 7 — Supplementary Data 4 [file 41467_2021_26537_MOESM7_ESM.gz › Dataset S1/chrysopterus_projection_MESS]

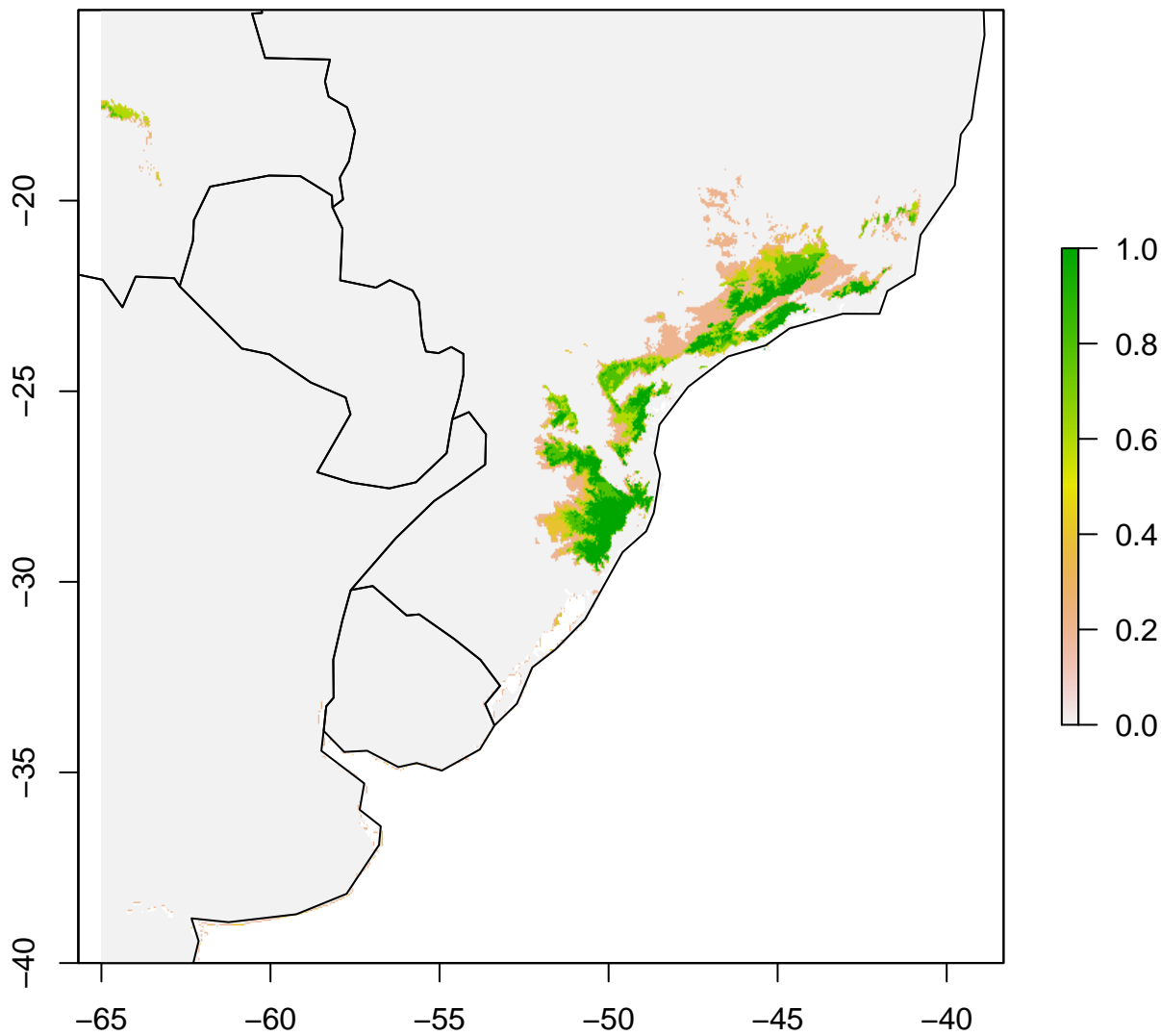

Supplement: Supplementary file 7 — Supplementary Data 4 [file 41467_2021_26537_MOESM7_ESM.gz › Dataset S1/thoracica_stability_map.pdf]

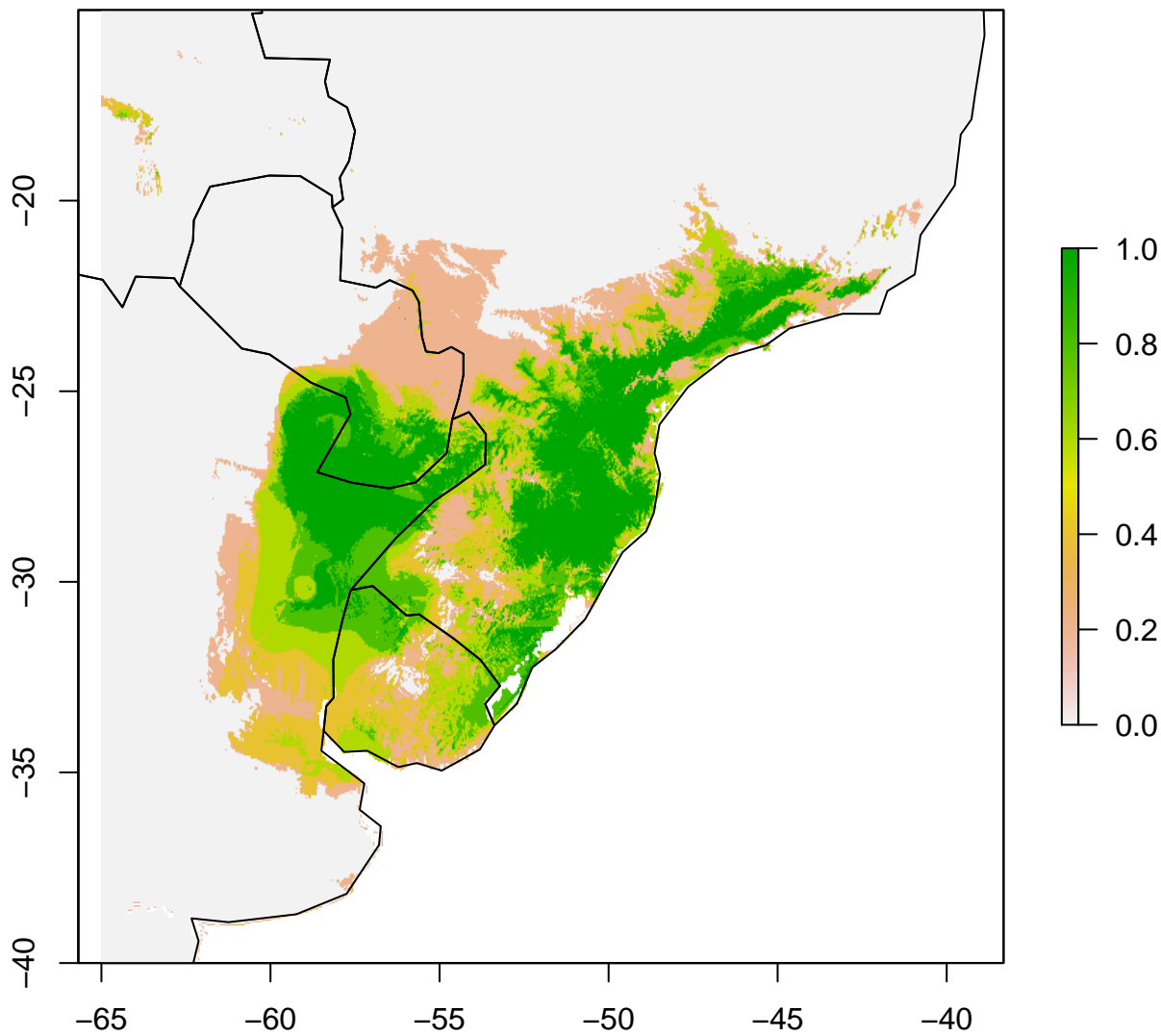

Supplement: Supplementary file 7 — Supplementary Data 4 [file 41467_2021_26537_MOESM7_ESM.gz › Dataset S1/chrysopterus_stability_map.pdf]

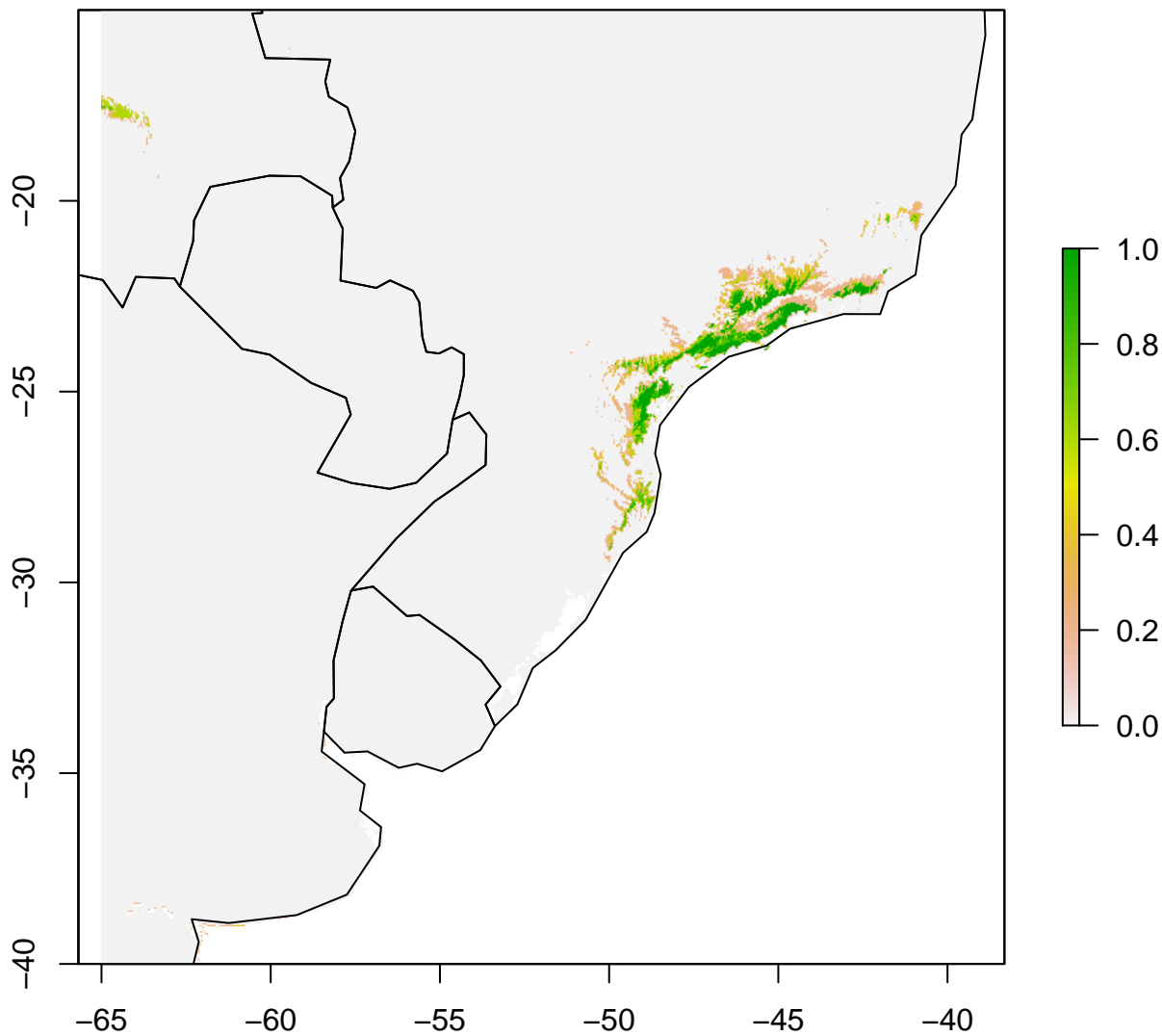

Supplement: Supplementary file 7 — Supplementary Data 4 [file 41467_2021_26537_MOESM7_ESM.gz › Dataset S1/xanthopterus_stability_map.pdf]

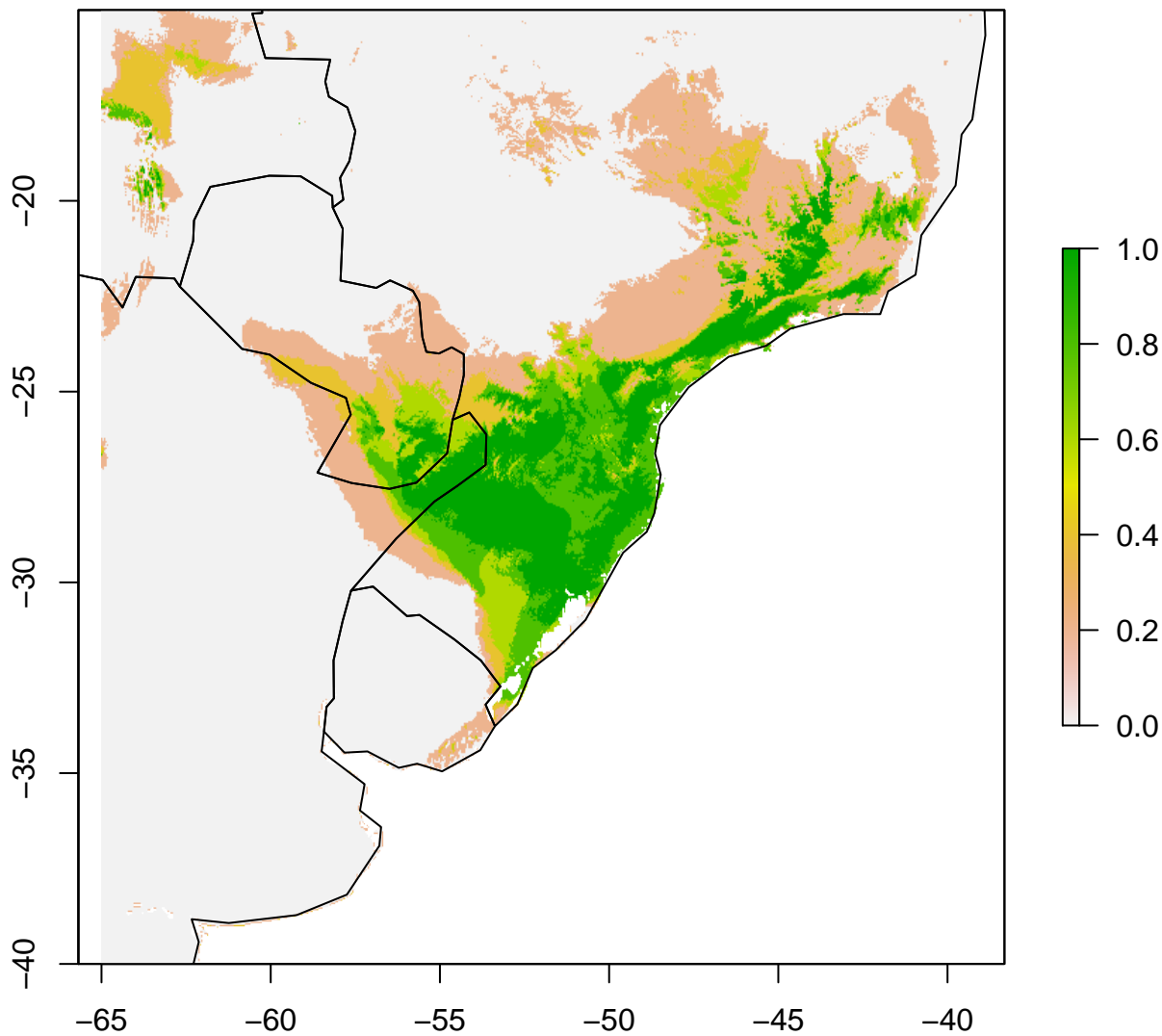

Supplement: Supplementary file 7 — Supplementary Data 4 [file 41467_2021_26537_MOESM7_ESM.gz › Dataset S1/cinerascens_stability_map.pdf]

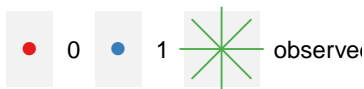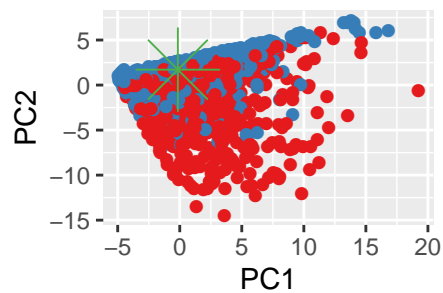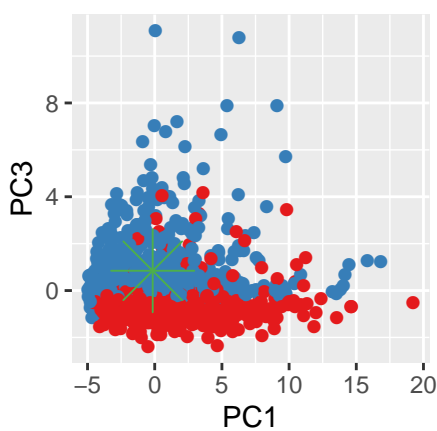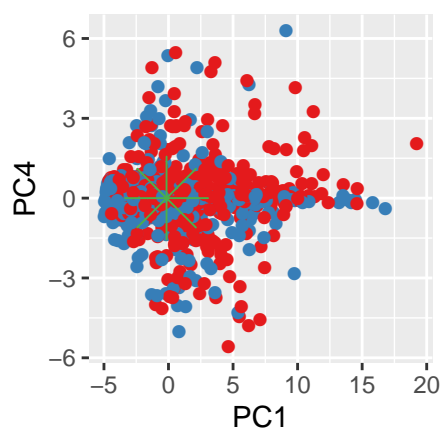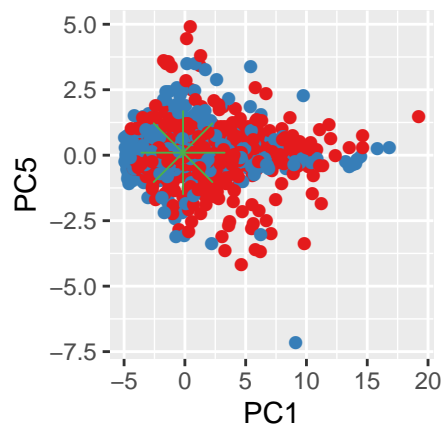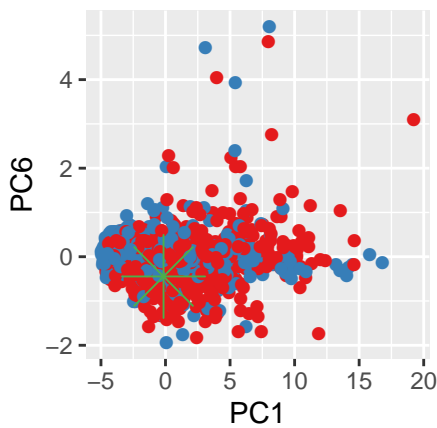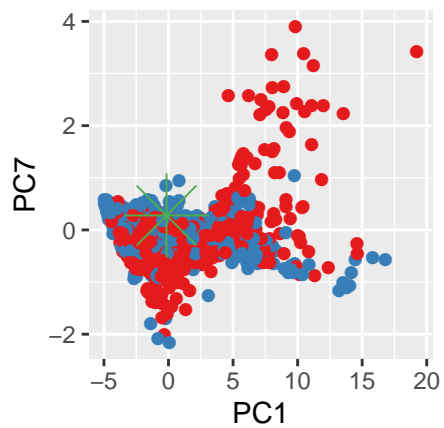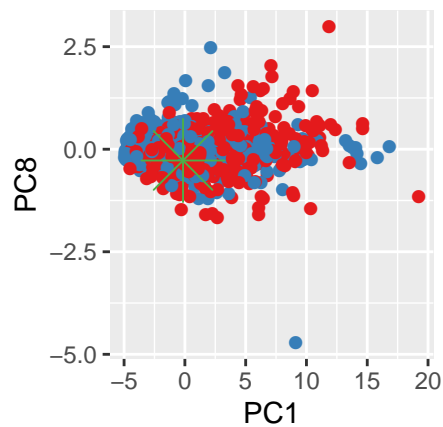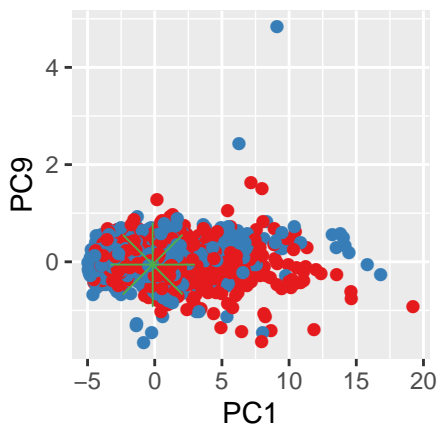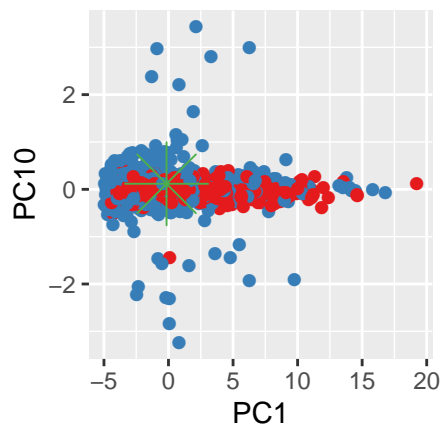

# Phylloscartes ventralis Island

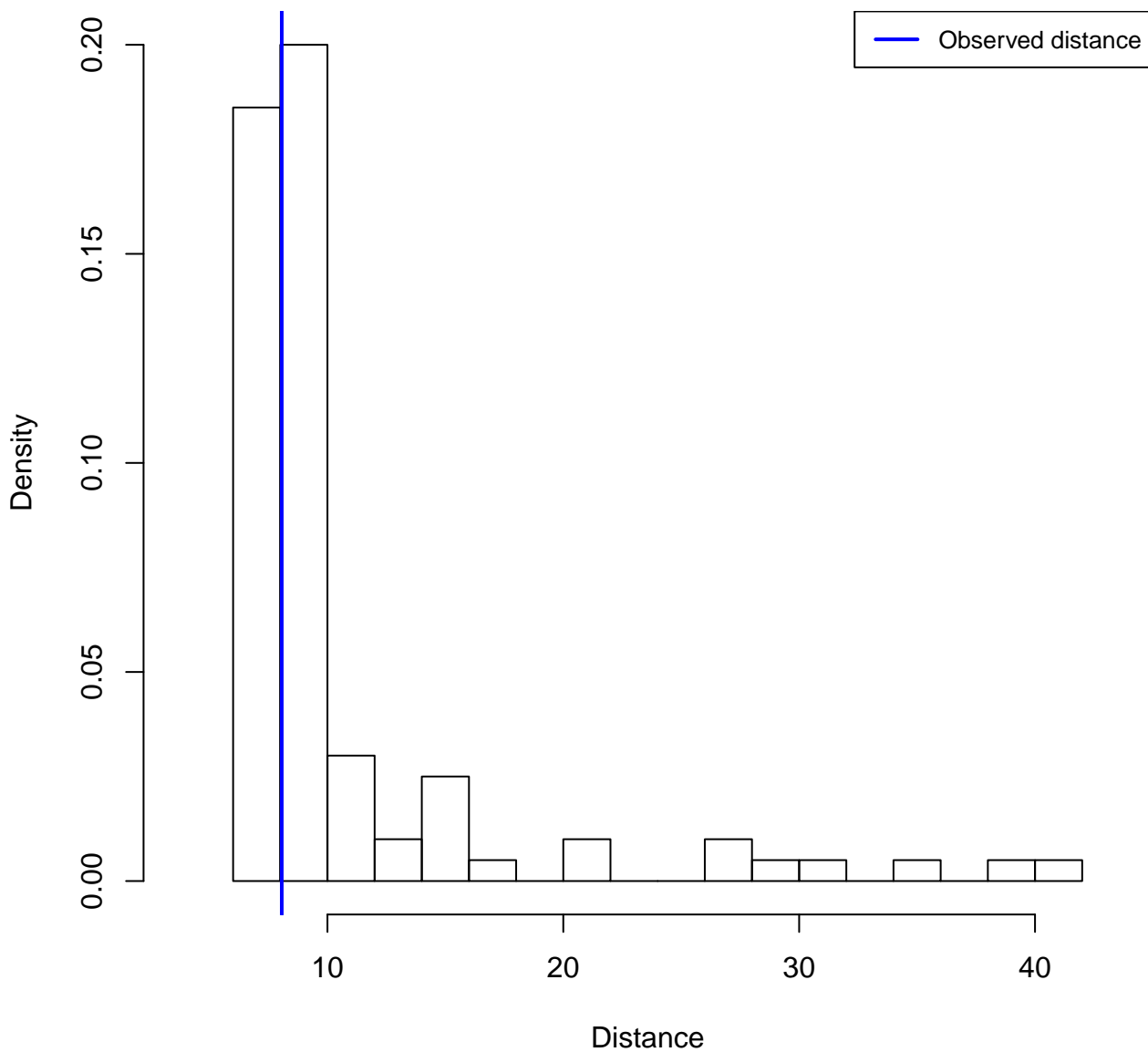

# Phylloscartes ventralis IBD

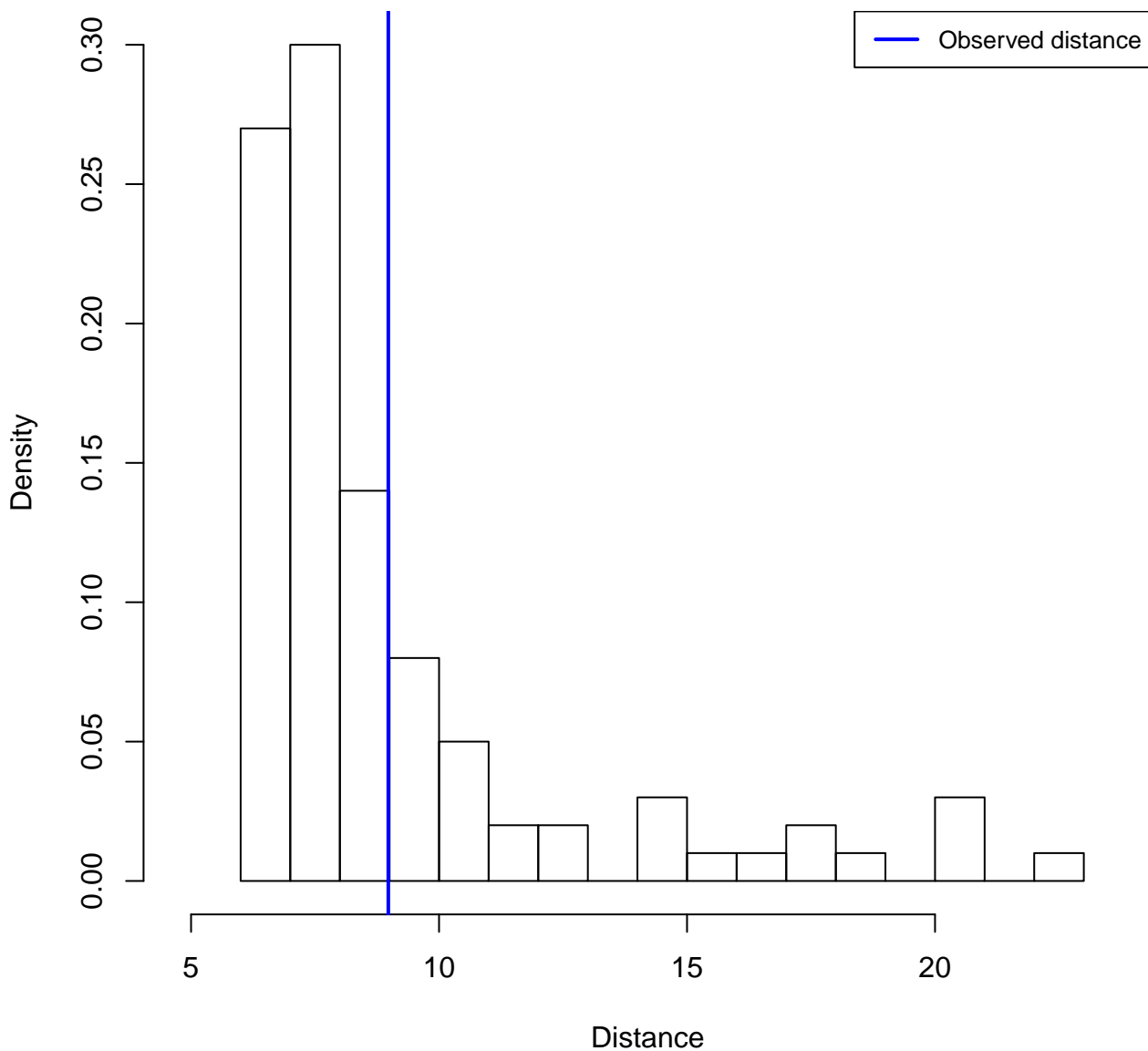

Supplement: Supplementary file 8 — Supplementary Data 5 [file 41467_2021_26537_MOESM8_ESM.gz › PCAs/ventralis_N_PCA.pdf]

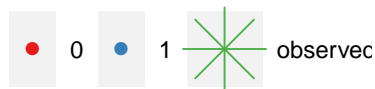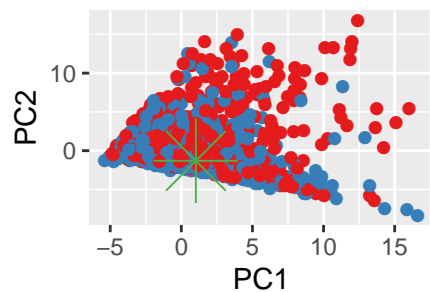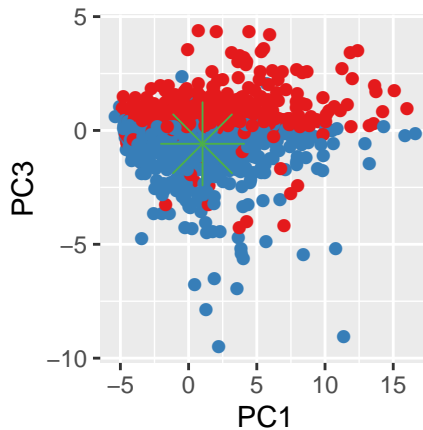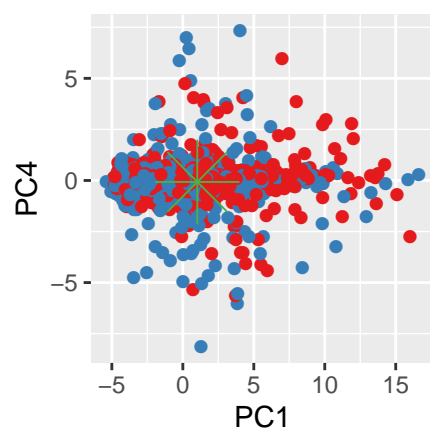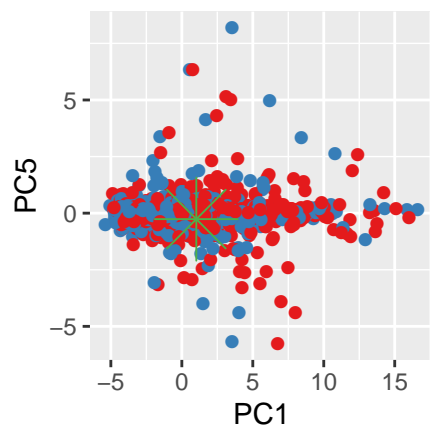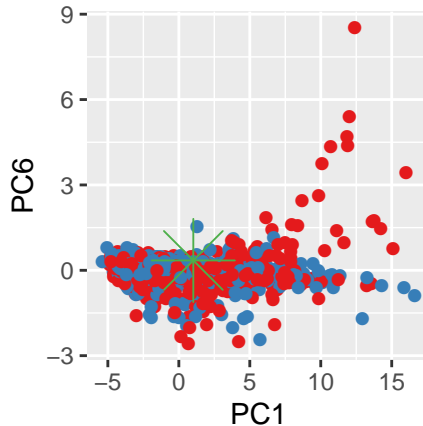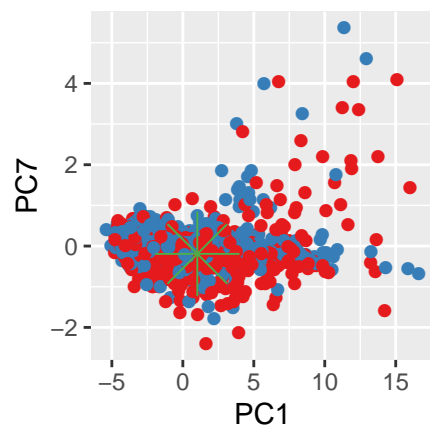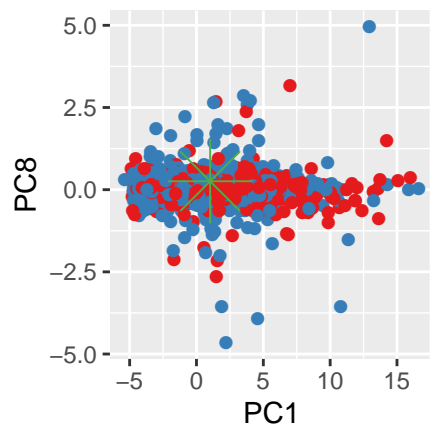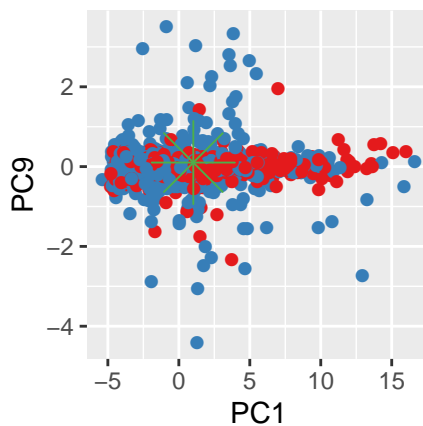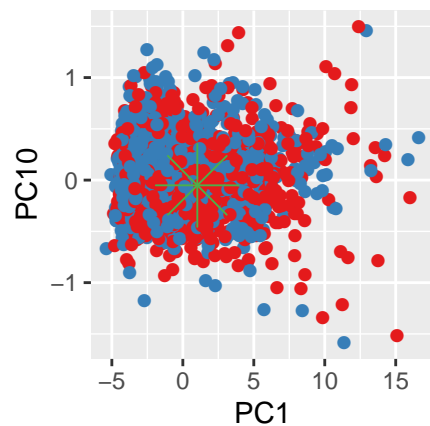

# Saltator maxillosus Island

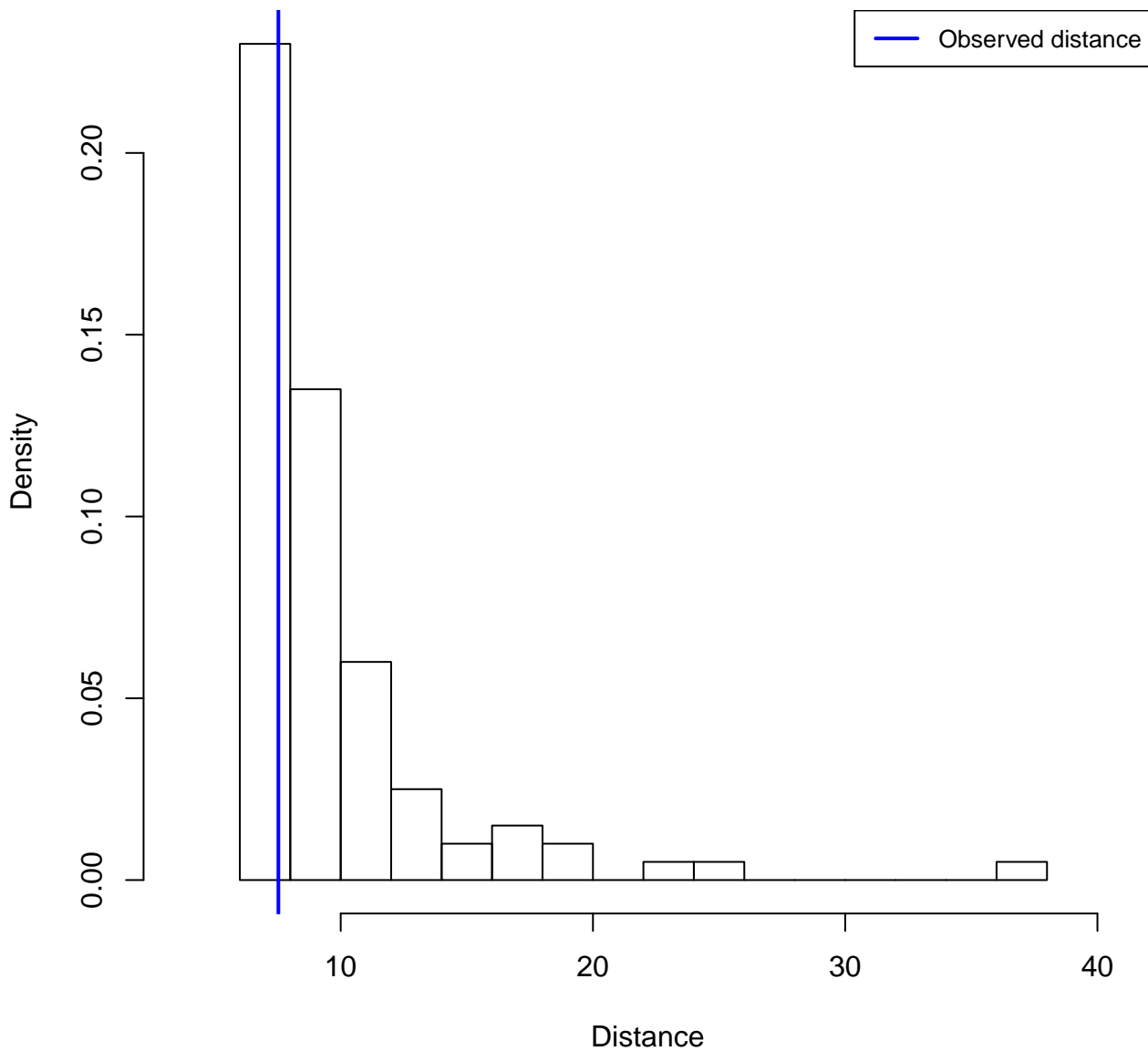

**Saltator maxillosus IBD**

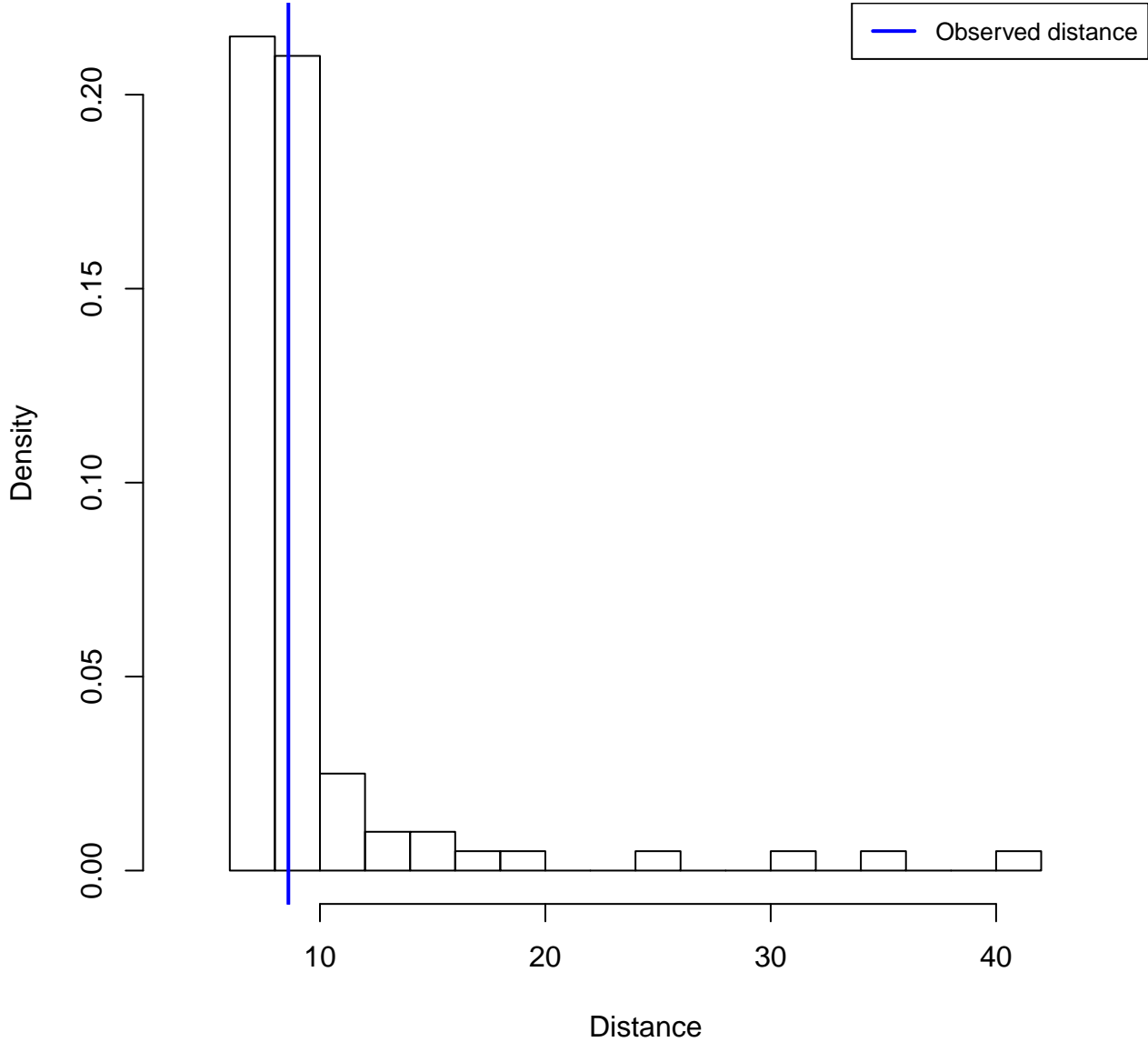

Supplement: Supplementary file 8 — Supplementary Data 5 [file 41467_2021_26537_MOESM8_ESM.gz › PCAs/maxillosus_N_PCA.pdf]

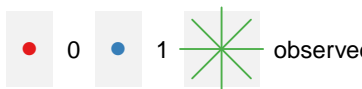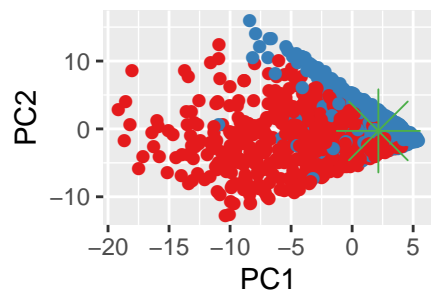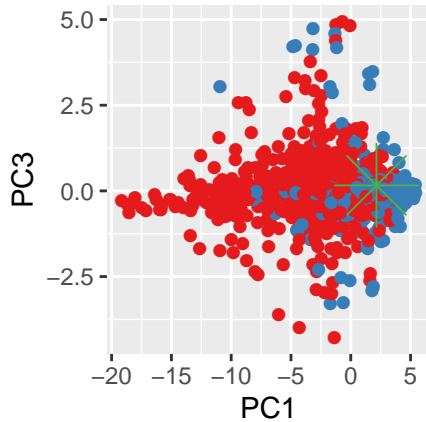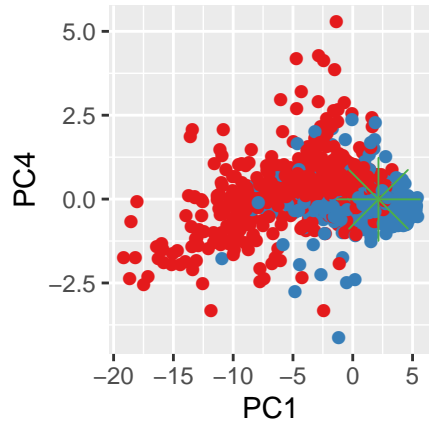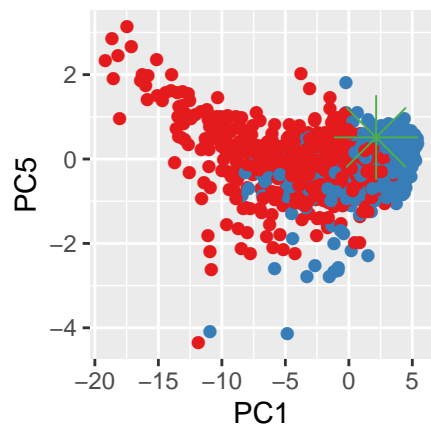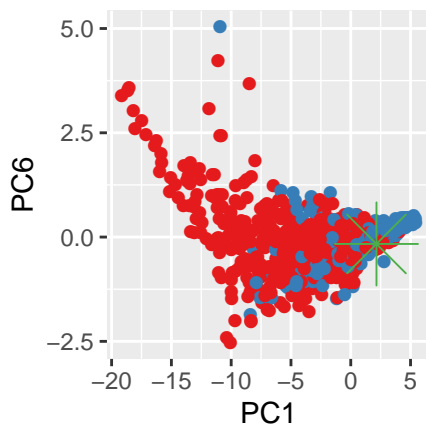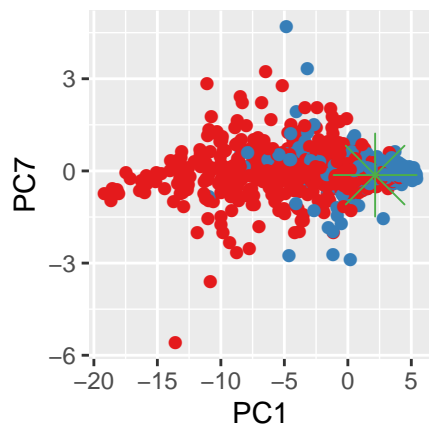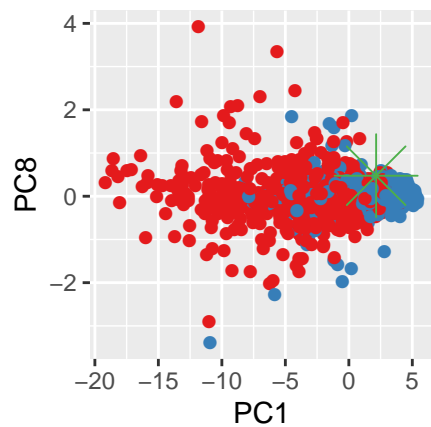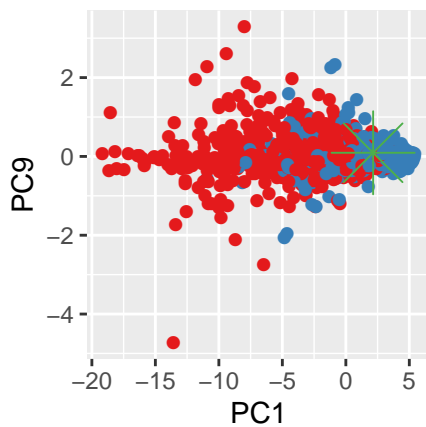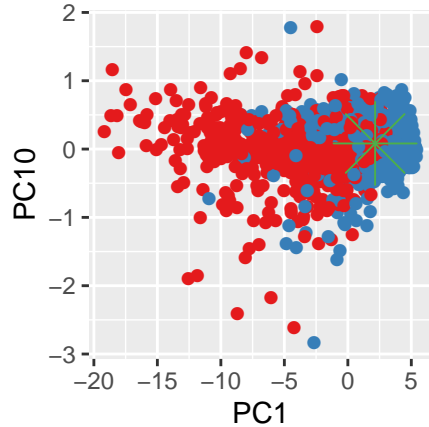

# Syndactyla rufosuperciliata Island

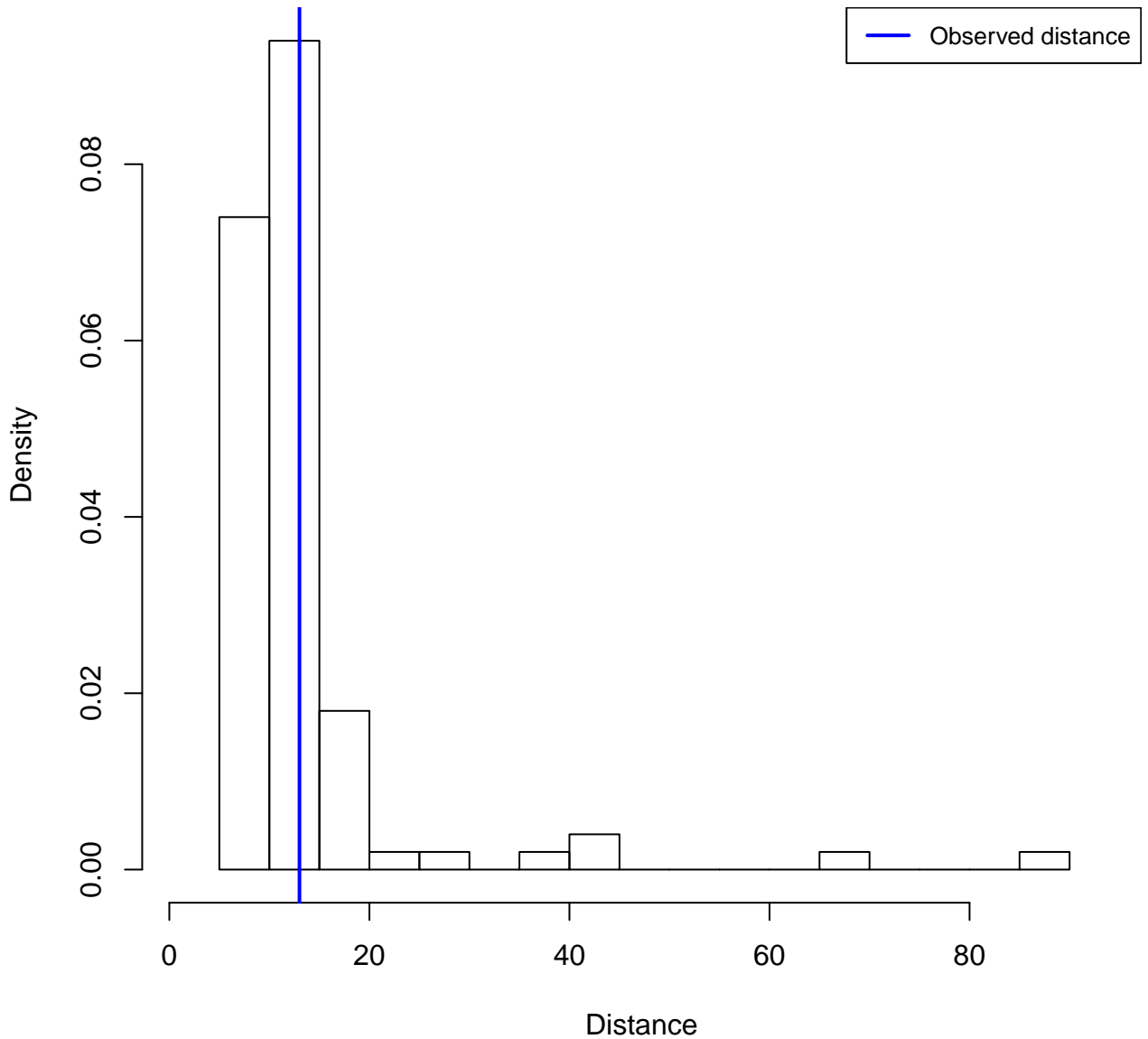

# Syndactyla rufosuperciliata IBD

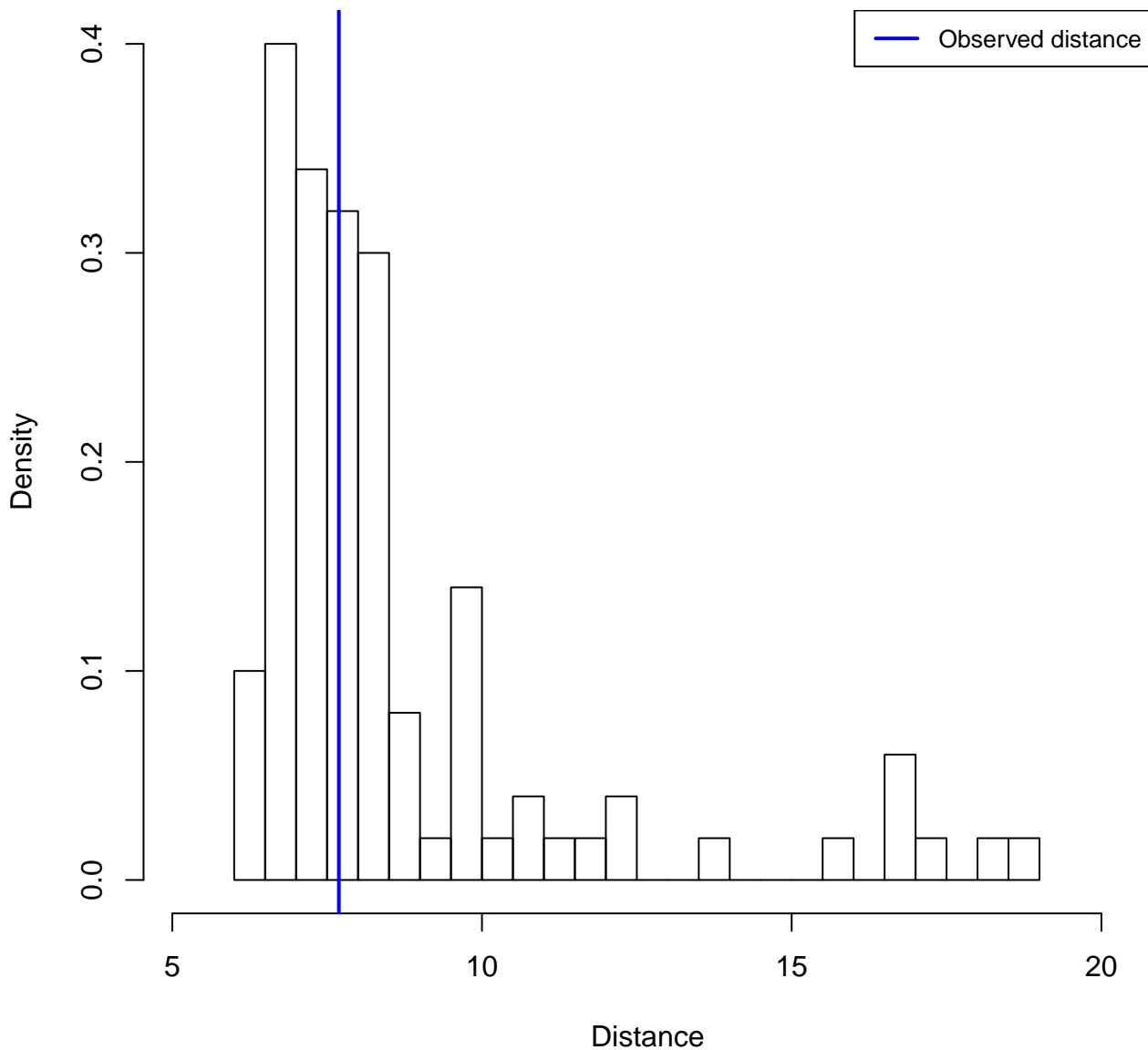

Supplement: Supplementary file 8 — Supplementary Data 5 [file 41467_2021_26537_MOESM8_ESM.gz › PCAs/rufosuperciliata_S_PCA.pdf]

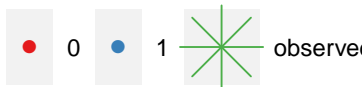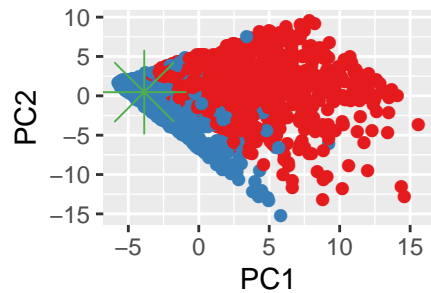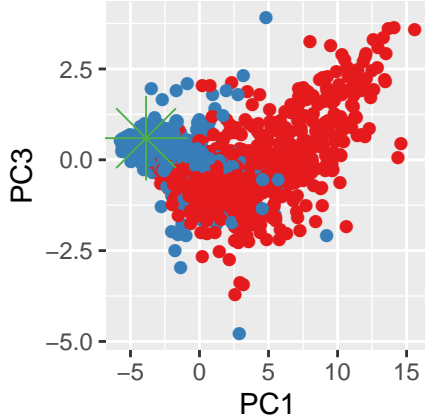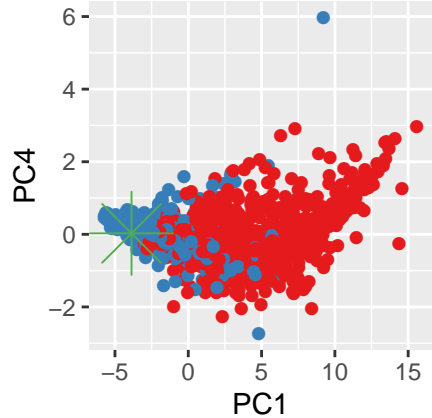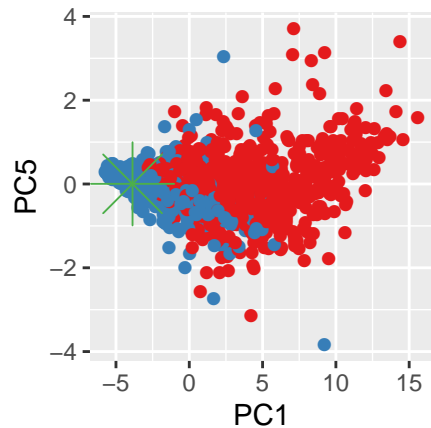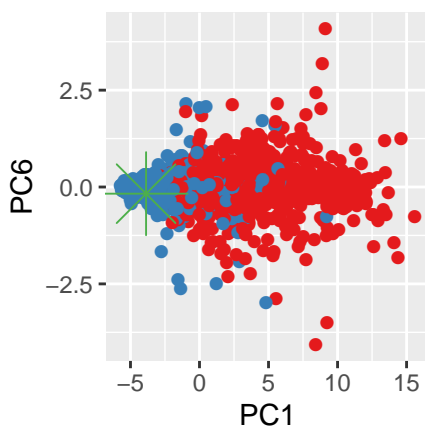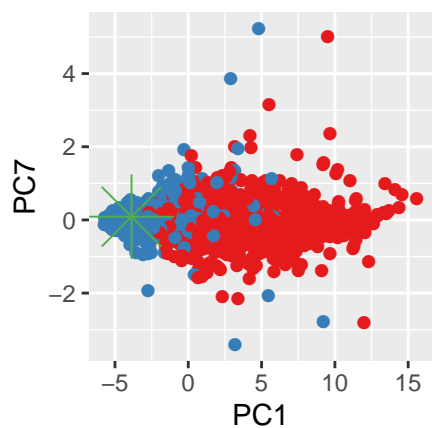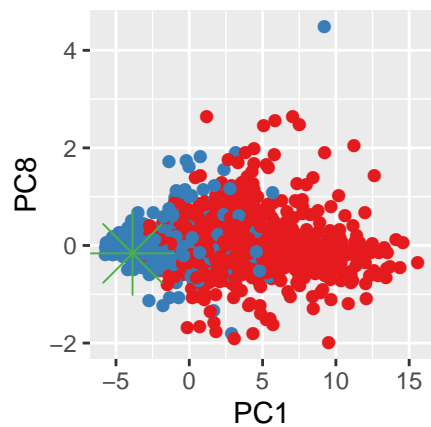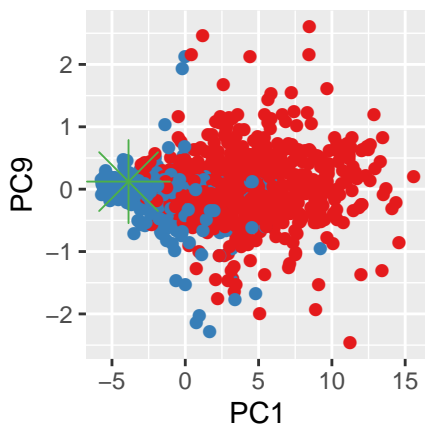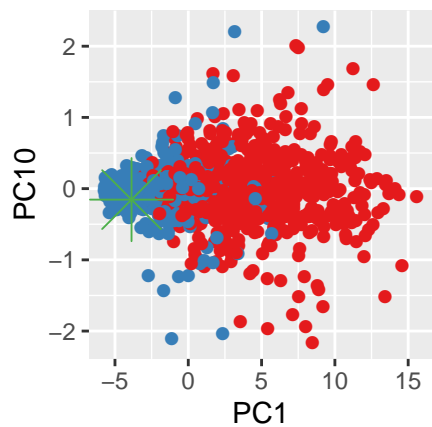

# Heliobletus contaminatus Island

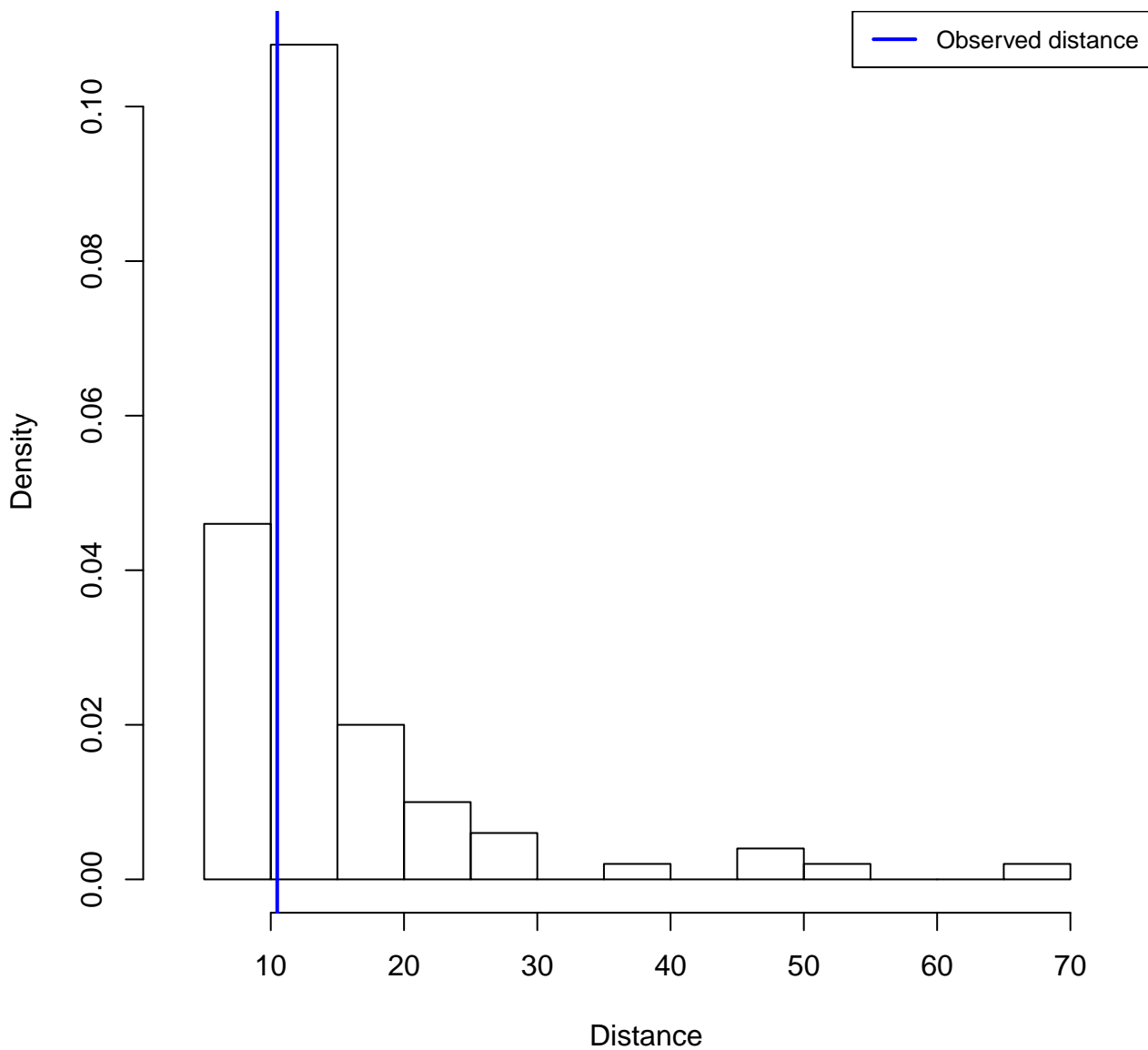

# Heliobletus contaminatus IBD

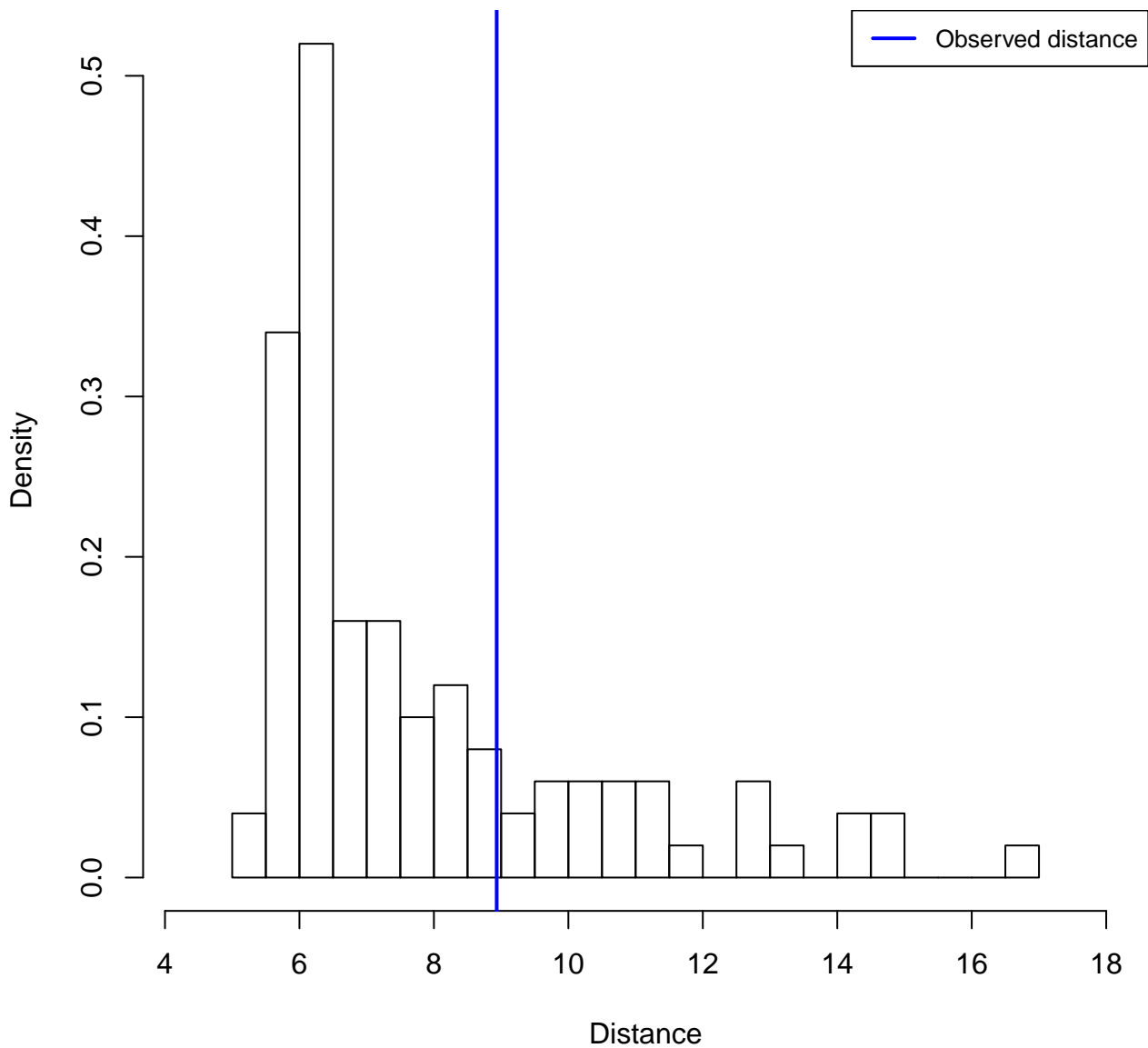

Supplement: Supplementary file 8 — Supplementary Data 5 [file 41467_2021_26537_MOESM8_ESM.gz › PCAs/contaminatus_S_PCA.pdf]

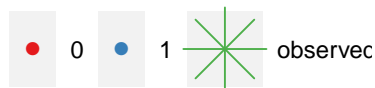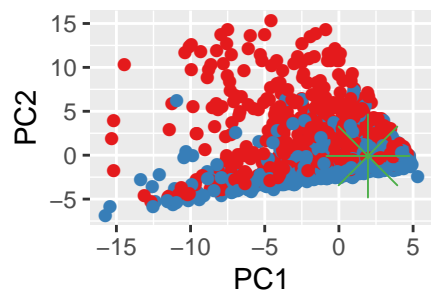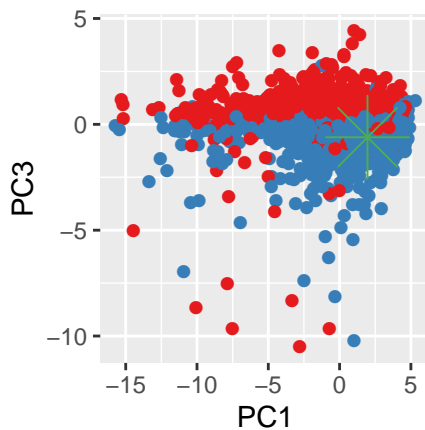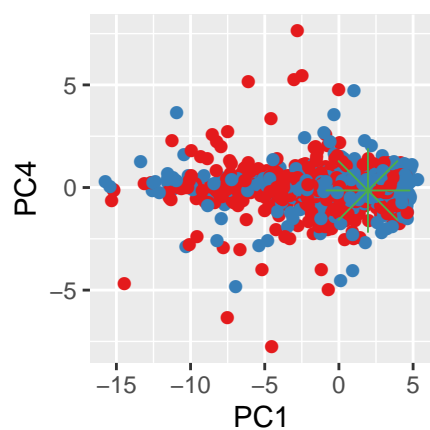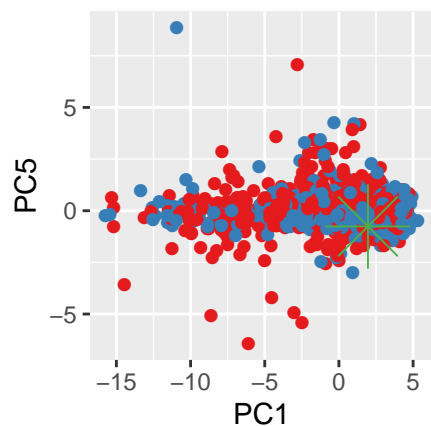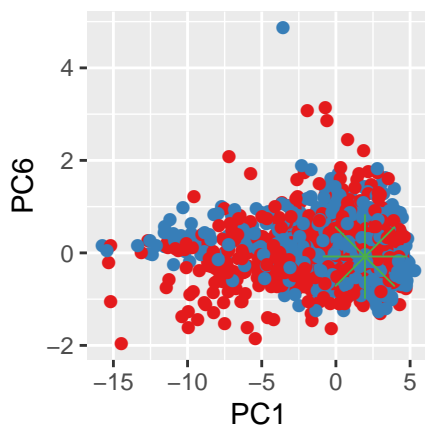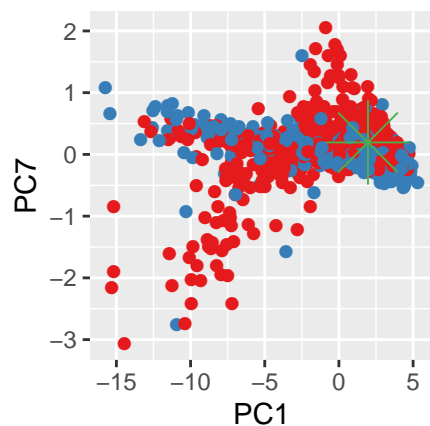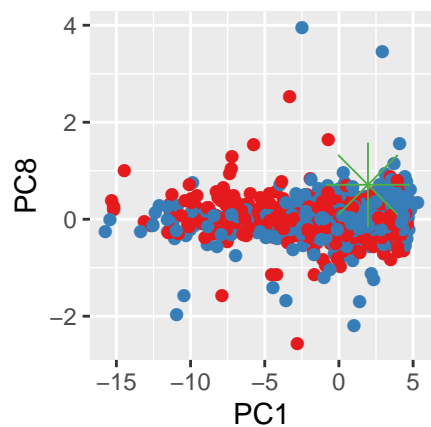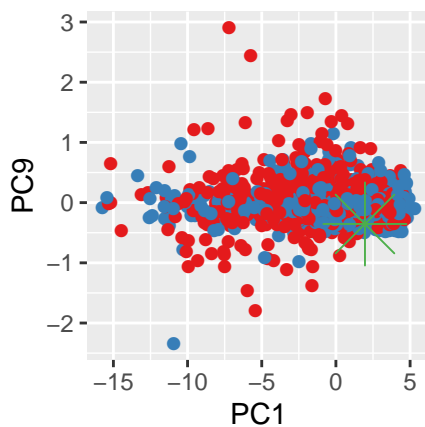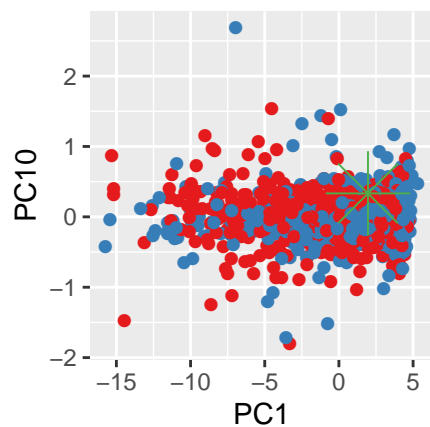

# Cacicus chrysipater Island

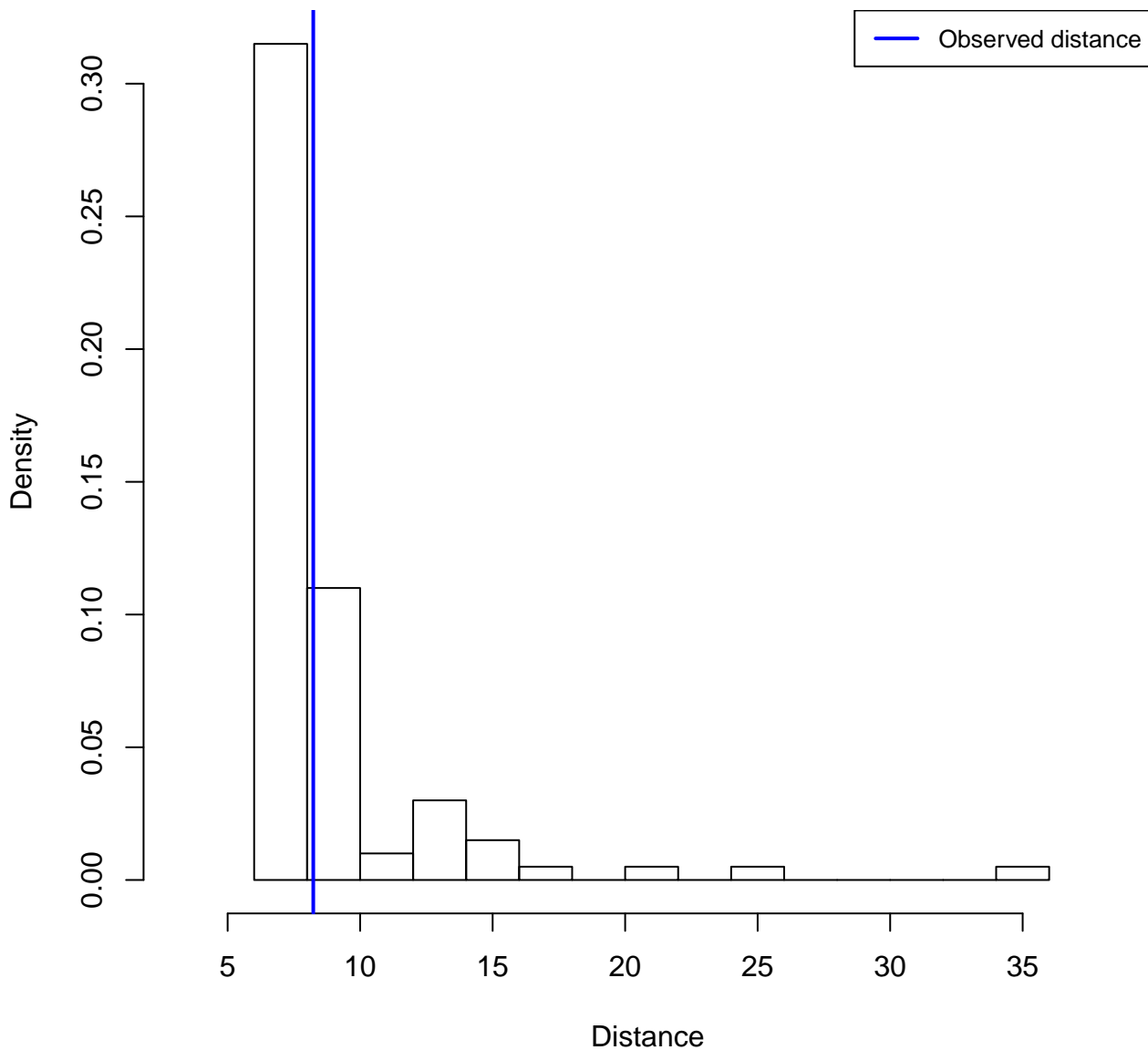

# Cacicus chrysipaterus IBD

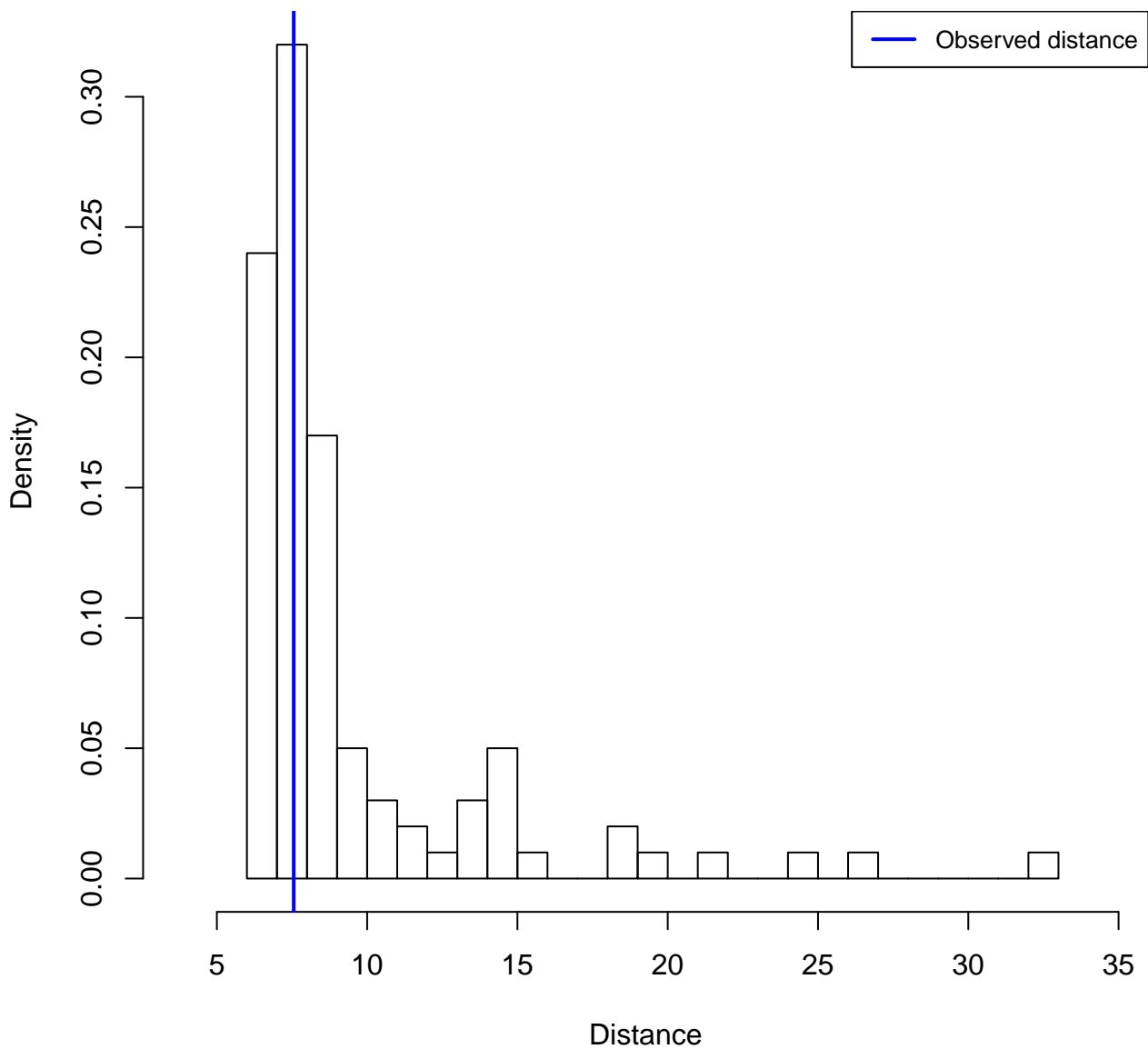

Supplement: Supplementary file 8 — Supplementary Data 5 [file 41467_2021_26537_MOESM8_ESM.gz › PCAs/chrysopterus_N_PCA.pdf]

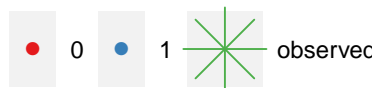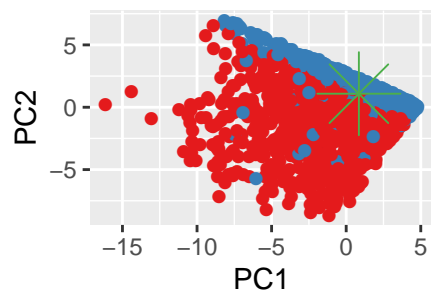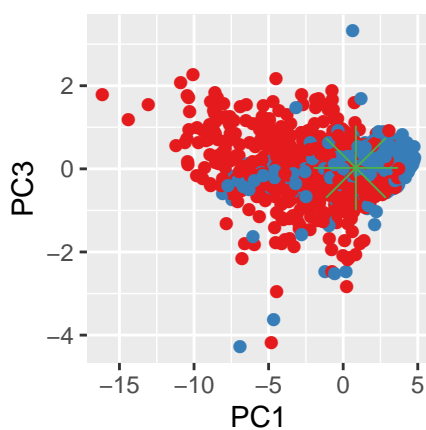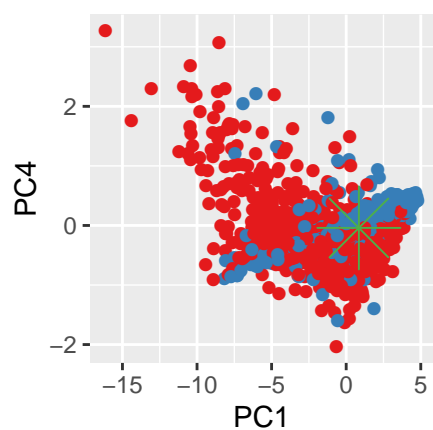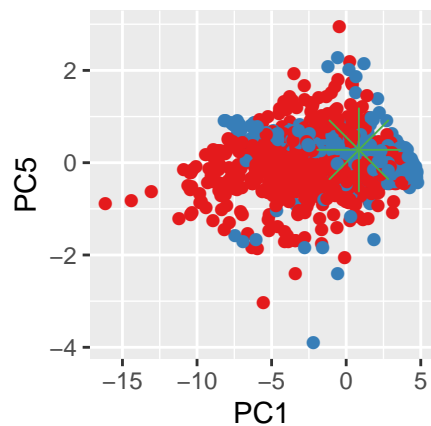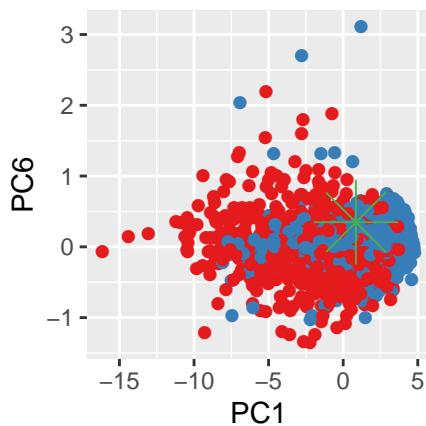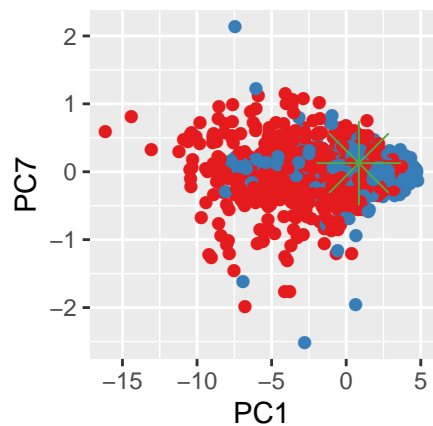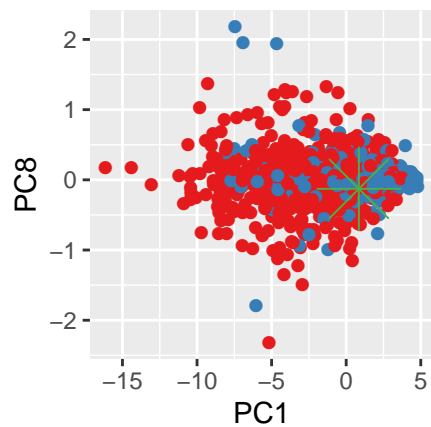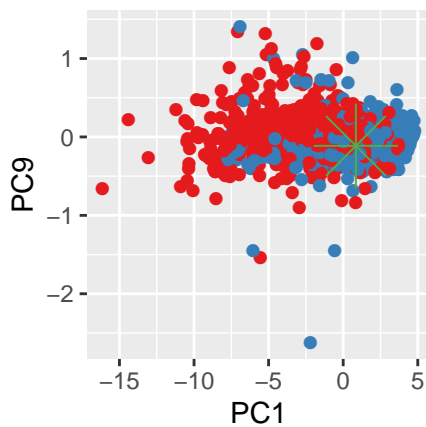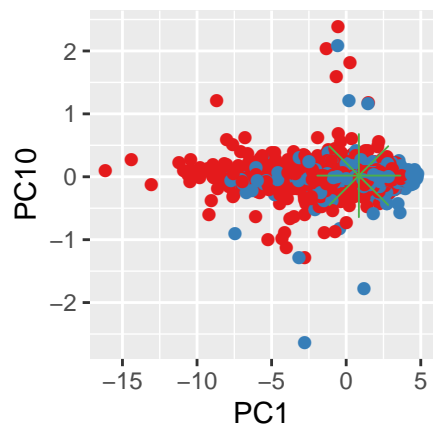

# Hemitriccus obsoletus Island

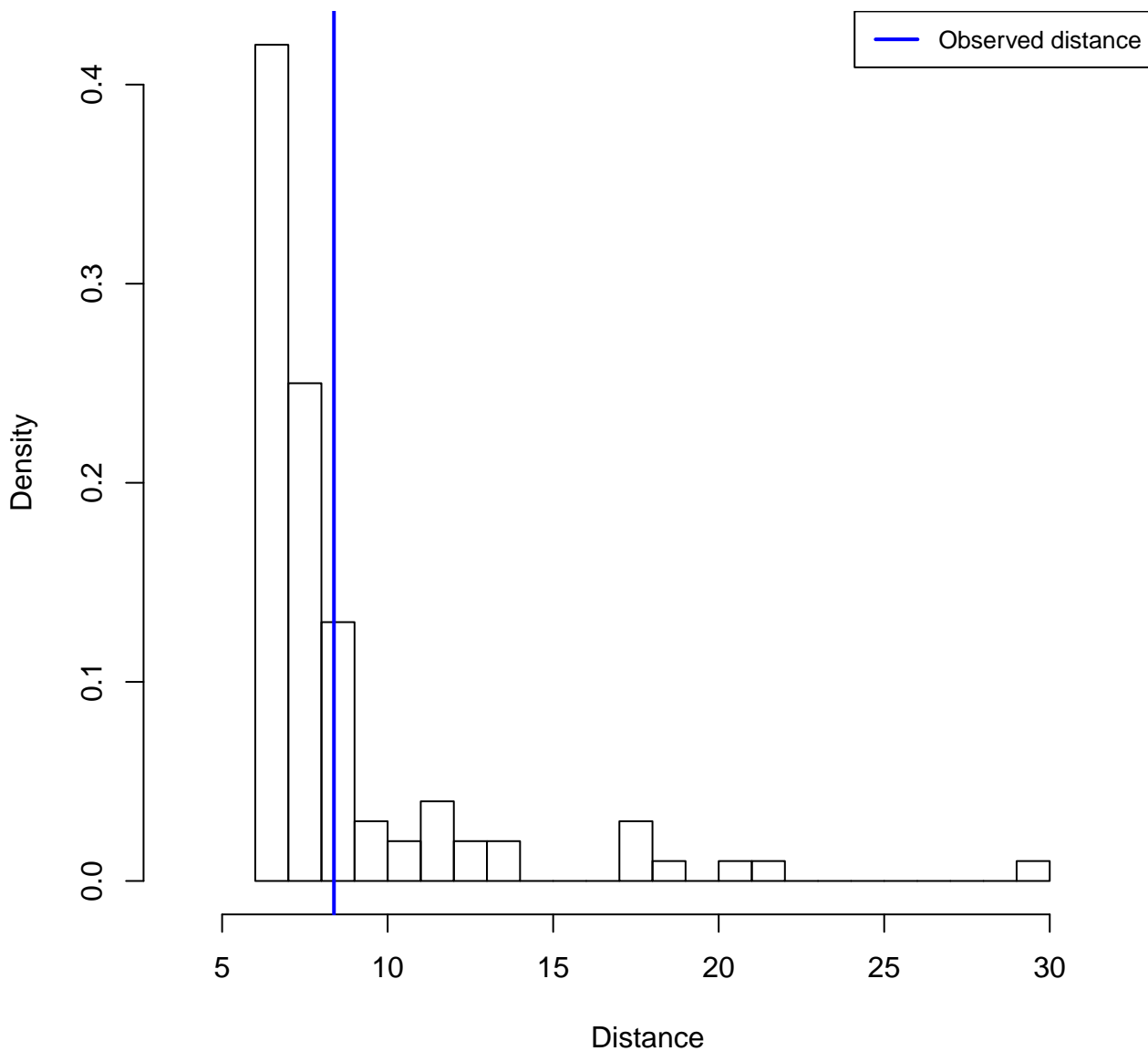

# Hemitriccus obsoletus IBD

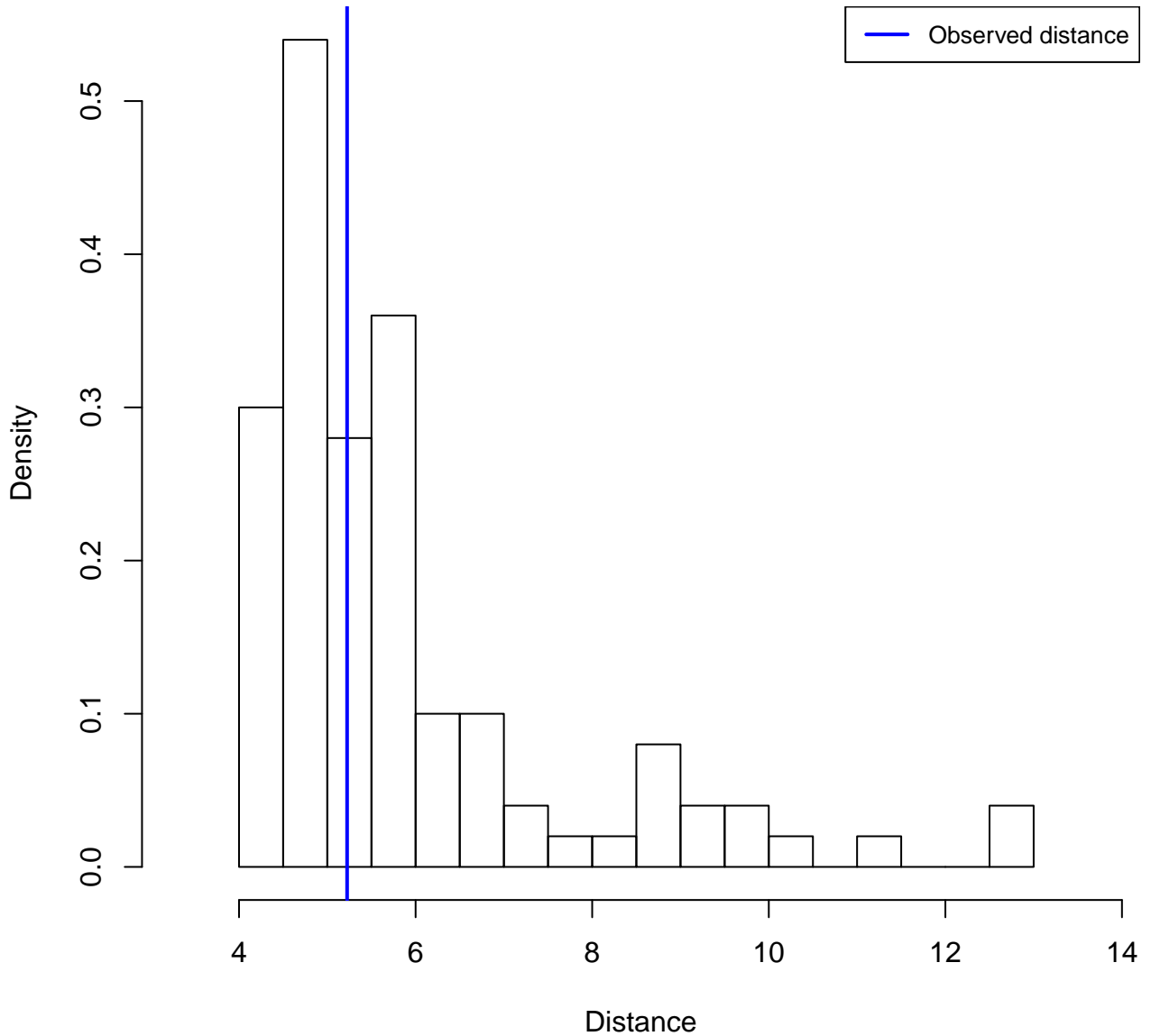

Supplement: Supplementary file 8 — Supplementary Data 5 [file 41467_2021_26537_MOESM8_ESM.gz › PCAs/obsoletus_S_PCA.pdf]

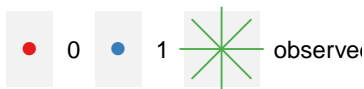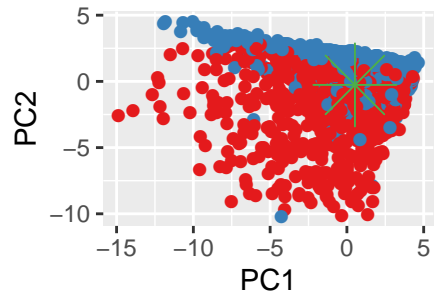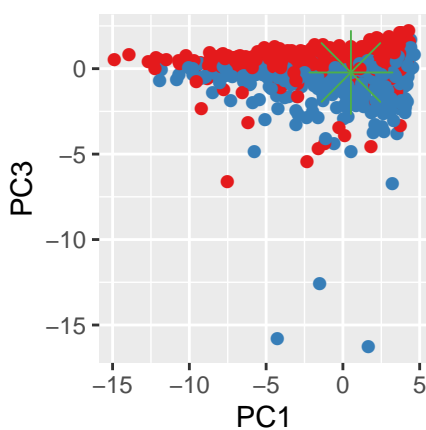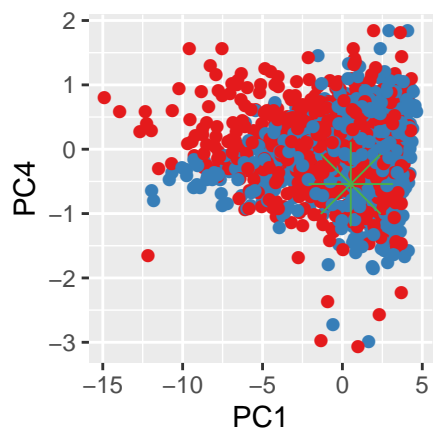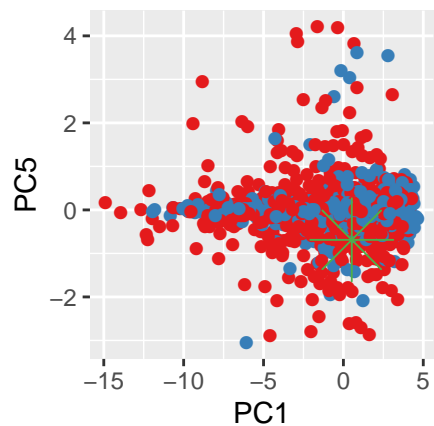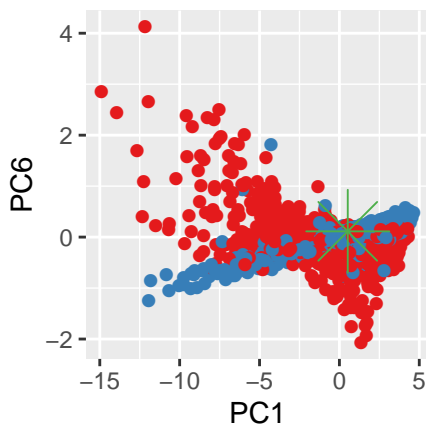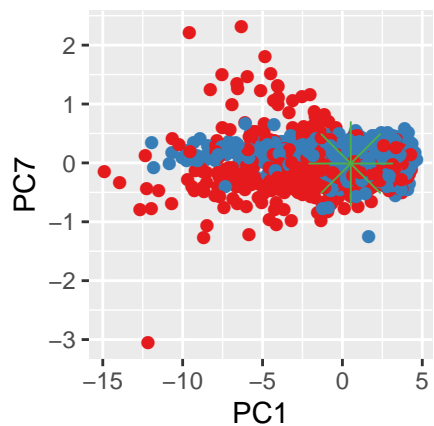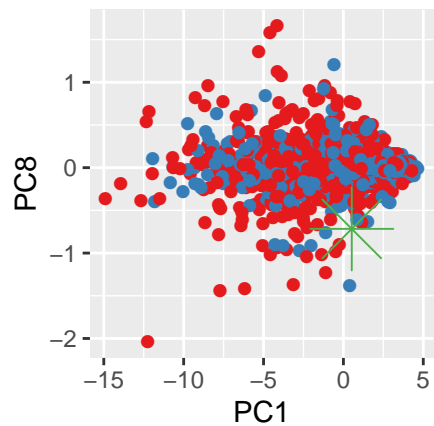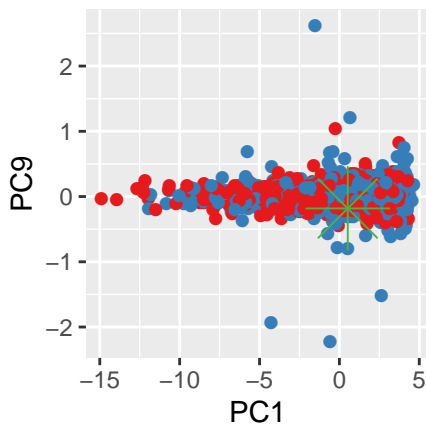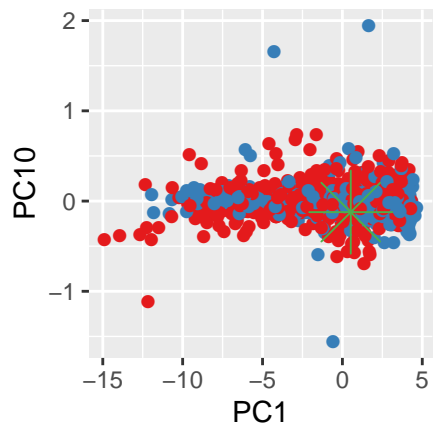

# Chamaeza ruficalda Island

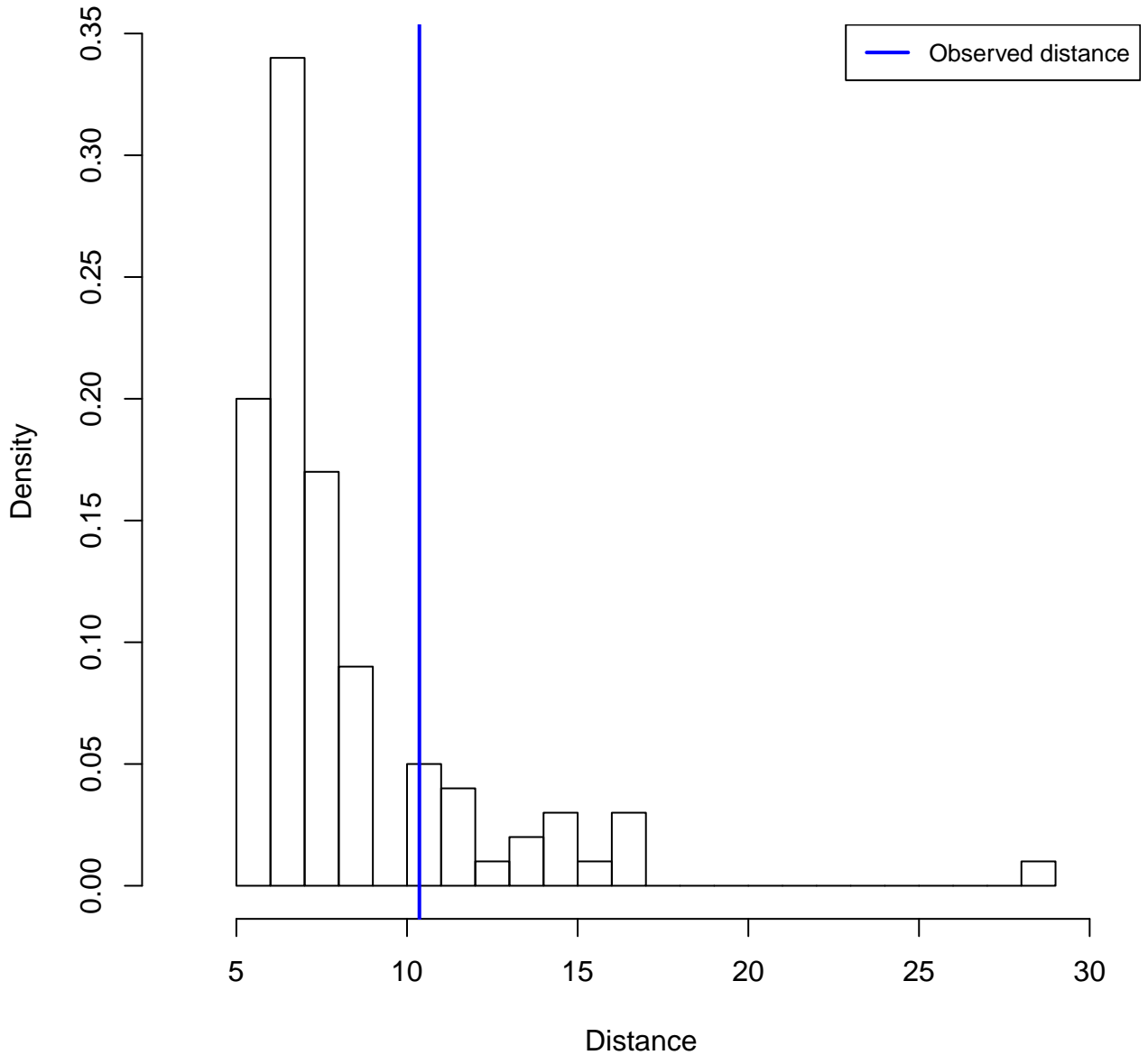

# Chamaeza ruficalda IBD

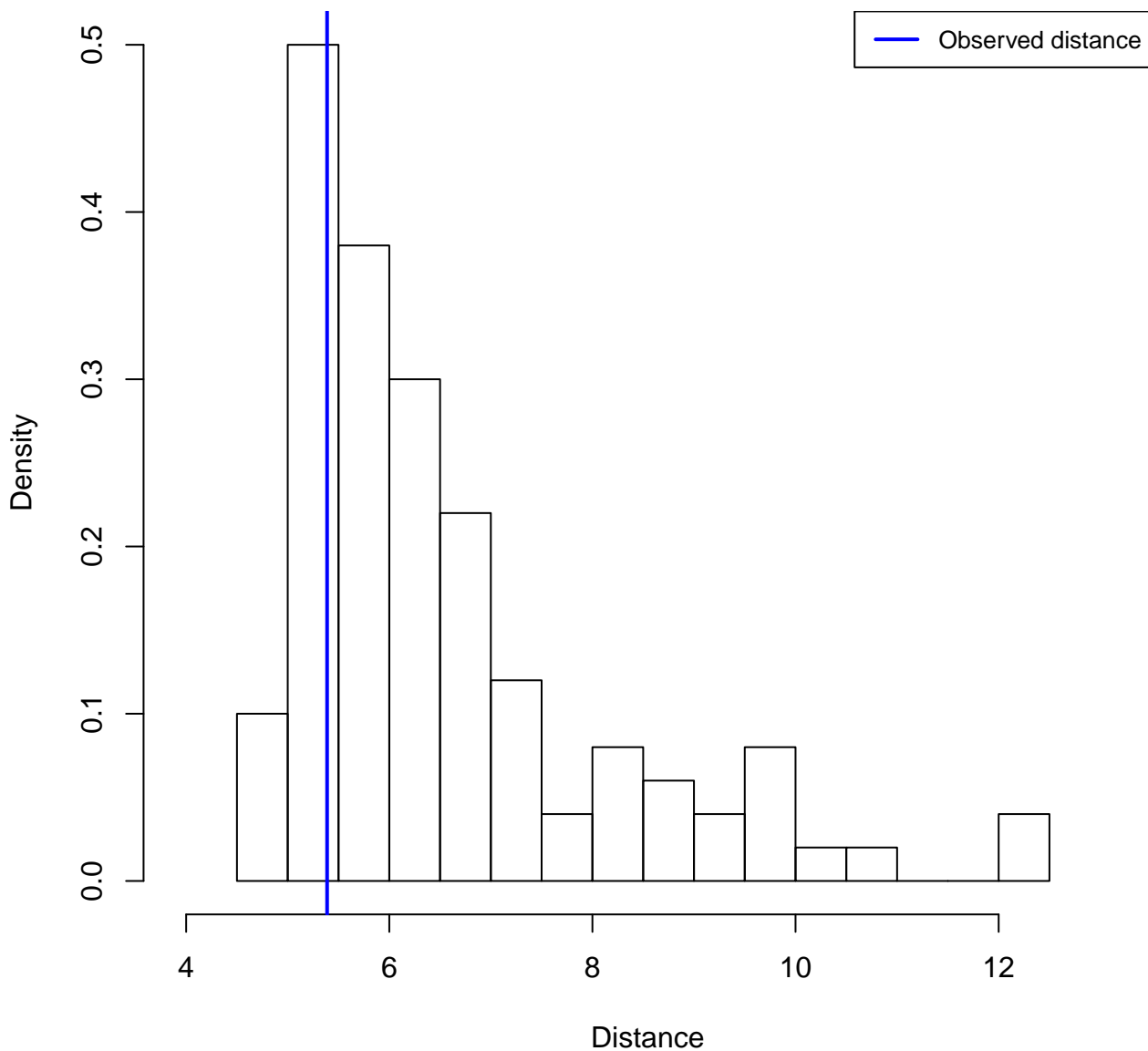

Supplement: Supplementary file 8 — Supplementary Data 5 [file 41467_2021_26537_MOESM8_ESM.gz › PCAs/ruficauda_S_PCA.pdf]

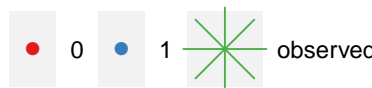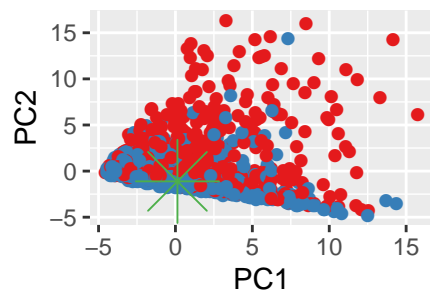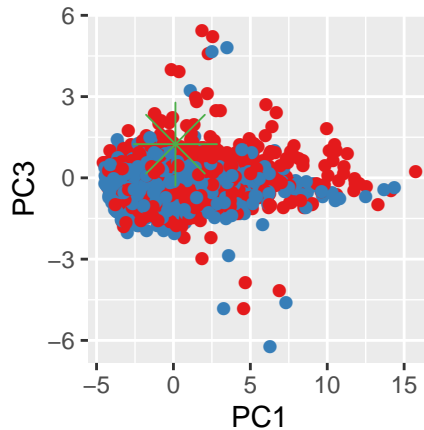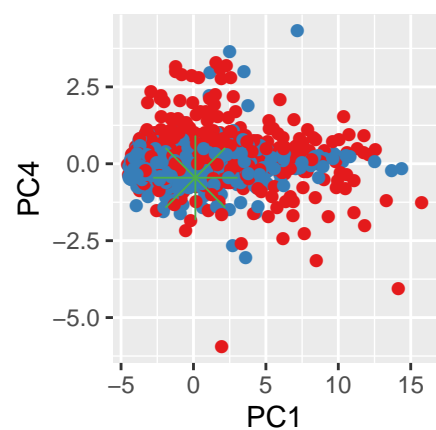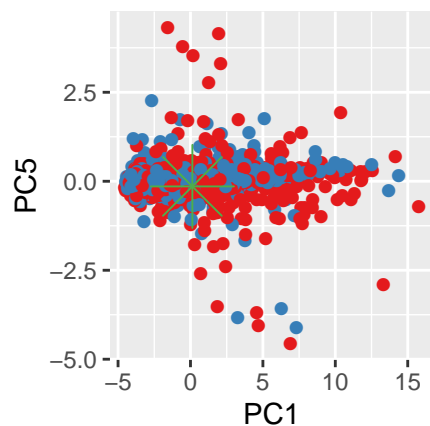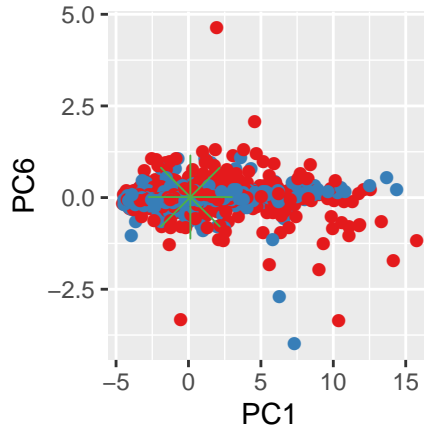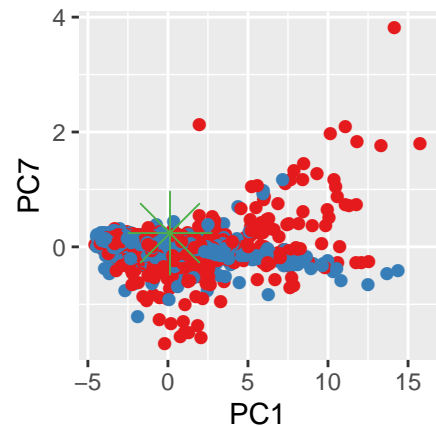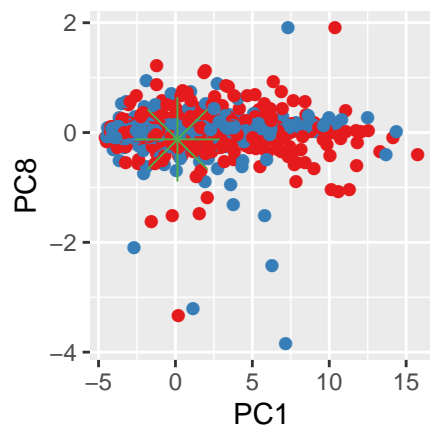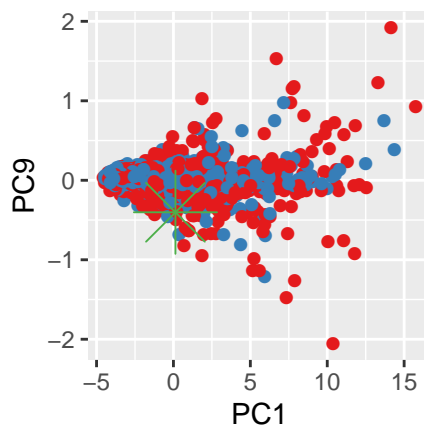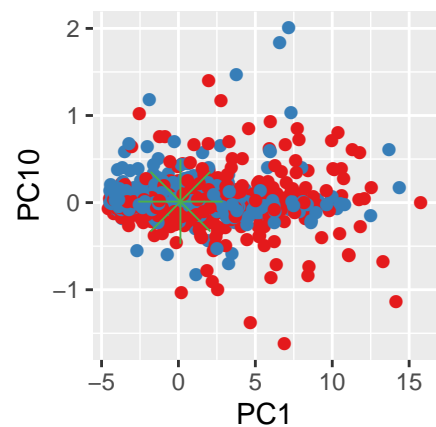

# Syndactyla rufosuperciliata Island

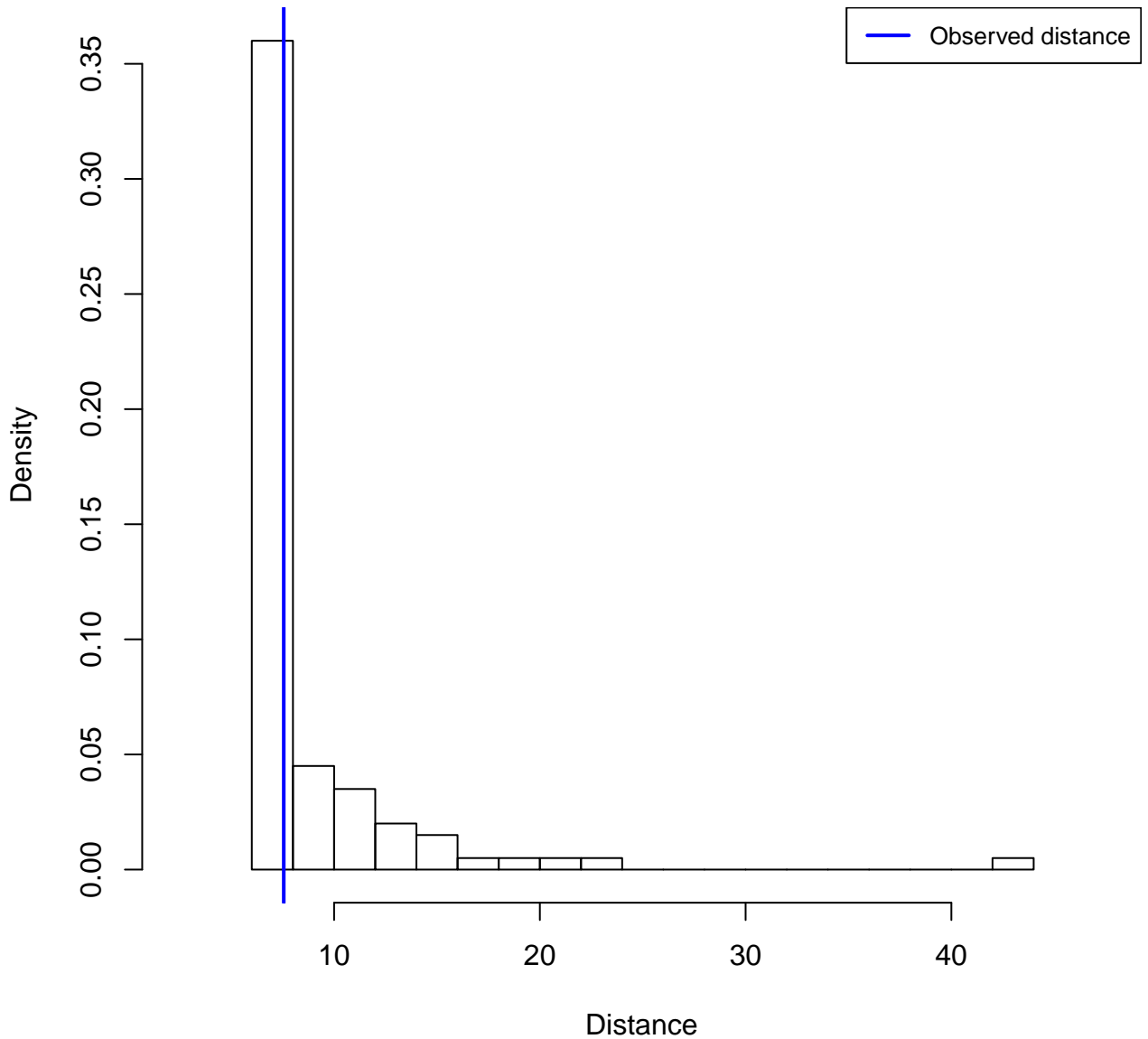

# Syndactyla rufosuperciliata IBD

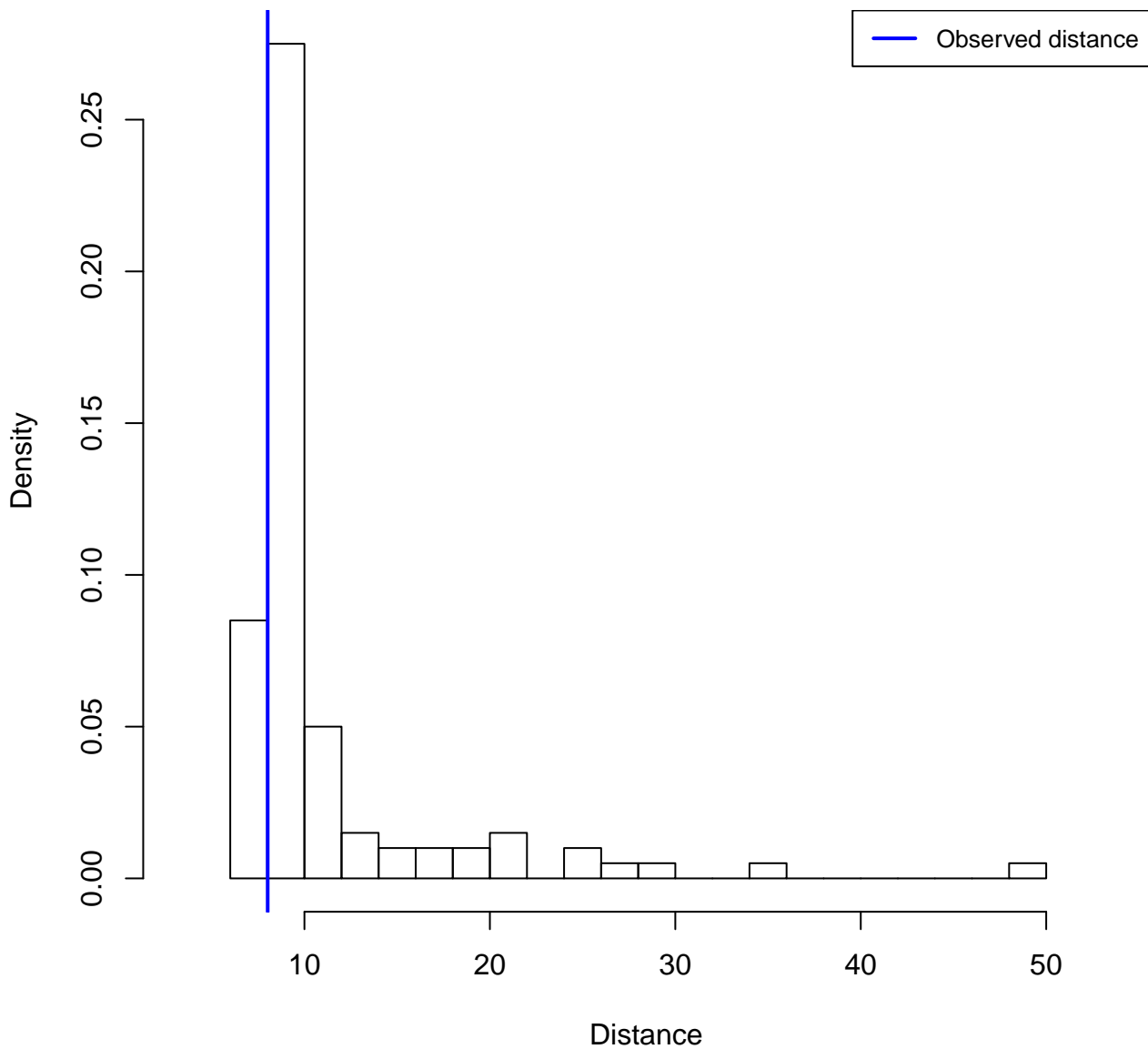

Supplement: Supplementary file 8 — Supplementary Data 5 [file 41467_2021_26537_MOESM8_ESM.gz › PCAs/rufosuperciliata_N_PCA.pdf]

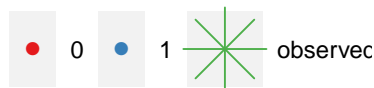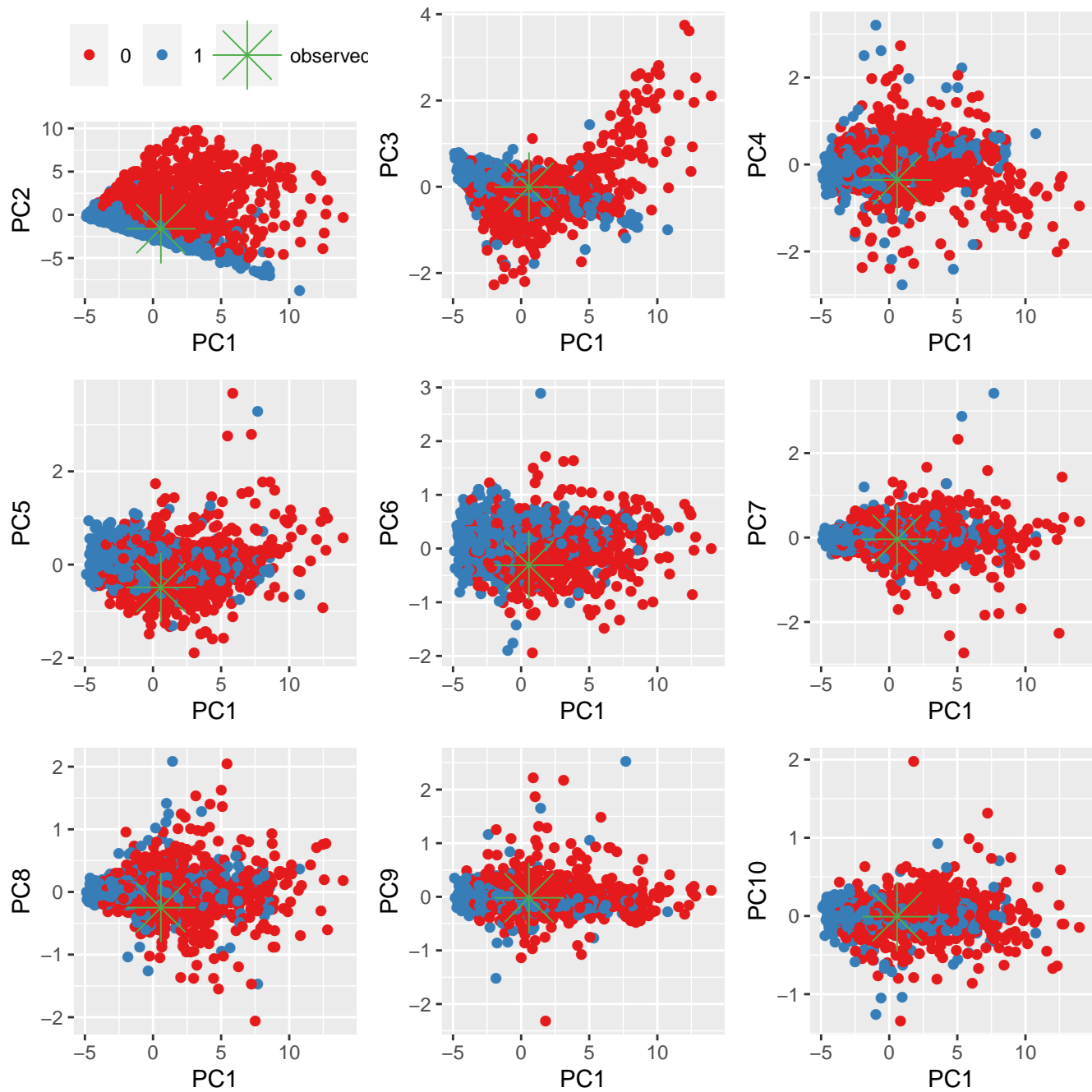

# Stephanophorus diadematus Island

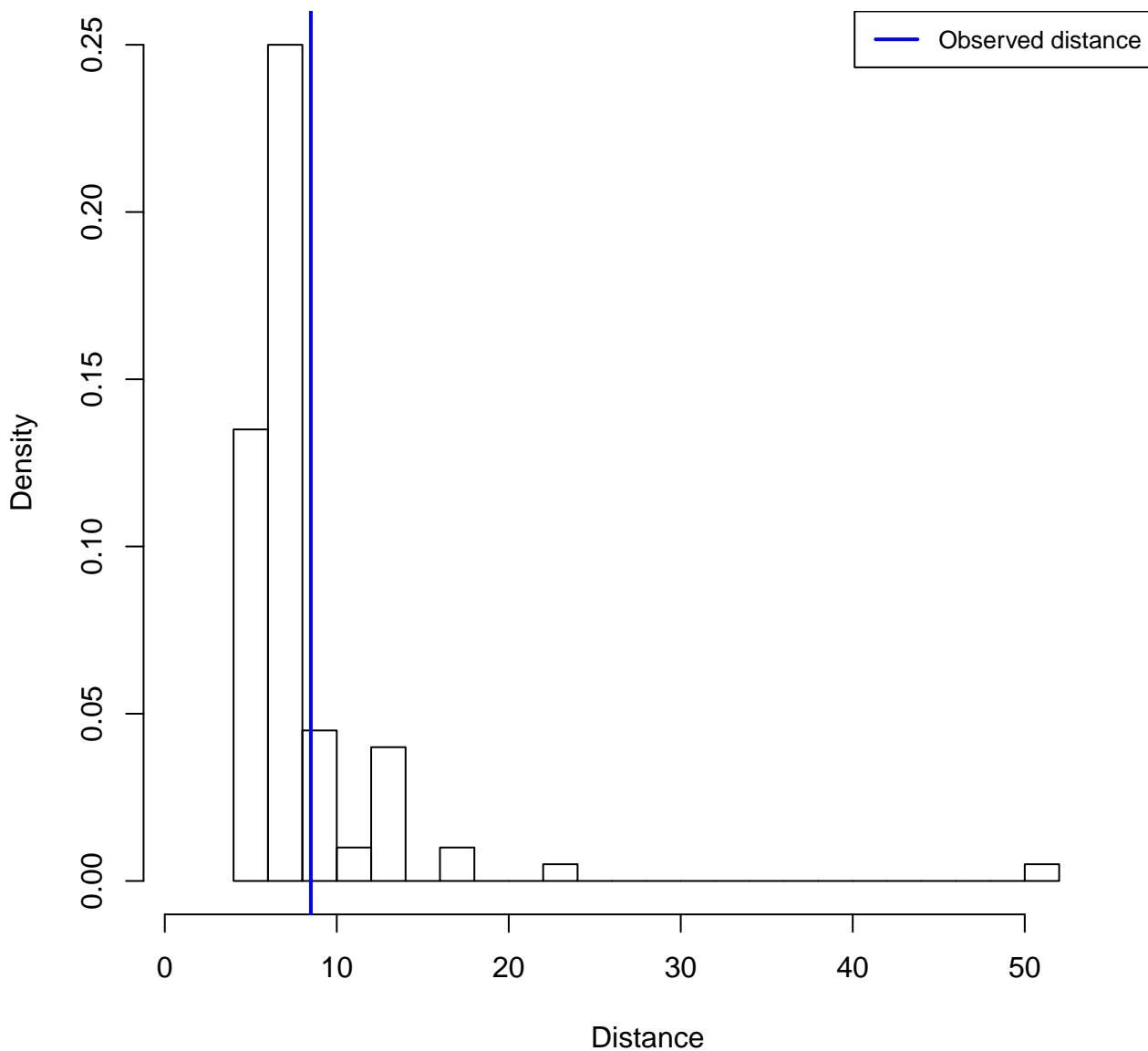

# Stephanophorus diadematus IBD

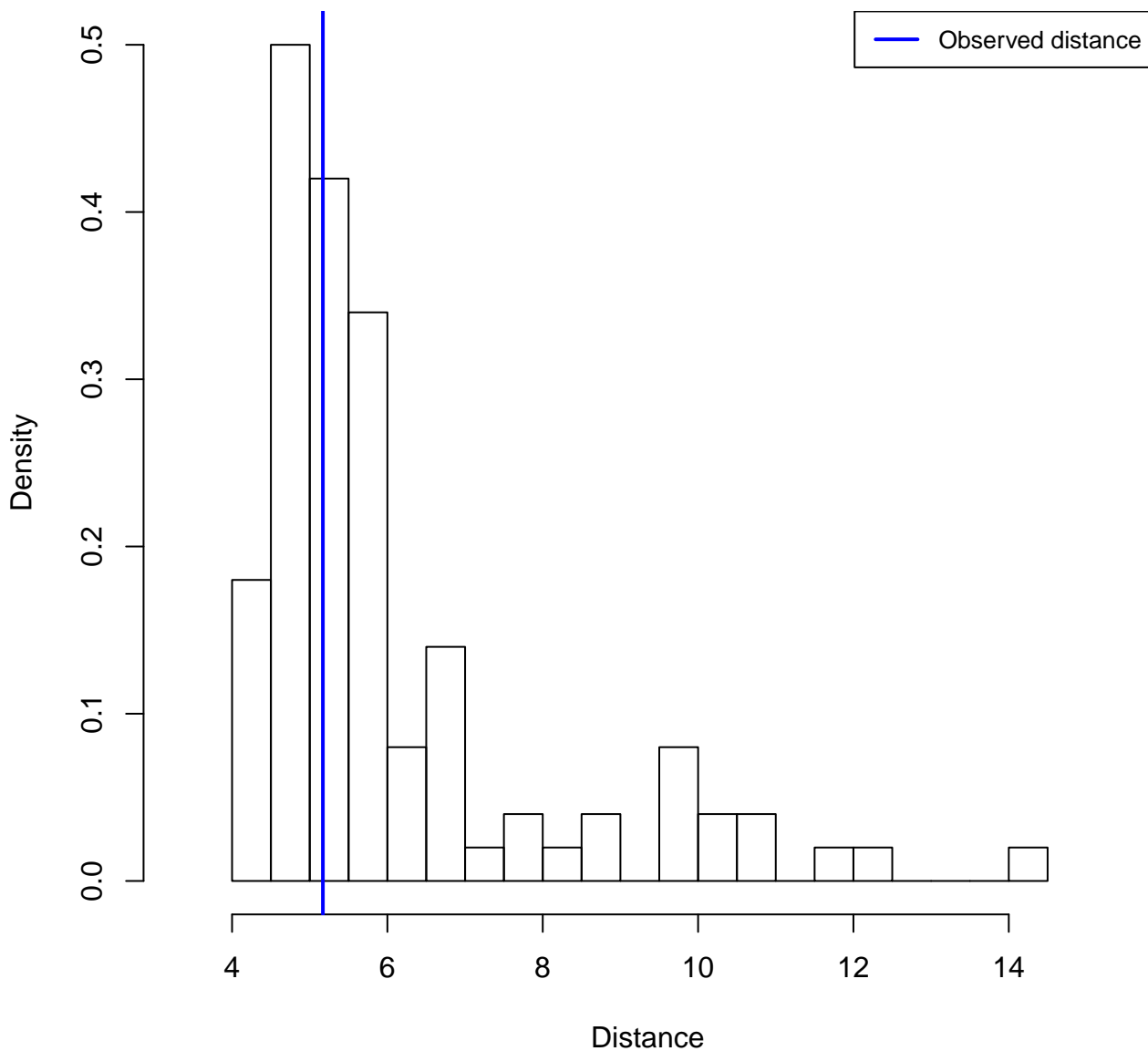

Supplement: Supplementary file 8 — Supplementary Data 5 [file 41467_2021_26537_MOESM8_ESM.gz › PCAs/diadematus_S_PCA.pdf]

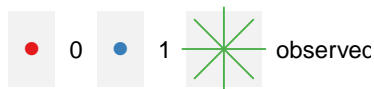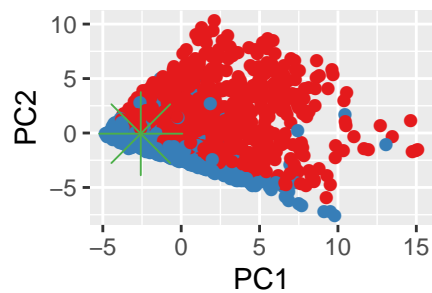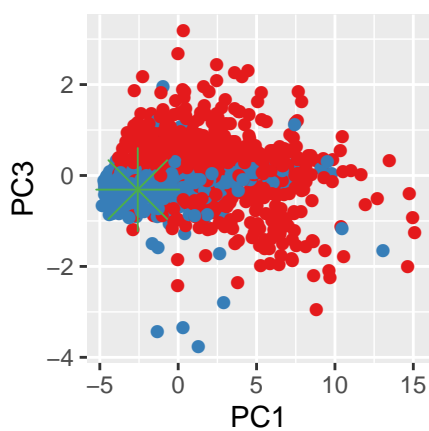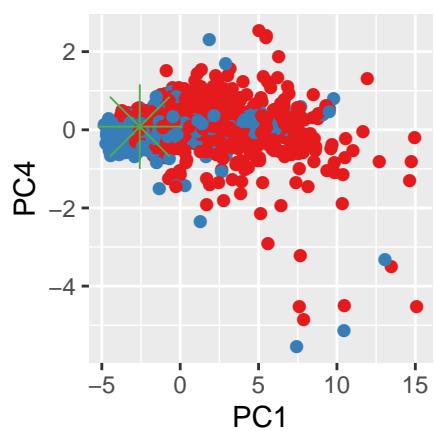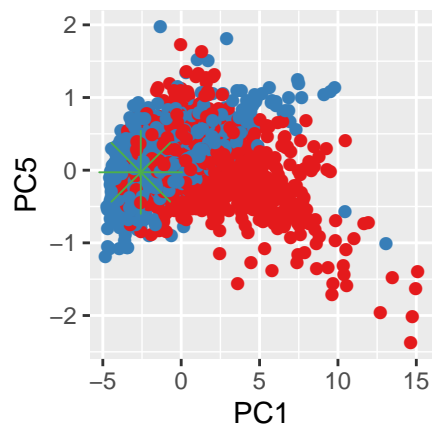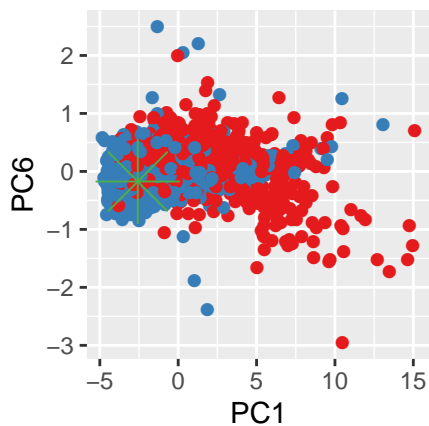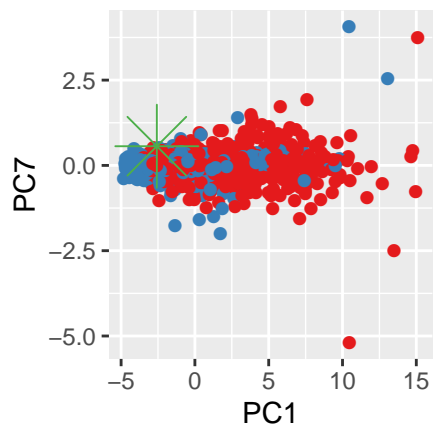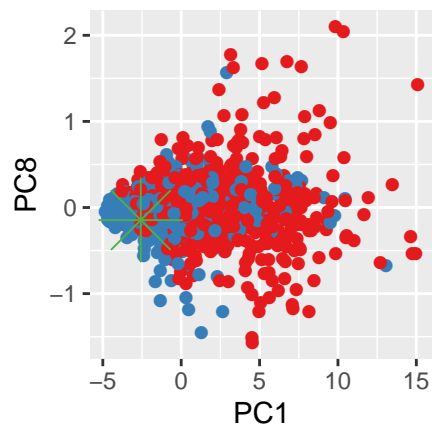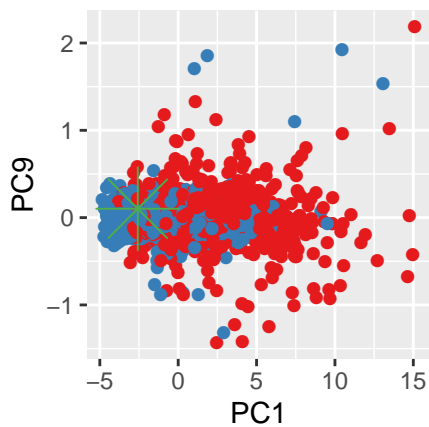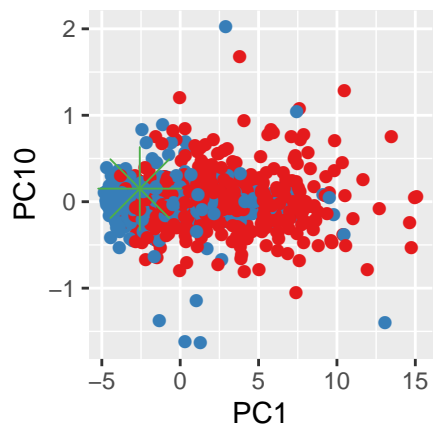

# Cacicus chrysops Island

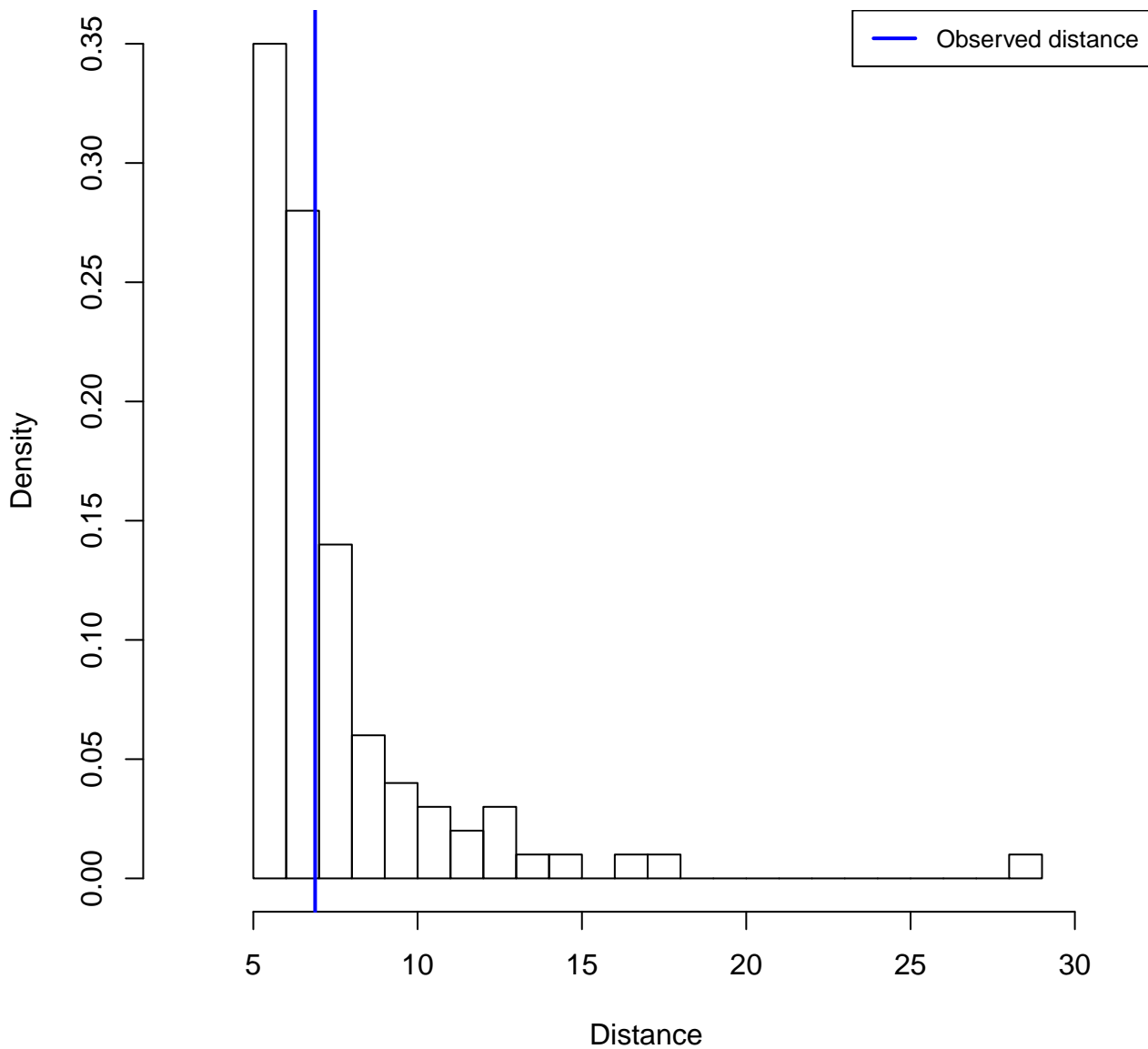

# Cacicus chrysipaterus IBD

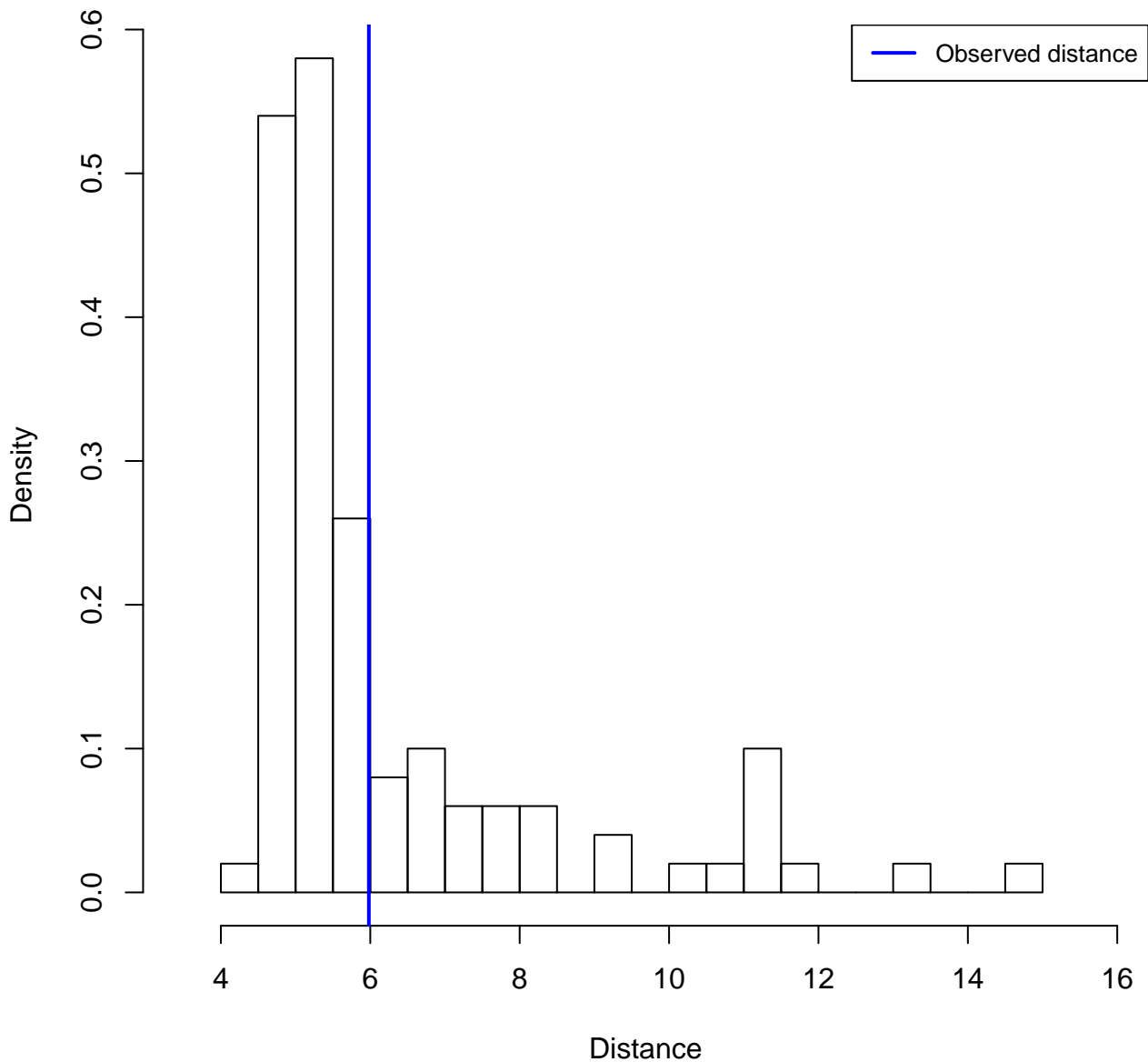

Supplement: Supplementary file 8 — Supplementary Data 5 [file 41467_2021_26537_MOESM8_ESM.gz › PCAs/chrysopterus_S_PCA.pdf]

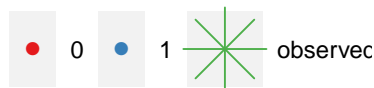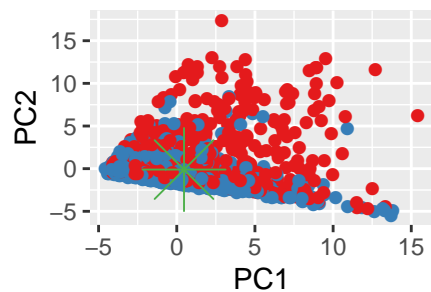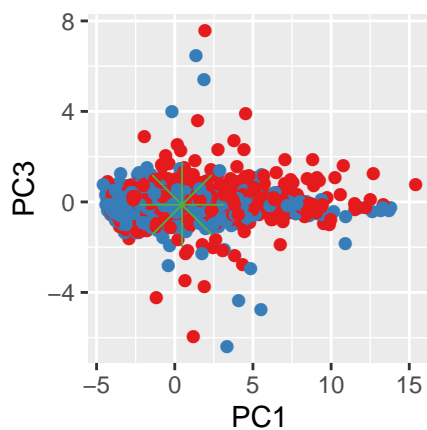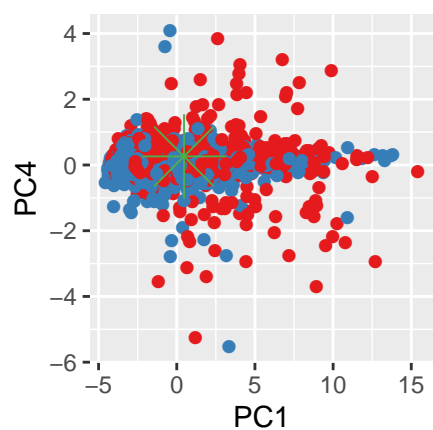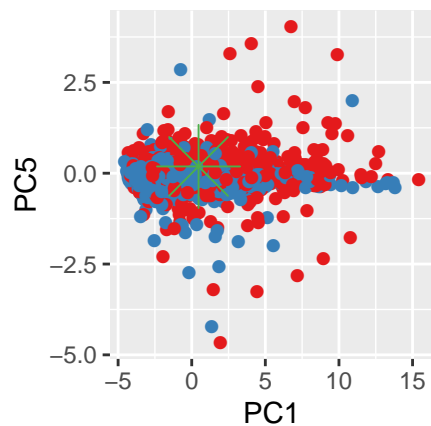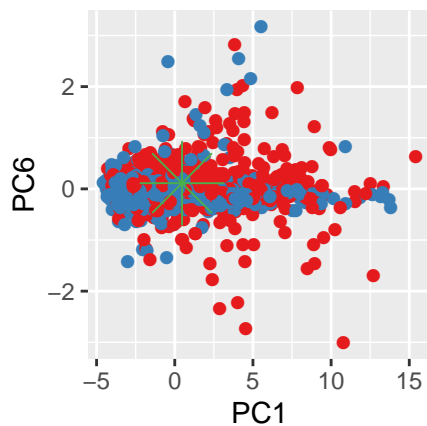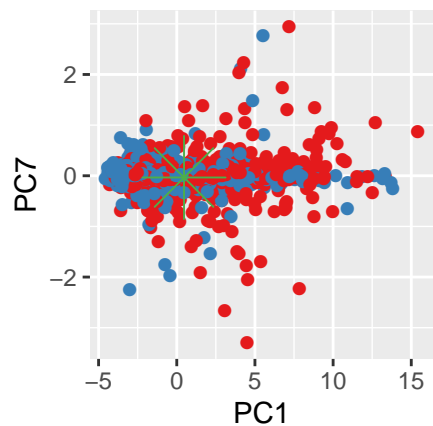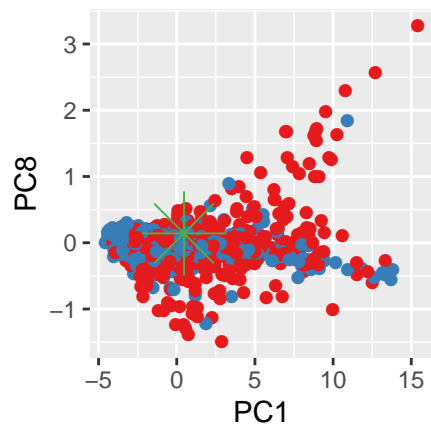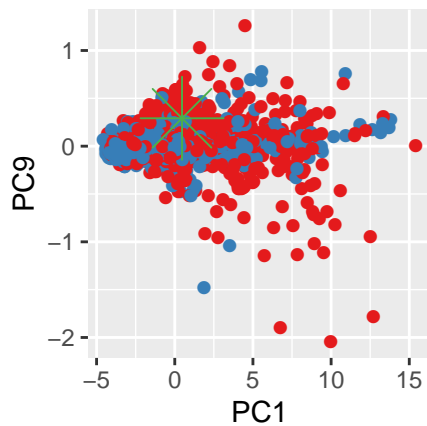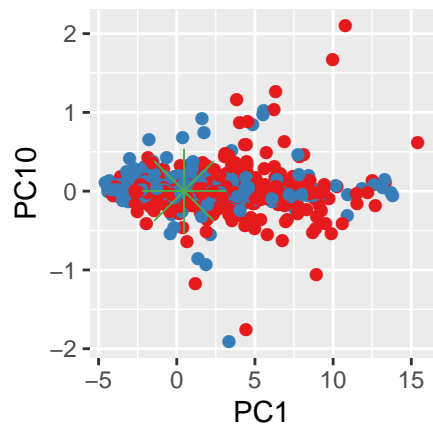

# Hemitriccus obsoletus Island

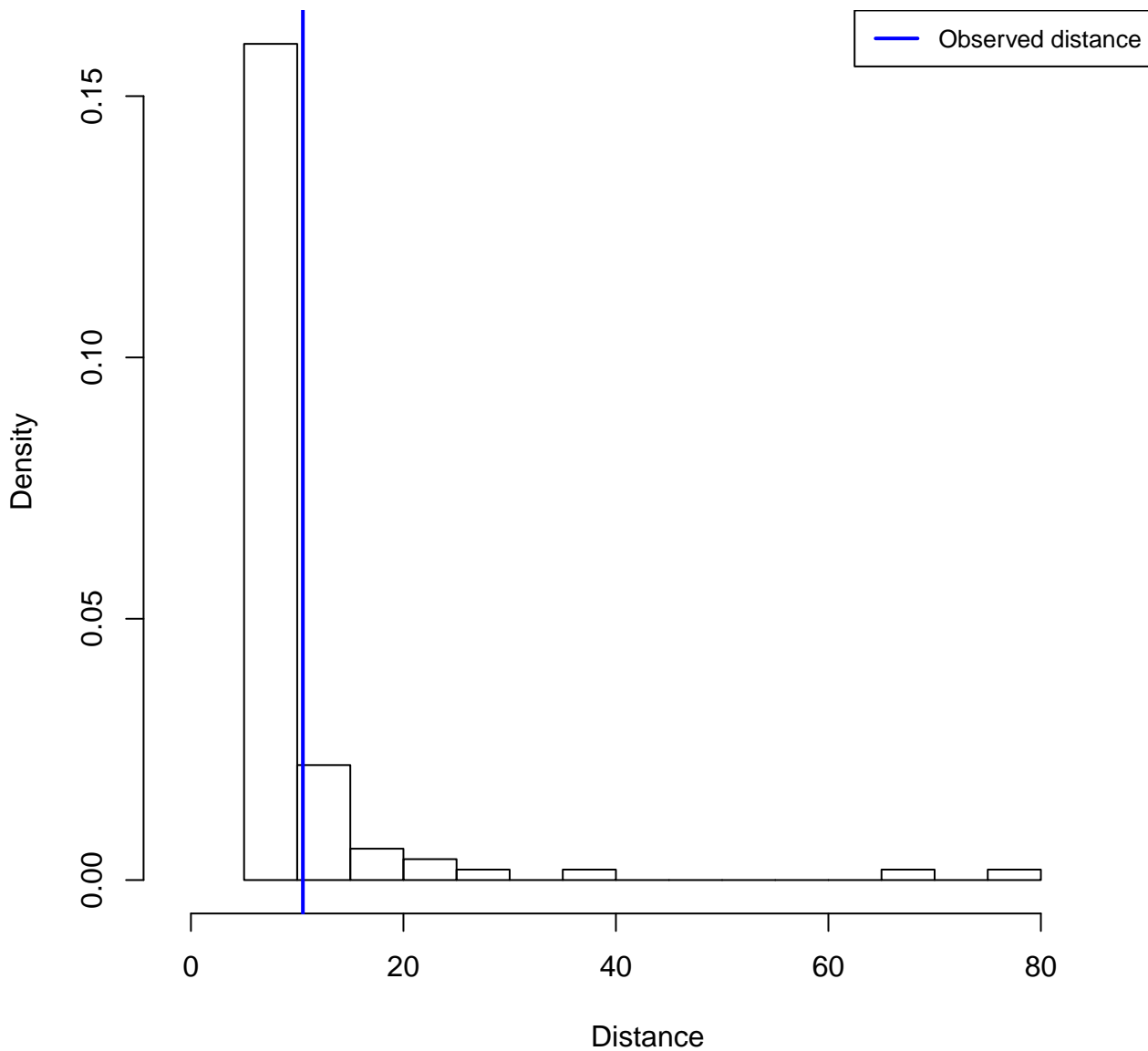

# Hemitriccus obsoletus IBD

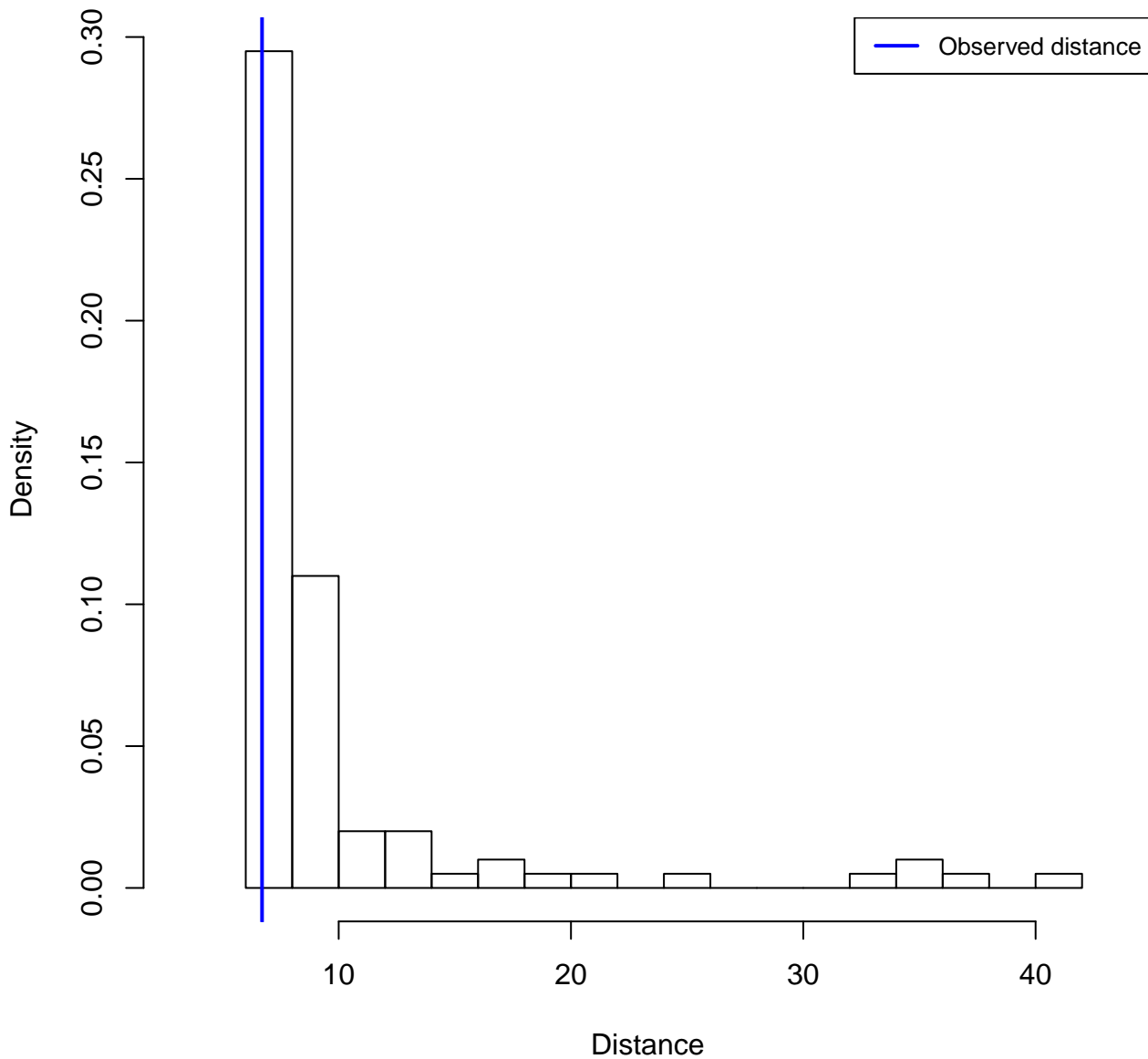

Supplement: Supplementary file 8 — Supplementary Data 5 [file 41467_2021_26537_MOESM8_ESM.gz › PCAs/obsoletus_N_PCA.pdf]

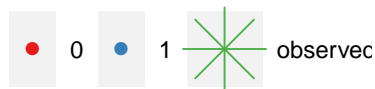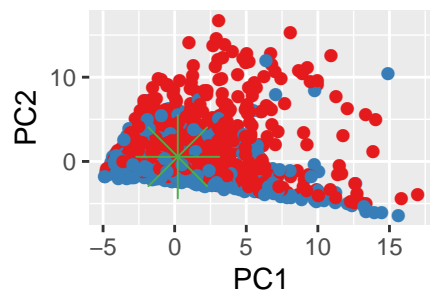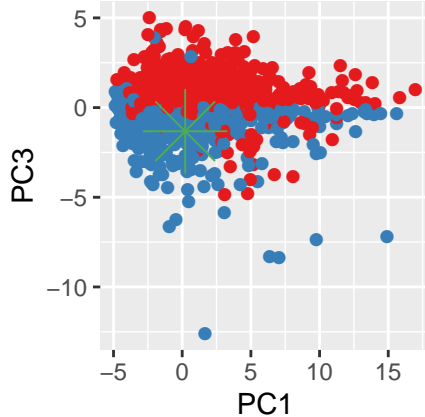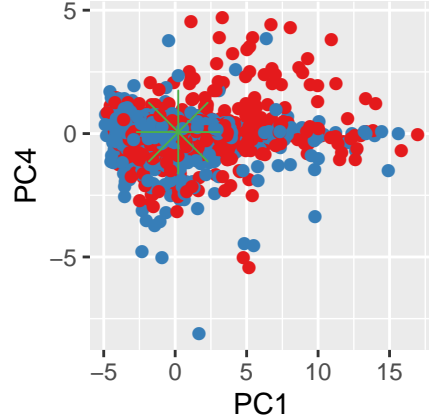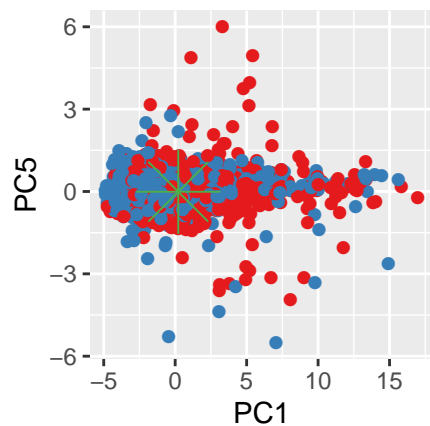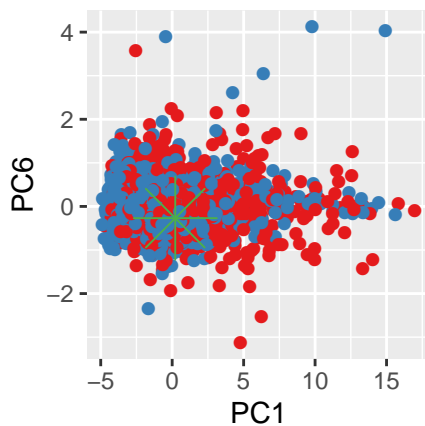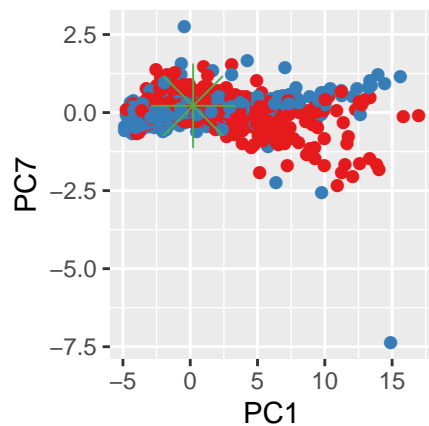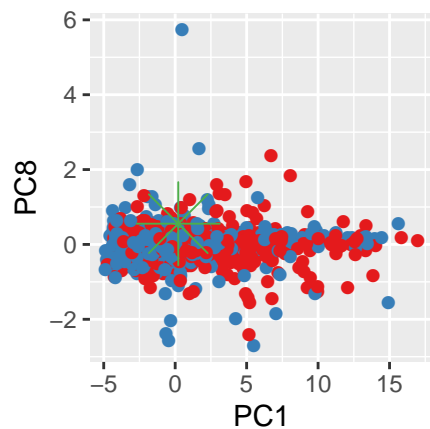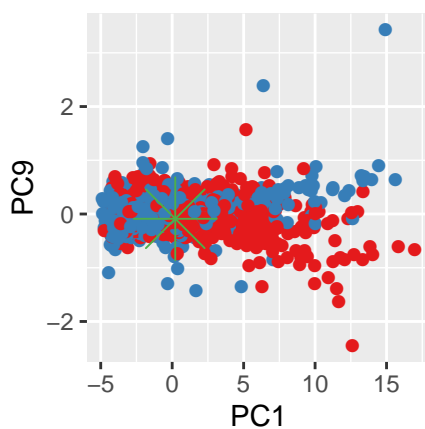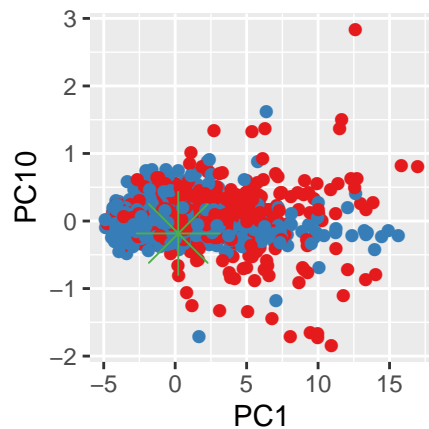

# Chamaeza ruficalda Island

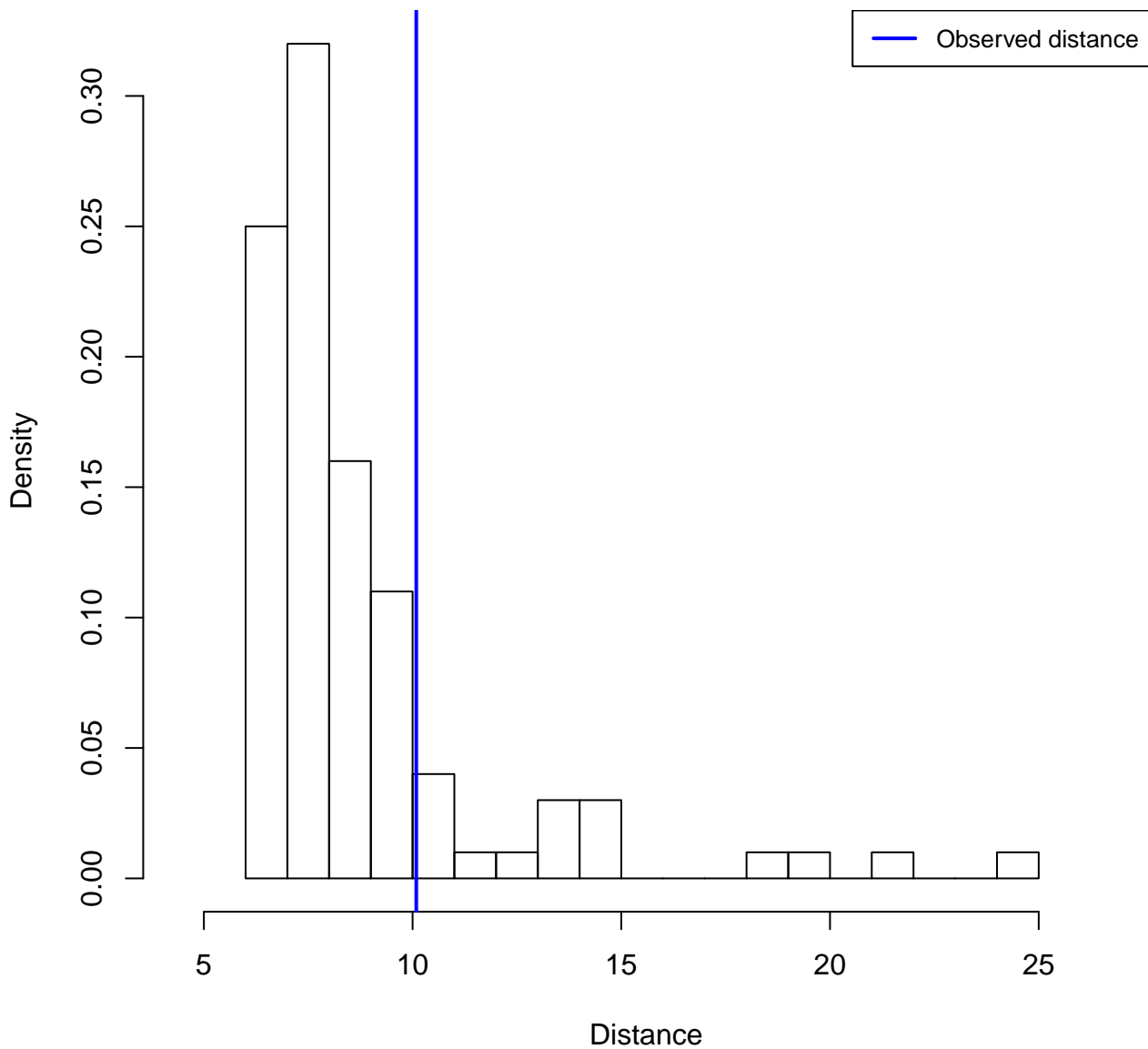

# Chamaeza ruficalda IBD

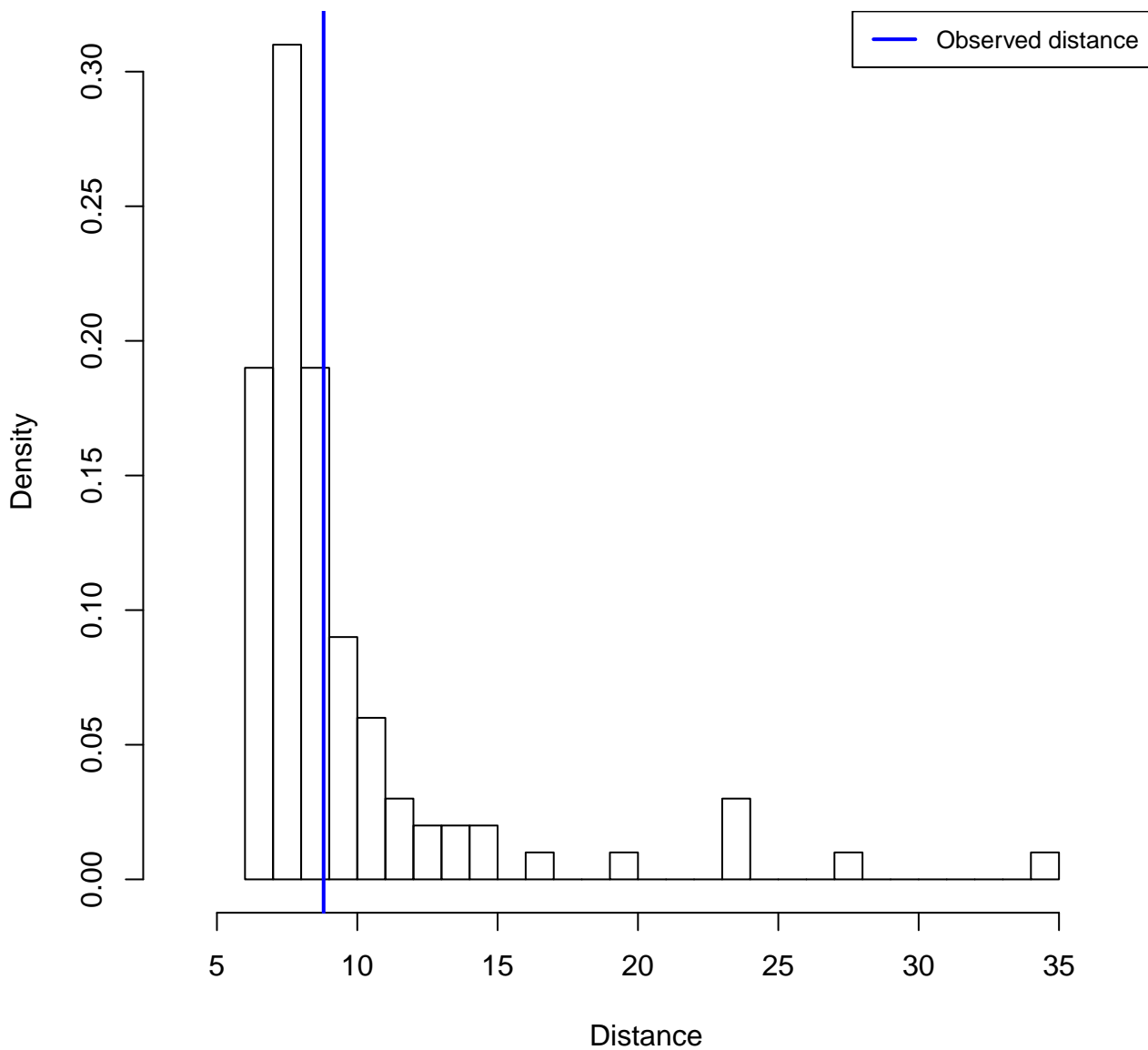

Supplement: Supplementary file 8 — Supplementary Data 5 [file 41467_2021_26537_MOESM8_ESM.gz › PCAs/ruficauda_N_PCA.pdf]

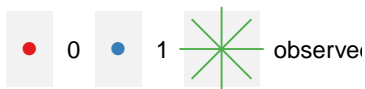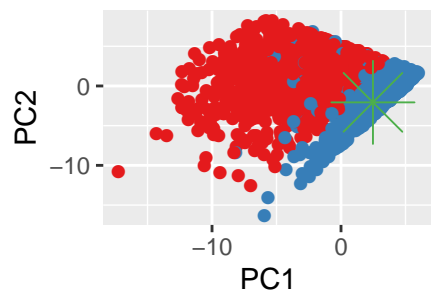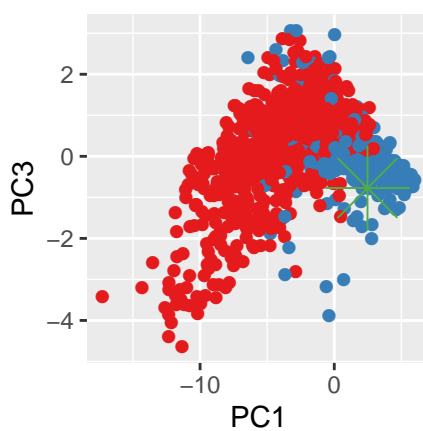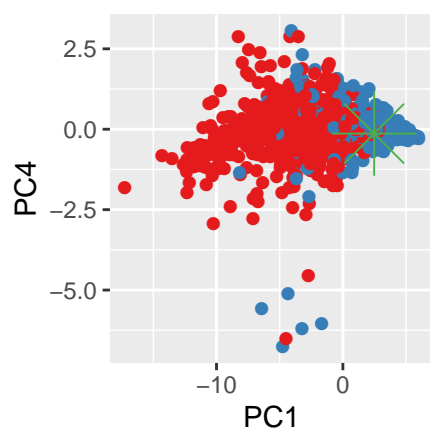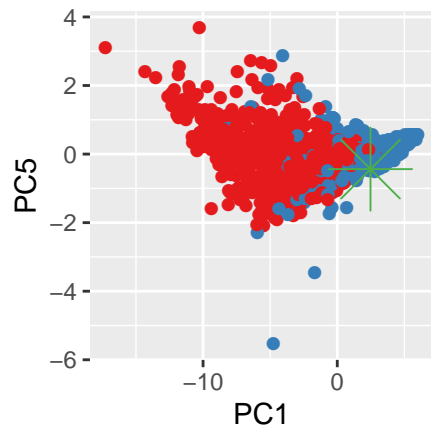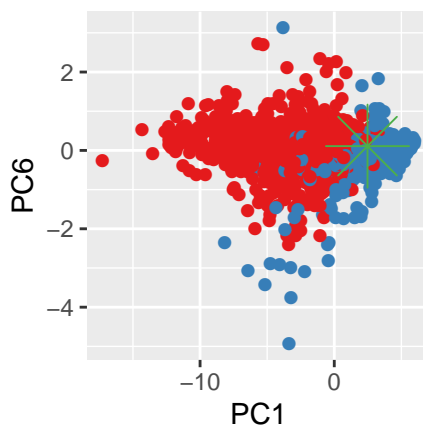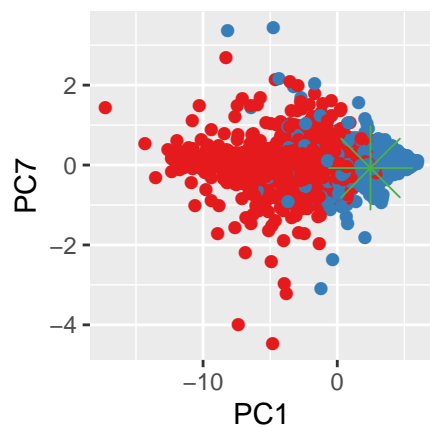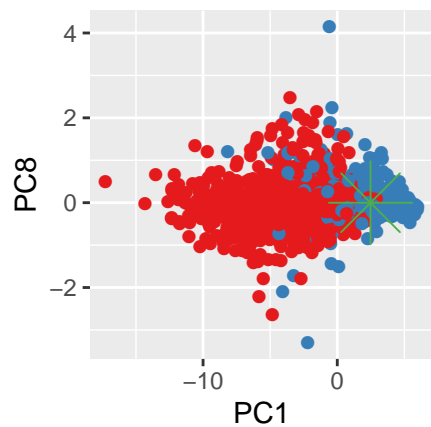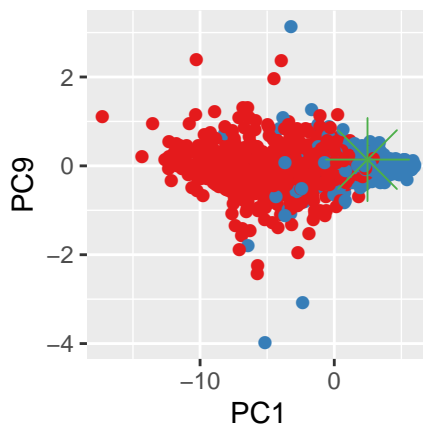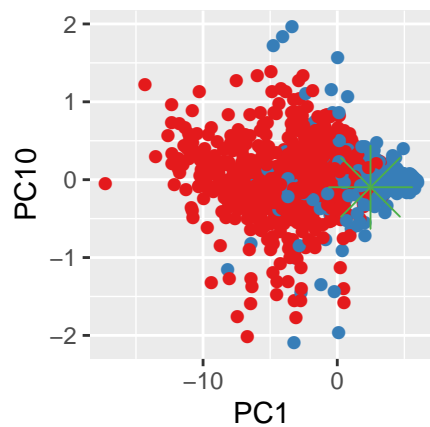

# Microspingus lateralis Island

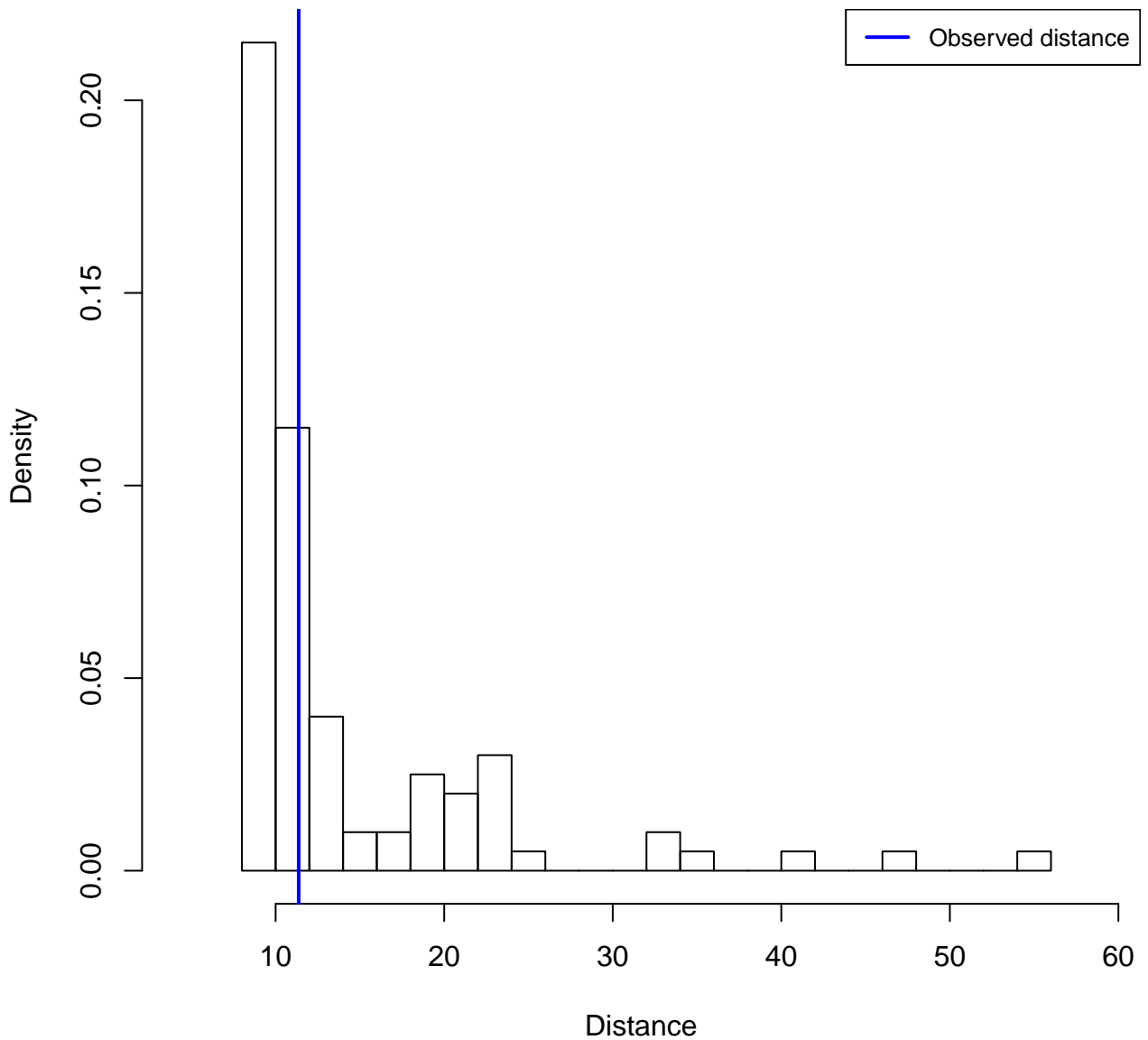

**Microspingus lateralis IBD**

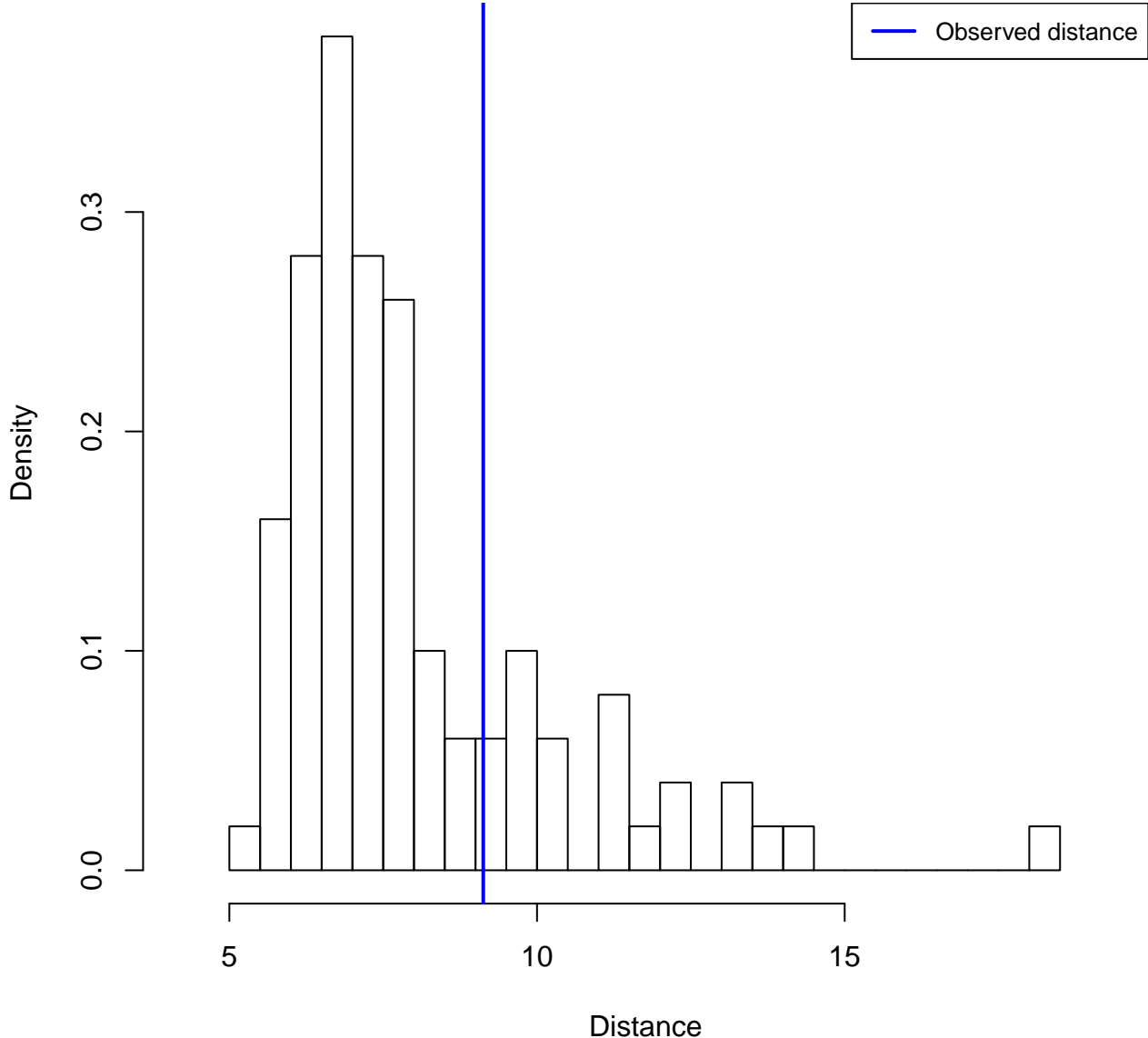

Supplement: Supplementary file 8 — Supplementary Data 5 [file 41467_2021_26537_MOESM8_ESM.gz › PCAs/lateralis_S_PCA.pdf]

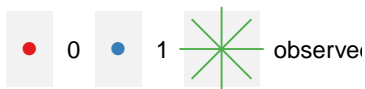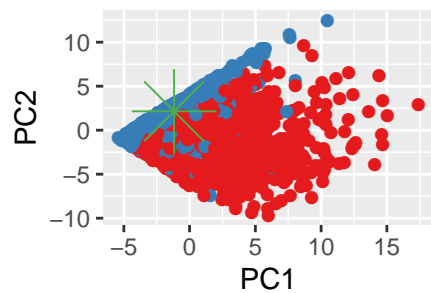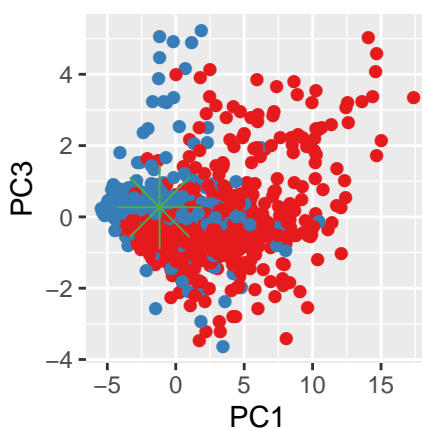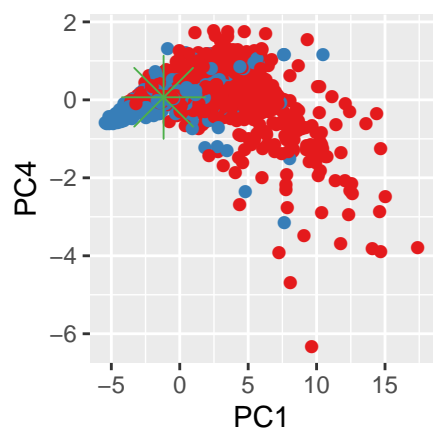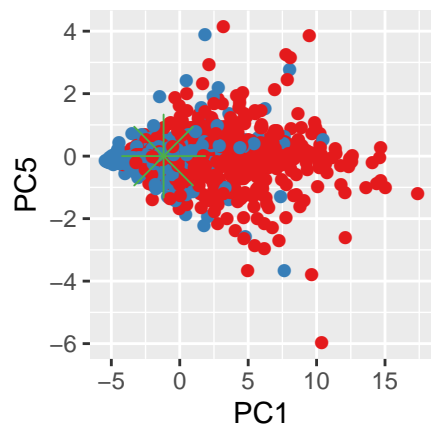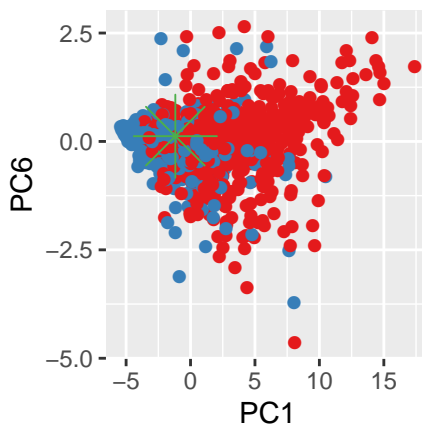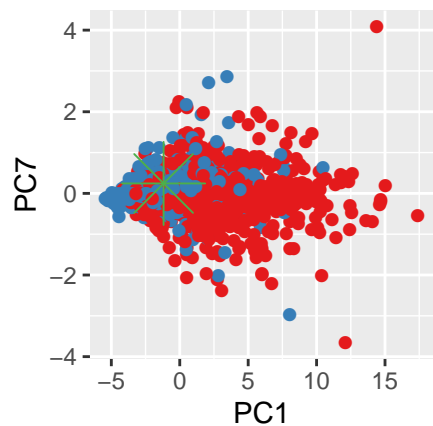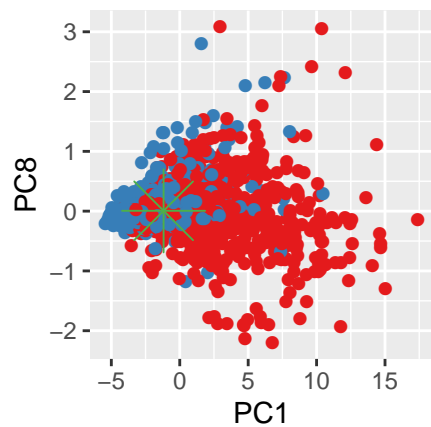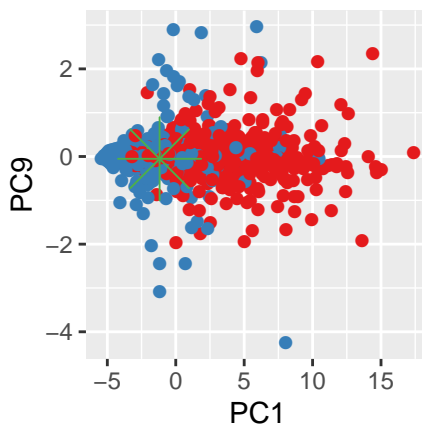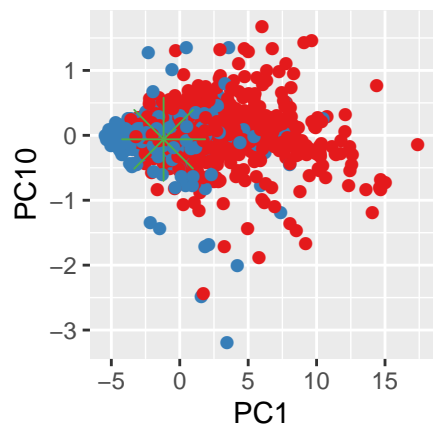

# Stephanoxix lalandi Island

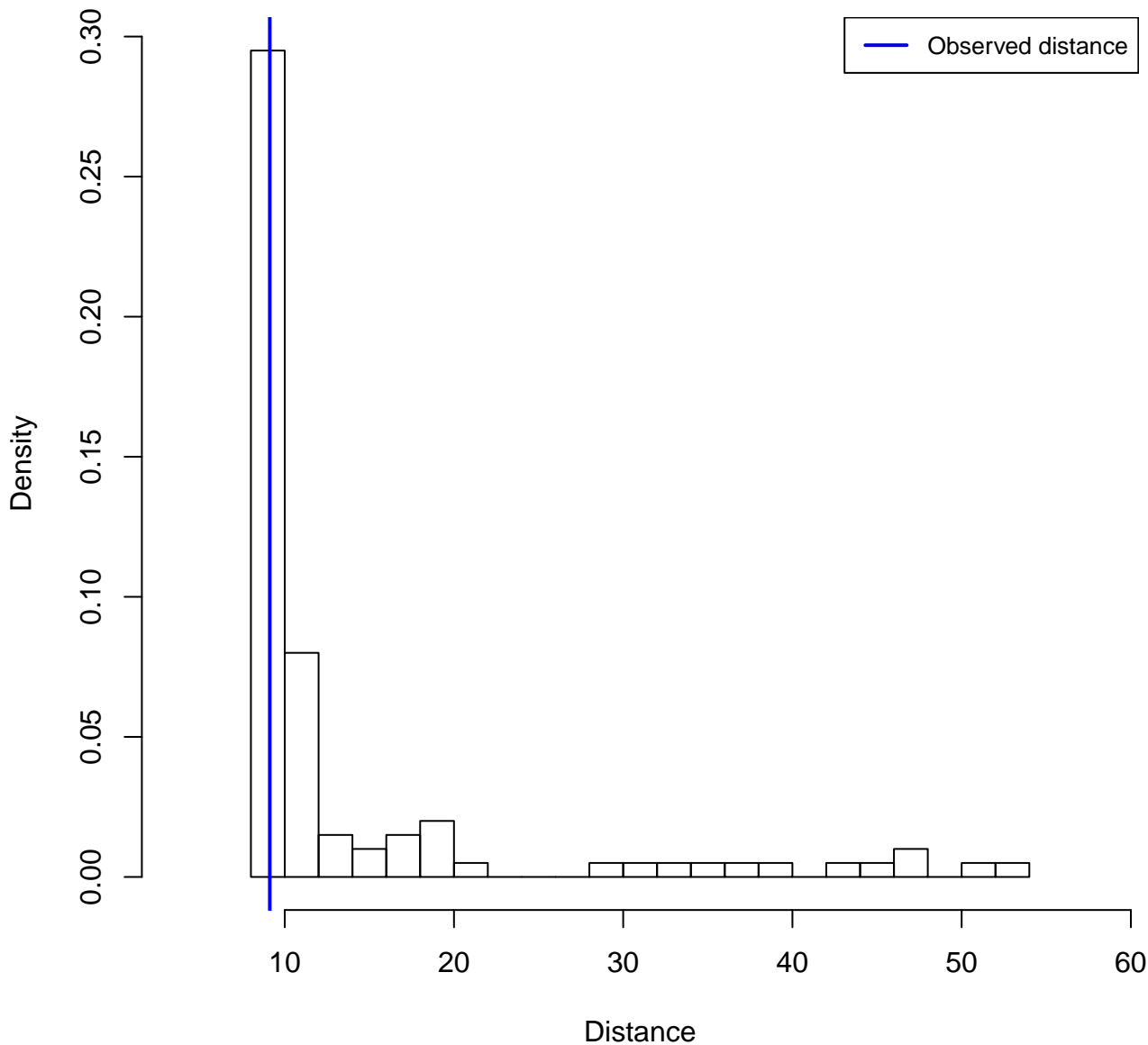

# Stephanoxix lalandi IBD

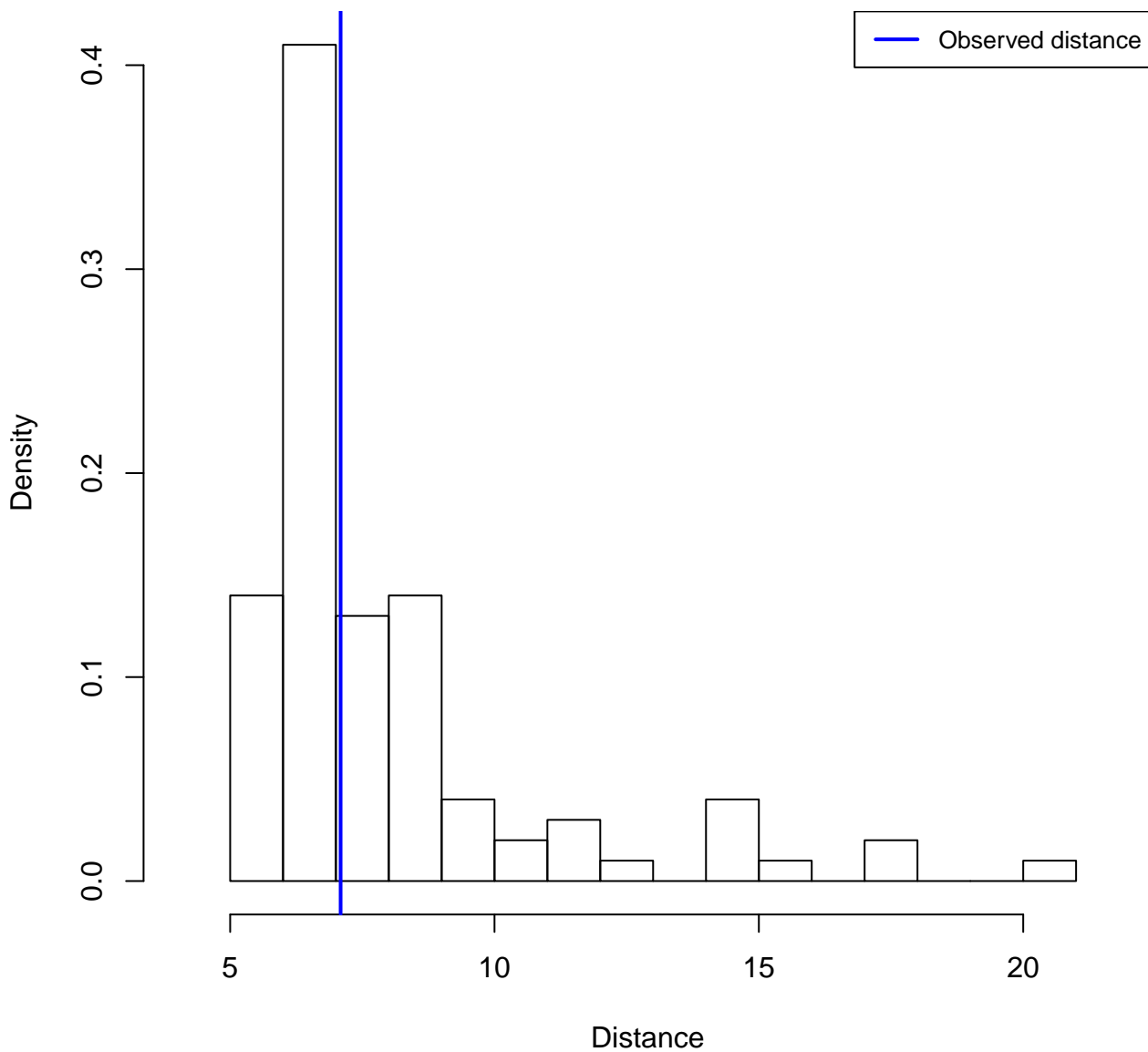

Supplement: Supplementary file 8 — Supplementary Data 5 [file 41467_2021_26537_MOESM8_ESM.gz › PCAs/lalandi_S_PCA.pdf]

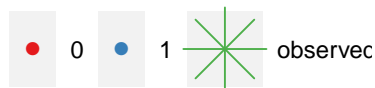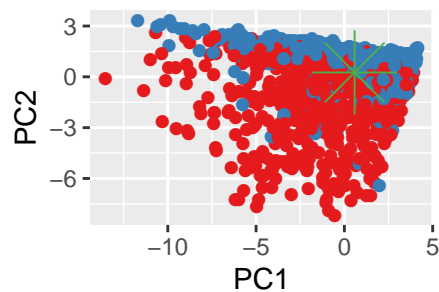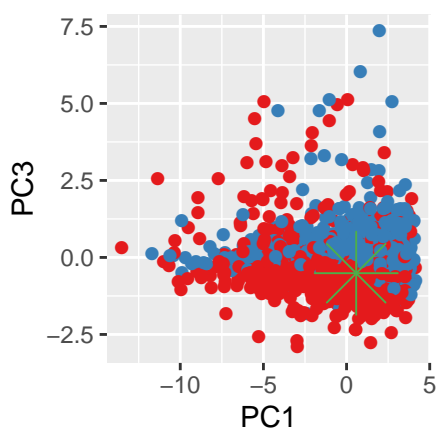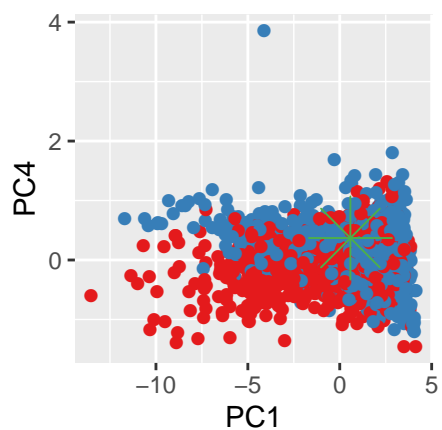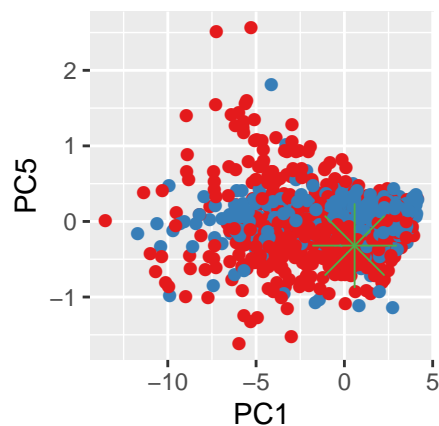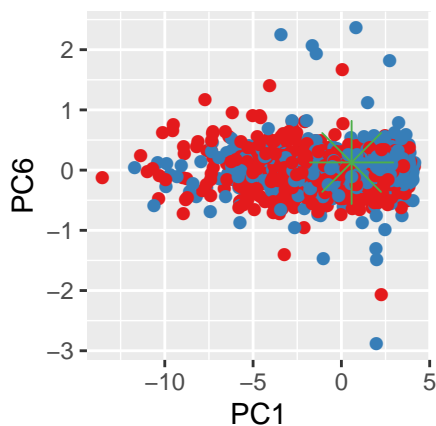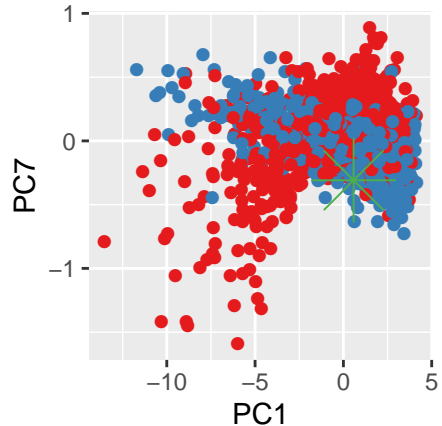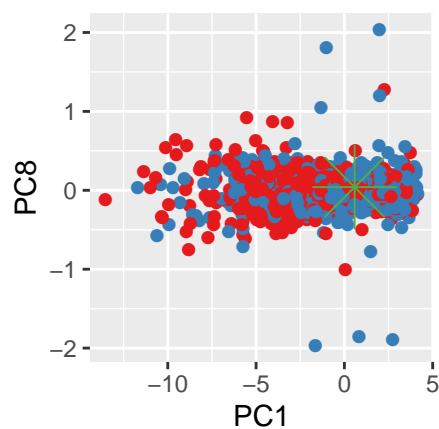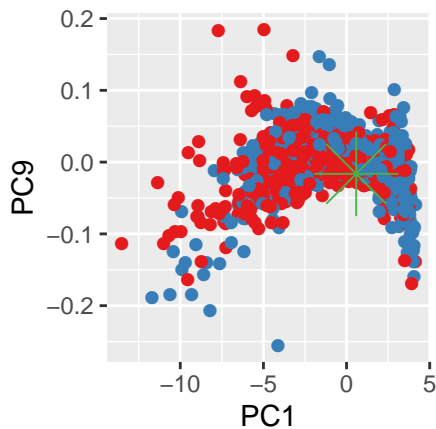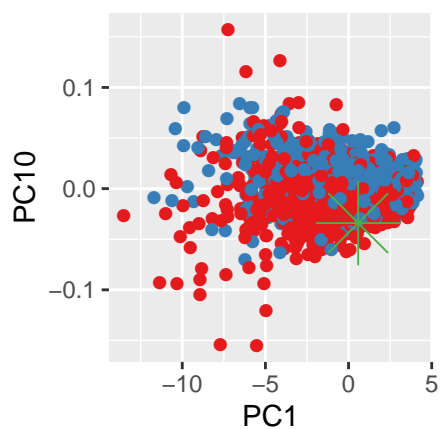

# Dysithamnus xanthopterus Island

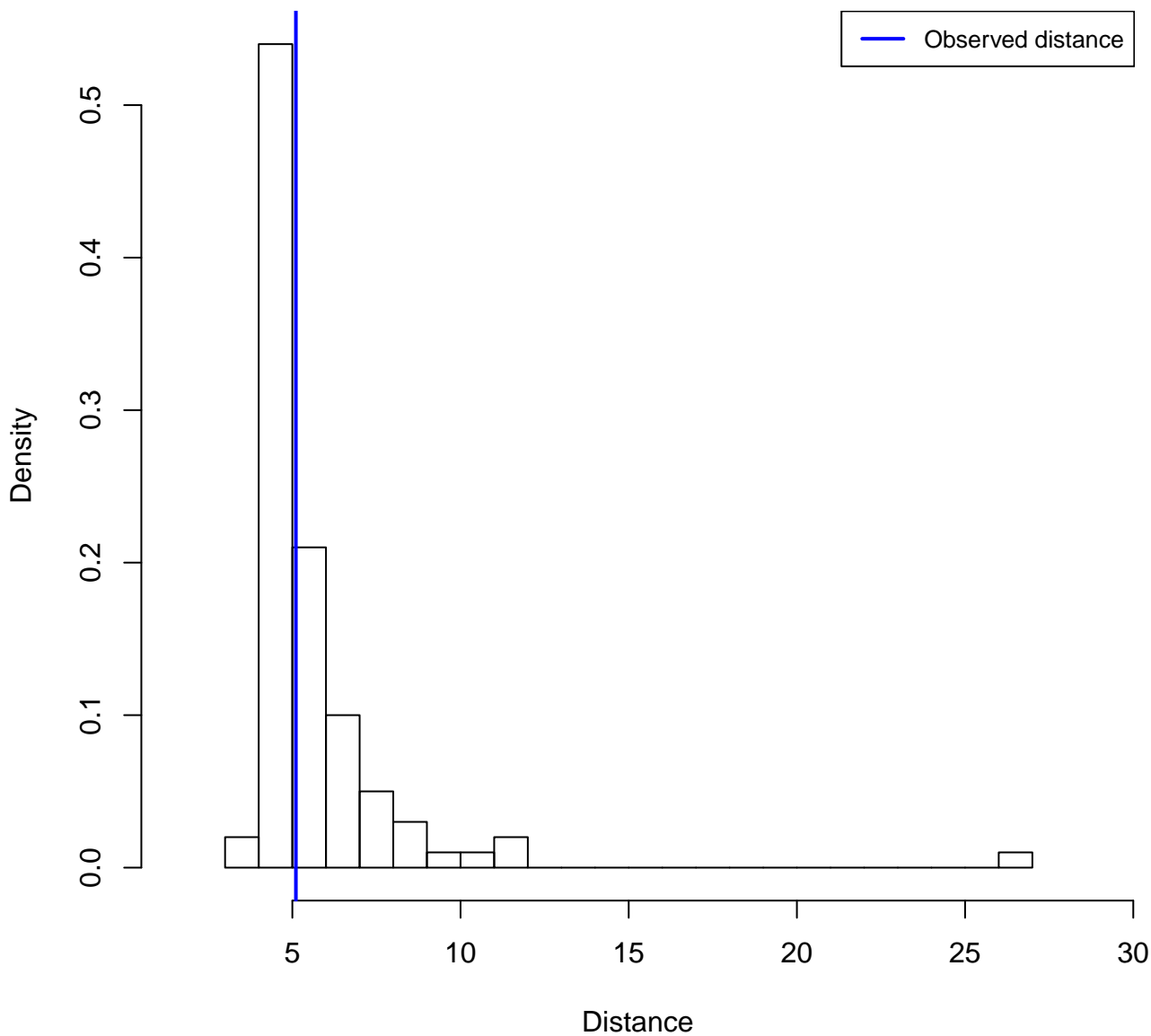

# Dysithamnus xanthopterus IBD

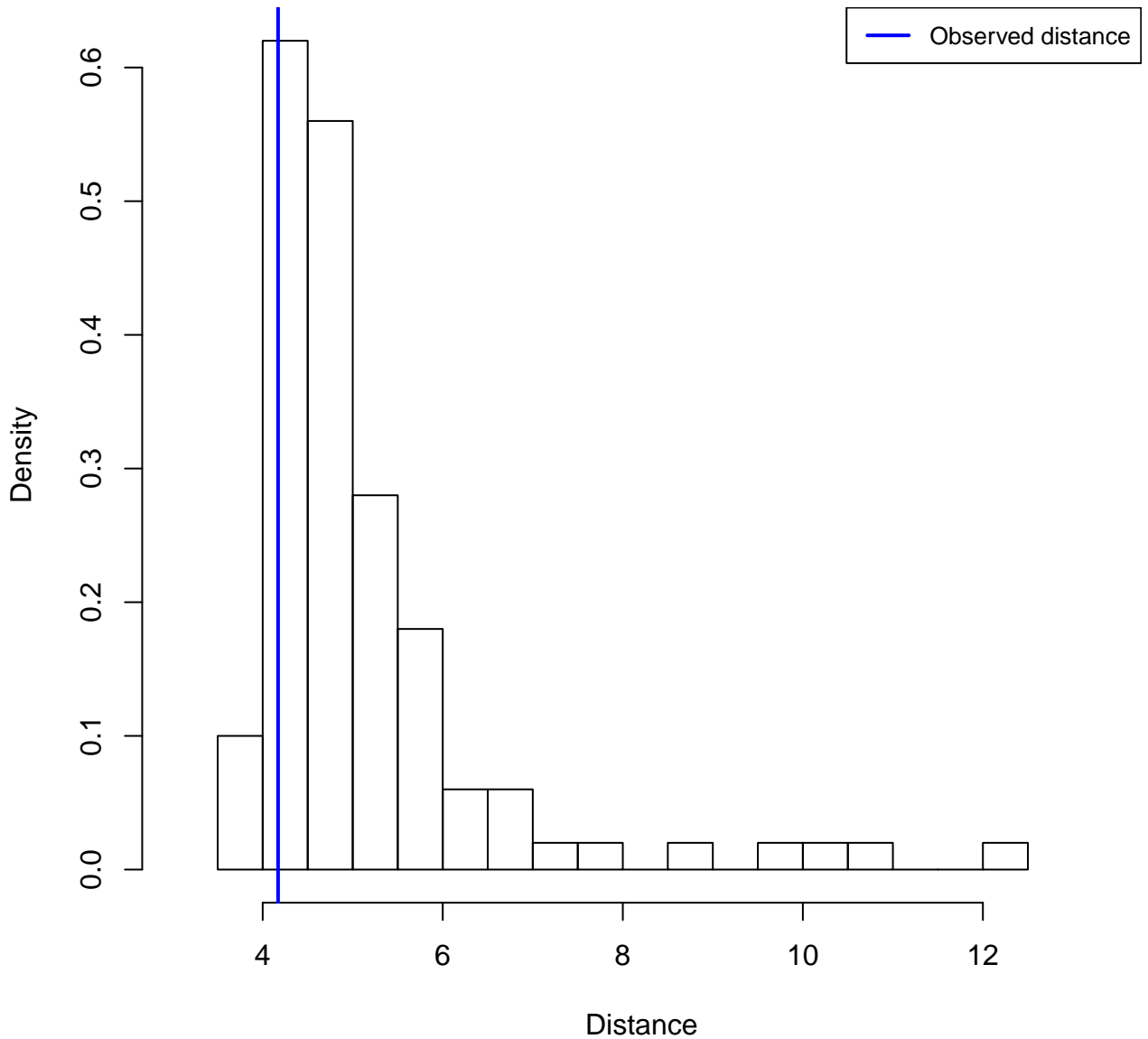

Supplement: Supplementary file 8 — Supplementary Data 5 [file 41467_2021_26537_MOESM8_ESM.gz › PCAs/xanthopterus_S_PCA.pdf]

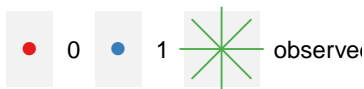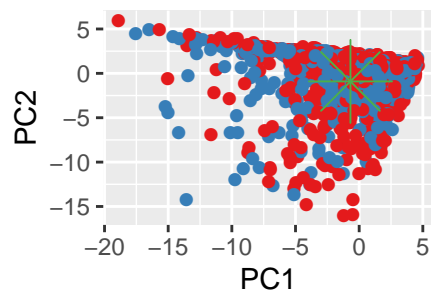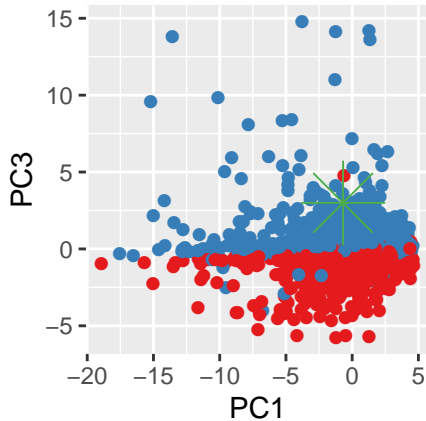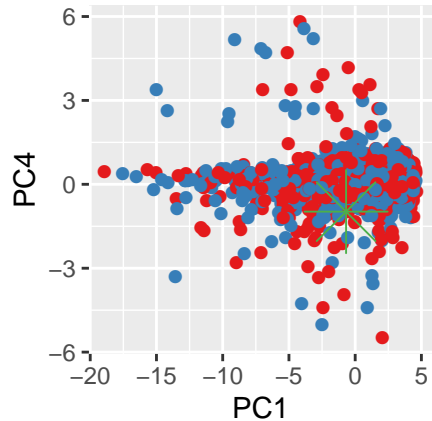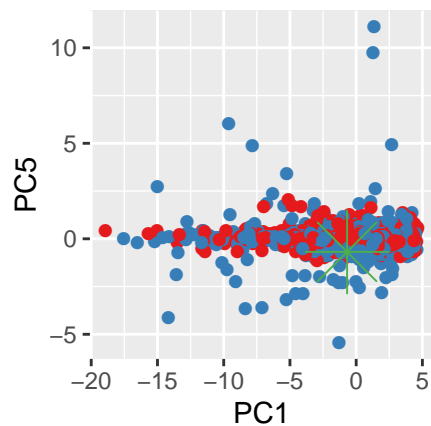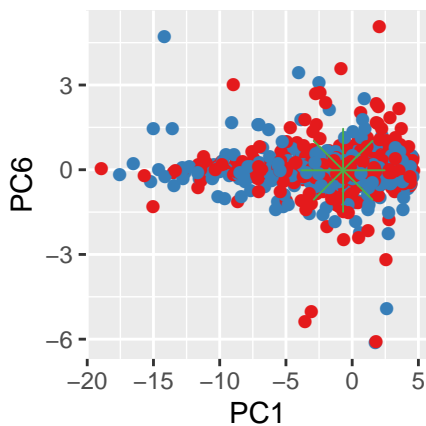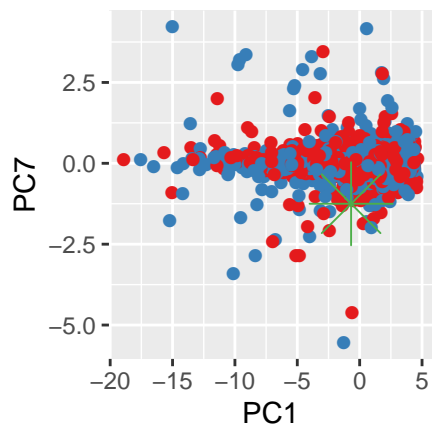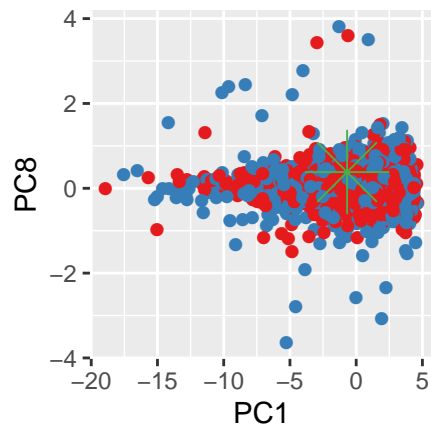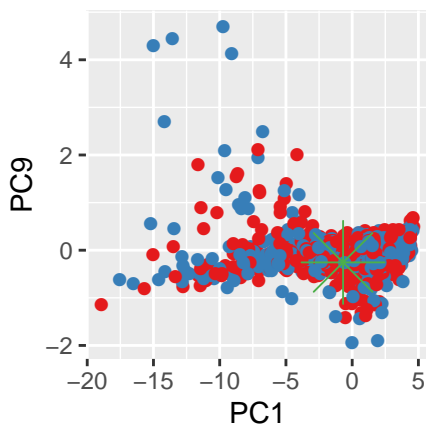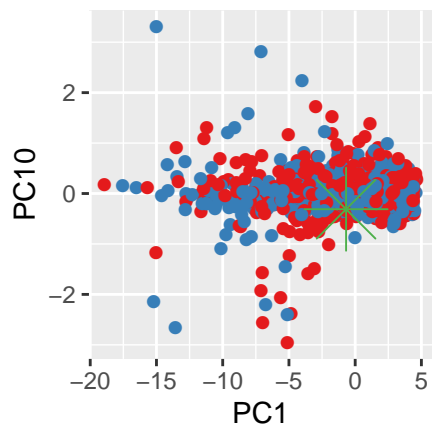

# Stephanophorus diadematus Island

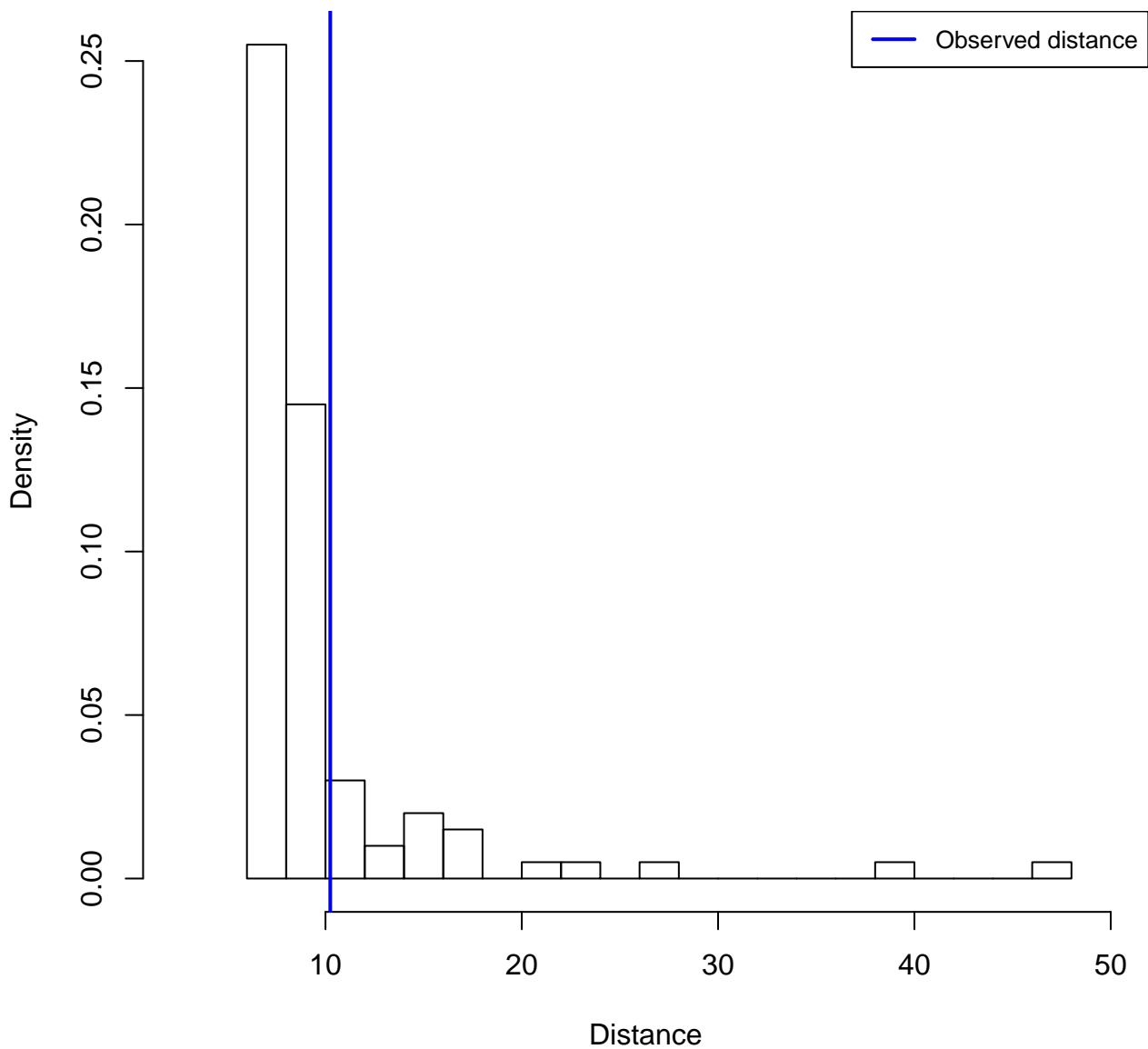

# Stephanophorus diadematus IBD

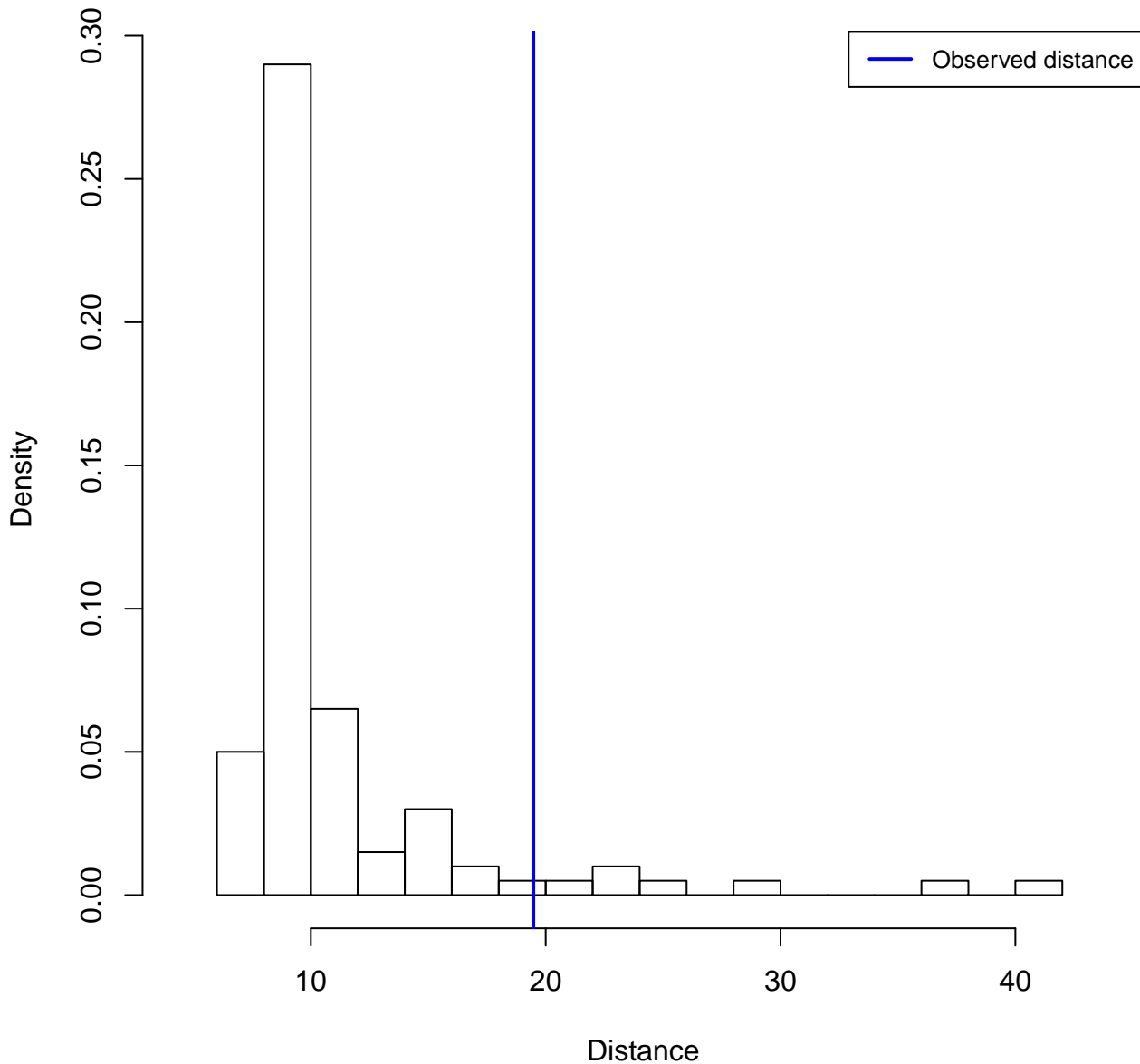

Supplement: Supplementary file 8 — Supplementary Data 5 [file 41467_2021_26537_MOESM8_ESM.gz › PCAs/diadematus_N_PCA.pdf]

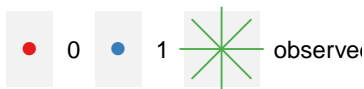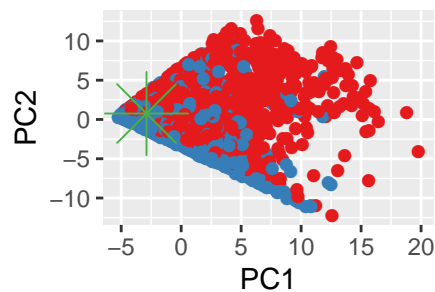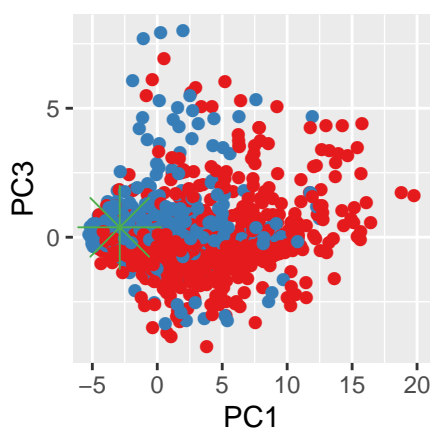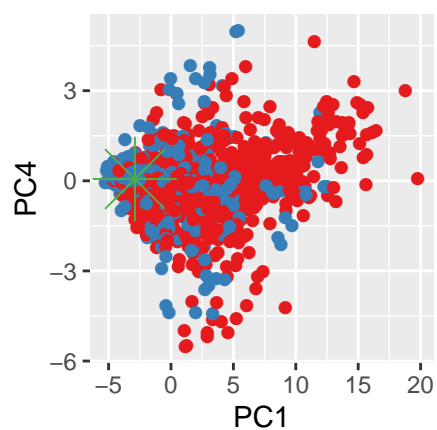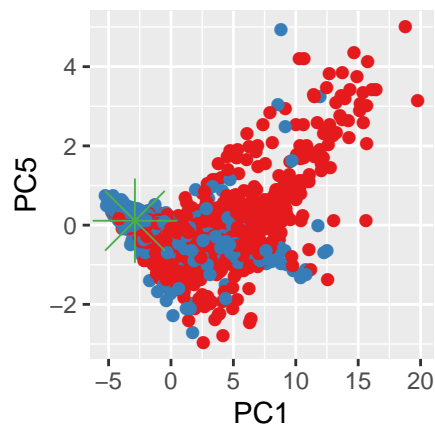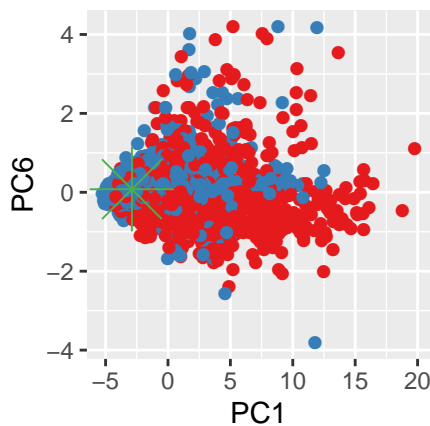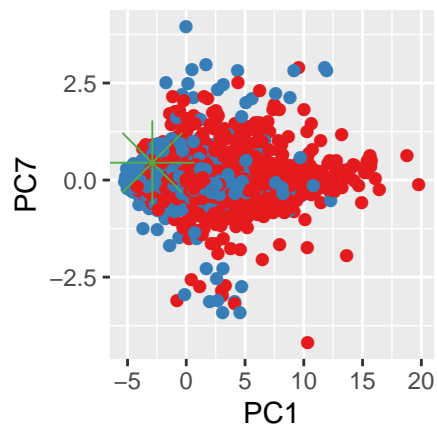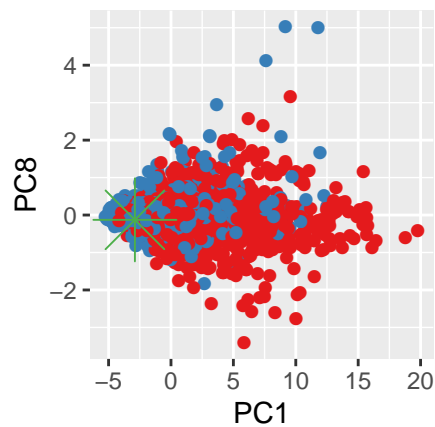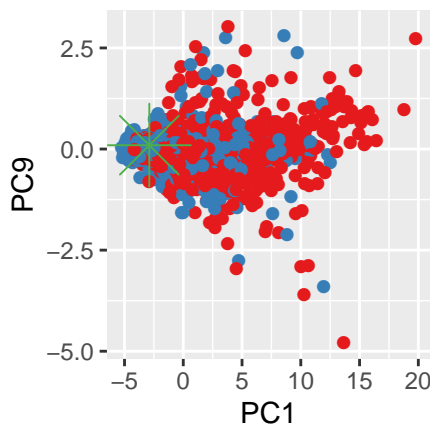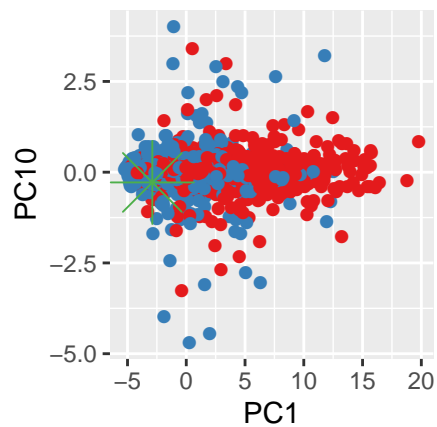

# Piculus aurulentus Island

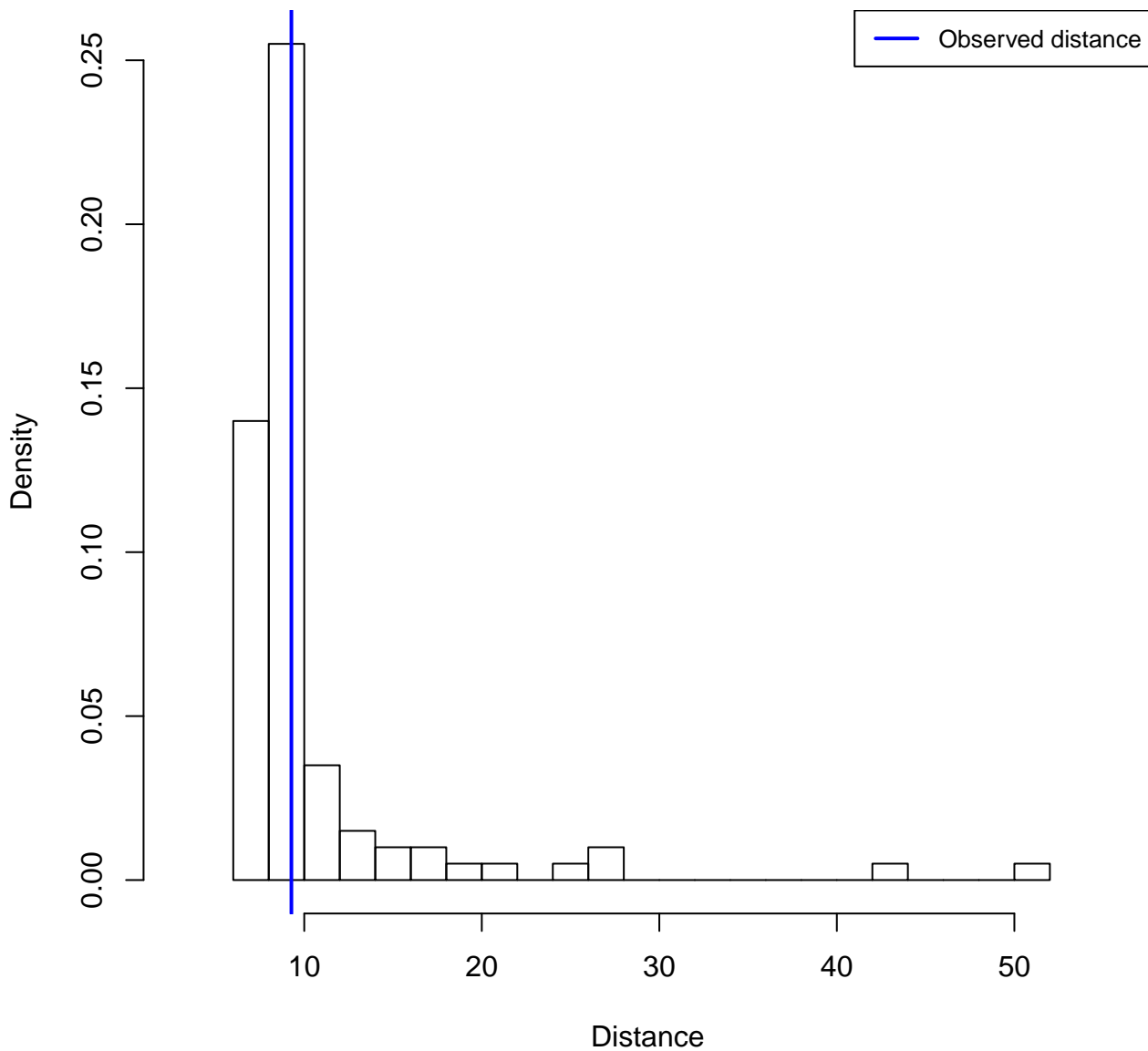

# Piculus aurulentus IBD

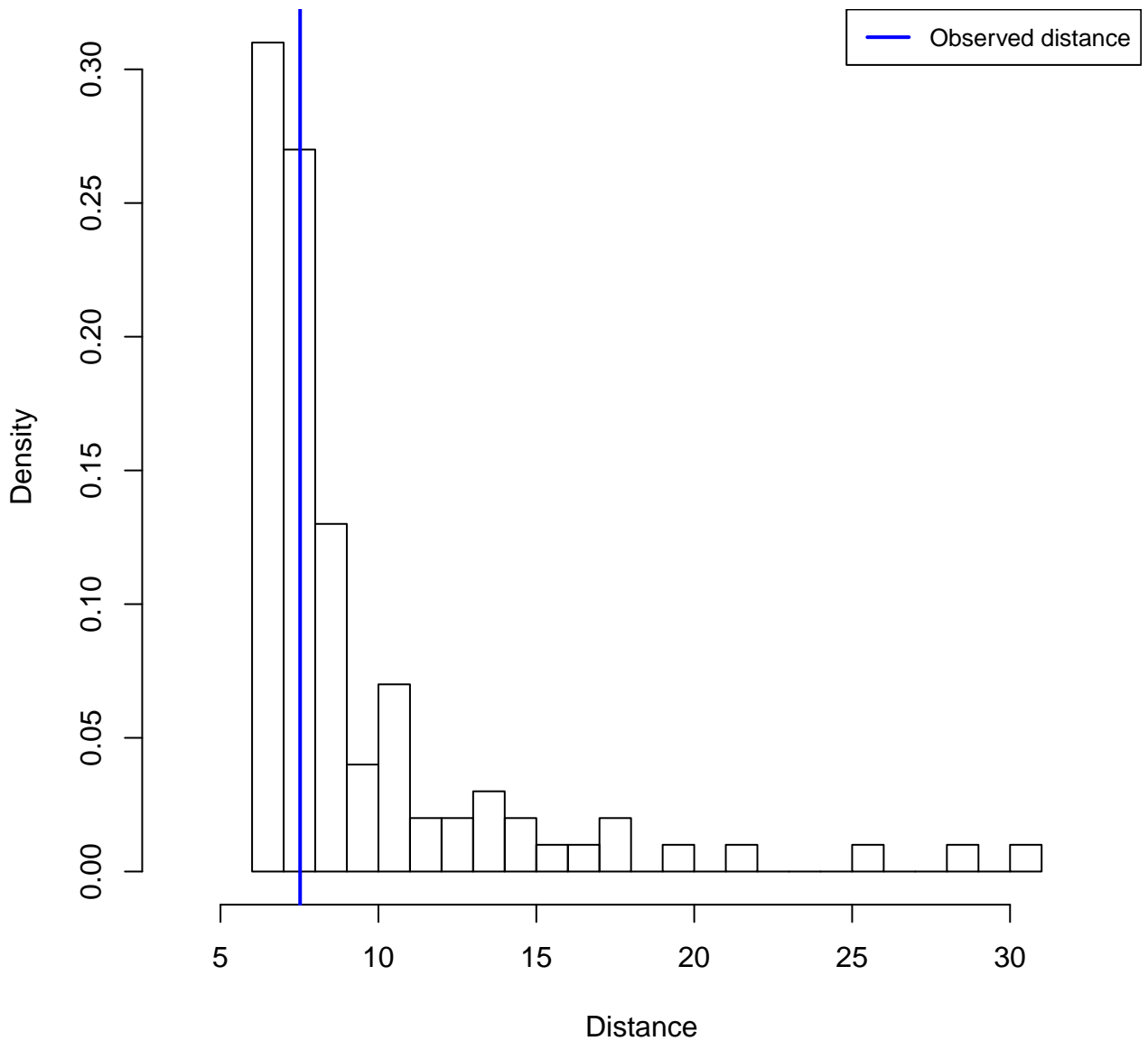

Supplement: Supplementary file 8 — Supplementary Data 5 [file 41467_2021_26537_MOESM8_ESM.gz › PCAs/aurulentus_S_PCA.pdf]

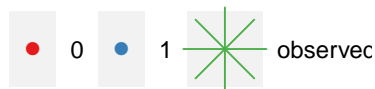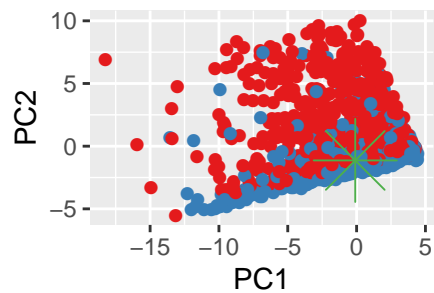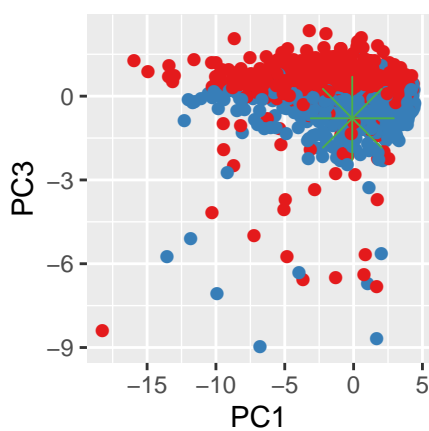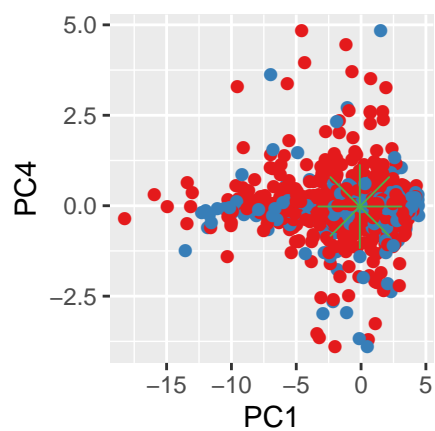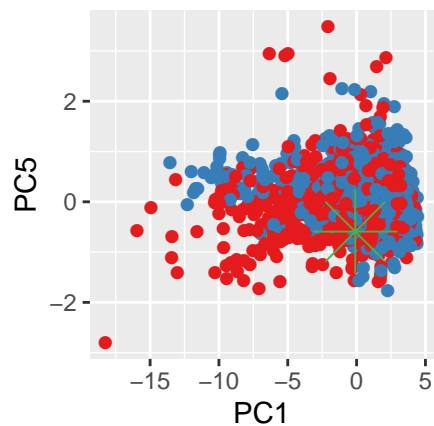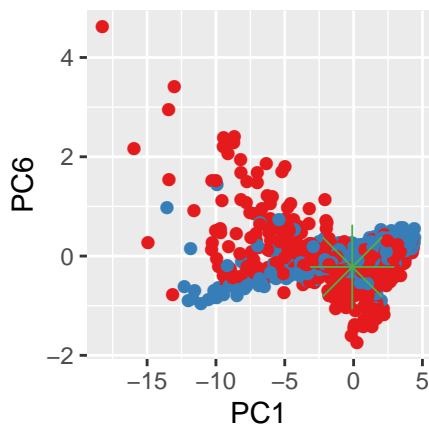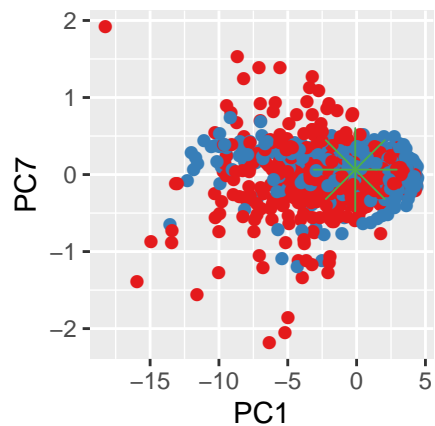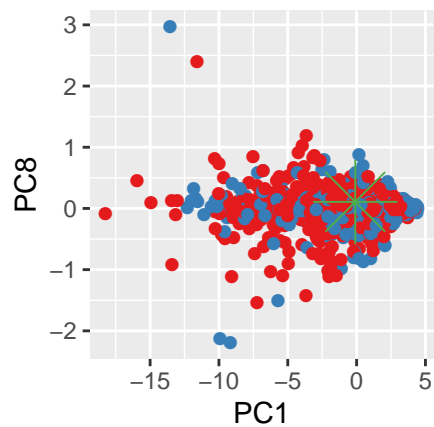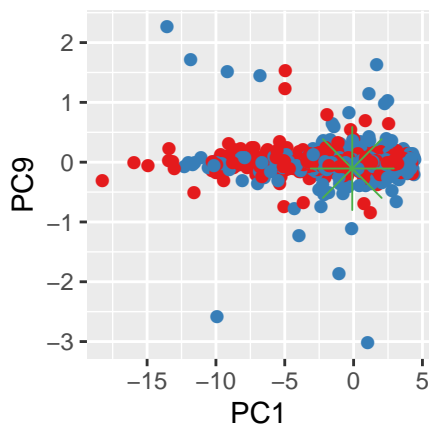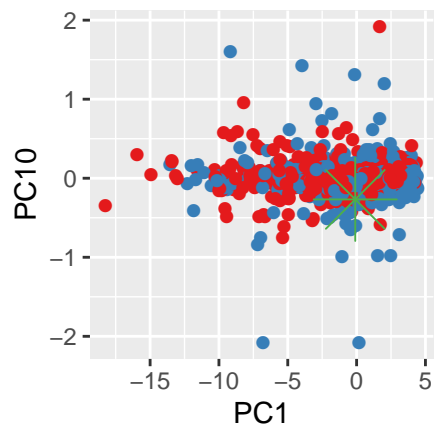

# Heliobletus contaminatus Island

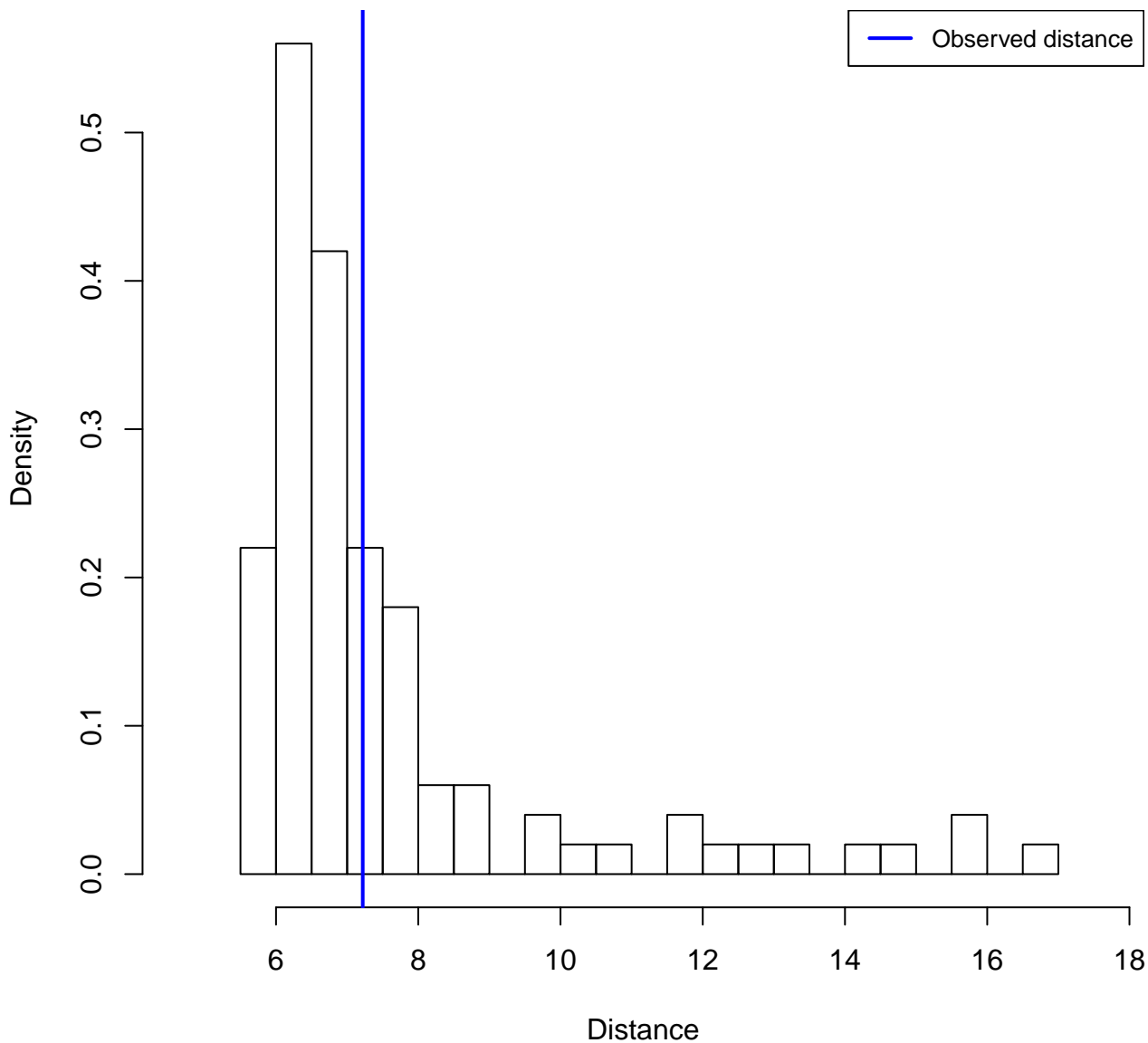

# Heliobletus contaminatus IBD

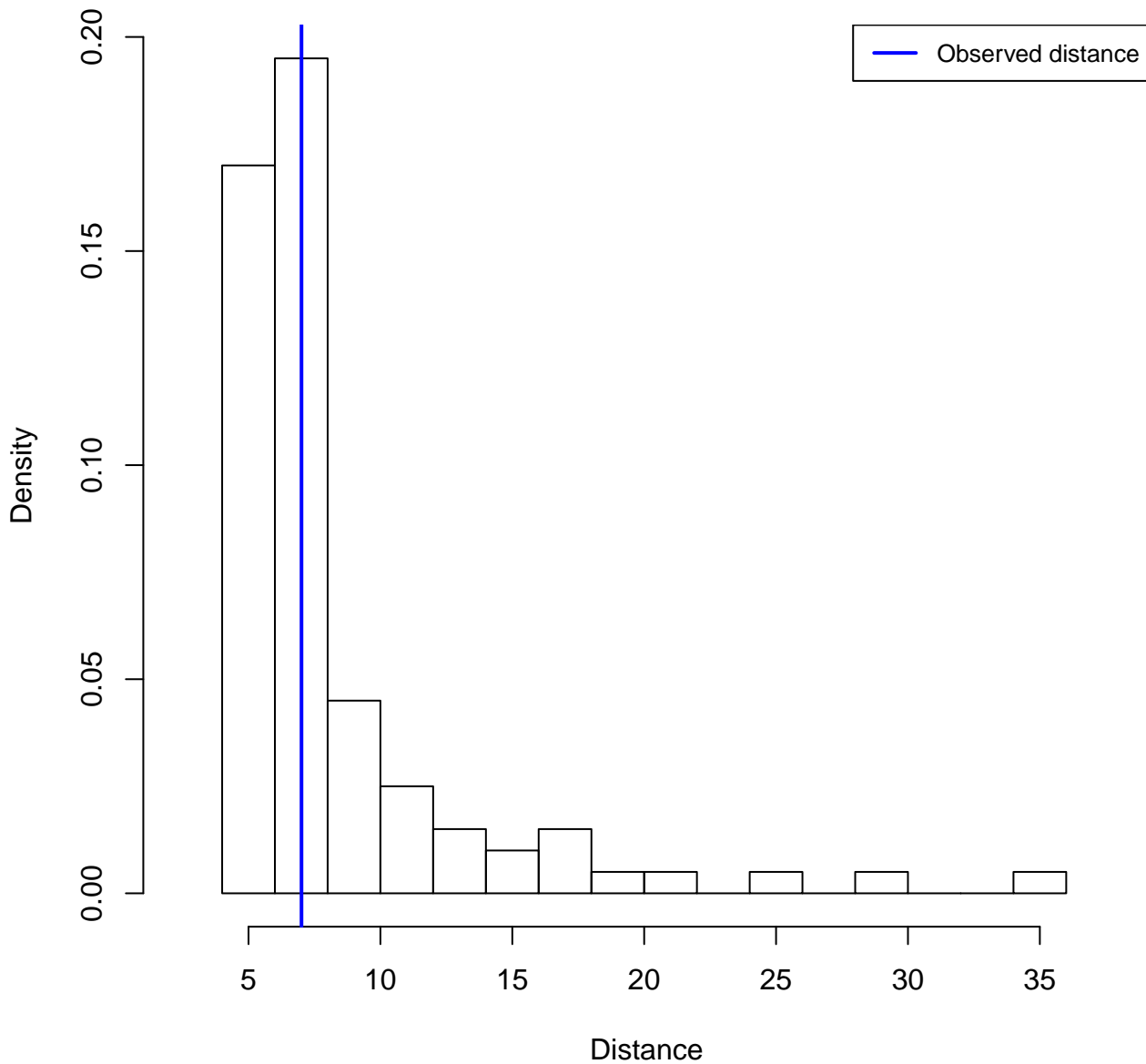

Supplement: Supplementary file 8 — Supplementary Data 5 [file 41467_2021_26537_MOESM8_ESM.gz › PCAs/contaminatus_N_PCA.pdf]

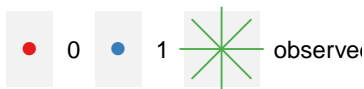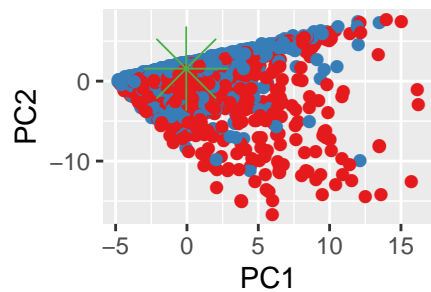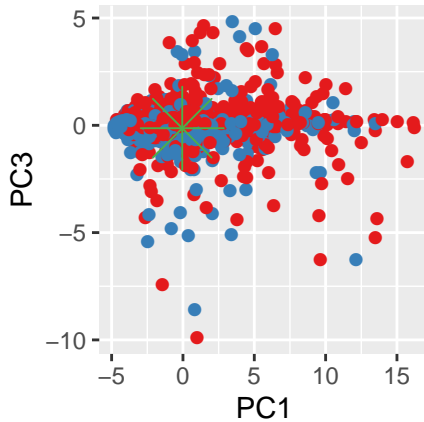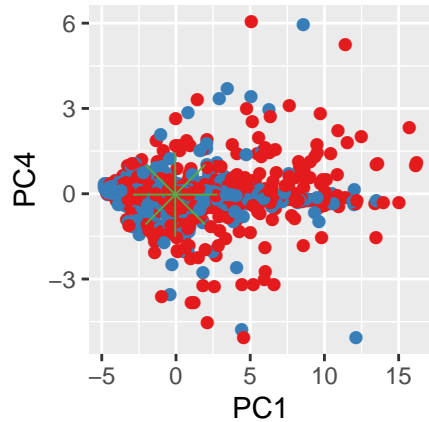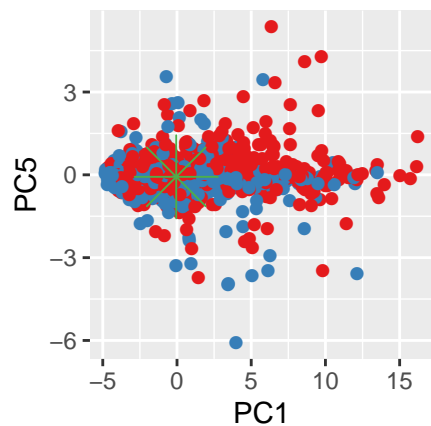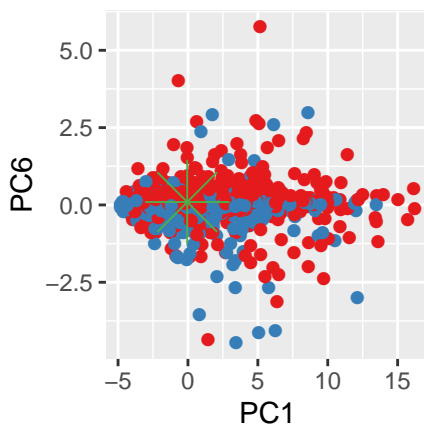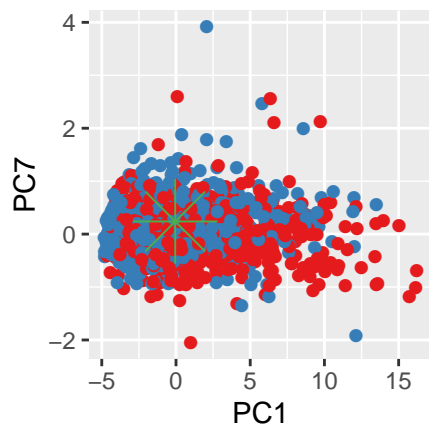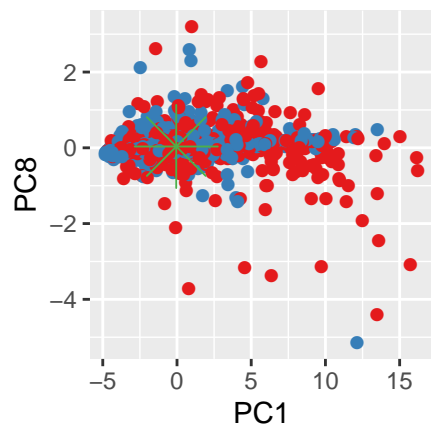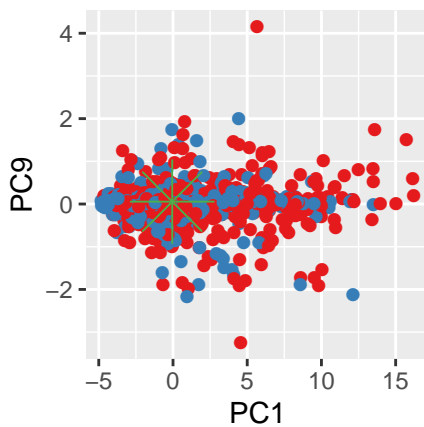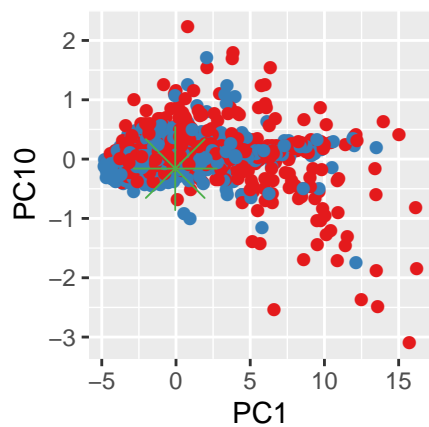

# Stephanoxix lalandi Island

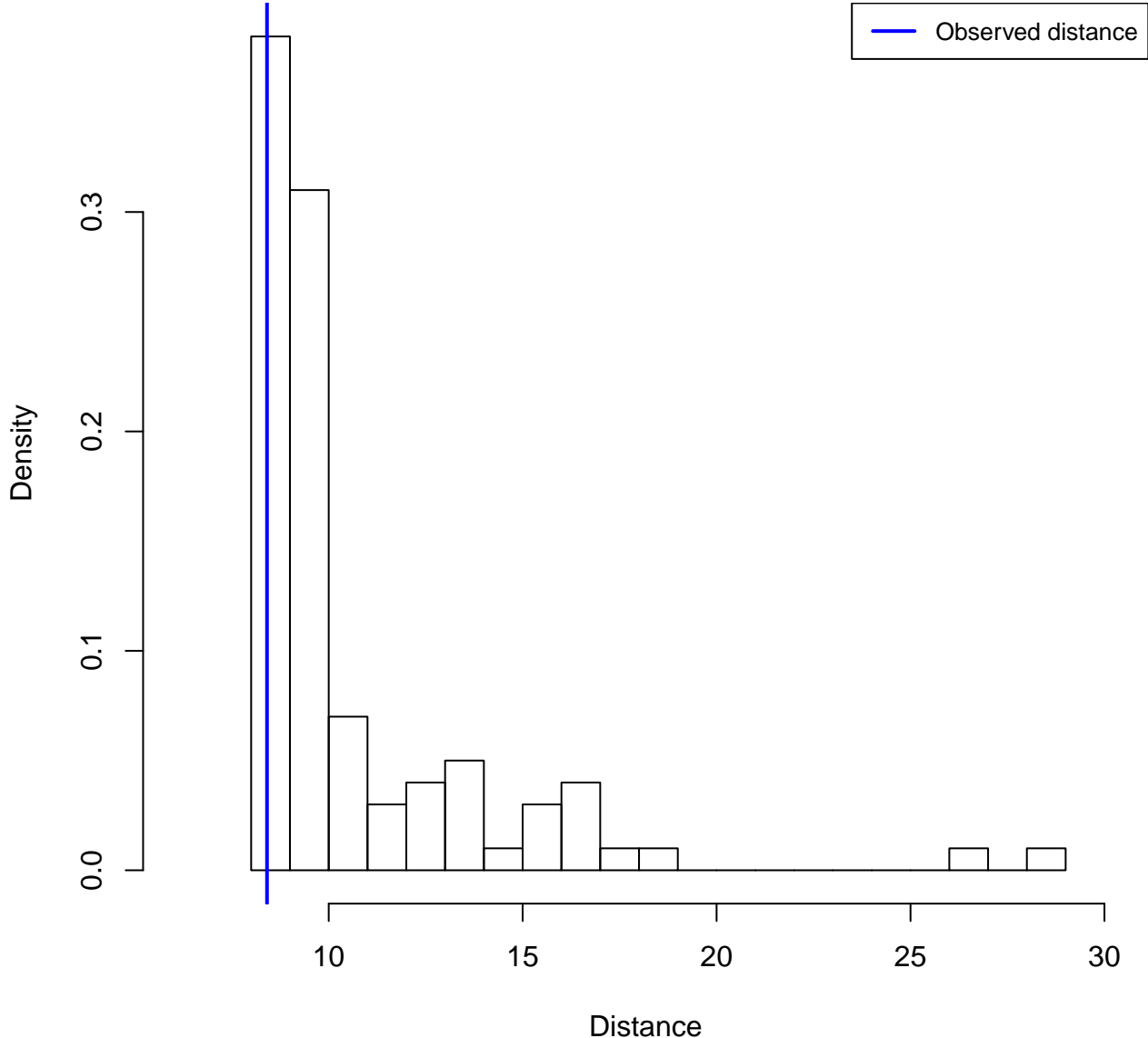

# Stephanoxix lalandi IBD

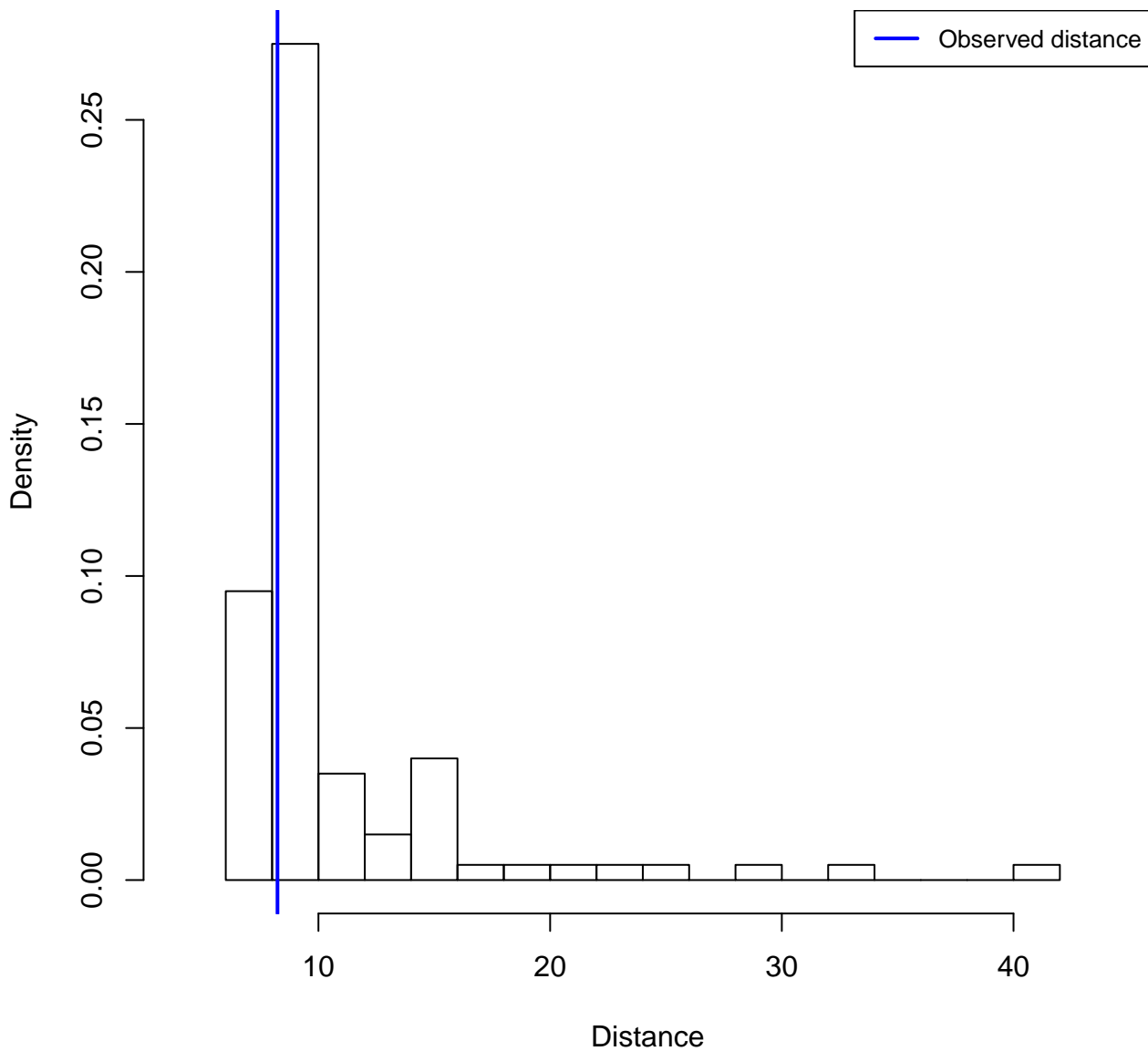

Supplement: Supplementary file 8 — Supplementary Data 5 [file 41467_2021_26537_MOESM8_ESM.gz › PCAs/lalandi_N_PCA.pdf]

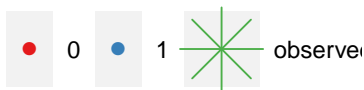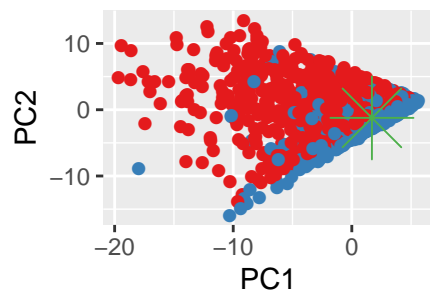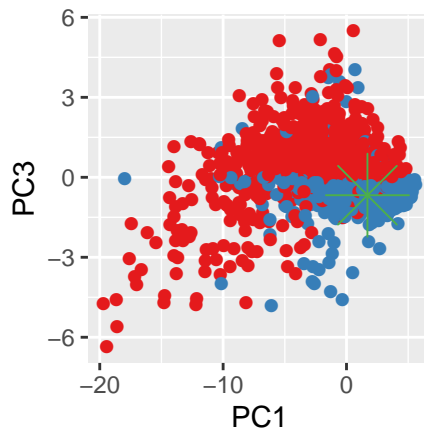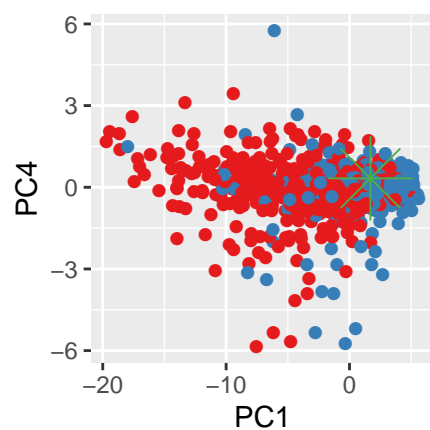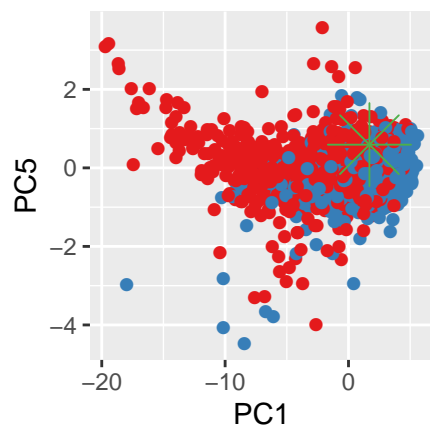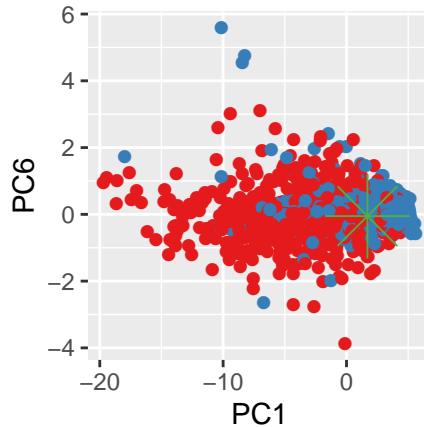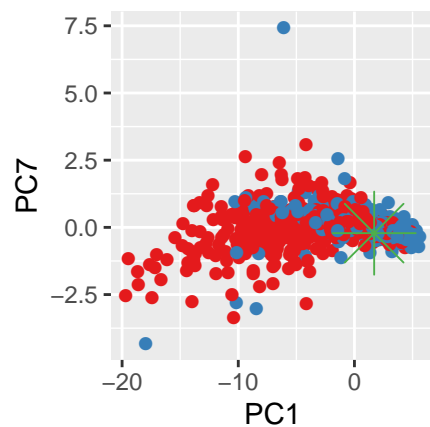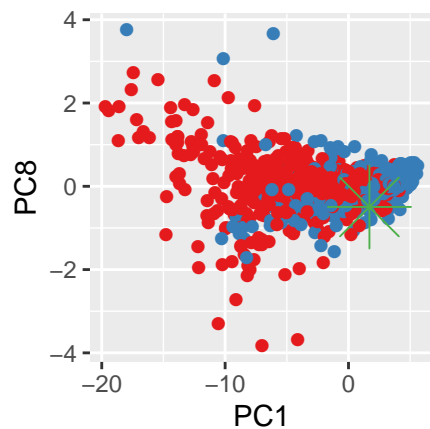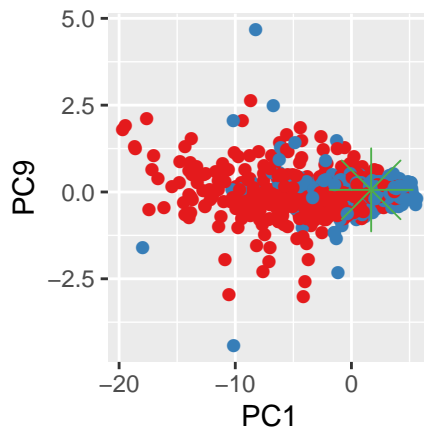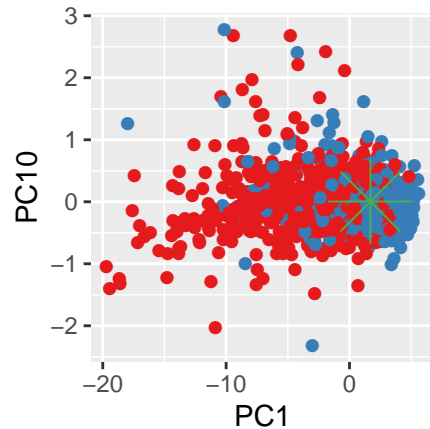

# Castanozoster thoracicus Island

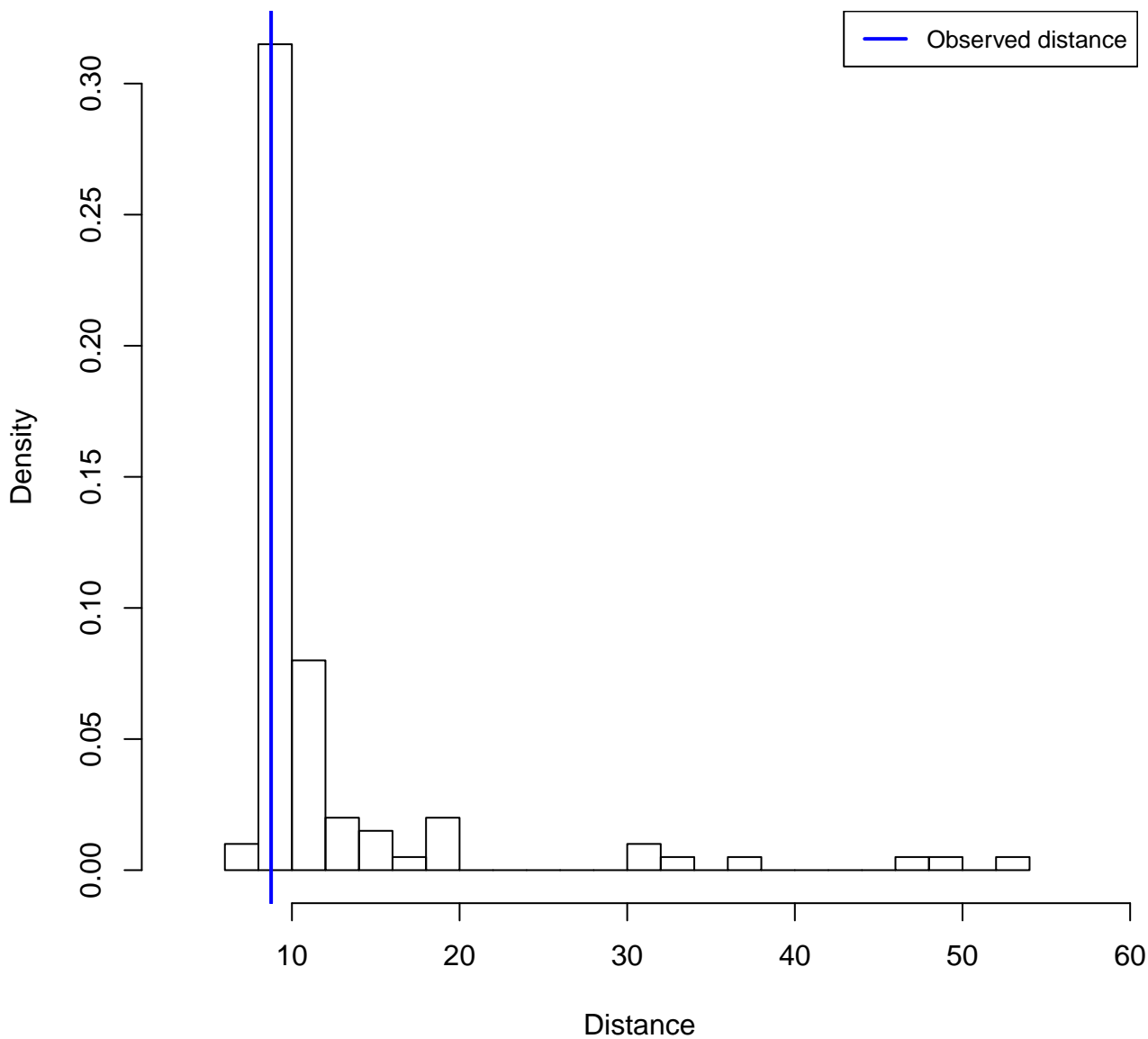

# Castanozoster thoracicus IBD

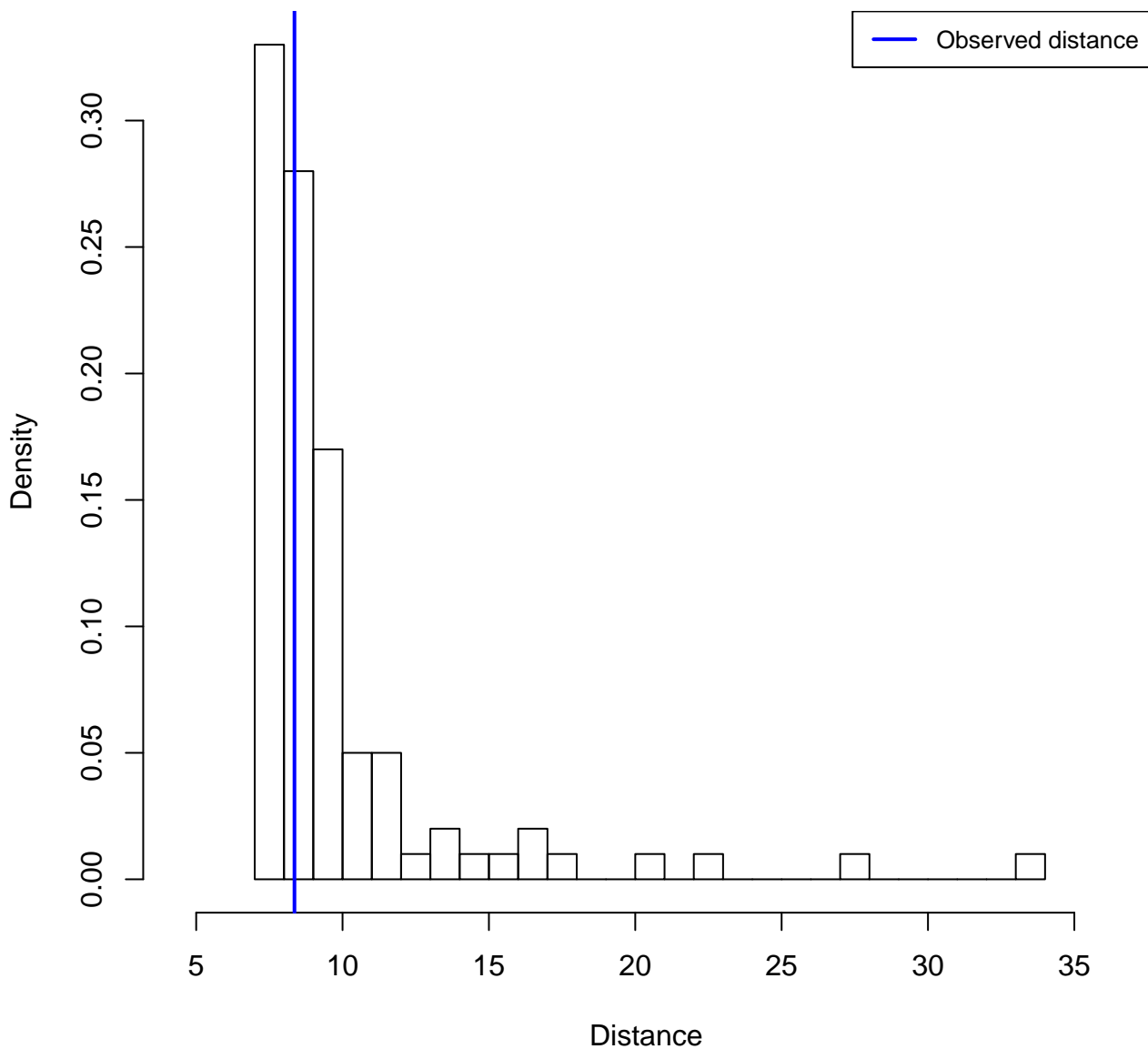

Supplement: Supplementary file 8 — Supplementary Data 5 [file 41467_2021_26537_MOESM8_ESM.gz › PCAs/thoracica_S_PCA.pdf]

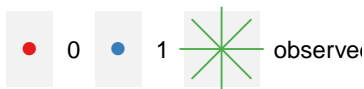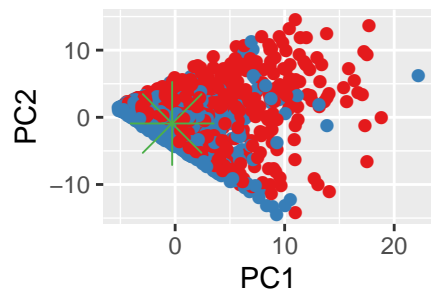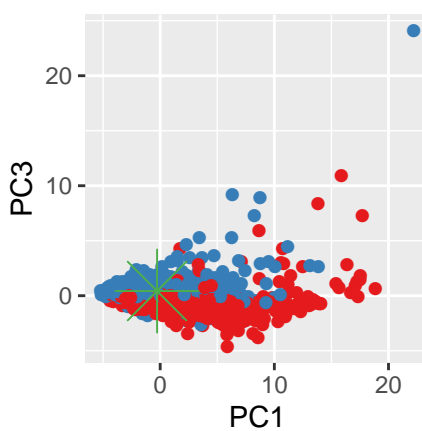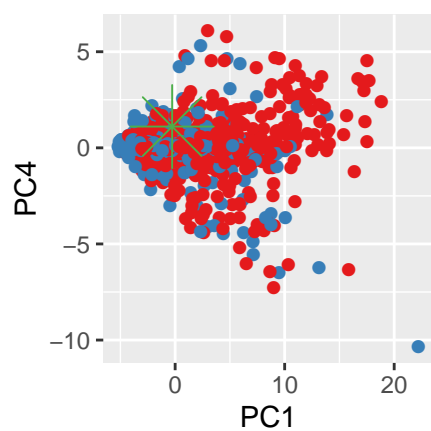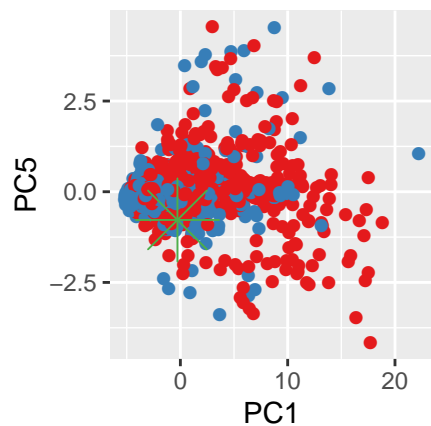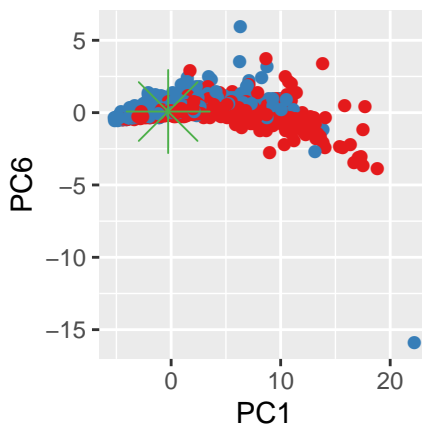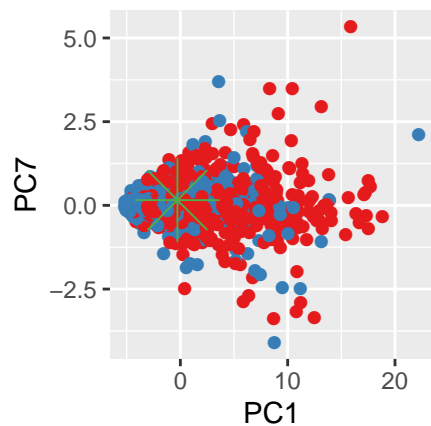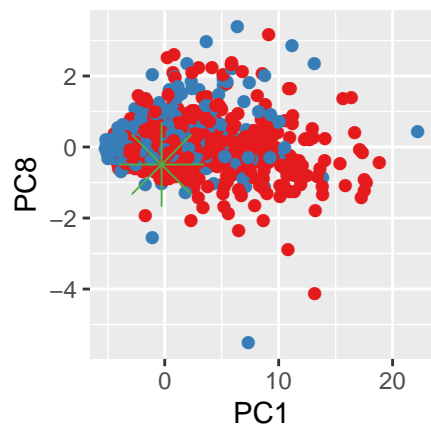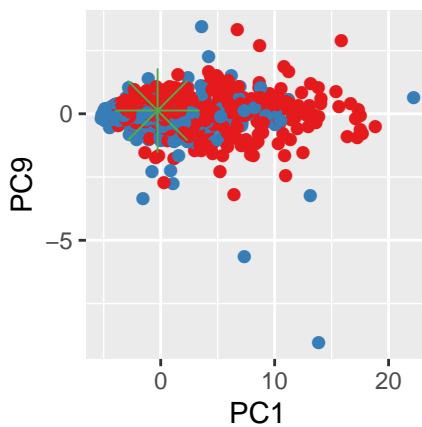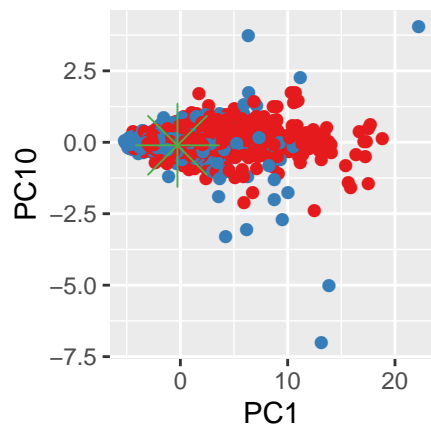

# Microspingus lateralis Island

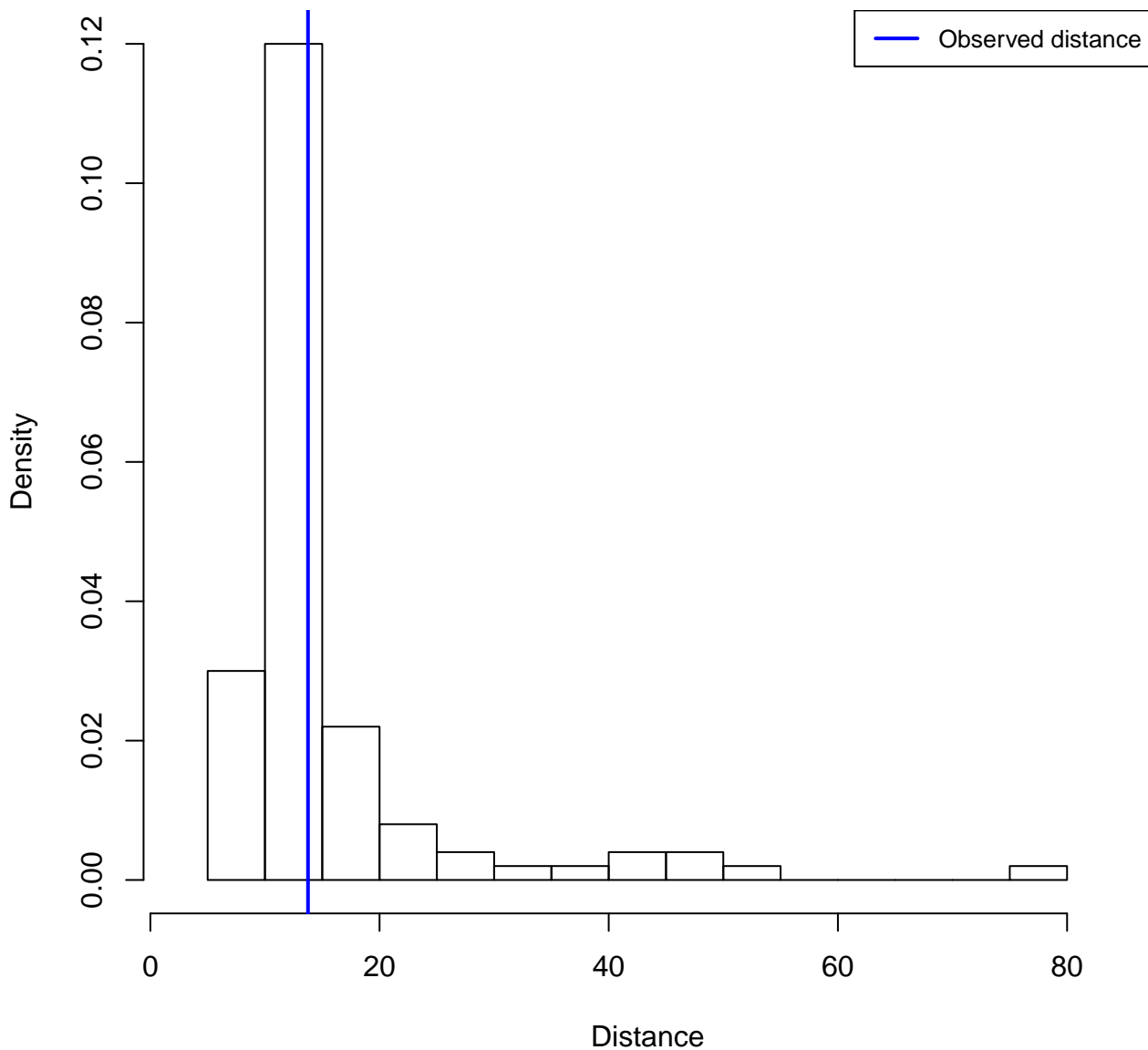

# Microspingus lateralis IBD

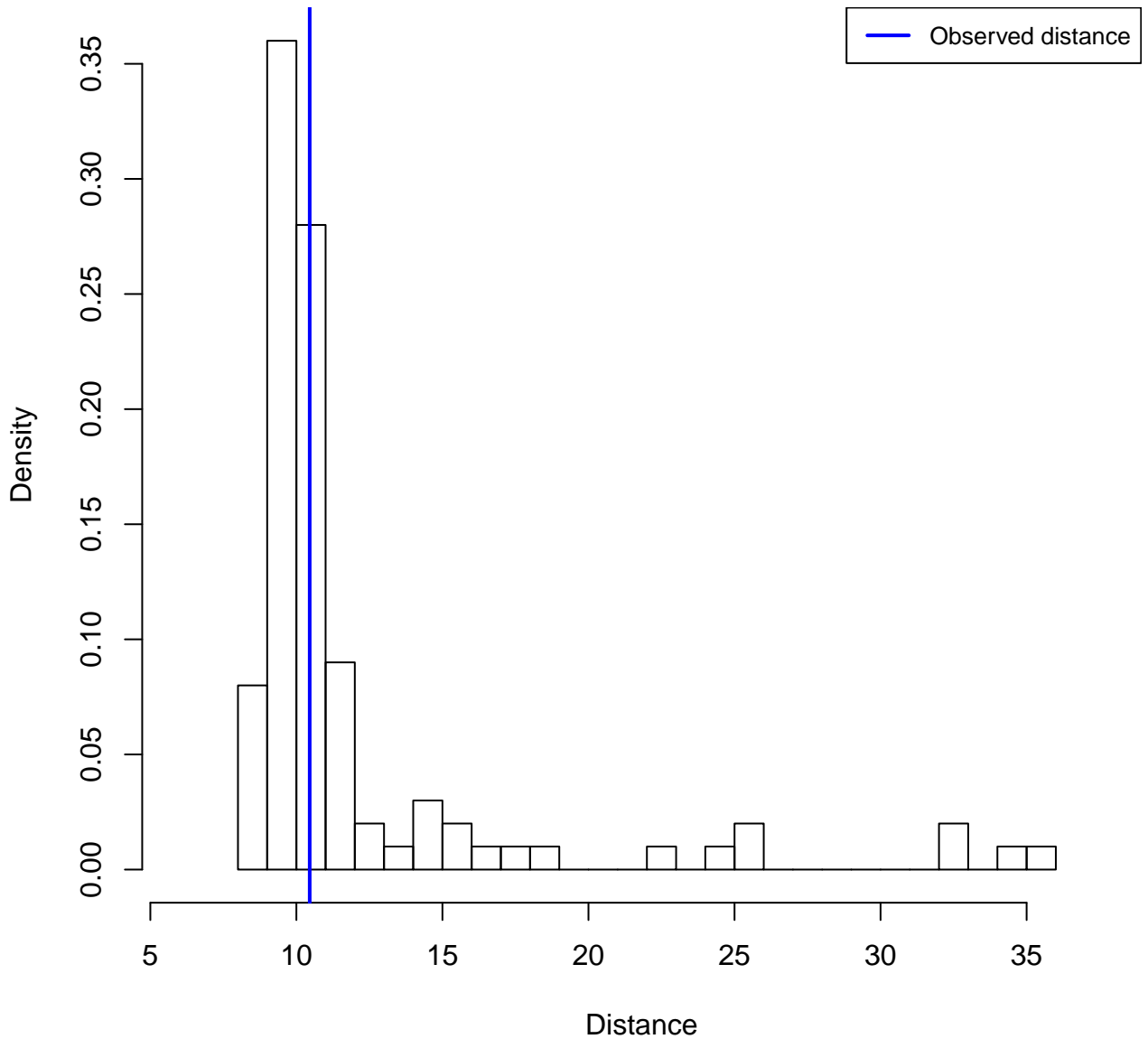

Supplement: Supplementary file 8 — Supplementary Data 5 [file 41467_2021_26537_MOESM8_ESM.gz › PCAs/lateralis_N_PCA.pdf]

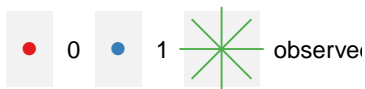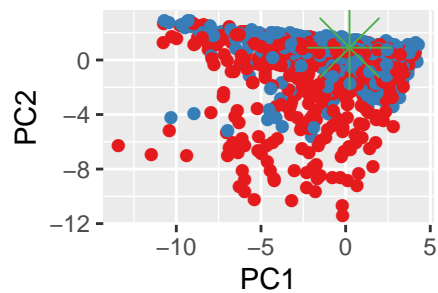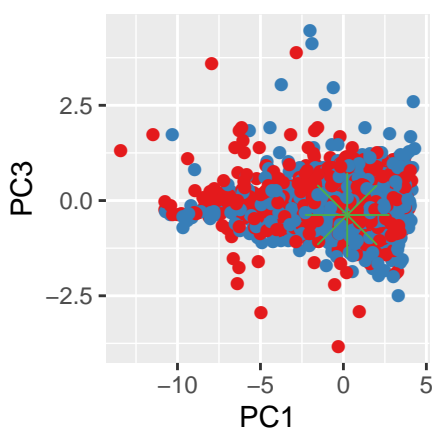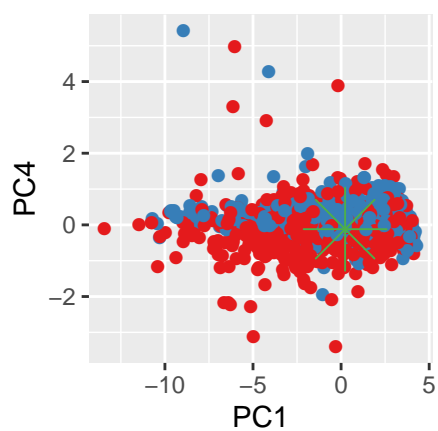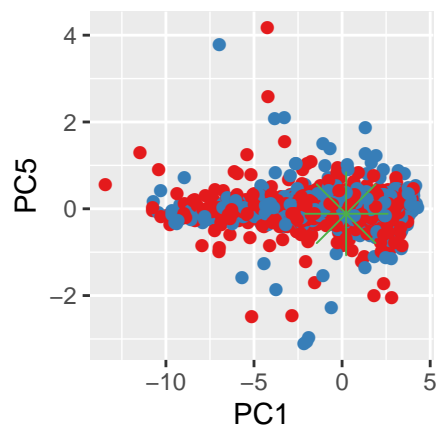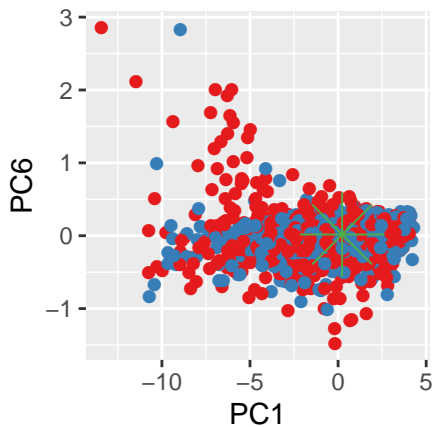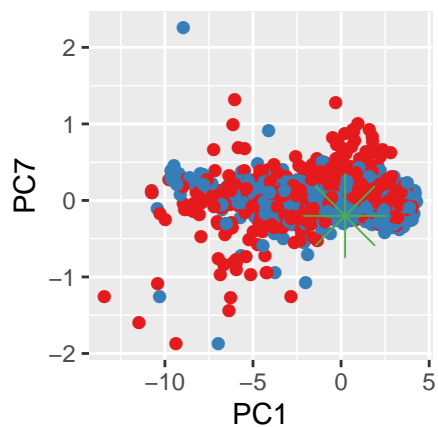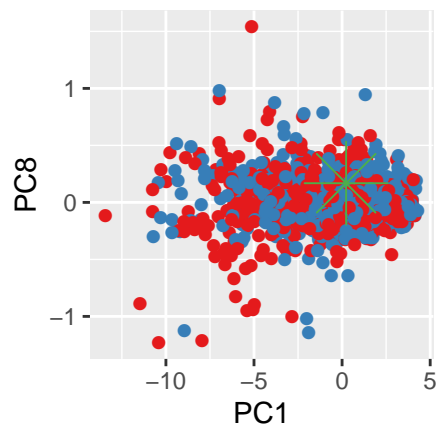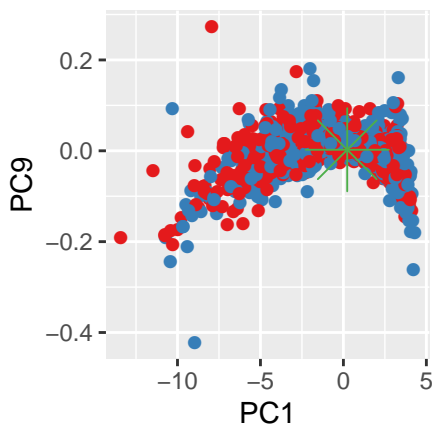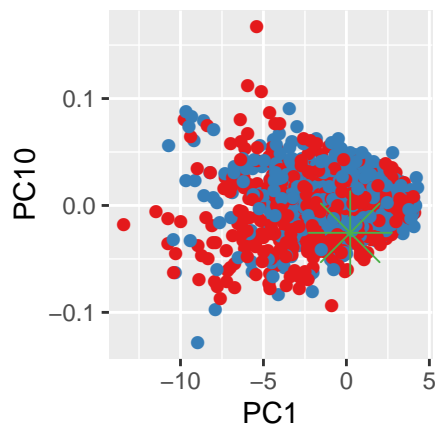

# Dysithamnus xanthopterus Island

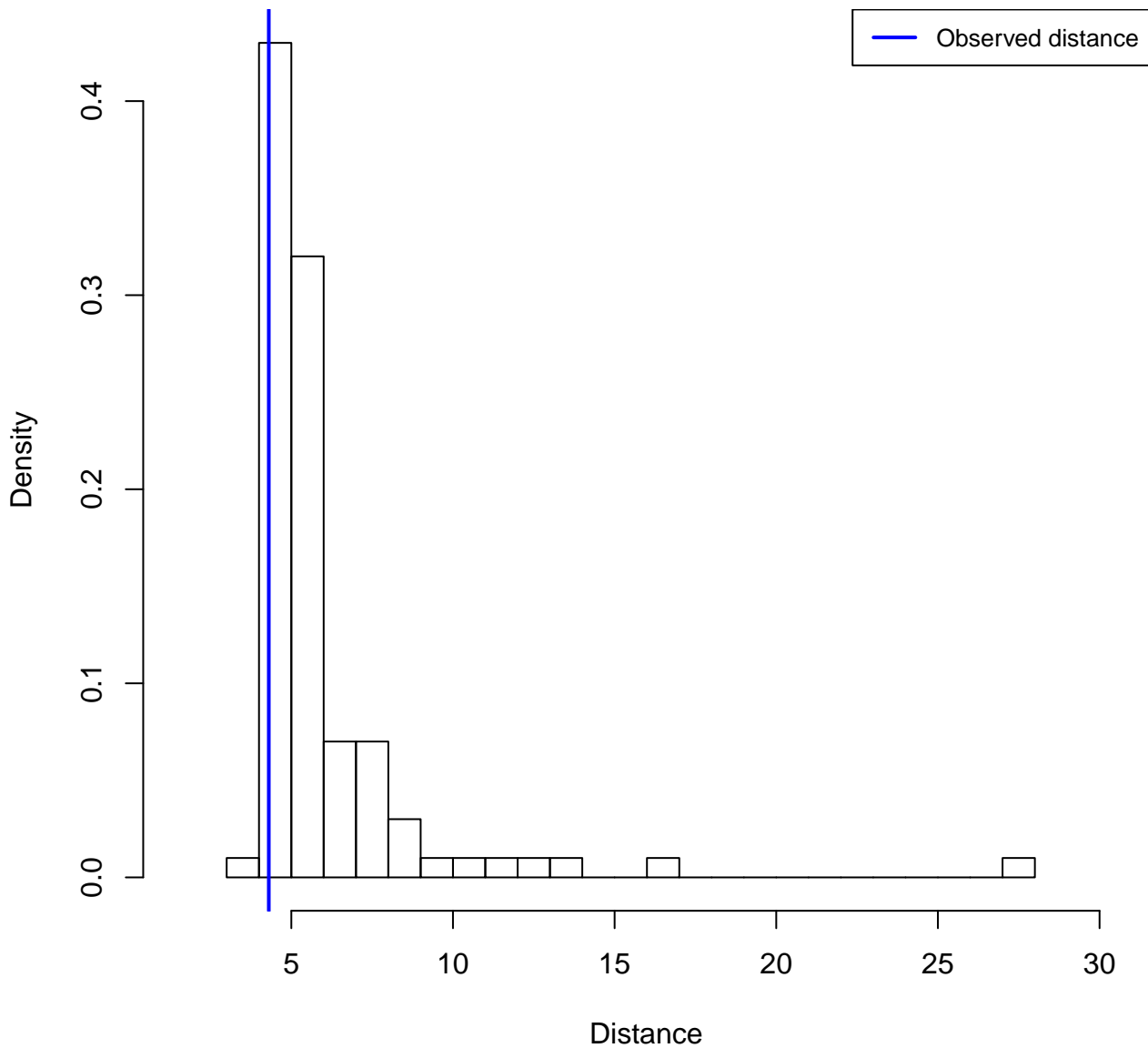

# Dysithamnus xanthopterus IBD

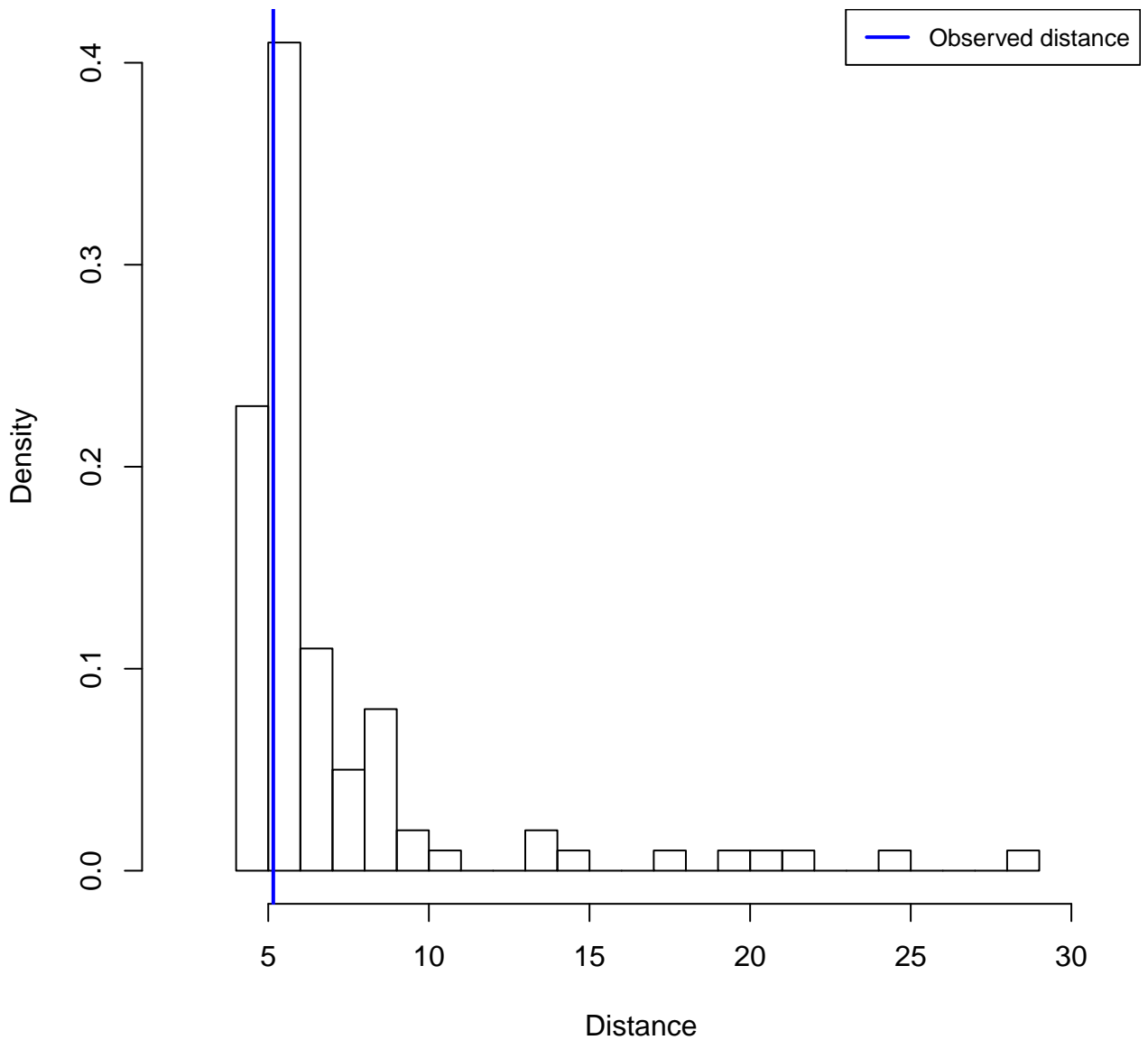

Supplement: Supplementary file 8 — Supplementary Data 5 [file 41467_2021_26537_MOESM8_ESM.gz › PCAs/xanthopterus_N_PCA.pdf]

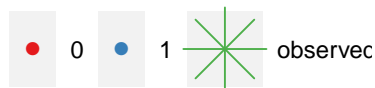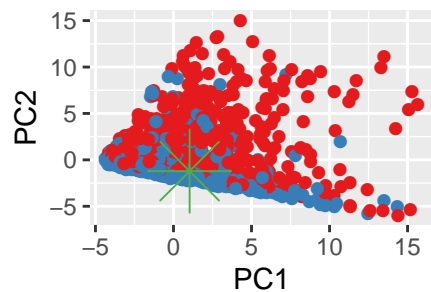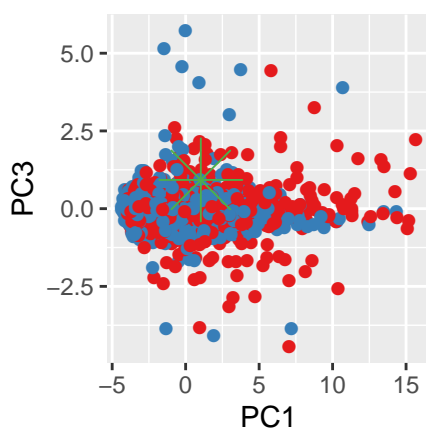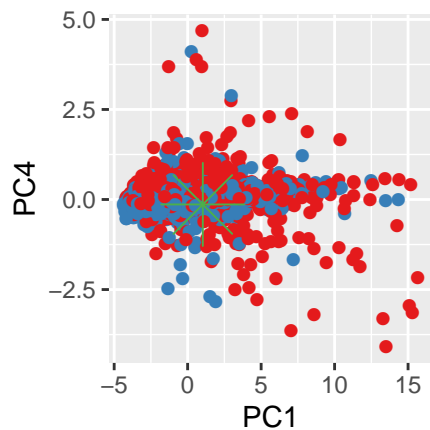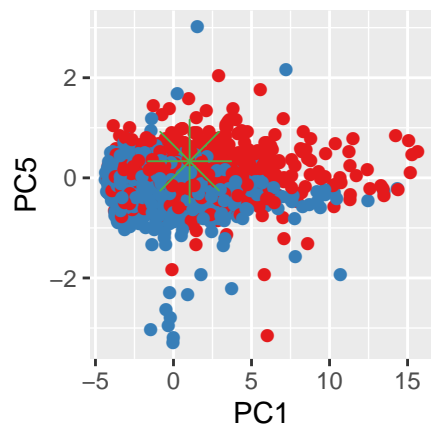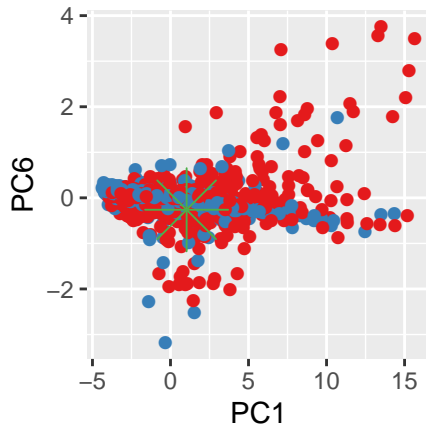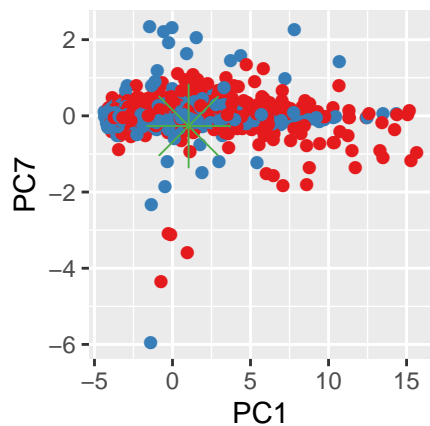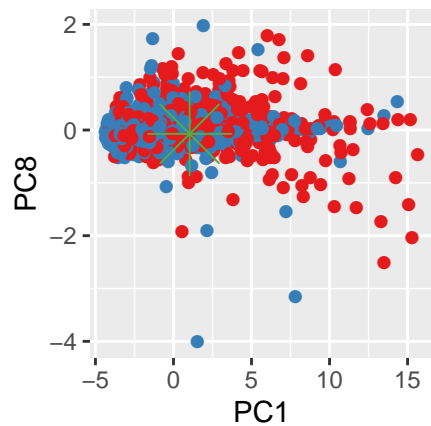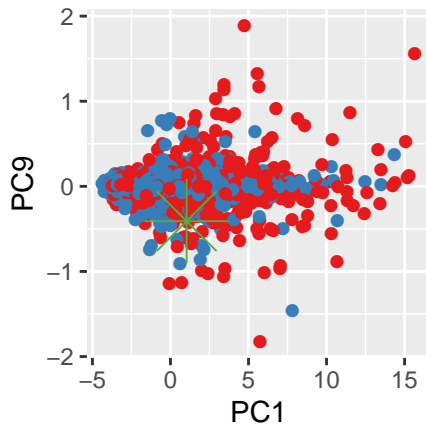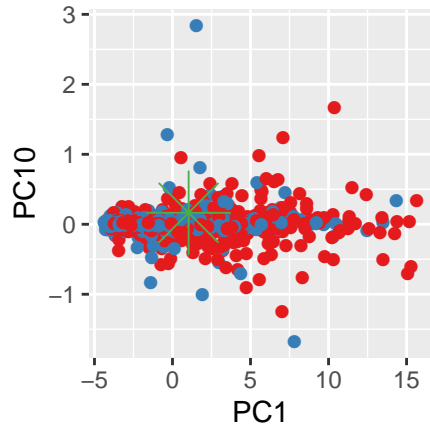

# Tangara desmaresti Island

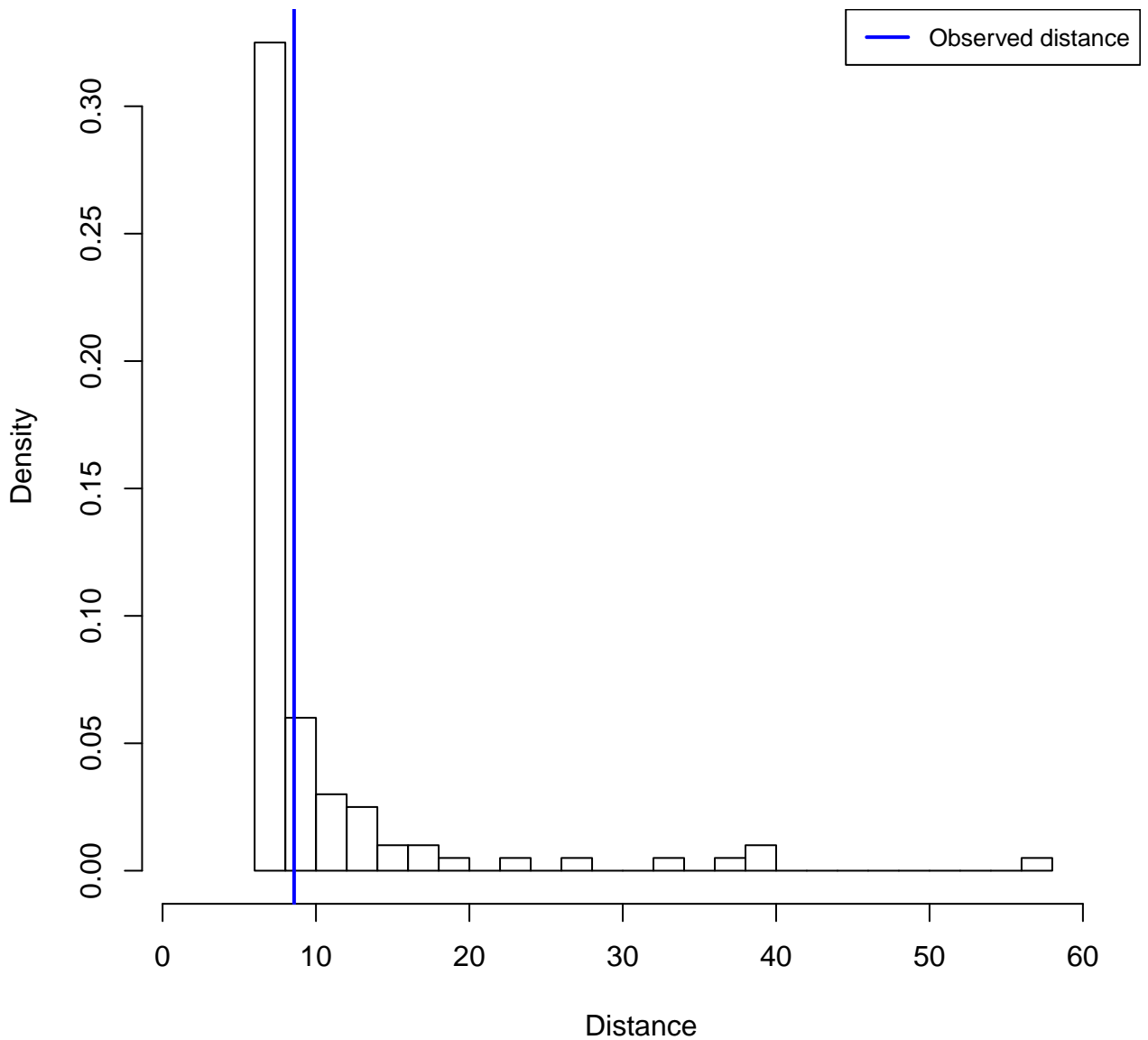

# Tangara desmaresti IBD

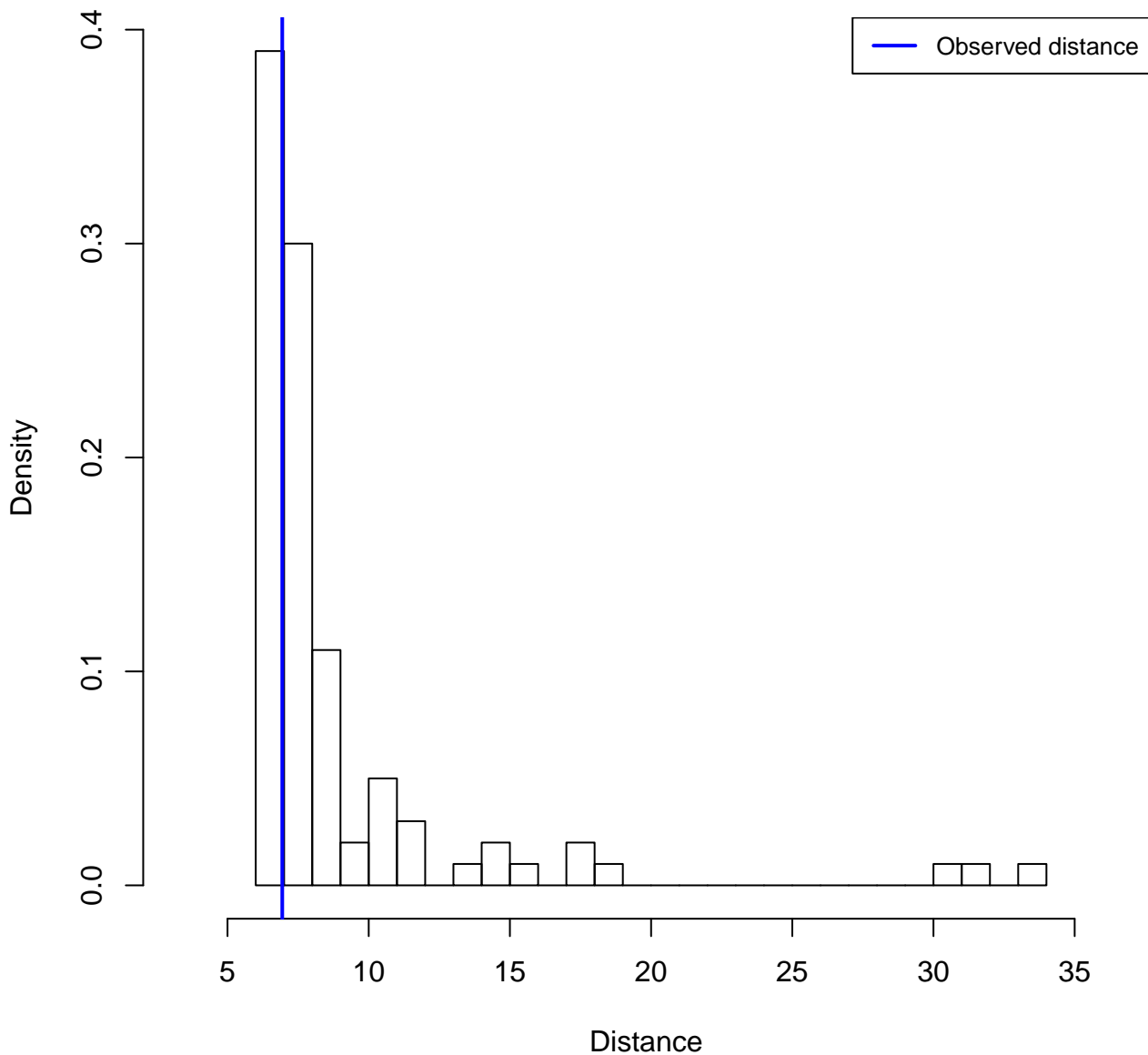

Supplement: Supplementary file 8 — Supplementary Data 5 [file 41467_2021_26537_MOESM8_ESM.gz › PCAs/desmaresti_S_PCA.pdf]

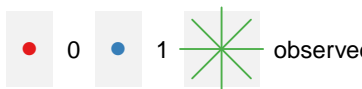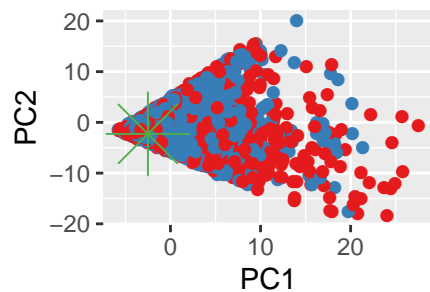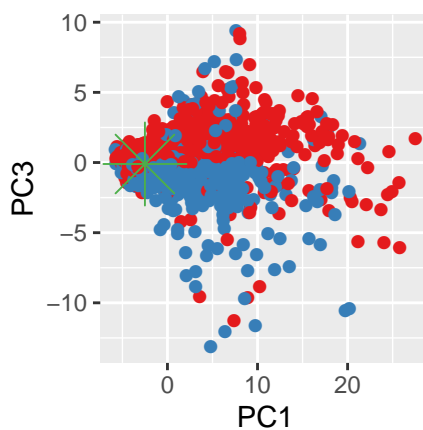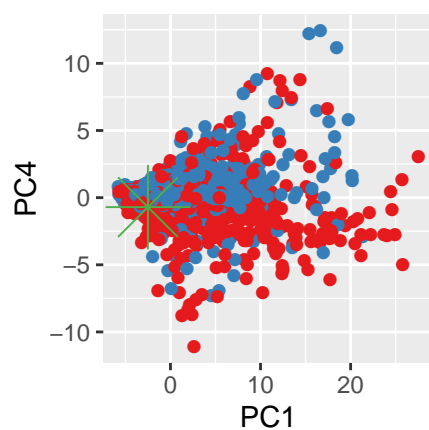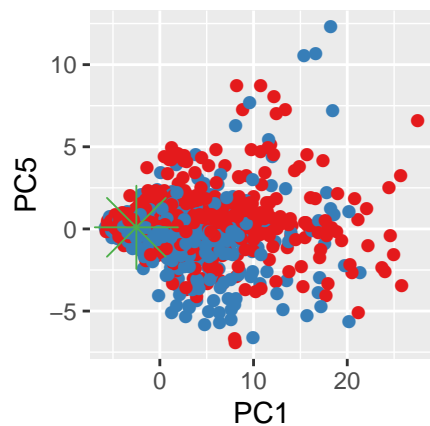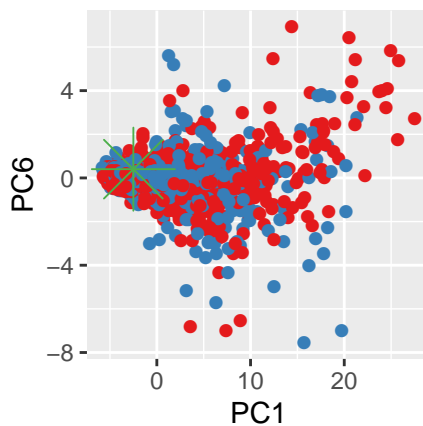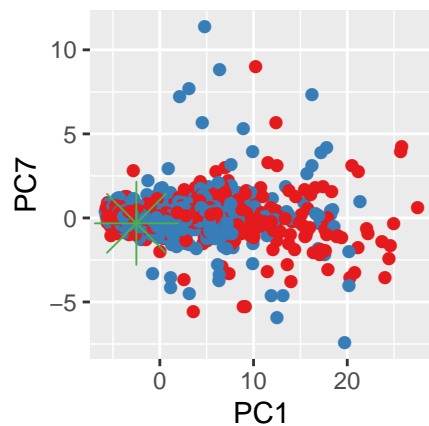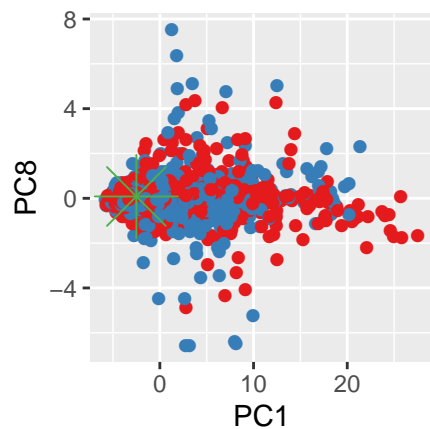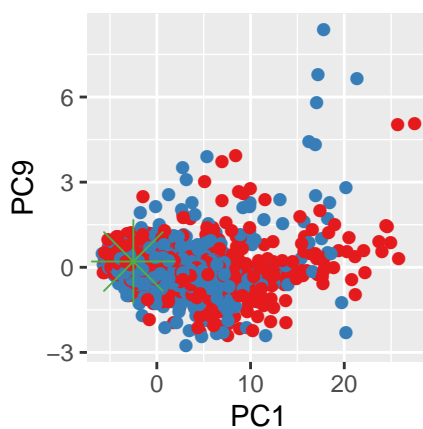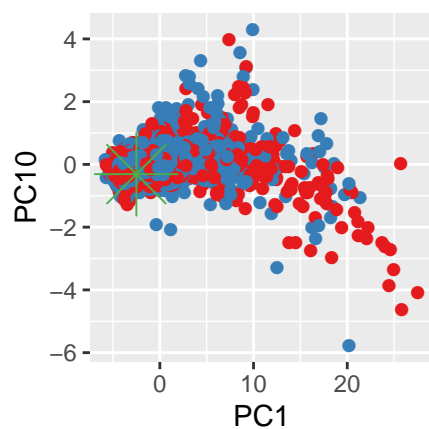

# Piculus aurulentus Island

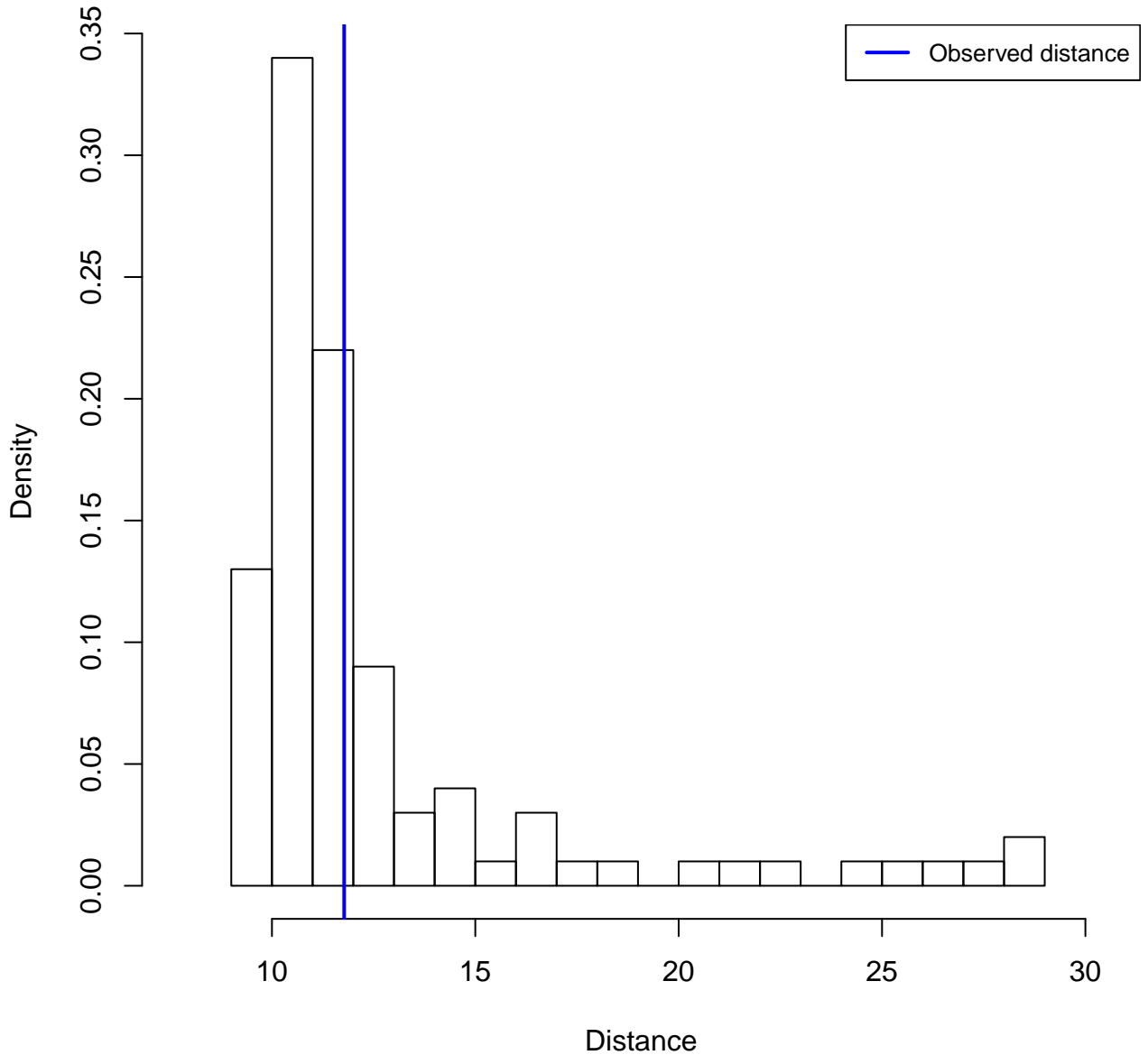

# Piculus aurulentus IBD

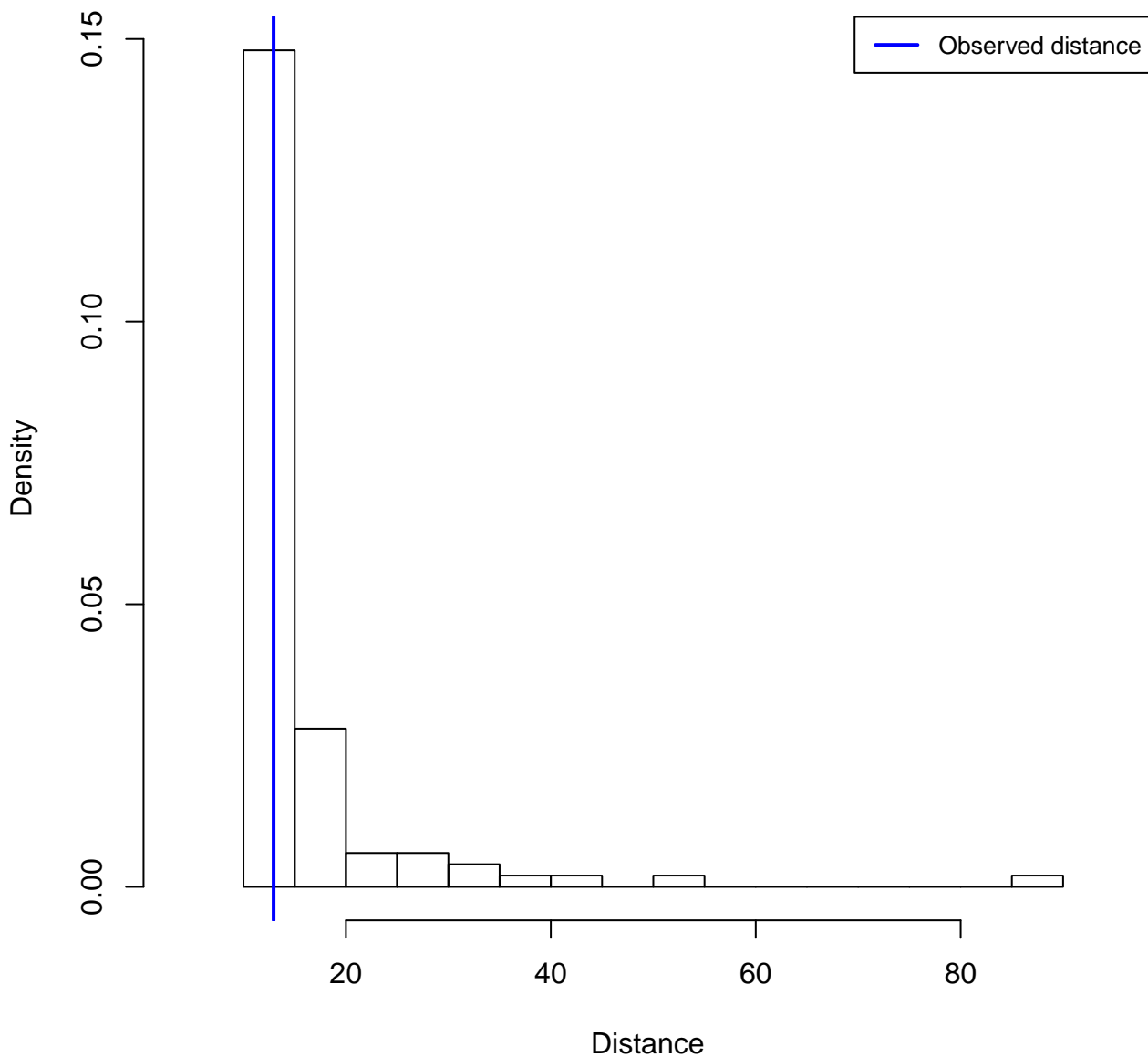

Supplement: Supplementary file 8 — Supplementary Data 5 [file 41467_2021_26537_MOESM8_ESM.gz › PCAs/aurulentus_N_PCA.pdf]

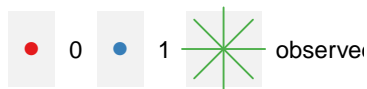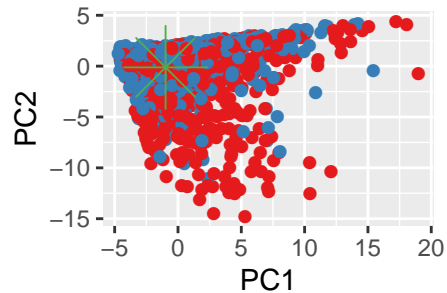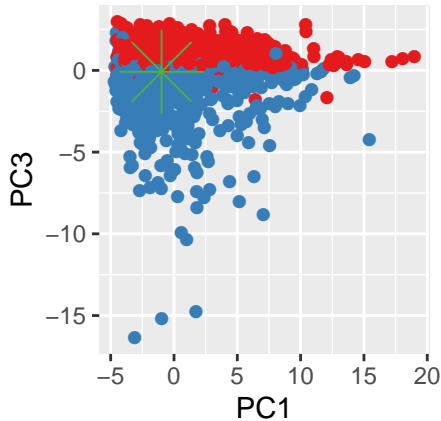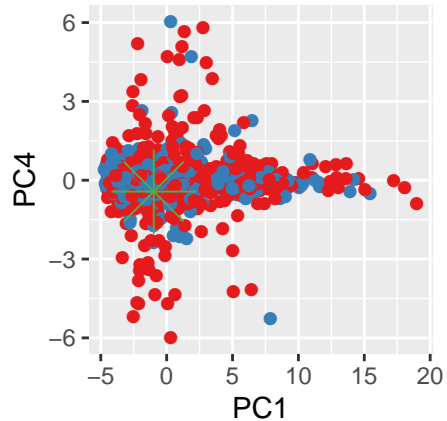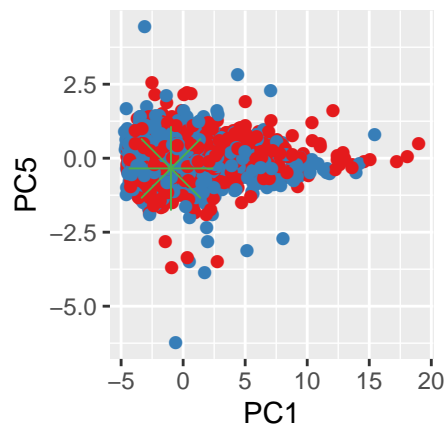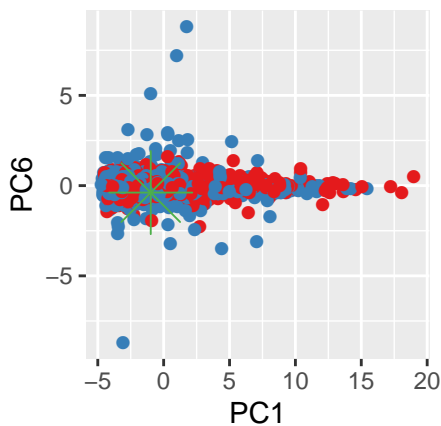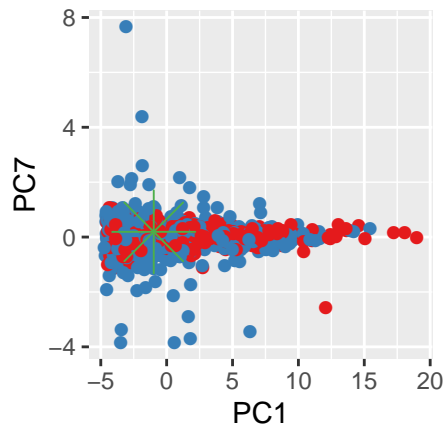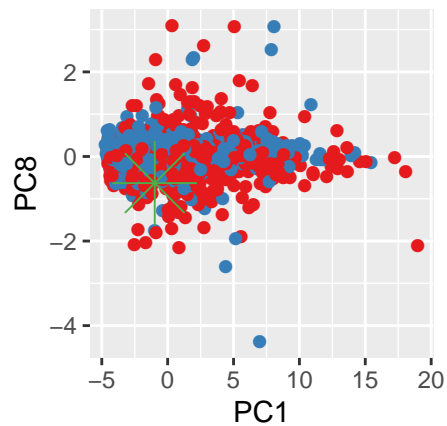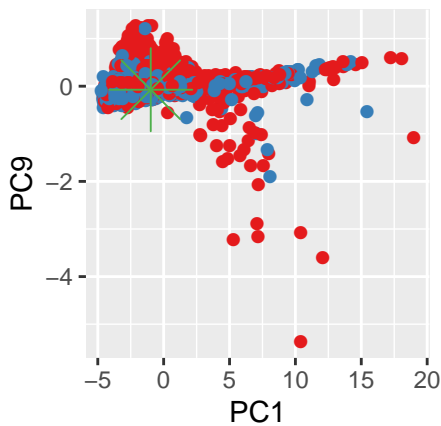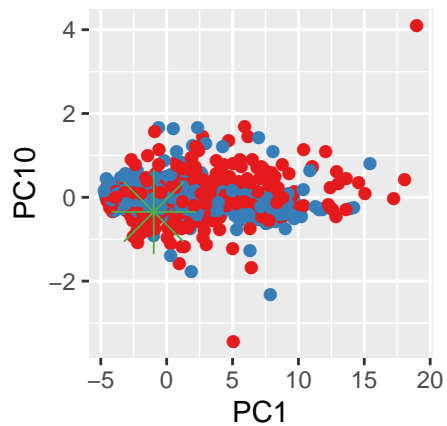

# Castanozoster thoracicus Island

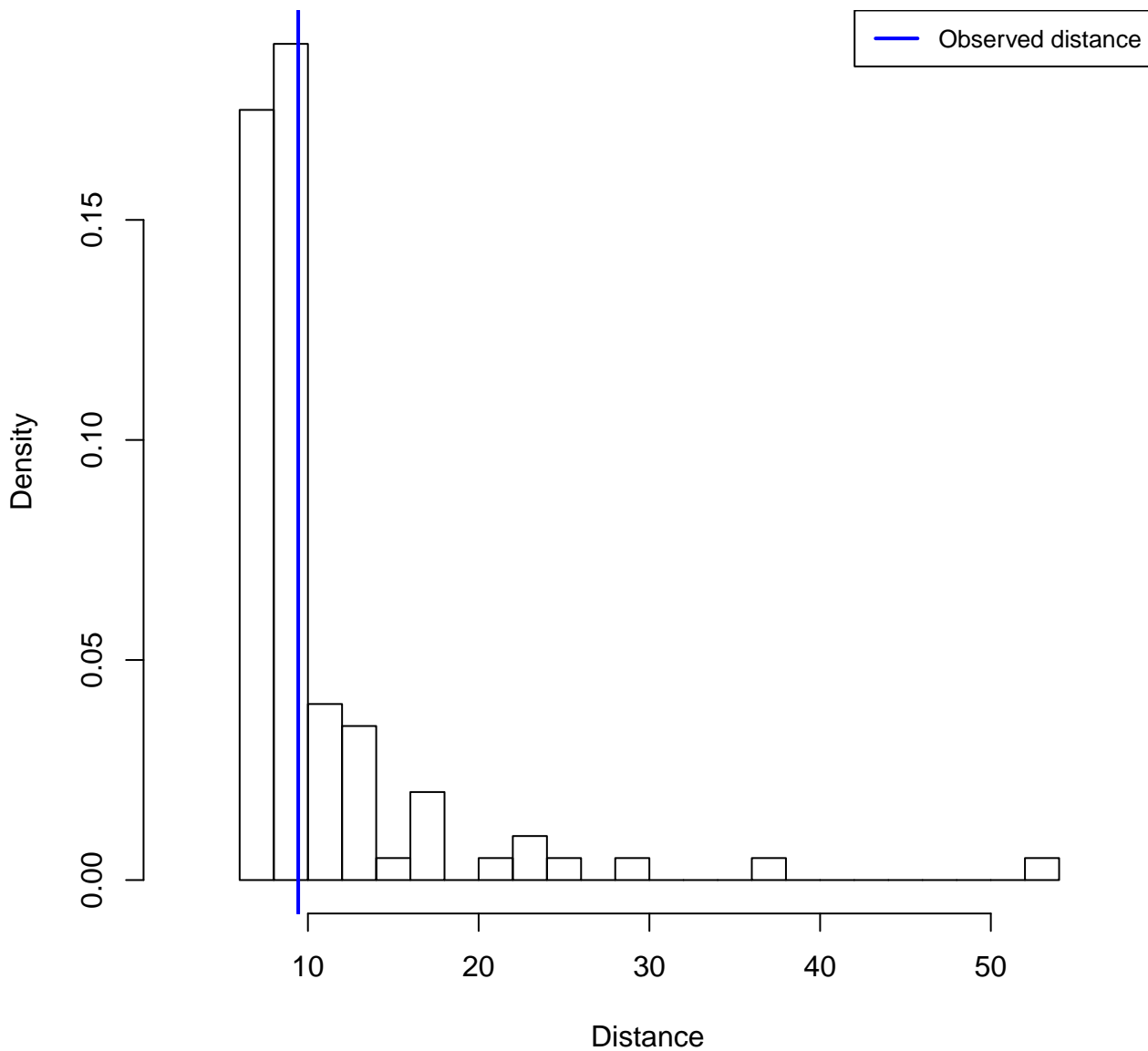

# Castanozoster thoracicus IBD

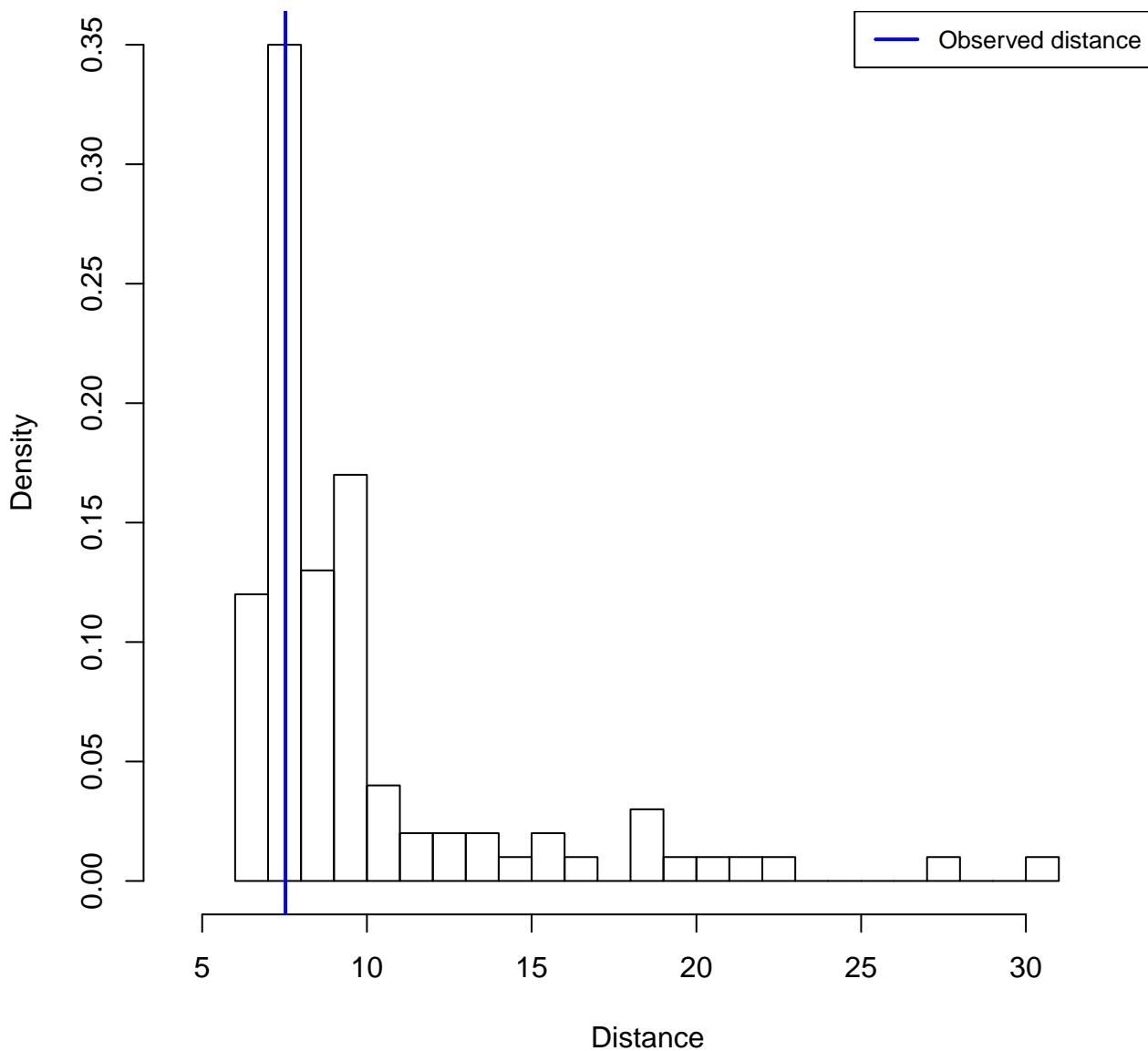

Supplement: Supplementary file 8 — Supplementary Data 5 [file 41467_2021_26537_MOESM8_ESM.gz › PCAs/thoracica_N_PCA.pdf]

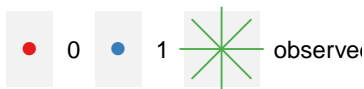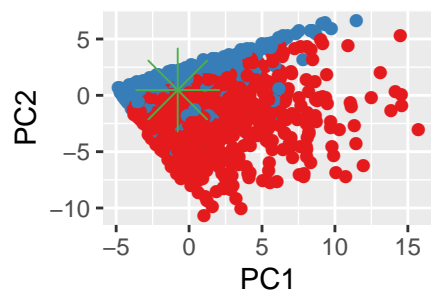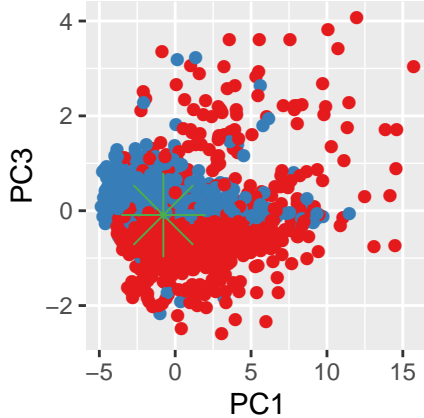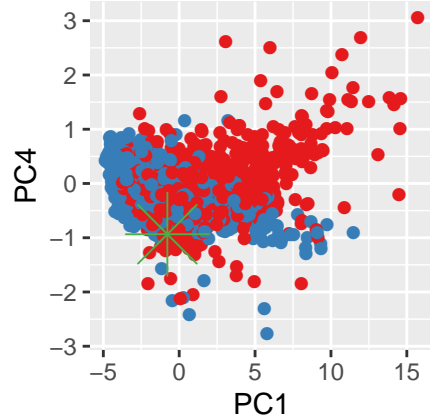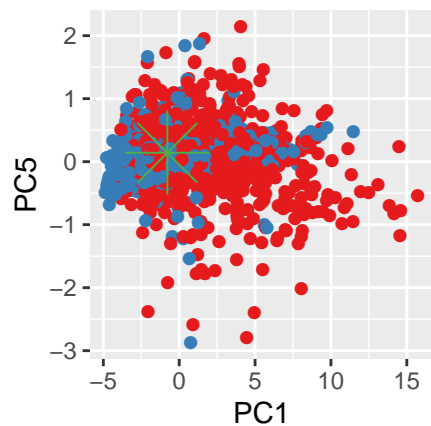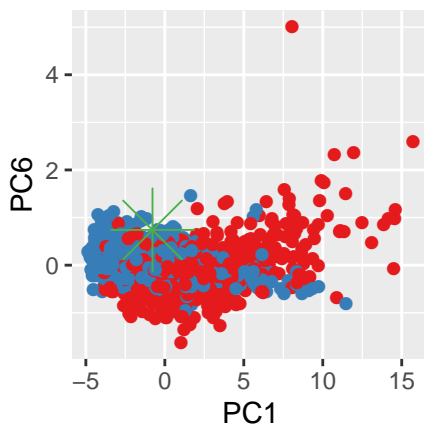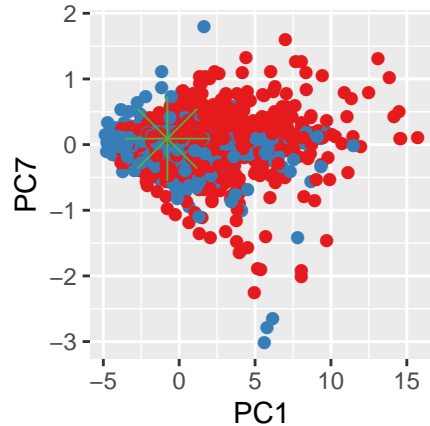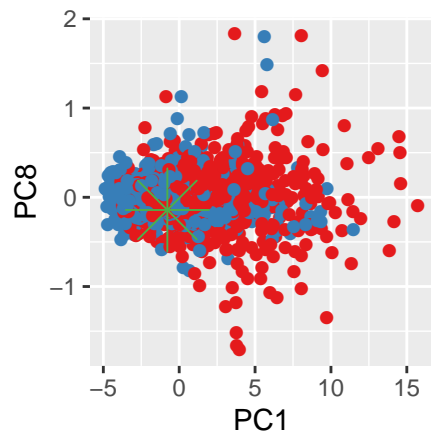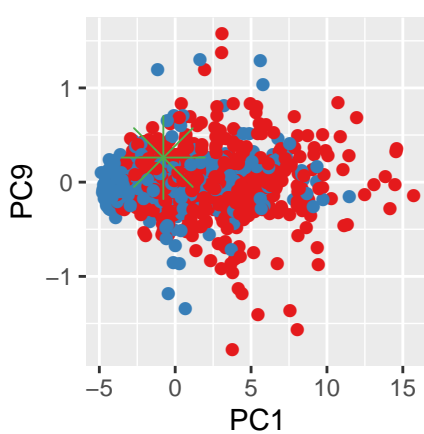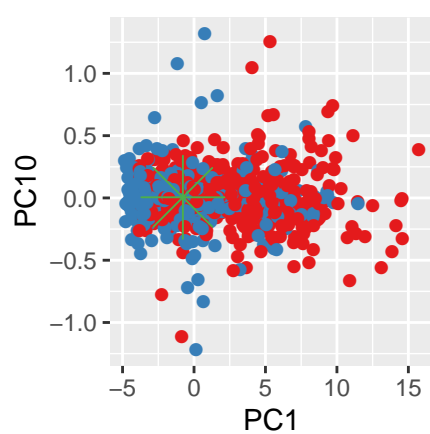

# Synallaxis cinerascens Island

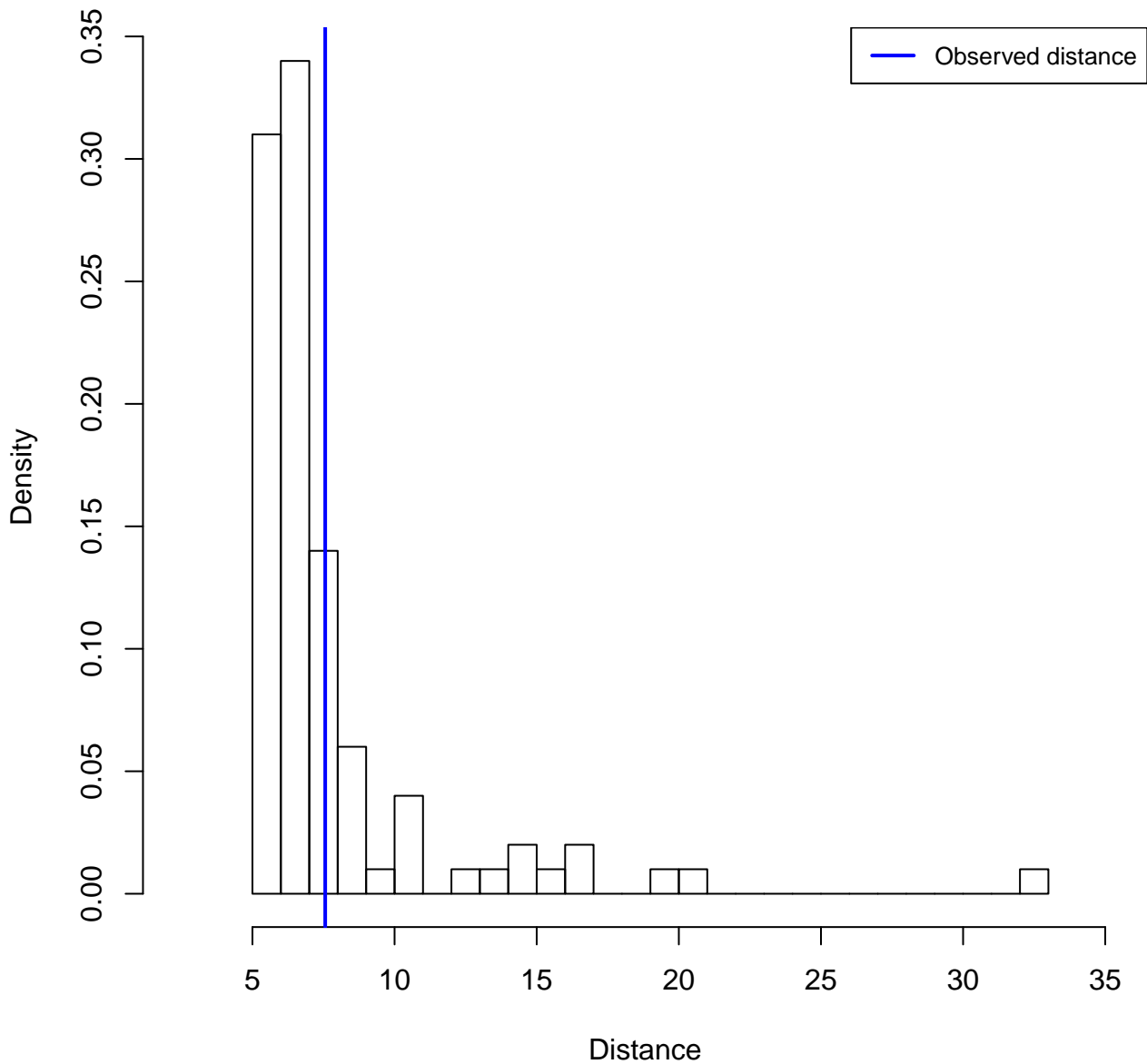

# Synallaxis cinerascens IBD

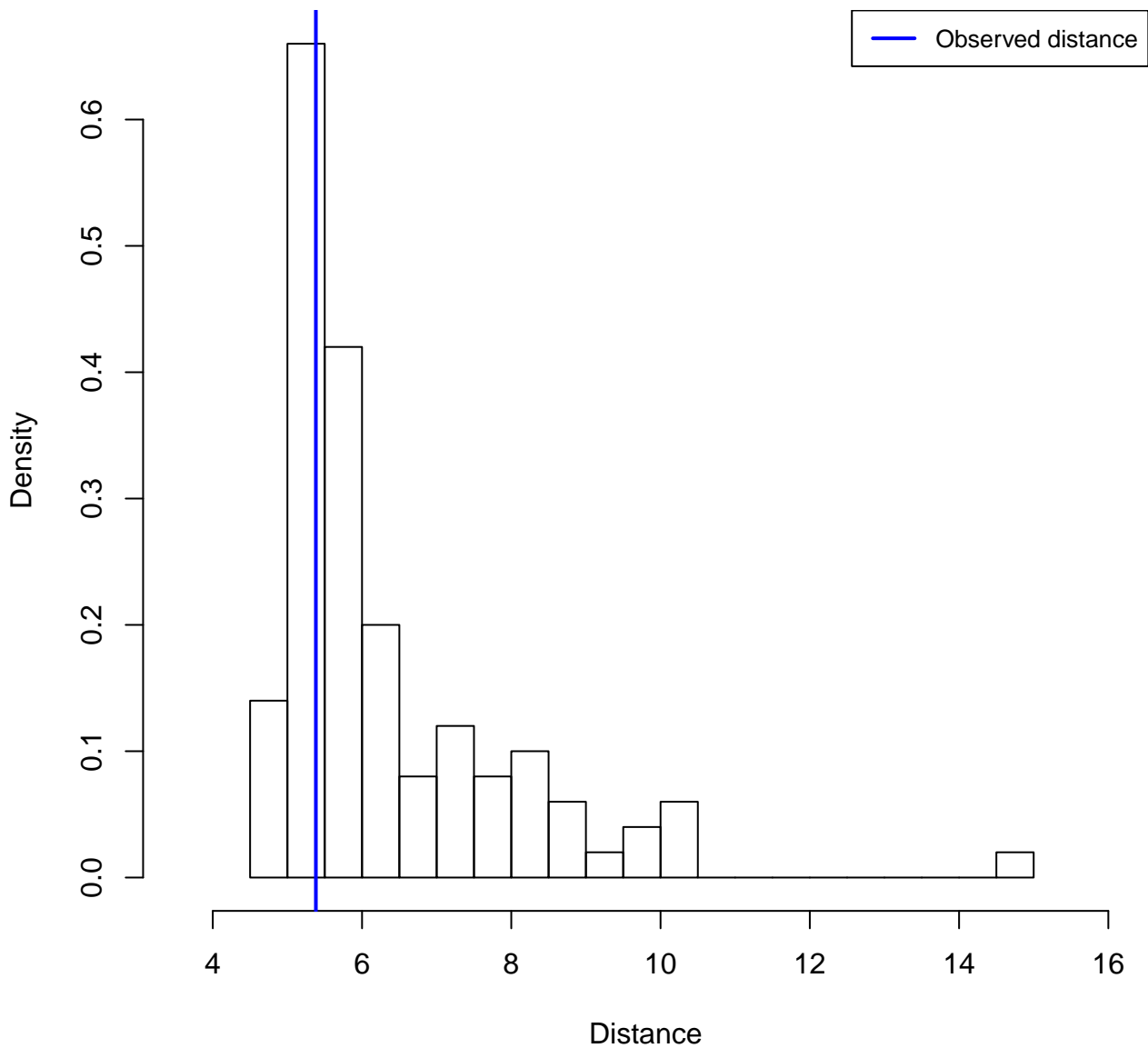

Supplement: Supplementary file 8 — Supplementary Data 5 [file 41467_2021_26537_MOESM8_ESM.gz › PCAs/cinerascens_S_PCA.pdf]

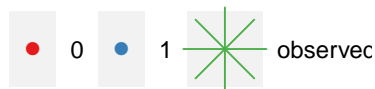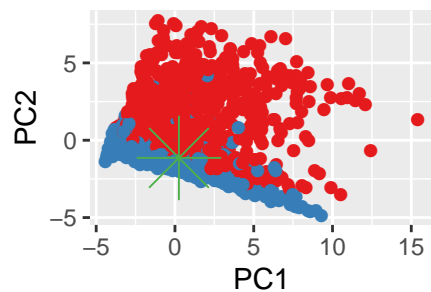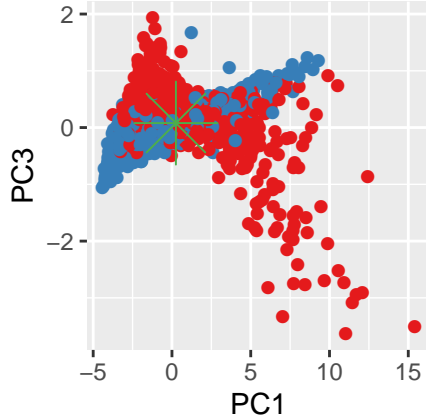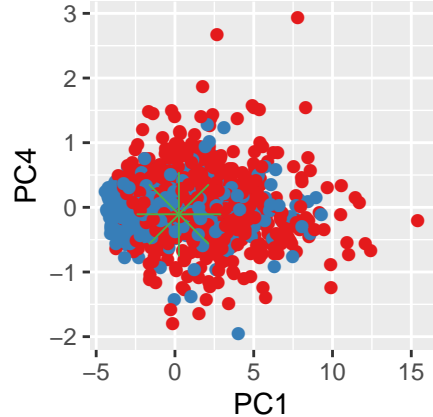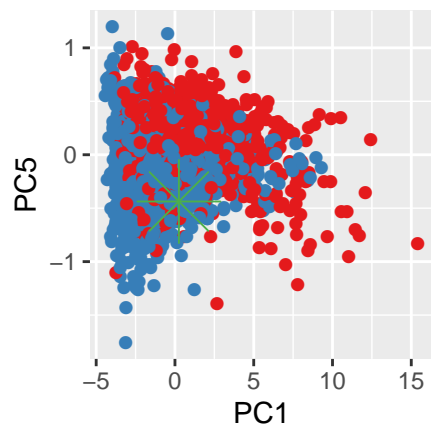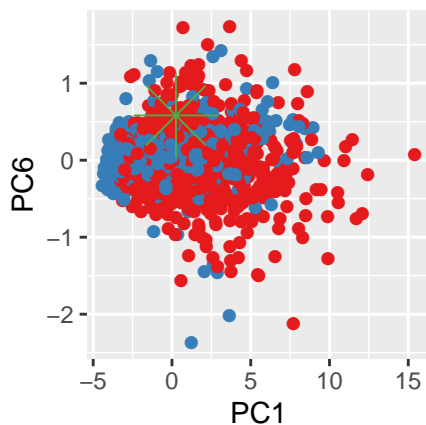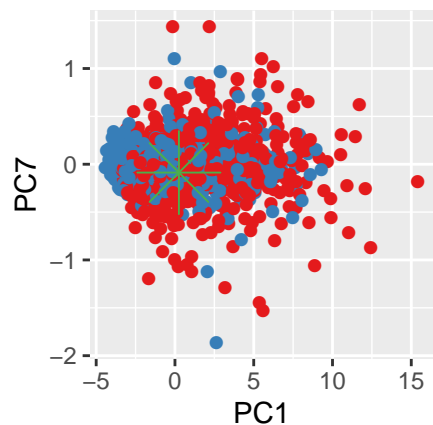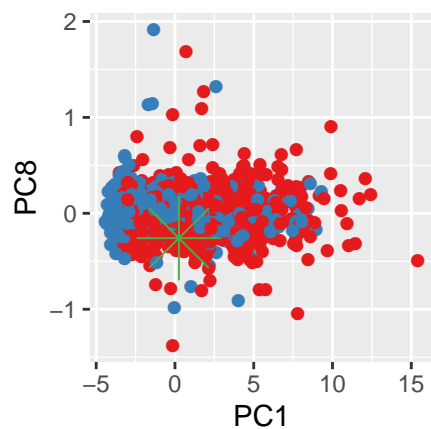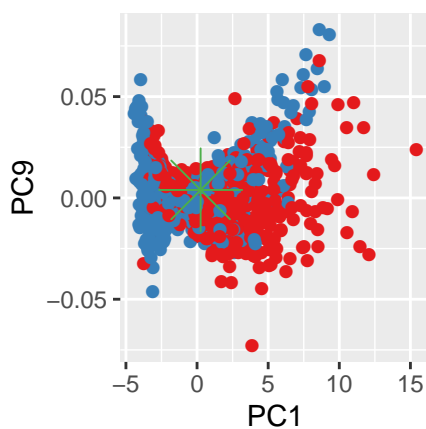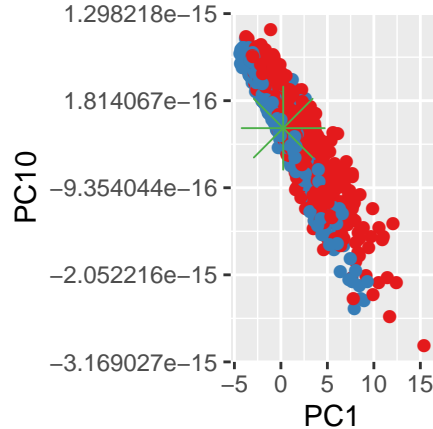

# Muscipipra vetula Island

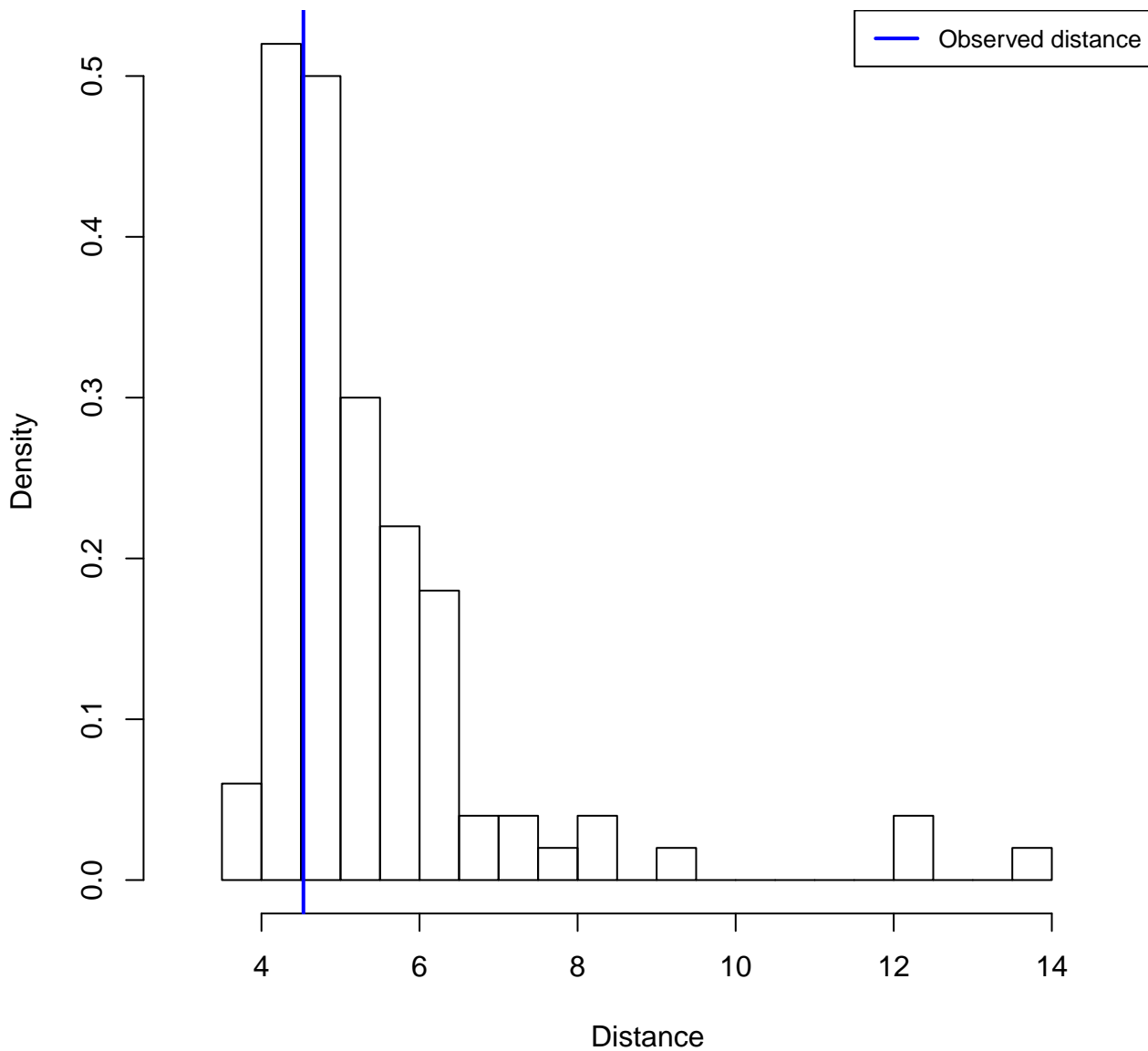

# Muscipipra vetula IBD

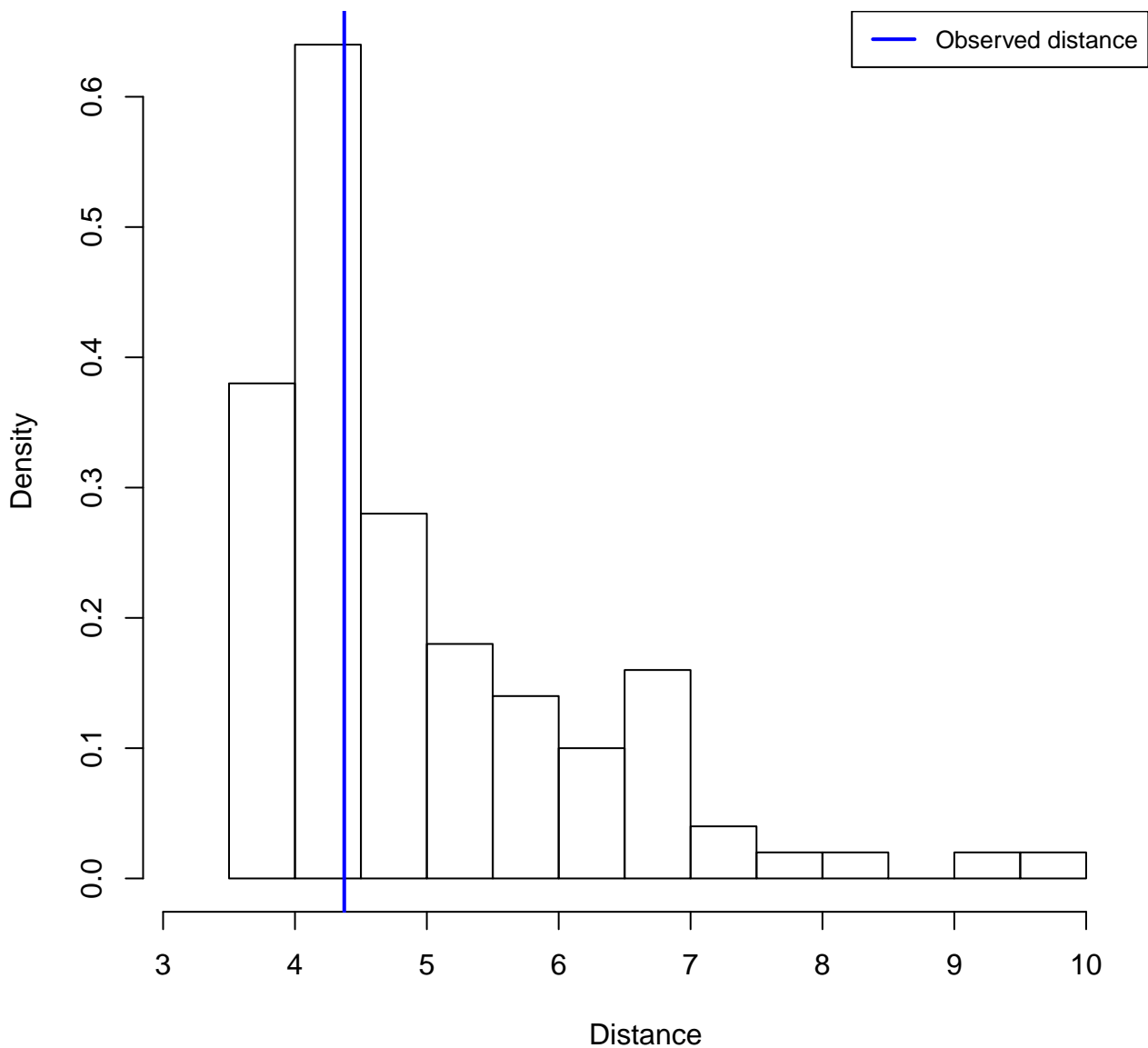

Supplement: Supplementary file 8 — Supplementary Data 5 [file 41467_2021_26537_MOESM8_ESM.gz › PCAs/vetula_S_PCA.pdf]

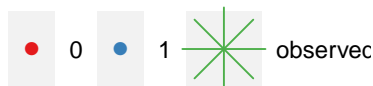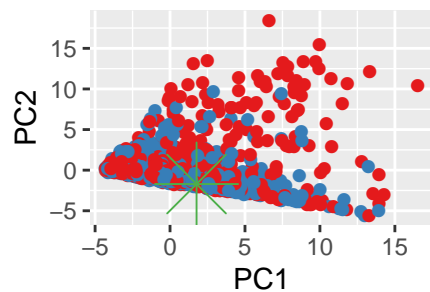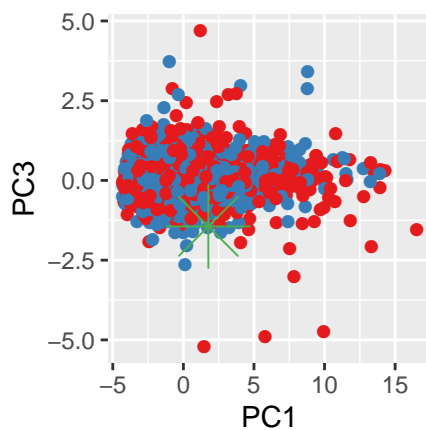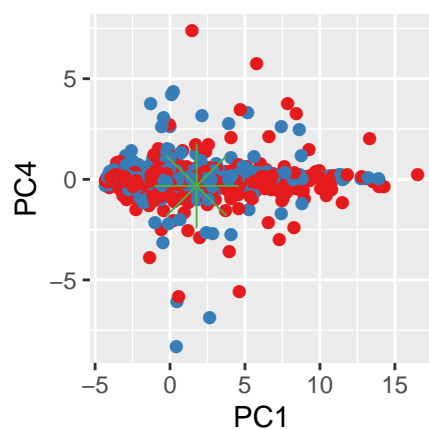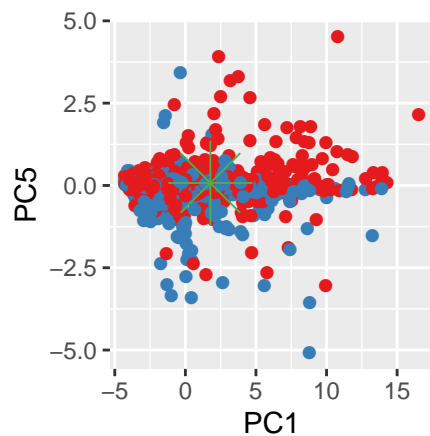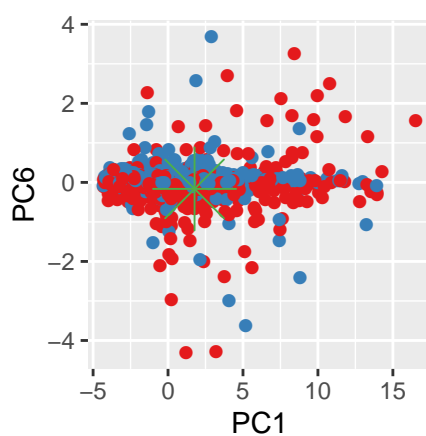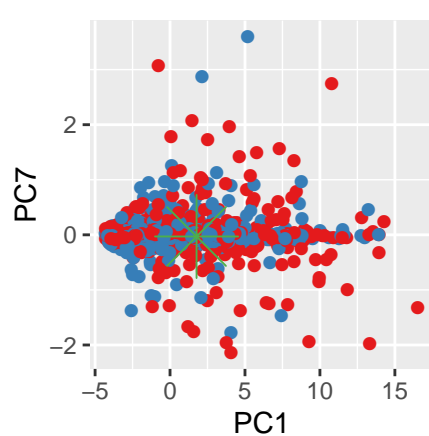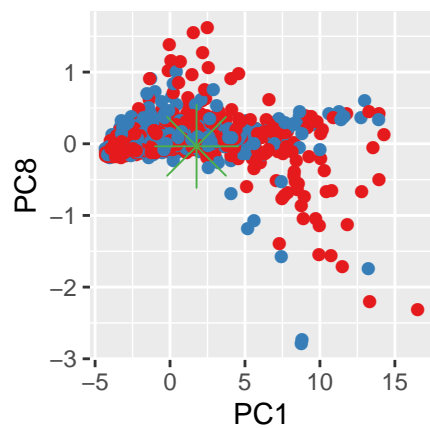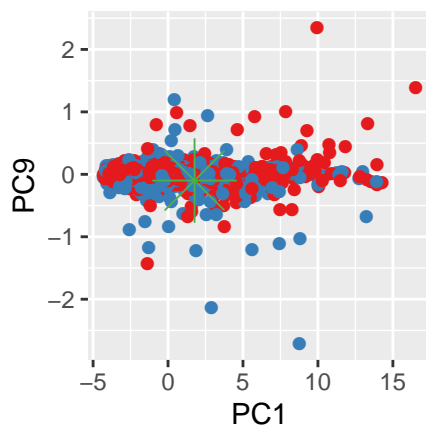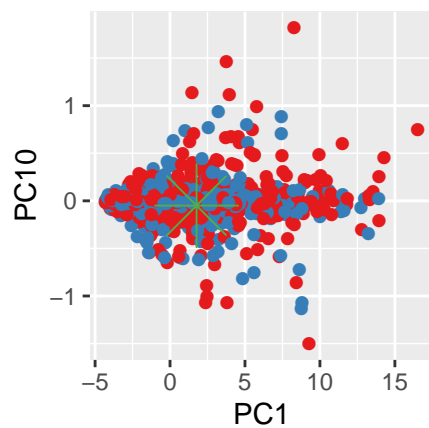

# Tangara desmaresti Island

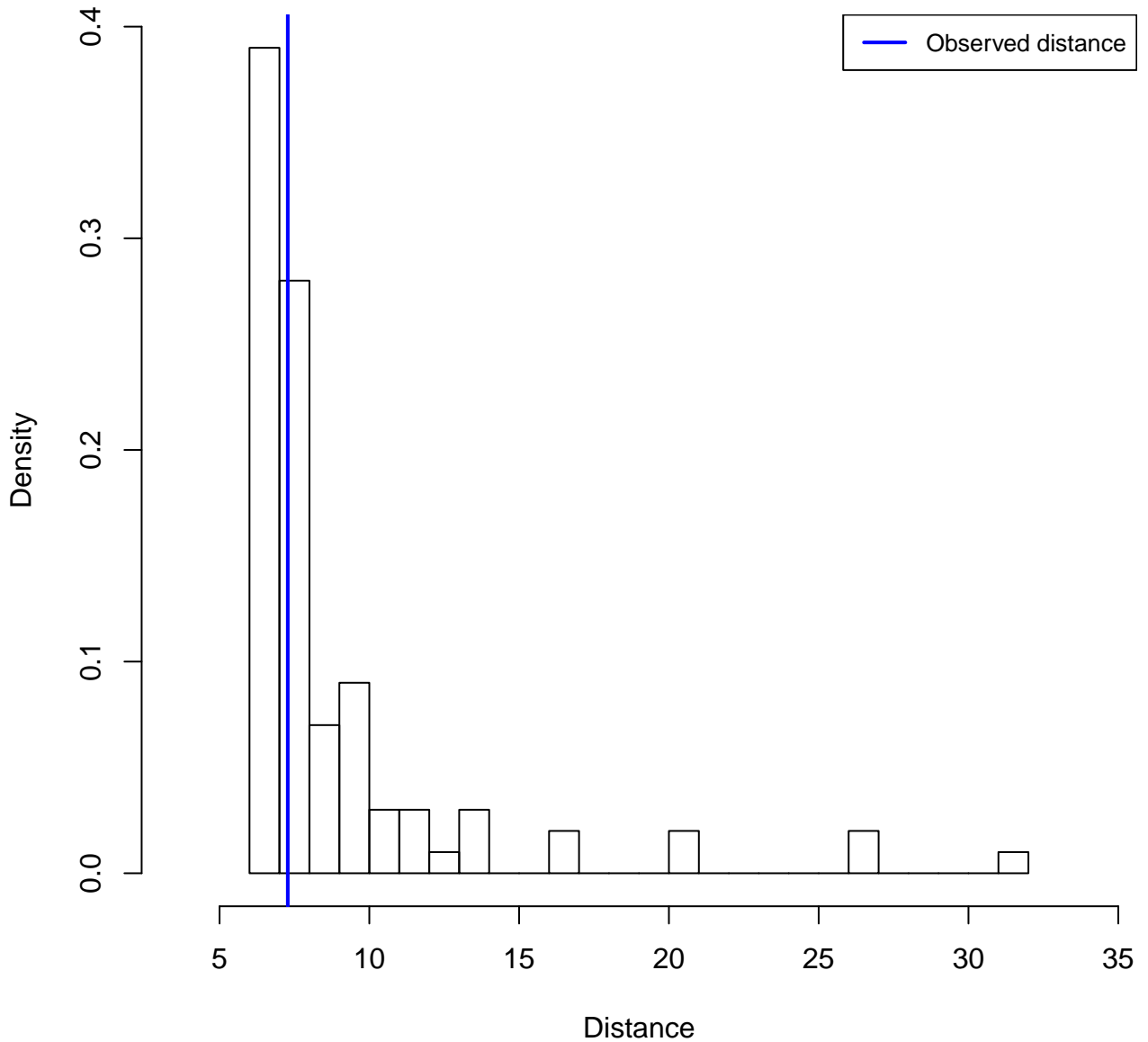

# Tangara desmaresti IBD

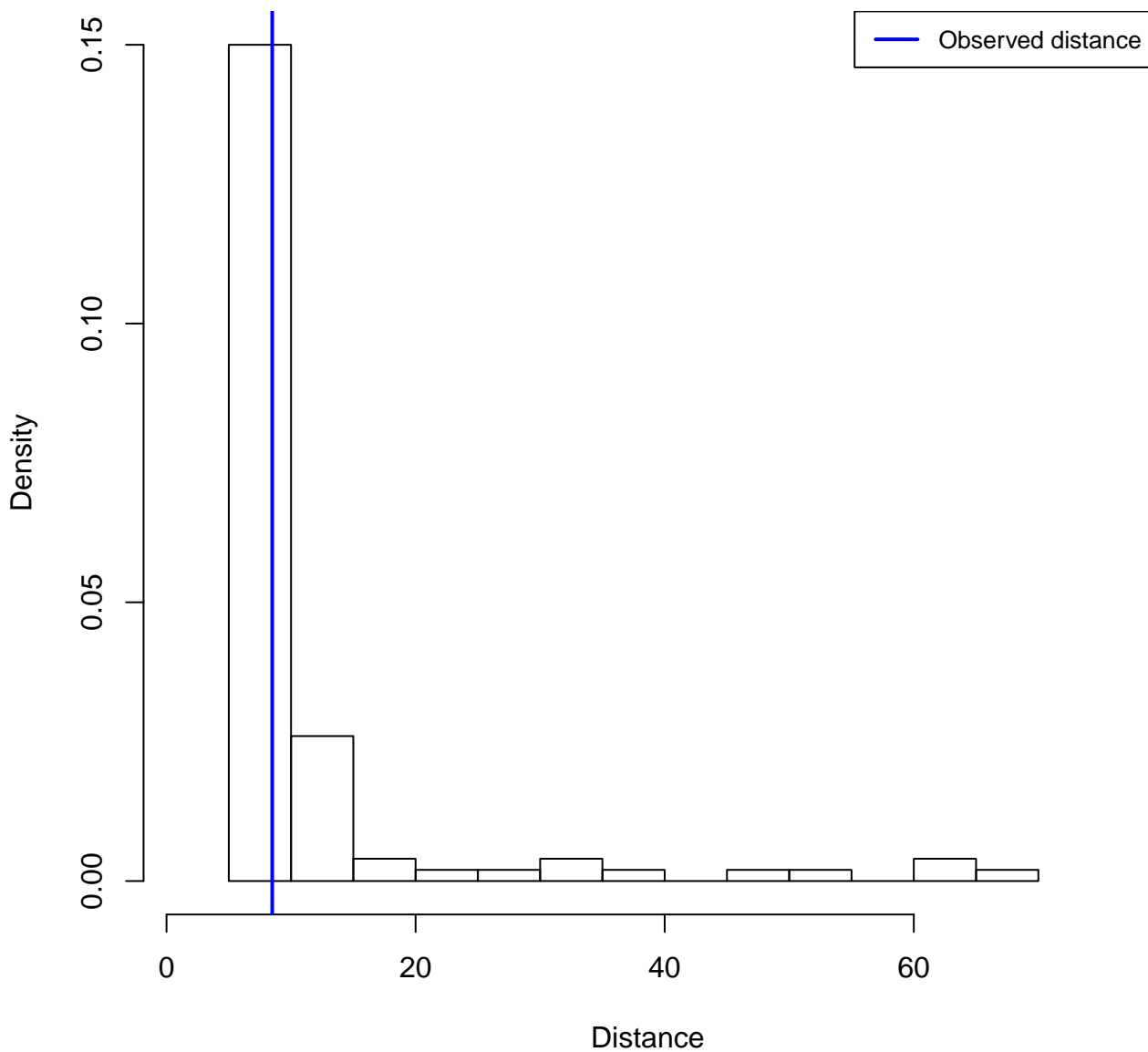

Supplement: Supplementary file 8 — Supplementary Data 5 [file 41467_2021_26537_MOESM8_ESM.gz › PCAs/desmaresti_N_PCA.pdf]

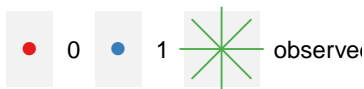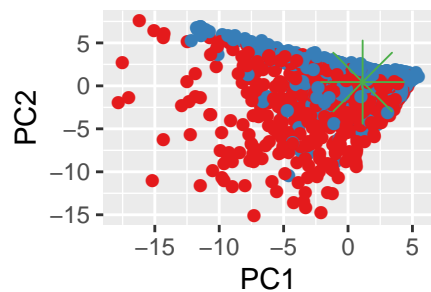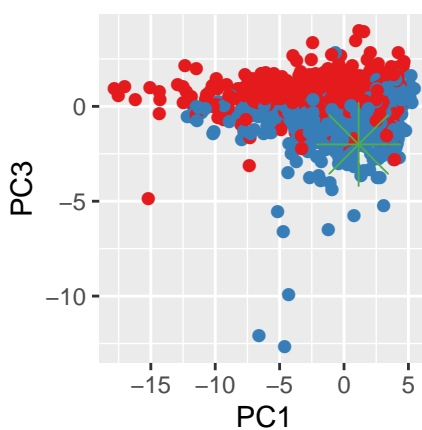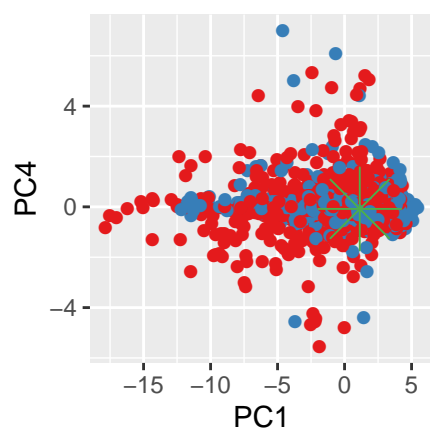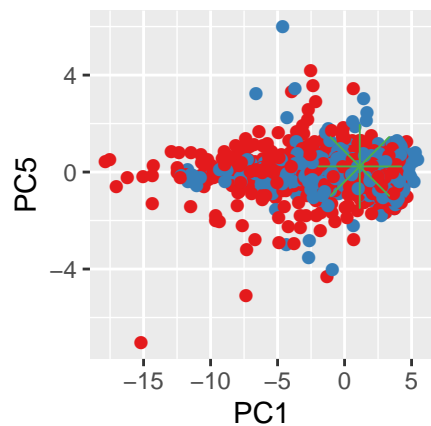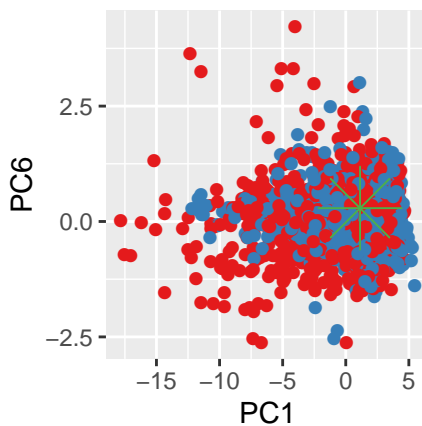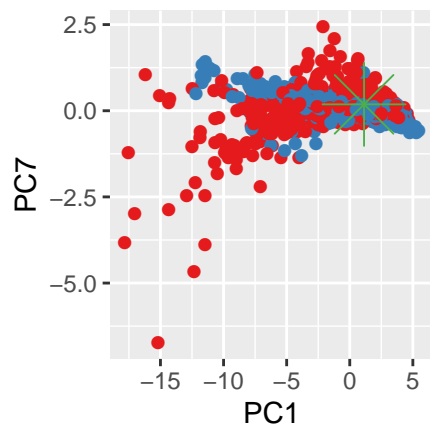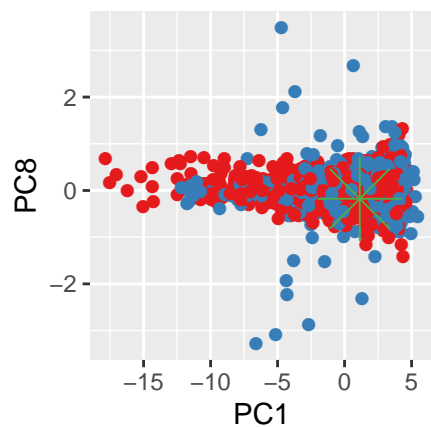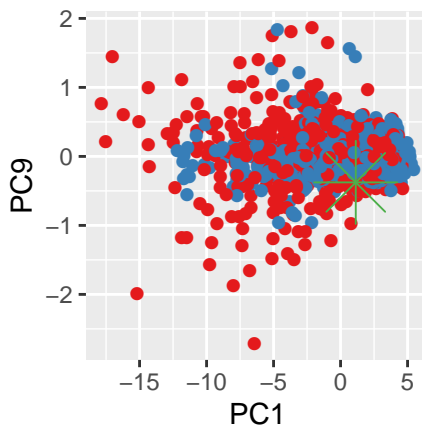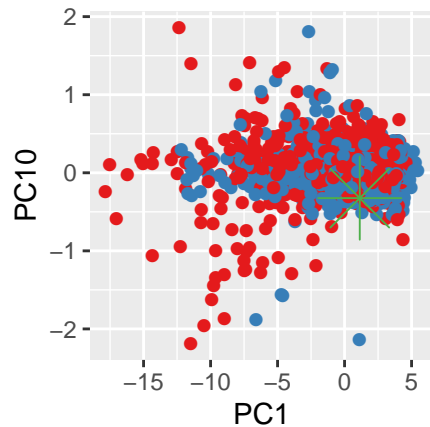

# Muscipipra vetula Island

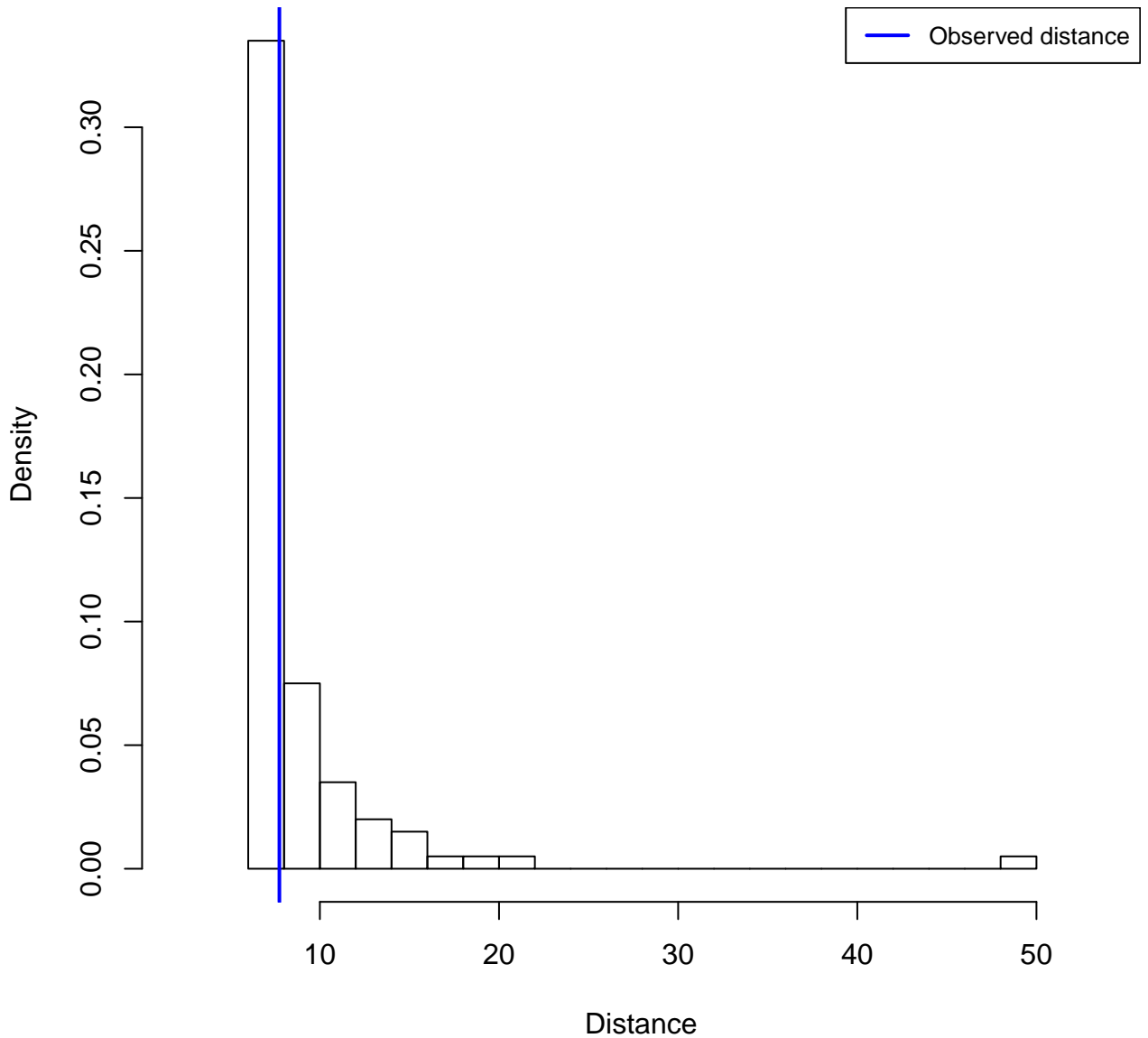

# Muscipipra vetula IBD

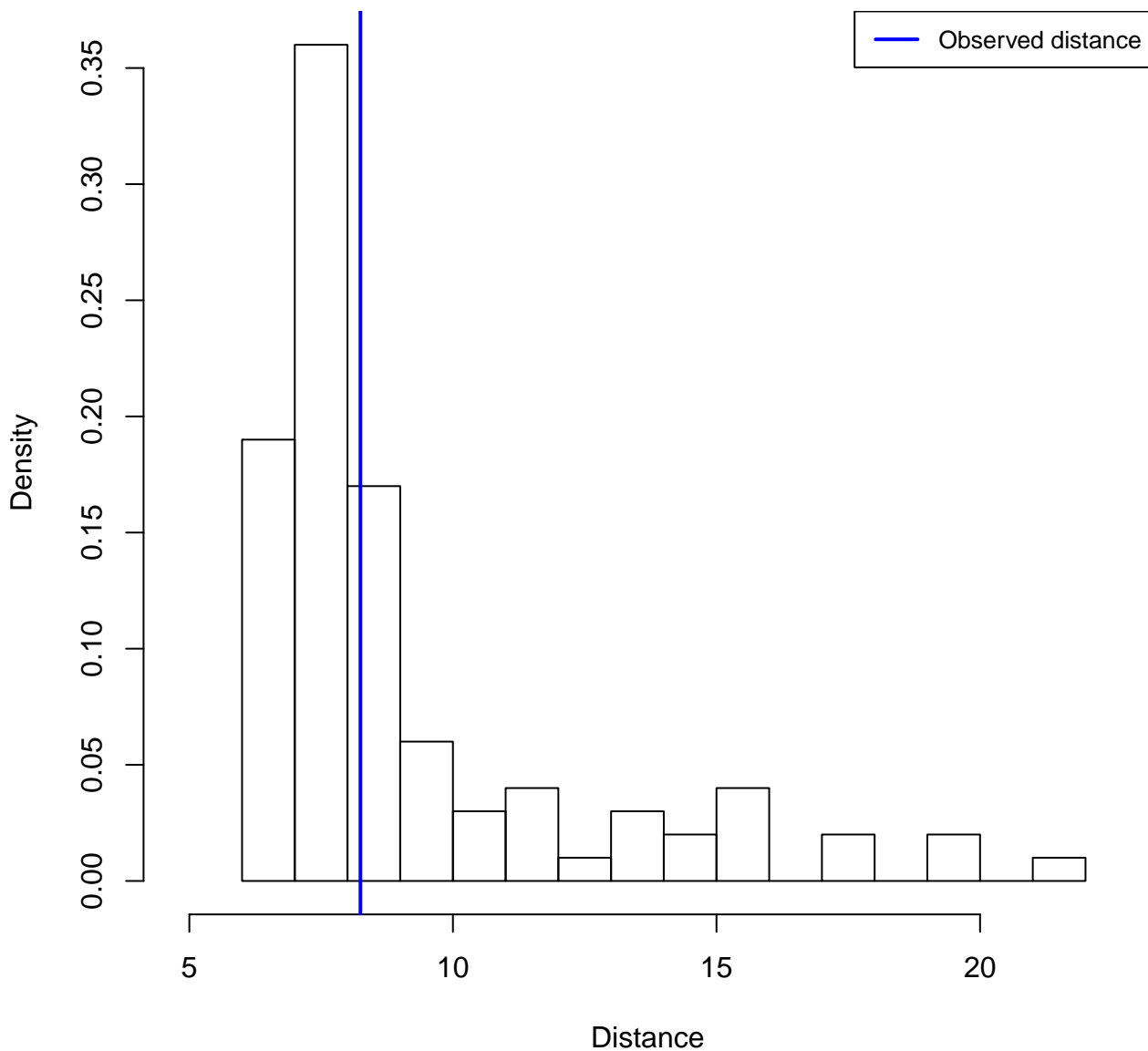

Supplement: Supplementary file 8 — Supplementary Data 5 [file 41467_2021_26537_MOESM8_ESM.gz › PCAs/vetula_N_PCA.pdf]

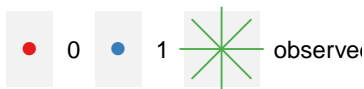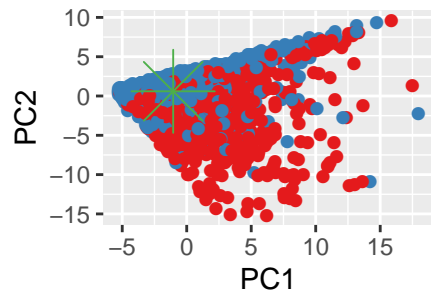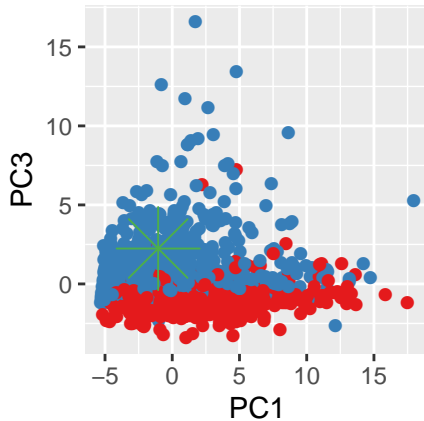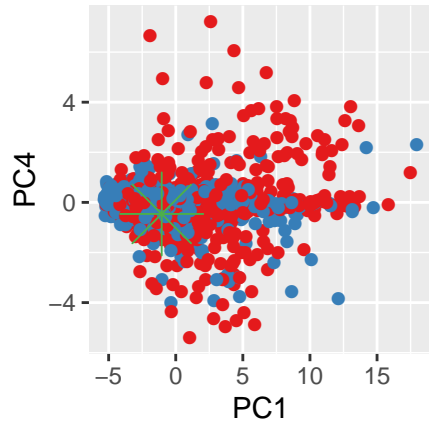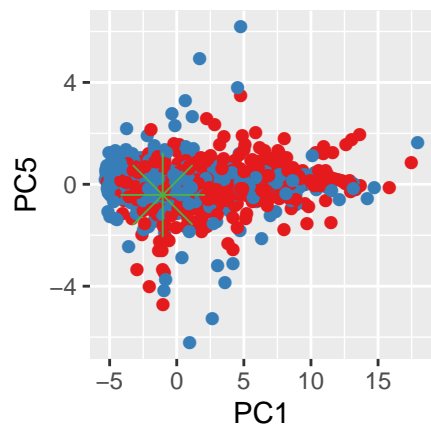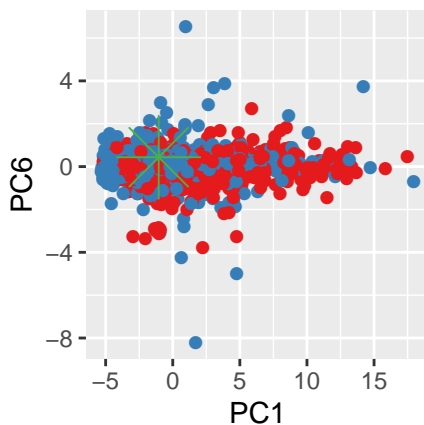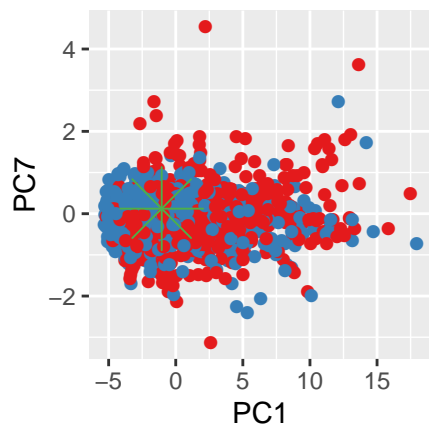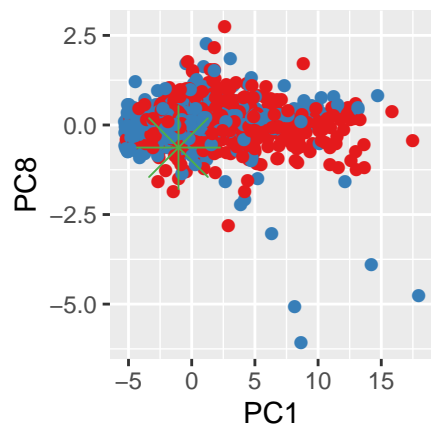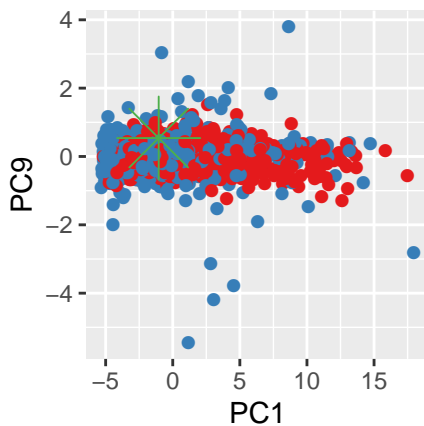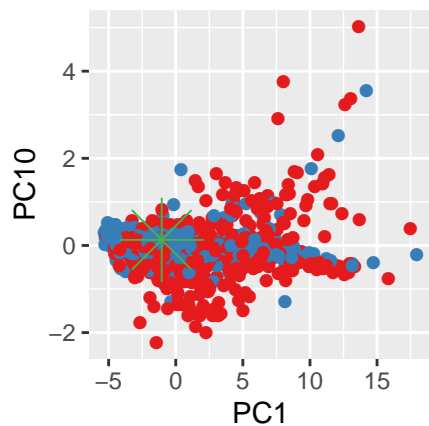

# Synallaxis cinerascens Island

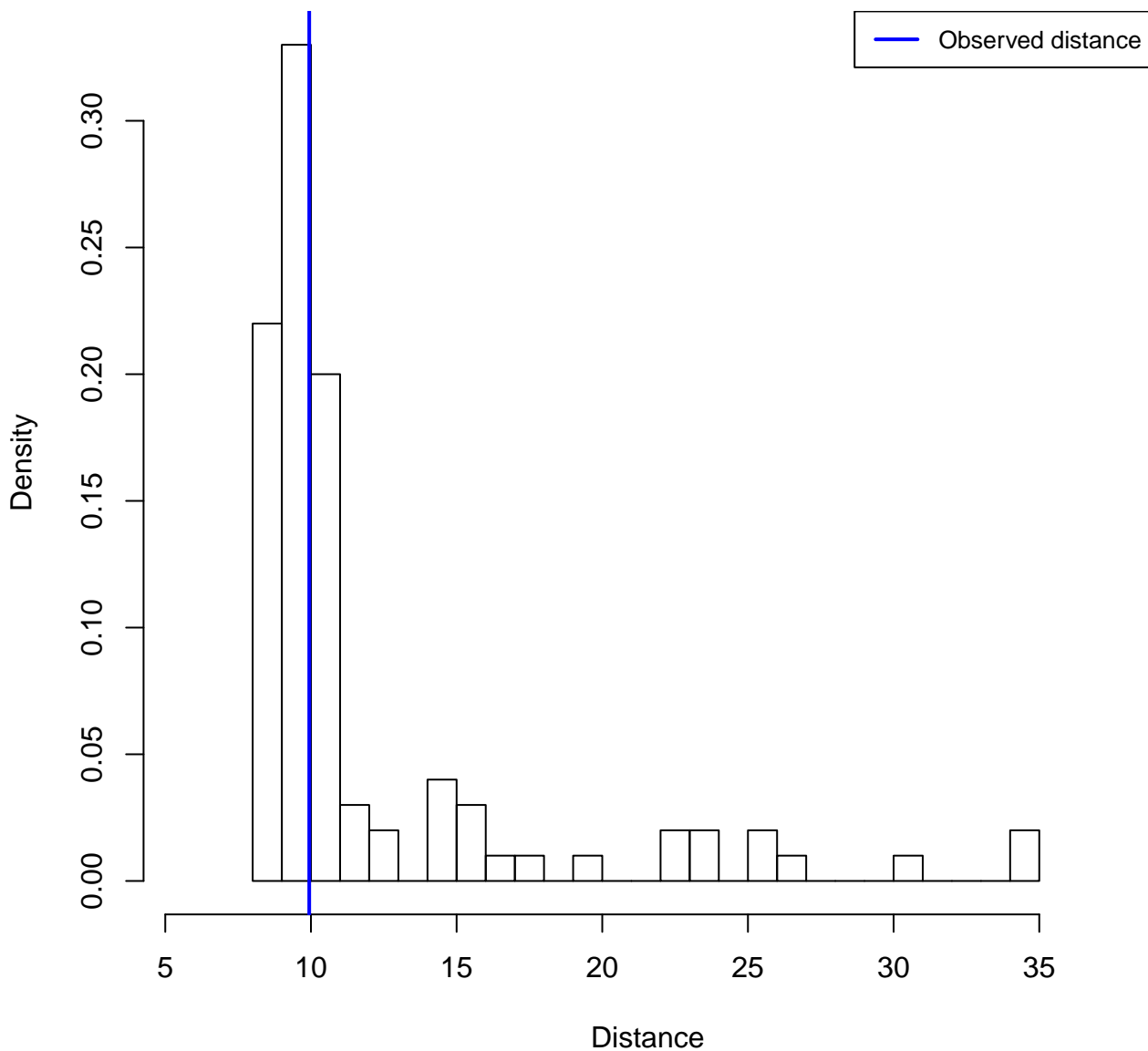

# Synallaxis cinerascens IBD

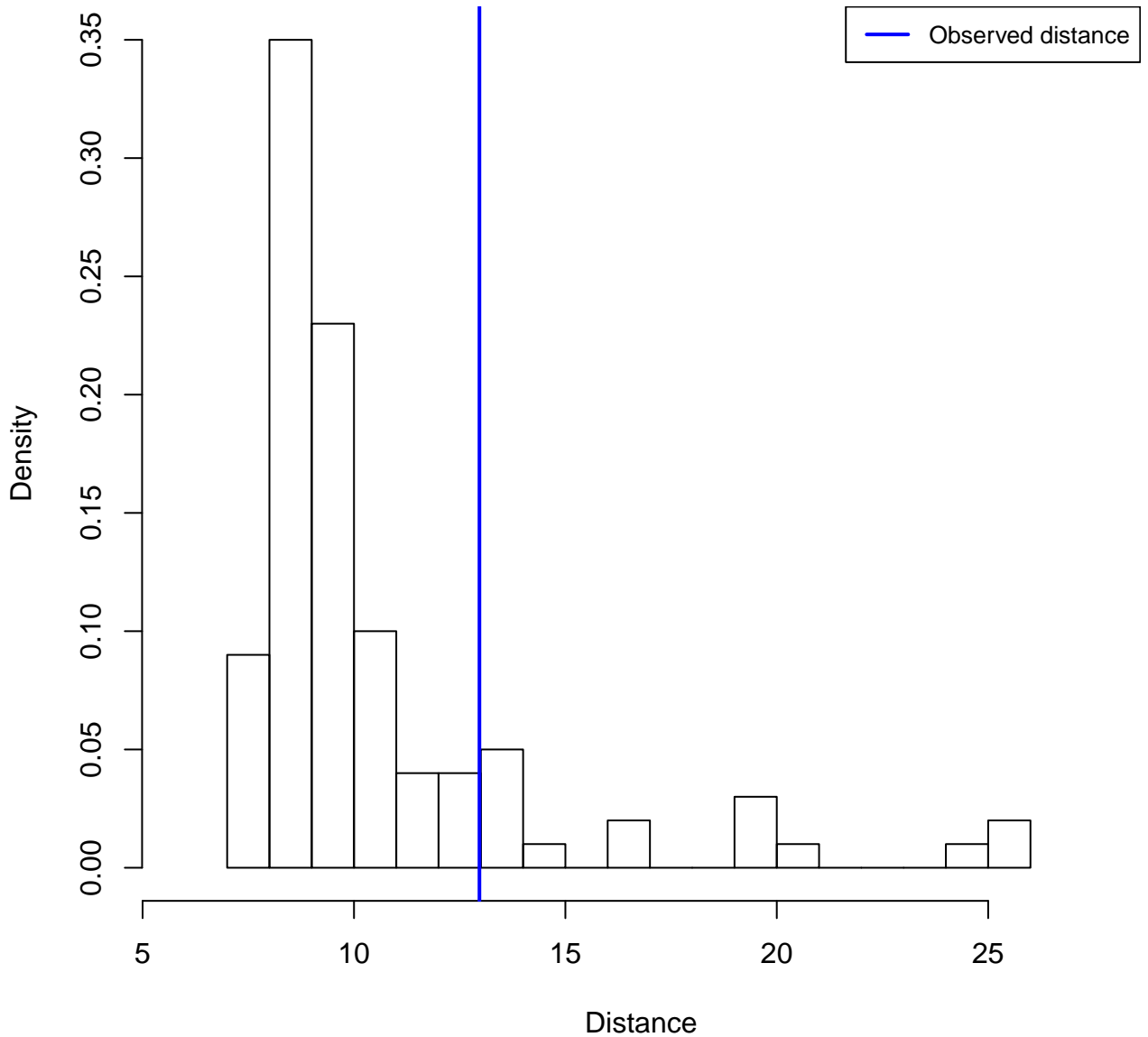

Supplement: Supplementary file 8 — Supplementary Data 5 [file 41467_2021_26537_MOESM8_ESM.gz › PCAs/cinerascens_N_PCA.pdf]

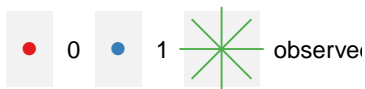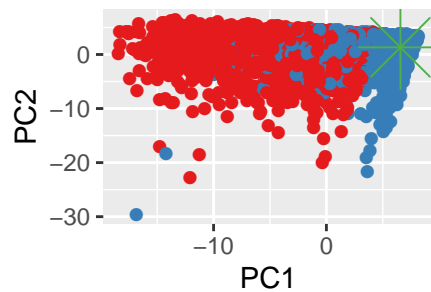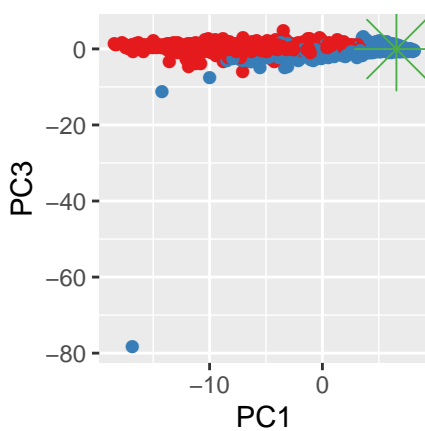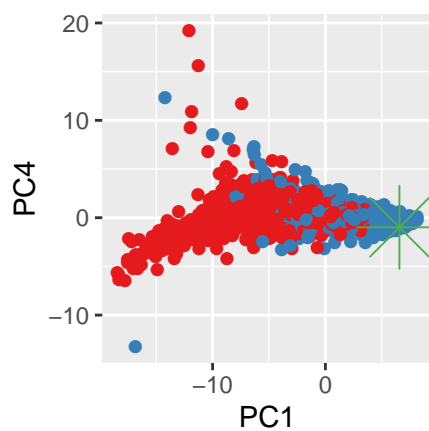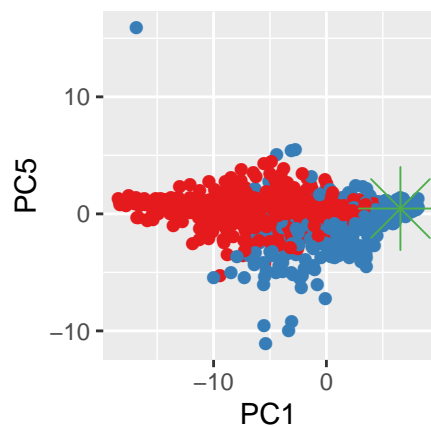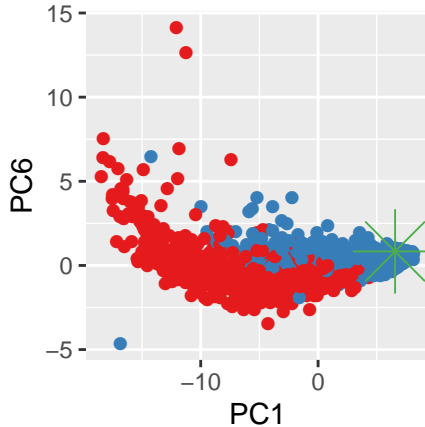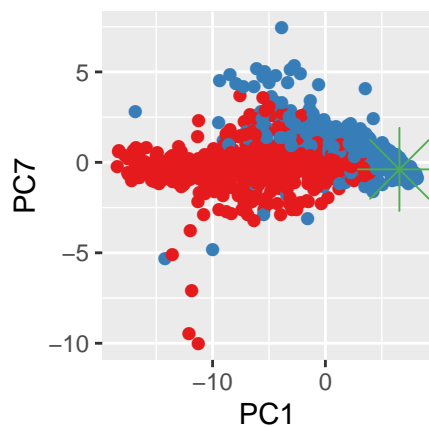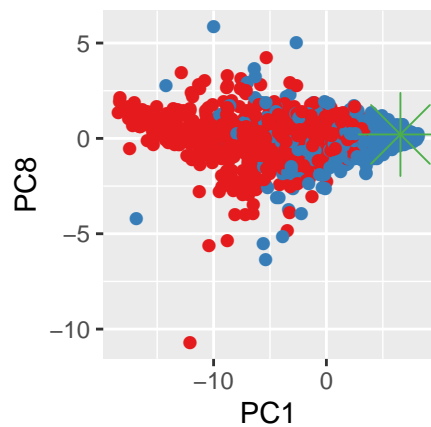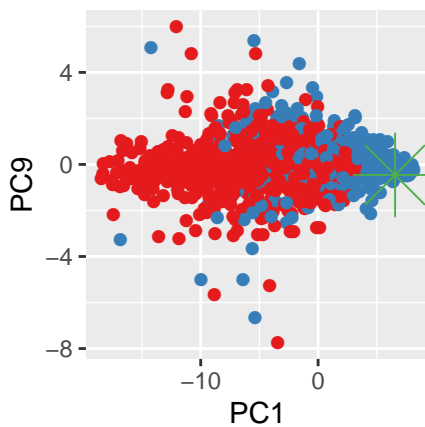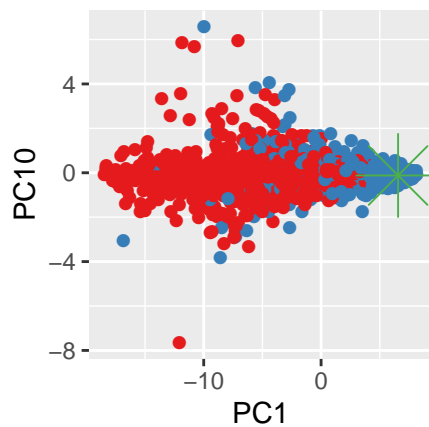

# Phylloscartes ventralis Island

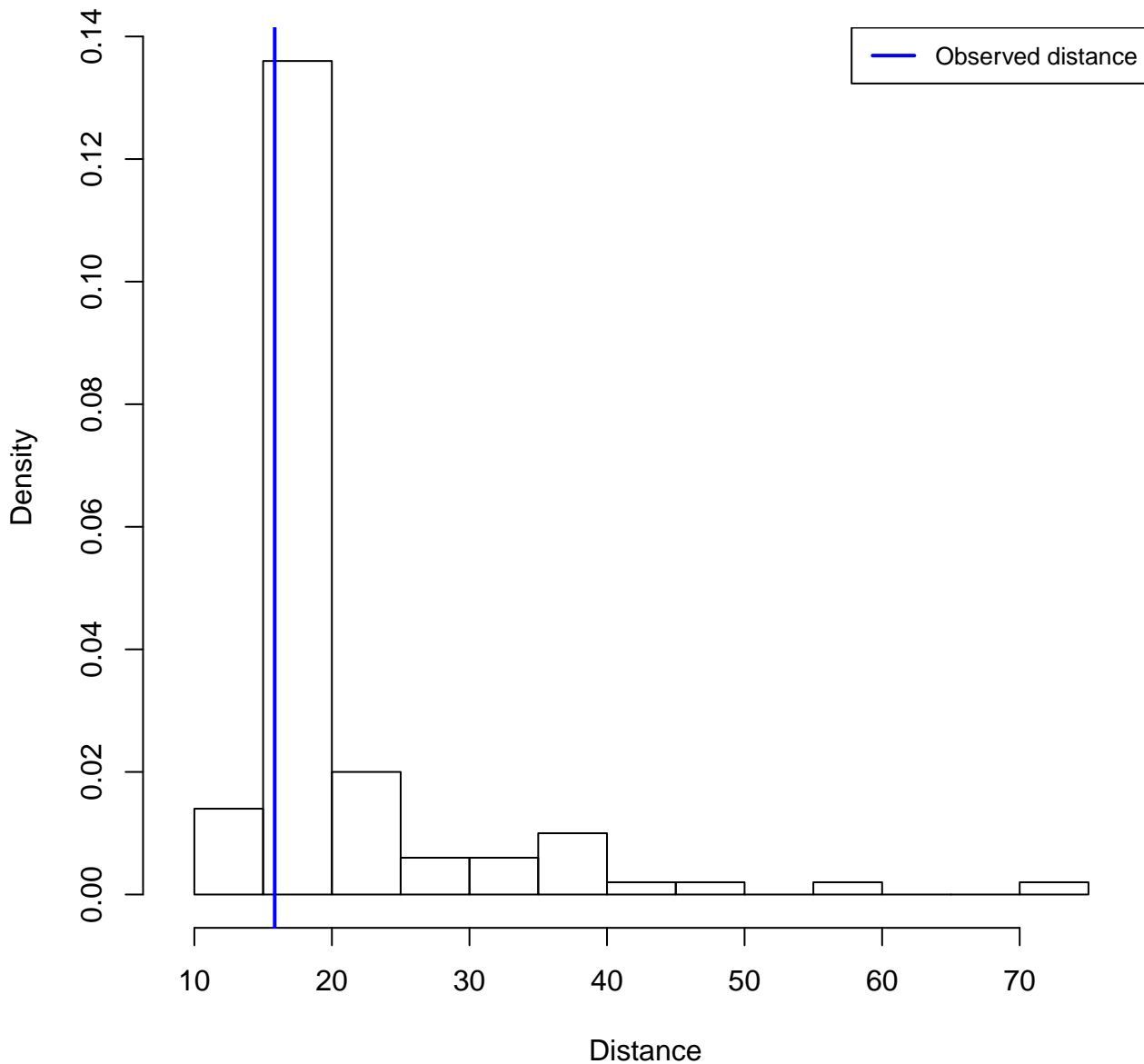

# Phylloscartes ventralis IBD

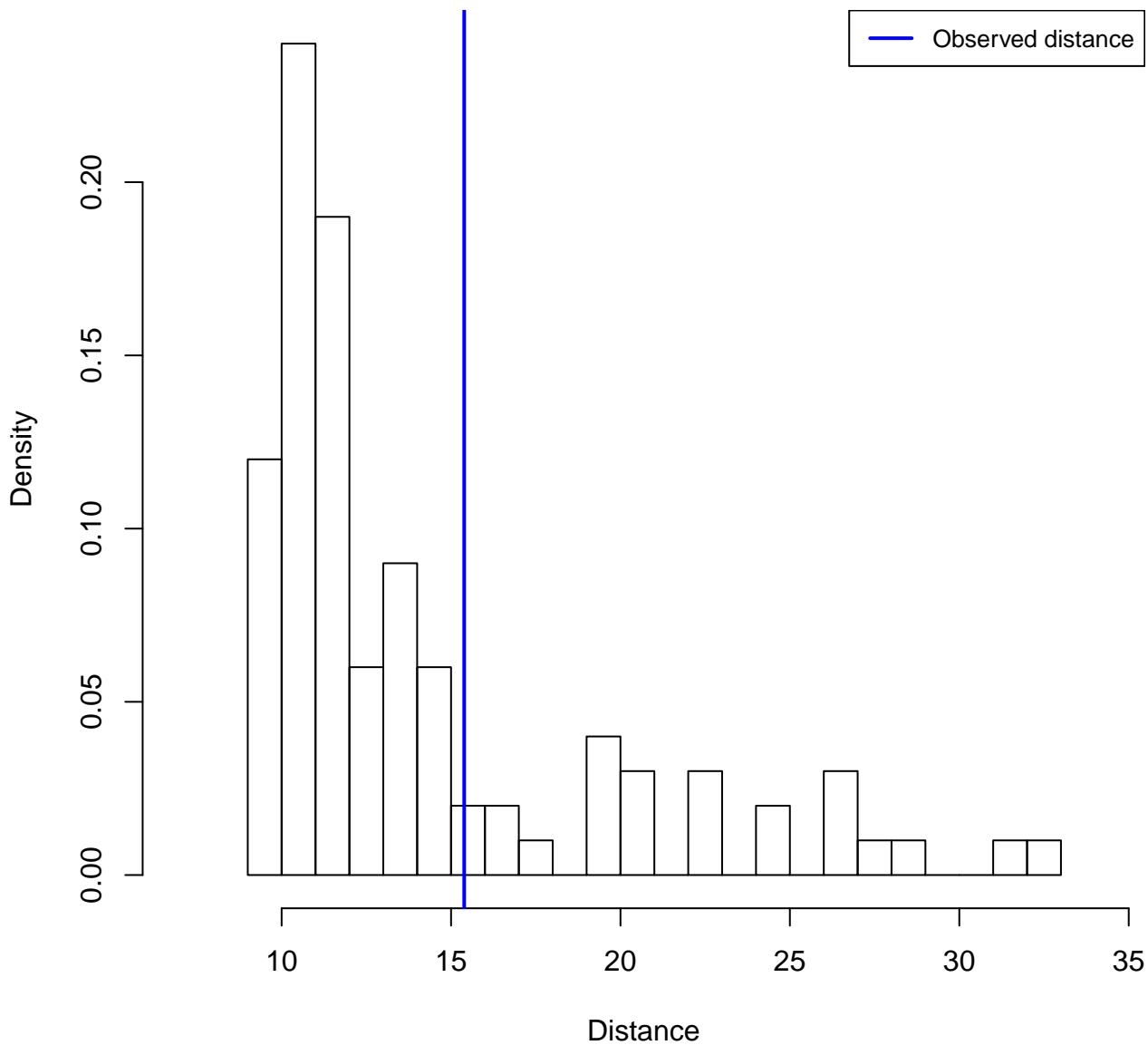

Supplement: Supplementary file 8 — Supplementary Data 5 [file 41467_2021_26537_MOESM8_ESM.gz › PCAs/ventralis_S_PCA.pdf]

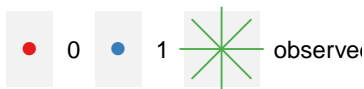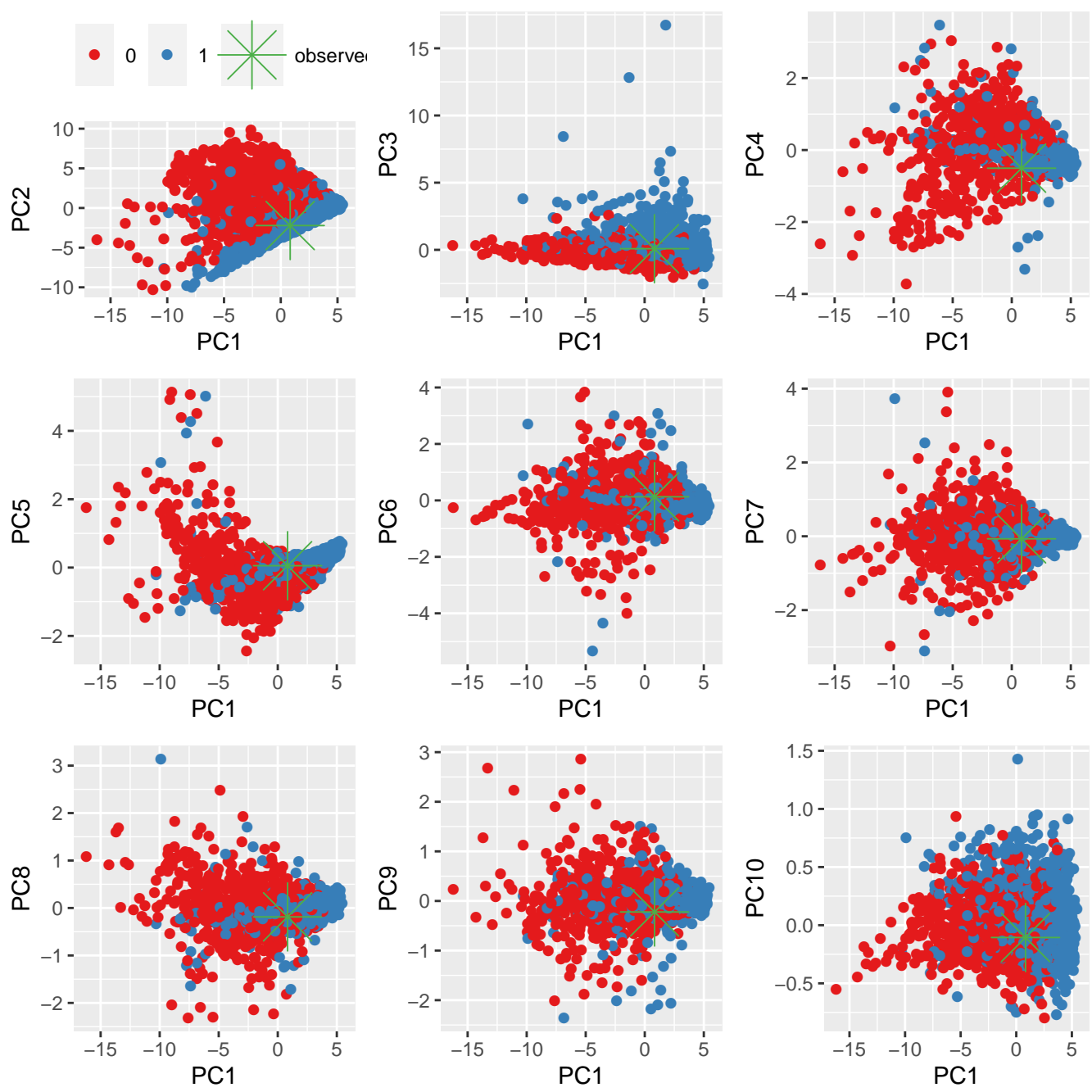

# Saltator maxillosus Island

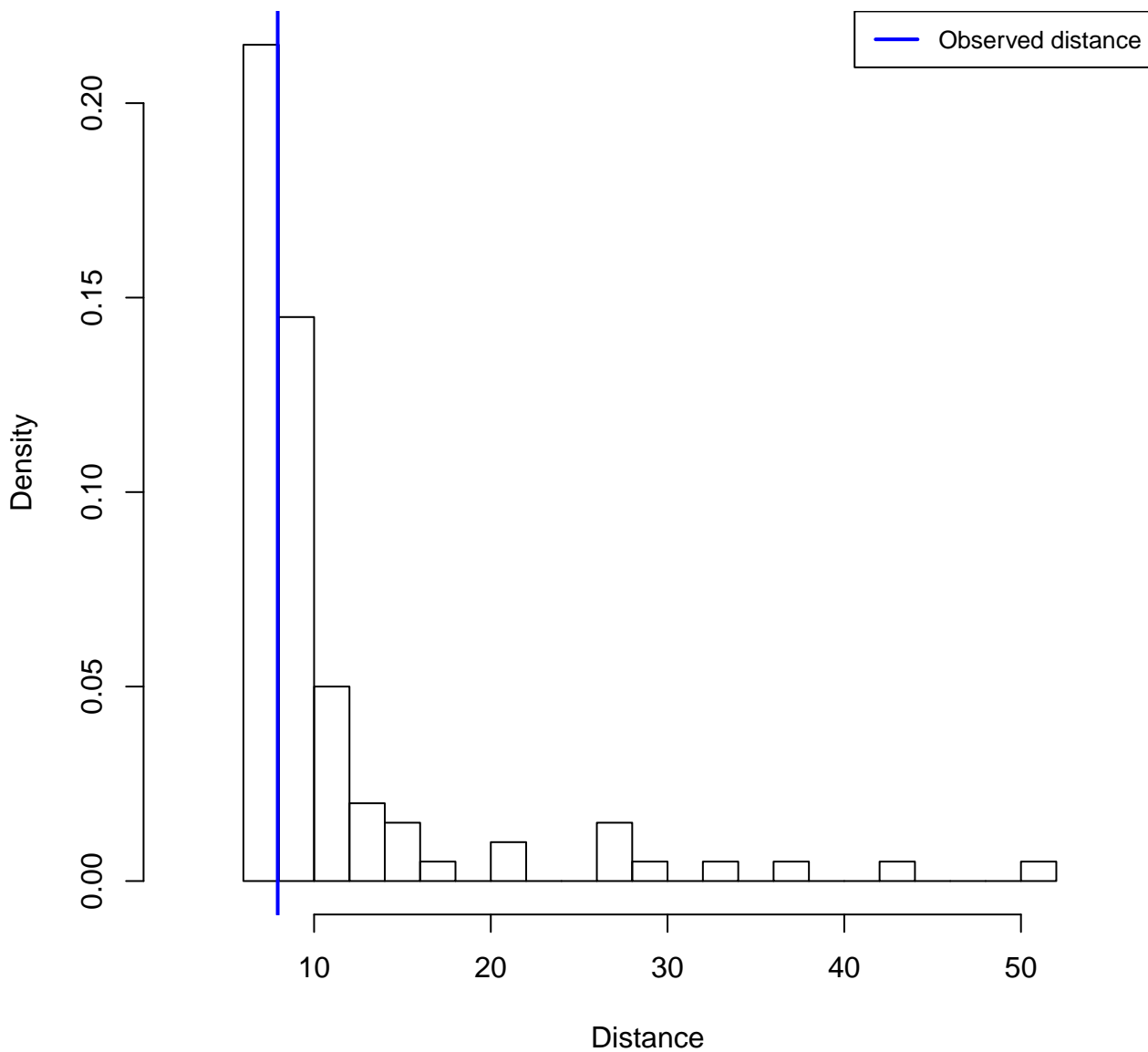

# Saltator maxillosus IBD

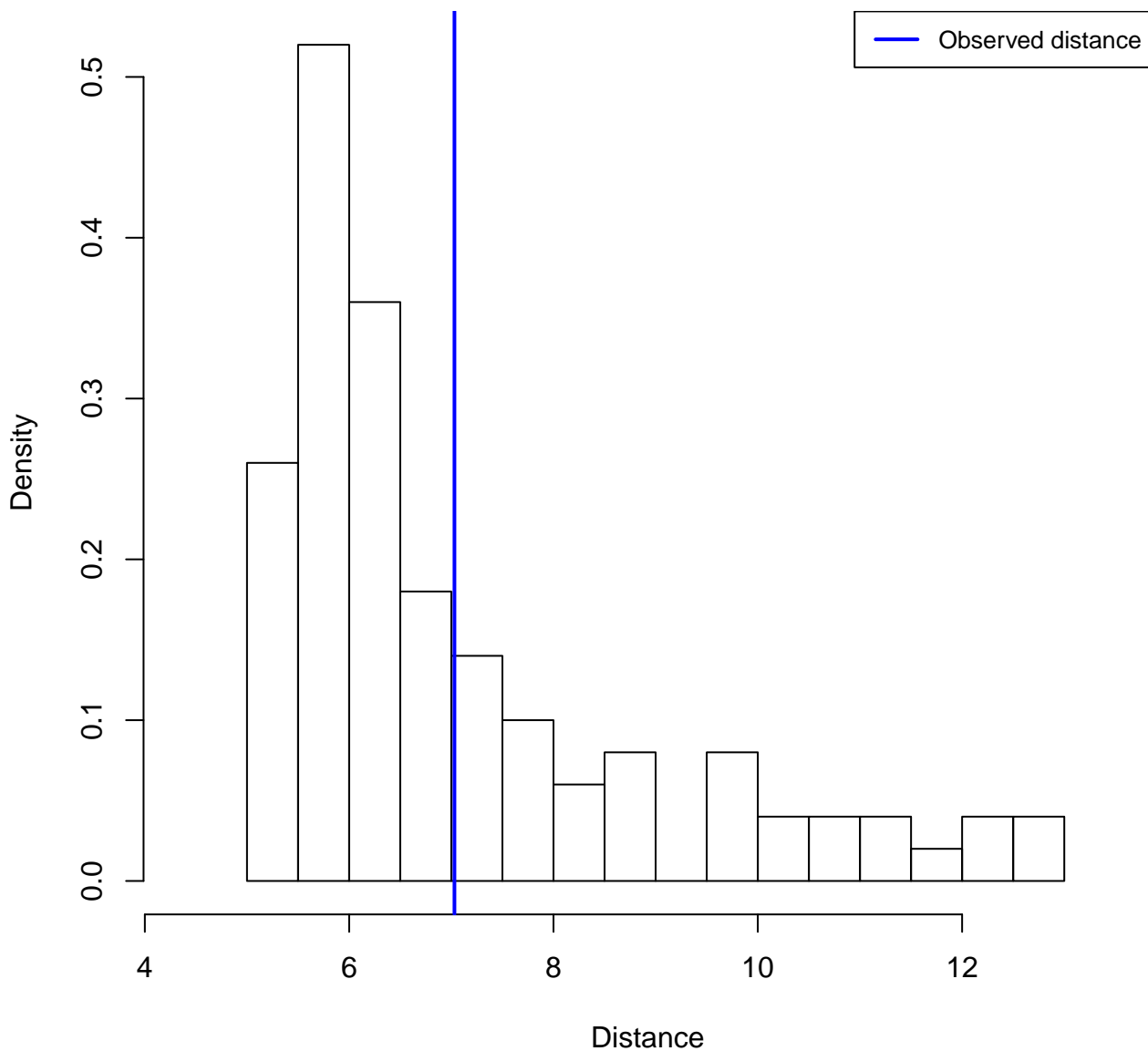

Supplement: Supplementary file 8 — Supplementary Data 5 [file 41467_2021_26537_MOESM8_ESM.gz › PCAs/maxillosus_S_PCA.pdf]
