# Supplementary material for: Microevolutionary dynamics show tropical valleys are deeper for montane birds of the Atlantic Forest
Source: Nat Commun. 2021 Nov 1;12:6269. doi: 10.1038/s41467-021-26537-9 (PMC8560783; doi:10.1038/s41467-021-26537-9)
Supplement: Supplementary file 9 — Reporting Summary [file 41467_2021_26537_MOESM9_ESM.pdf]

nature portfolio

Corresponding author(s): Gregory Thom

Last updated by author(s): Oct 1, 2021

Reporting Summary

Nature Portfolio wishes to improve the reproducibility of the work that we publish. This form provides structure for consistency and transparency in reporting. For further information on Nature Portfolio policies, see our Editorial Policies and the Editorial Policy Checklist.

Statistics

For all statistical analyses, confirm that the following items are present in the figure legend, table legend, main text, or Methods section.

n/a	Confirmed	
☐	☒	The exact sample size (*n*) for each experimental group/condition, given as a discrete number and unit of measurement
☒	☐	A statement on whether measurements were taken from distinct samples or whether the same sample was measured repeatedly
☐	☒	The statistical test(s) used AND whether they are one- or two-sided<br>*Only common tests should be described solely by name; describe more complex techniques in the Methods section.*
☐	☒	A description of all covariates tested
☐	☒	A description of any assumptions or corrections, such as tests of normality and adjustment for multiple comparisons
☐	☒	A full description of the statistical parameters including central tendency (e.g. means) or other basic estimates (e.g. regression coefficient) AND variation (e.g. standard deviation) or associated estimates of uncertainty (e.g. confidence intervals)
☐	☒	For null hypothesis testing, the test statistic (e.g. *F*, *t*, *r*) with confidence intervals, effect sizes, degrees of freedom and *P* value noted<br>*Give P values as exact values whenever suitable.*
☒	☐	For Bayesian analysis, information on the choice of priors and Markov chain Monte Carlo settings
☐	☒	For hierarchical and complex designs, identification of the appropriate level for tests and full reporting of outcomes
☒	☐	Estimates of effect sizes (e.g. Cohen's *d*, Pearson's *r*), indicating how they were calculated

Our web collection on statistics for biologists contains articles on many of the points above.

Software and code

Policy information about availability of computer code

Data collection

To obtain species occurrence records, we data mined and filtered records from the Global Biodiversity Information Facility, GBIF.org. The data was downloaded using the script "1_Downloading_gbif_data.R", available at https://github.com/GregoryThom/ and at https://doi.org/10.5281/zenodo.5510615. Climatic data was obtained from https://chelsa-climate.org/downloads/ with a 2.5 minutes spatial resolution. The genetic samples were obtained in the field and genomic data was generated in the lab and Library preparation and Illumina sequencing was outsourced to the University of Wisconsin Biotechnology Center (UWBC, Madison, WI).

Data analysis

BSSM
To simulate the effect of distance on individuals dispersion we built for the tropical and subtropical populations of each species, a bidimensional stepping stone model (BSSM) where each locality represented a population with an assigned effective population size (Ne). Populations were connected through migration (Nm) to two of their nearest neighbors. We simulated summary statistics using msABC (Pavlidis et al. 2010). We generated goodness-of-fit plots using the gfit function of abc v2.1 in R v3.6. The simulated datasets were used to train a neural network (NN) regression model, using the R v3.6 interface to Keras v2.3 (https://github.com/rstudio/keras). The Bidimensional stepping stone coalescent modeling is a novel software described in our manuscript. The code necessary to replicate our approach is available at https://doi.org/10.5281/zenodo.5512912 (https://github.com/gehara/BiSSM).

Bioinformatics and genetic structure between and within mountain regions

Sample demultiplexing, de novo assembly, and SNP calling for each species was performed in ipyrad v0.9.18. Parameter files used in Ipyrad for each species are available at https://github.com/GregoryThom/ and at https://doi.org/10.5281/zenodo.5510615.
We tested for the number of ancestral populations using conStruct in R v3.6. To obtain pairwise genetic distance matrices we used Adegenet v1.347 in R v3.6.

Estimation of historical demographic parameters
To estimate historical demographic parameters, we used Fastsimcoal v 2.6. An example of parameter files (.est and .tpl) is available at https://github.com/GregoryThom/ and at https://doi.org/10.5281/zenodo.5510615. We used easySFS v1.0 (https://github.com/isaacovercast/easySFS; access date: 05/01/2020) to estimate the projection of SNPs that maximize the number of segregating sites in the Joint SFSs. To estimate the number of segregating sites (S), nucleotide diversity (π), Tajima's D, and pairwise FST, we used PipeMaster v0.0.9 47 in R v3.6. Script available at https://github.com/GregoryThom/ and at https://doi.org/10.5281/zenodo.5510615, "1_summary_stats_pipemaster.R".

Species distribution models
We reduced sampling biases and homogenized the density of occurrences across space, by applying a spatial thinning using a 10 km distance in spThin v0.1.0.1. Current and past habitat suitability was estimated in Maxent v3.4.1. To avoid model overfitting, we evaluated distinct feature classes and regularization multipliers using the ENMeval v0.3.0. Variables were normalized to the mean and submitted to a PCA as implemented in prcomp v3.6.1. To remove extrapolation from the projected SDMs generated by combinations of climatic variables not represented by the training dataset, we used a multivariate environmental similarity surface (MESS) analysis in dismo v1.3-3. All programs mentioned above were used in R v3.6. The pipelines using these programs is available at https://github.com/GregoryThom/ and at https://doi.org/10.5281/zenodo.5510615, "1_ENM_in_R_CHELSA.R" and "1_ENM_past in_R_CHELSA.R".

Occupied environmental space
Values of the CHELSA bioclimatic variables at 30 seconds resolution were extracted from occurrence localities in raster v2.6-7. Variables were normalized and submitted to a PCA with prcomp v 3.6.3. We computed a per-species multidimensional volume of the occupied climatic space using hypervolume v2.0.12. All programs mentioned above were used in R v3.6. The pipelines using these programs is available at https://github.com/GregoryThom/ and at https://doi.org/10.5281/zenodo.5510615, "1_hypervolume.R"

Testing predictors for the spatial variation in population differentiation
We built mixed-effect models exploring the relative contribution of temperature seasonality and local elevation using the lme4 in R v3.6.
We assessed the individual and the combined effects of landscape resistance layers on genetic distance using ResistanceGA v4.0 in R v3.6. A script to replicate our ResistanceGA analyses is available at https://github.com/GregoryThom/ and at https://doi.org/10.5281/zenodo.5510615, "1_Resistance_GA.R"
To test for a community-wide predictor of genetic differentiation, we implemented a maximum likelihood population effect model (MLPE) with nlme v3.1 and corMLPE v0.0.2 (https://github.com/nspope/corMLPE) in R v3.6. A script to replicate these analyses is available at https://github.com/GregoryThom/ and at https://doi.org/10.5281/zenodo.5510615, "1_LMEM.R"

Testing for associations between the environment and genetic summary statistics.
To test for the association between multiple population genetics summary statistics and environmental variables with mountain regions controlling for the relatedness among species, we applied Phylogenetic Generalized Least Squares (PGLS) models using caper v1.0.1. To test for the phylogenetic signal on the measured variables we calculated Blomberg's K with adiv v2.0. All programs mentioned above were used in R v3.6. A script to replicate these analyses is available at https://github.com/GregoryThom/ and at https://doi.org/10.5281/zenodo.5510615, "1_PGLS".

For manuscripts utilizing custom algorithms or software that are central to the research but not yet described in published literature, software must be made available to editors and reviewers. We strongly encourage code deposition in a community repository (e.g. GitHub). See the Nature Portfolio guidelines for submitting code & software for further information.

Data

Policy information about availability of data

All manuscripts must include a data availability statement. This statement should provide the following information, where applicable:

- Accession codes, unique identifiers, or web links for publicly available datasets
- A description of any restrictions on data availability
- For clinical datasets or third party data, please ensure that the statement adheres to our policy

All data needed to evaluate the conclusions of this study are present in the manuscript and/or in the "Supplementary Information". Additional data related to this study is available at https://github.com/GregoryThom/ and at https://doi.org/10.5281/zenodo.5510615. The genetic data generated in this study have been deposited at the NCBI Short Read Archive under the BioProject PRJNA723198 at https://www.ncbi.nlm.nih.gov/bioproject/PRJNA723198. The occurrence records used in this study were obtained from the Global Biodiversity Information Facility and are available at https://doi.org/10.15468/dl.8383sp. The maps on figure 1a, supplementary figure 3 and supplementary figure 4 were designed using QGIS (https://qgis.org/en/site/), and the open source "Elevation - Global version 1" shapefile obtained at https://globalmaps.github.io/el.html (Geospatial Information Authority of Japan). Climatic data was obtained from https://chelsa-climate.org/downloads/. Additional data generated in this study are provided in the Supplementary Data files. Source data are provided with this paper.

Field-specific reporting

Please select the one below that is the best fit for your research. If you are not sure, read the appropriate sections before making your selection.

☐ Life sciences ☐ Behavioural & social sciences ☒ Ecological, evolutionary & environmental sciences

For a reference copy of the document with all sections, see nature.com/documents/nr-reporting-summary-flat.pdf

Ecological, evolutionary & environmental sciences study design

All studies must disclose on these points even when the disclosure is negative.

Study description	All data needed to evaluate the conclusions of this study are present in the manuscript and/or in the "Supplementary Information".

Study description	Additional data related to this study is available at https://github.com/GregoryThom/ and at https://doi.org/10.5281/zenodo.5510615. The genetic data generated in this study have been deposited at the NCBI Short Read Archive under the BioProject PRJNA723198 at https://www.ncbi.nlm.nih.gov/bioproject/PRJNA723198. The occurrence records used in this study were obtained from the Global Biodiversity Information Facility and are available at https://doi.org/10.15468/dl.8383sp. Climatic data was obtained from https://chelsa-climate.org/downloads/. Additional data generated in this study are provided in the Supplementary Data files. Source data are provided with this paper.
Research sample	A total of 229 tissue samples of 21 species were obtained in the field or requested from natural history collections (mean = 11.2; SD = 3.08 samples per species). A detailed description of each sample, including geographic coordinates, voucher number, institution that currently mantain the sample is available in table S12.
Sampling strategy	Our sampling design focused on obtaining the best spatial distribution of samples as possible. However a standardize sampling scheme for all species was not possible due to variation in species distributional ranges, abundance and microhabitat occupation. Here we focused in obtaining approximately 10 samples per species (five per population), that is an adequate number for demographic parameter estimation, and for our BSSM approach, which recovered a high accuracy in parameter estimation with this sample size. We included a section on the discussion of our manuscript describing our sampling scheme, "Signal versus noise: the benefits of community wide sampling": "Despite the relatively reduced number of samples per species (mean=11.2), our results remained consistent among species and between independent approaches. By analyzing individual taxa we were able to explore the variation in the main predictors among species, and by combining many species in the same analyses we reduced the effect of low sampling for individual taxa, capturing the major process driving community-wide genetic differentiation across a latitudinal gradient".
Data collection	Specimens were collected in the field with mist nets by Gregory Thom under the ICMBio/SISBIO permits 30835-3 and 47205-1 and approved by the Animal Ethics Committee of the Universidade Federal de São Paulo (CEP 0069/12, CEUA 3139240719 and CEUA 6433170817). All genetic samples were included on the SisGen platform under the protocols R2F97C8 and RB9572E. Additional samples were requested from Scientific collections that are described in the Acknowledgments
Timing and spatial scale	The samples used in this study were obtained over the years, between 2000 and 2018. This time frame does not affect the results of our study. Our sampling design focused on optimizing the spatial distribution of samples across their geographic distribution and spanned the entire Southern Atlantic Forest.
Data exclusions	We excluded species with limited number of samples, and filtered out loci with elevated amount of missing data from specific analyses. We also reduced sampling biases and homogenized the density of occurrences across space, and excluded occurrences on the elevation limit of the species.<br>Only loci with less than 25% missing information were retained.<br>To reduce the possibility of linked SNPs, one variant per locus was randomly selected for downstream analyses.<br>Species with populations with less than 3 individuals were excluded from our demographic analyses.<br>We apply a spatial thinning using a 10 km distance, removing duplicated occurrences within this radius.<br>We removed the top and bottom 5% of the occurrence records based on elevation to avoid the inclusion of outliers records outside the elevational distribution of the species.<br>We only included species with at least three sampled localitiesin our BSSM approach.
Reproducibility	Both data processing and analyses were conducted multiple times in order to adjust parameters and confirm our results. We based our main conclusions on distinct pieces of evidence generated by an independent set of analyses that corroborated a similar scenario. We included a section in the discussion of our manuscript describing this process "Signal versus noise: the benefits of community-wide sampling".
Randomization	Individuals were assigned to a mountain region (tropical or subtropical) based on geographic location. When testing the relative contribution of environmental matrixes on genetic diversity we used an approach that fits a mixed-effect model with a correlation structure designed to account for the non-independence of pairwise distance matrices, treating the residuals of the pairwise comparisons as the sum of two random populational level effects. This procedure accounted for the correlated nature of pairwise distance matrices, species-specific variation in genetic differentiation, and taxonomy (represented by the family of each species) as random effects.
Blinding	A blind experiment was not possible or applicable to our study given that we needed the geographic location of the samples to be revealed so we could build our models, and test our hypothesis

Did the study involve field work? ☒ Yes ☐ No

Field work, collection and transport

Field conditions	The conditions on which the samples were obtained are not disclosed in the metadata of tissue samples deposited in scientific collections. However, specific conditions when the tissue samples were obtained are not relevant for our study. We tested if seasonal climatic variation could impact the elevational distribution of the occurrence records obtained from Gbif. Our results suggest the at the geographic scale of our study the season on which an occurrence record was obtained does not affect the elevational distribution of a population (Table S5)
Location	A detailed description of each sample, including geographic coordinates, voucher number, institution that currently maintains the sample is available in Supplementary data 1.

Access & import/export	Genetic samples were exported from Brazil after authorization by the competent organ, SisGen protocols R2F97C8, and RB9572E
Disturbance	Any significant impact on biodiversity was caused by this study. Our sampling scheme aimed to collect between 1-3 individuals per species in distant localities, minimizing the impact on the local abundance of the species. We prioritize samples available in Scientific collections to avoid resampling of individuals from a given locality.

Reporting for specific materials, systems and methods

We require information from authors about some types of materials, experimental systems and methods used in many studies. Here, indicate whether each material, system or method listed is relevant to your study. If you are not sure if a list item applies to your research, read the appropriate section before selecting a response.

Materials & experimental systems

n/a	Involved in the study
☒	☐ Antibodies
☒	☐ Eukaryotic cell lines
☒	☐ Palaeontology and archaeology
☐	☒ Animals and other organisms
☒	☐ Human research participants
☒	☐ Clinical data
☒	☐ Dual use research of concern

Methods

n/a	Involved in the study
☒	☐ ChIP-seq
☒	☐ Flow cytometry
☒	☐ MRI-based neuroimaging

Animals and other organisms

Policy information about studies involving animals; ARRIVE guidelines recommended for reporting animal research

Laboratory animals

This study did not use laboratory animals

Wild animals

Specimens collected in the field were euthanized with chest compression or with anesthetic, muscle tissue was extracted and fixed in absolute ethanol. Specimens were deposited a the Laboratorio de Genetica e Evolucao Molecular de Aves (LGEMA), and Museu de Zoologia da Universidade de Sao Paulo (MZUSP). All samples were collected under the ICMBio/SISBIO permits 30835-3 and 47205-1 and approved by the Animal Ethics Committee of the Universidade Federal de São Paulo (CEP 0069/12, CEUA 3139240719 and CEUA 6433170817). Especiemens used in this study are deposited in the following institutions: FURB - Coleção Zoológica da Universidade Regional de Blumenau; LGEMA - Laboratório de Genética e Evolução Molecular de Aves, MPEG - Museu Paraense Emílio Goeldi, MCT-PUCRS - Museu de Ciências e Tecnologia da Pontifícia Universidade Católica do Rio Grande do Sul; MZUSP - Museu de Zoologia da Universidade de São Paulo.
List of specimens used in this study:
Cacicus chrysopterus (Brazil Itati- Rota do Sol- RS -29.355 -50.177 LGEMA18848)
Cacicus chrysopterus (Brazil Ponte Serrada- Caratuva- Parque Nacional das Araucárias- SC -26.808 -51.975 FURB13869)
Cacicus chrysopterus (Brazil Caçador- Linha São Francisco- SC -26.755 -51.079 MPEGT14970)
Cacicus chrysopterus (Brazil Doutor Pedrinho- Reserva Biológica Estadual do Sassafrás – REBIO- SC -26.713 -49.677 FURB13447)
Cacicus chrysopterus (Brazil Estação Biológica de Boracéia- Salesópolis -23.656 -45.891 MPEGT14967)
Cacicus chrysopterus (Brazil Bananal- E.E. Bananal- SP -22.781 -44.387 LGEMA18744)
Cacicus chrysopterus (Brazil Bananal- E.E. Bananal- SP -22.783 -44.36 LGEMA18753)
Cacicus chrysopterus (Brazil Piquete- Estada vicinal- SP` -22.567 -45.223 LGEMA18874)
Cacicus chrysopterus (Brazil Delfim Moreira- Estrada para pico dos Marins- MG -22.547 -45.196 LGEMA18821)
Chamaeza ruficauda (Brazil Maquine- Serra do Umbu- RS -29.499 -50.324 LGEMA18849)
Chamaeza ruficauda (Brazil Urubici- Morro do Antena- SC -28.001 -49.616 LGEMA18861)
Chamaeza ruficauda (Brazil Urubici- Morro do Antena- SC -28.005 -49.625 LGEMA18862)
Chamaeza ruficauda (Brazil Doutor Pedrinho- Reserva Biológica Estadual do Sassafrás – REBIO- SC -26.713 -49.677 FURB13361)
Chamaeza ruficauda (Brazil Sao Jose do Barreiro- Entorno do PARNA da Serra da Bocaina- SP -22.692 -44.636 LGEMA18796)
Chamaeza ruficauda (Brazil Sao Jose do Barreiro- Entorno do PARNA da Serra da Bocaina- SP -22.694 -44.633 LGEMA18800)
Chamaeza ruficauda (Brazil Piquete- Trilha do ataque- pista de voo - SP -22.569 -45.226 LGEMA19527)
Chamaeza ruficauda (Brazil Marmelópolis- Estrada para pico dos Marins - SC -22.533 -45.168 LGEMA19542)
Chamaeza ruficauda (Brazil PN Serra dos Orgaos Teresopolis -22.458 -43.026 LGEMA18508)
Drymophila rubricollis (Brazil PARNA Serra do Itajaí- Indaial -27.011 -49.21 LGEMA18576)
Drymophila rubricollis (Brazil PARNA Serra do Itajaí- Indaial -27.084 -49.258 LGEMA18590)
Drymophila rubricollis (Brazil Quatro Barras- Estrada do Corvo- PR -25.337 -48.914 LGEMA18668)
Drymophila rubricollis (Brazil Quatro Barras- Morro do Capivari- PR -25.122 -48.825 LGEMA18517)
Drymophila rubricollis (Brazil Quatro Barras- Morro do Capivari- PR -25.121 -48.823 LGEMA18547)
Drymophila rubricollis (Brazil Barra do Chapeu- SP -24.509 -48.994 LGEMA18425)
Drymophila rubricollis (Brazil Bananal- E.E. Bananal- SP -22.781 -44.387 LGEMA18636)
Drymophila rubricollis (Brazil Sao Jose do Barreiro- Entorno do PARNA da Serra da Bocaina- SP -22.694 -44.633 LGEMA18794)
Drymophila rubricollis (Brazil Sao Jose do Barreiro- Entorno do PARNA da Serra da Bocaina- SP -22.692 -44.636 LGEMA18802)
Drymophila rubricollis (Brazil Piquete- Trilha do ataque- pista de voo - SP -22.567 -45.254 LGEMA19531)
Drymophila rubricollis (Brazil Piquete- Trilha do ataque- pista de voo - SP -22.584 -45.254 LGEMA19539)
Drymophila rubricollis (Brazil Piquete- Trilha do ataque- pista de voo - SP -22.569 -45.226 LGEMA19541)
Dysithamnus xanthopterus (Brazil Quatro Barras- Morro do Capivari- PR -25.121 -48.823 LGEMA18568)

Dysithamnus xanthopterus (Brazil Quatro Barras- Morro do Capivari- PR -25.122 -48.825 LGEMA18569)
Dysithamnus xanthopterus (Brazil Ibiuna- SP -23.829 -47.184 LGEMA17783)
Dysithamnus xanthopterus (Brazil Piedade- SP -23.804 -47.322 LGEMA2449)
Dysithamnus xanthopterus (Brazil Bananal- E.E. Bananal- SP -22.781 -44.387 LGEMA14718)
Dysithamnus xanthopterus (Brazil Sao Jose do Barreiro- Entorno do PARNA da Serra da Bocaina- SP -22.694 -44.633 LGEMA18774)
Dysithamnus xanthopterus (Brazil Sao Jose do Barreiro- Entorno do PARNA da Serra da Bocaina- SP -22.692 -44.636 LGEMA18785)
Dysithamnus xanthopterus (Brazil Piquete- Trilha do ataque- pista de voo - SP -22.569 -45.226 LGEMA19533)
Dysithamnus xanthopterus (Brazil Piquete- Estada vicinal- SP` -22.567 -45.223 LGEMA18869)
Dysithamnus xanthopterus (Brazil Piquete- Estada vicinal- SP` -22.584 -45.226 LGEMA18870)
Elaenia mesoleuca (Brazil Sao Francisco De Paula- Centro de Pesquisas e Conservação da Natureza Pró-Mata- RS -29.481 -50.175 PUC5295)
Elaenia mesoleuca (Brazil PNSJ- Gnid PPBio- Ponto 3500- SC -28.115 -49.506 FURB25287)
Elaenia mesoleuca (Brazil Campos Belo do Sul- Florestal Gateados- SC -27.983 -50.85 MPEGT15001)
Elaenia mesoleuca (Brazil Caçador- Linha São Francisco- SC -26.755 -51.079 MPEGT14969)
Elaenia mesoleuca (Brazil Bananal- E.E. Bananal- SP -22.781 -44.387 LGEMA2573)
Elaenia mesoleuca (Brazil Campos do Jordao- Pico do Imbiri - SP -22.703 -45.589 LGEMA19548)
Elaenia mesoleuca (Brazil Campos do Jordao- Pedra do Bau - SP -22.671 -45.622 LGEMA19550)
Heliobletus contaminatus (Brazil Sao Francisco de Paula- Parque Municipal da RONDA- RS -29.447 -50.544 LGEMA18834)
Heliobletus contaminatus (Brazil Rancho Queimado- SC -27.737 -49.124 LGEMA2194)
Heliobletus contaminatus (Brazil Ponte Serrada- Caratuva- Parque Nacional das Araucárias- SC -26.808 -51.975 FURB13872)
Heliobletus contaminatus (Brazil Caçador- Linha São Francisco- SC -26.755 -51.079 MPEGT14981)
Heliobletus contaminatus (Brazil Quatro Barras- Morro do Capivari- PR -25.122 -48.825 LGEMA18520)
Heliobletus contaminatus (Brazil Quatro Barras- Morro do Capivari- PR -25.121 -48.823 LGEMA18561)
Heliobletus contaminatus (Brazil Itanhaem- Parque Estadual da Serra do Mar- SP -23.95 -46.721 LGEMA15074)
Heliobletus contaminatus (Brazil Salesópolis- Estação Biológica de Boracéia -23.656 -45.891 LGEMA2450)
Heliobletus contaminatus (Brazil Bananal- E.E. Bananal- SP -22.781 -44.387 LGEMA18748)
Heliobletus contaminatus (Brazil Campos do Jordao- Pico do Imbiri - SP -22.703 -45.589 LGEMA19547)
Heliobletus contaminatus (Brazil Campos do Jordao- Pedra do Bau - SP -22.671 -45.622 LGEMA19549)
Hemitriccus obsoletus (Brazil Sao Bonifacio- Parque Estadual da Serra do Tabuleiro- SC -27.909 -48.873 PUC4973)
Hemitriccus obsoletus (Brazil Blumenau- Vale do Espingarda- Parque das Nascentes- sub-sede- SC -27.042 -49.154 FURB10148)
Hemitriccus obsoletus (Brazil Parna das AraucáriasPonte Serrada -26.836 -51.933 FURB13850)
Hemitriccus obsoletus (Brazil Quatro Barras- Morro do Capivari- PR -25.122 -48.825 LGEMA18155)
Hemitriccus obsoletus (Brazil Quatro Barras- Morro do Capivari- PR -25.121 -48.823 LGEMA18385)
Hemitriccus obsoletus (Brazil Bananal- E.E. Bananal- SP -22.783 -44.36 LGEMA18749)
Hemitriccus obsoletus (Brazil Bananal- E.E. Bananal- SP -22.781 -44.387 LGEMA18750)
Hemitriccus obsoletus (Brazil Bananal- E.E. Bananal- SP -22.777 -44.349 LGEMA18751)
Hemitriccus obsoletus (Brazil Campos do Jordão- Fazenda Prana -22.739 -45.53 LGEMA18845)
Hemitriccus obsoletus (Brazil Sao Jose do Barreiro- Entorno do PARNA da Serra da Bocaina- SP -22.694 -44.633 LGEMA18788)
Hemitriccus obsoletus (Brazil Piquete- Trilha do ataque- pista de voo- SP -22.569 -45.226 LGEMA18813)
Hemitriccus obsoletus (Brazil Piquete- Trilha do ataque- pista de voo - SP -22.584 -45.254 LGEMA19528)
Hemitriccus obsoletus (Brazil Piquete- Trilha do ataque- pista de voo - SP -22.567 -45.224 LGEMA19532)
Mackenziaena leachii (Brazil Maquine- RS -29.5 -50.314 LGEMA17997)
Mackenziaena leachii (Brazil Maquine- Serra do Umbu- RS -29.499 -50.324 LGEMA18847)
Mackenziaena leachii (Brazil Urubici- Morro do Antena- SC -28.001 -49.616 LGEMA18856)
Mackenziaena leachii (Brazil Quatro Barras- Estrada do Corvo- PR -25.337 -48.914 MPEGT6547)
Mackenziaena leachii (Brazil Campina Grande do Sul- Morro do Capivari- PR -25.125 -48.528 LGEMA18864)
Mackenziaena leachii (Brazil Sao Bernardo do Campo- SP -23.911 -46.567 LGEMA13690)
Mackenziaena leachii (Brazil Sao Jose do Barreiro- Entorno do PARNA da Serra da Bocaina- SP -22.694 -44.633 LGEMA18797)
Muscipipra vetula (Brazil Maquine- Serra do Umbu- RS -29.499 -50.324 LGEMA18850)
Muscipipra vetula (Brazil Urubici- Morro do Antena- SC -28.001 -49.616 LGEMA18858)
Muscipipra vetula (Brazil Doutor Pedrinho- Reserva Biológica Estadual do Sassafrás – REBIO- SC -26.713 -49.677 FURB13448)
Muscipipra vetula (Brazil Estação Biológica de Boracéia- Salesópolis -23.656 -45.891 LGEMA2690)
Muscipipra vetula (Brazil Bananal- E.E. Bananal- SP -22.781 -44.387 LGEMA18746)
Muscipipra vetula (Brazil Campos do Jordao- Pico do Imbiri - SP -22.703 -45.589 LGEMA19544)
Muscipipra vetula (Brazil Piquete- Trilha do ataque- pista de voo - SP -22.569 -45.226 LGEMA19535)
Muscipipra vetula (Brazil Piquete- Trilha do ataque- pista de voo - SP -22.569 -45.226 LGEMA19536)
Phylloscartes difficilis (Brazil Sao Francisco De Paula- Centro de Pesquisas e Conservação da Natureza Pró-Mata- RS -29.481 -50.175 PUC5452)
Phylloscartes difficilis (Brazil Quatro Barras- Morro do Capivari- PR -25.336 -48.909 LGEMA18386)
Phylloscartes difficilis (Brazil Quatro Barras- Morro do Capivari- PR -25.338 -48.916 LGEMA18387)
Phylloscartes difficilis (Brazil Quatro Barras- Morro do Capivari- PR -25.341 -48.821 LGEMA18527)
Phylloscartes difficilis (Brazil Quatro Barras- Morro do Capivari- PR -25.343 -48.927 LGEMA18529)
Phylloscartes difficilis (Brazil Paraty- PN Serra da Bocaina- RJ -23.198 -44.841 LGEMA18507)
Phylloscartes difficilis (Brazil Paraty- PN Serra da Bocaina- RJ -23.179 -44.832 LGEMA18480)
Phylloscartes difficilis (Brazil Cunha- Estrada vicinal- SP -23.168 -44.84 LGEMA18628)
Phylloscartes difficilis (Brazil Campos do Jordao- Pico do Imbiri - SP -22.703 -45.589 LGEMA19546)
Phylloscartes difficilis (Brazil Sao Jose do Barreiro- Entorno do PARNA da Serra da Bocaina- SP -22.694 -44.633 LGEMA18795)
Phylloscartes difficilis (Brazil Piquete- Trilha do ataque- pista de voo- SP -22.569 -45.226 LGEMA18817)
Phylloscartes difficilis (Brazil Piquete- Trilha do ataque- pista de voo - SP -22.567 -45.224 LGEMA19537)
Phylloscartes ventralis (Brazil Pelotas- Arroio do Padre- SC -31.367 -52.383 LGEMA2176)
Phylloscartes ventralis (Brazil Maquine- Serra do Umbu- RS -29.499 -50.324 LGEMA18842)
Phylloscartes ventralis (Brazil Sao Francisco de Paula- Parque Municipal da RONDA- RS -29.447 -50.544 LGEMA18837)
Phylloscartes ventralis (Brazil Urubici- imediacoes do PN Sao Joaquim -28.128 -49.668 LGEMA18675)
Phylloscartes ventralis (Brazil Ponte Serrada- Caratuva- Parque Nacional das Araucárias- SC -26.808 -51.975 FURB13842)

Phylloscartes ventralis (Brazil Doutor Pedrinho- Reserva Biológica Estadual do Sassafrás – REBIO- SC -26.713 -49.677 FURB13382)
Phylloscartes ventralis (Brazil Quatro Barras- Morro do Capivari- PR -25.338 -48.916 LGEMA18552)
Phylloscartes ventralis (Brazil Quatro Barras- Morro do Capivari- PR -25.341 -48.921 LGEMA18560)
Phylloscartes ventralis (Brazil Ibiuna- SP -23.829 -47.184 LGEMA17787)
Phylloscartes ventralis (Brazil Tapirai- Fazenda Santa Regina- SP -23.802 -47.327 LGEMA17875)
Phylloscartes ventralis (Brazil Biritiba Mirim- Estacao Ecologica de Boraceia- SP -23.656 -45.891 LGEMA16483)
Phylloscartes ventralis (Brazil Bananal- E.E. Bananal- SP -22.781 -44.387 LGEMA18745)
Phylloscartes ventralis (Brazil Bananal- E.E. Bananal- SP -22.781 -44.387 LGEMA18747)
Phylloscartes ventralis (Brazil Campos do Jordao- SP -22.762 -45.591 LGEMA18766)
Phylloscartes ventralis (Brazil Campos do Jordão- Fazenda Prana -22.739 -45.53 LGEMA18844)
Piculus aurulentus (Brazil Maquine- Serra do Umbu- RS -29.499 -50.324 LGEMA18851)
Piculus aurulentus (Brazil Sao Francisco de Paula- Condominio Alpes- RS -29.454 -50.616 LGEMA18852)
Piculus aurulentus (Brazil Sao Bonifacio- Parque Estadual da Serra do Tabuleiro- SC -27.909 -48.873 PUC4959)
Piculus aurulentus (Brazil Rio dos Cedros - SC -26.585 -49.522 LGEMA18805)
Piculus aurulentus (Brazil Quatro Barras- Estrada do Corvo- PR -25.337 -48.914 MPEGT6553)
Piculus aurulentus (Brazil Estação Biológica de Boracéia -23.656 -45.891 LGEMA18608)
Piculus aurulentus (Brazil Bananal- E.E. Bananal- SP -22.781 -44.387 LGEMA18743)
Piculus aurulentus (Brazil Campos do Jordao- SP -22.762 -45.591 LGEMA18763)
Piculus aurulentus (Brazil Campos do Jordão- Fazenda Prana -22.739 -45.53 LGEMA18752)
Piculus aurulentus (Brazil Campos do Jordão- Fazenda Prana -22.696 -45.463 LGEMA18760)
Piculus aurulentus (Brazil Sao Jose do Barreiro- Entorno do PARNA da Serra da Bocaina- SP -22.694 -44.633 LGEMA18773)
Piculus aurulentus (Brazil Piquete- Trilha do ataque- pista de voo- SP -22.569 -45.226 LGEMA18810)
Piprites pileata (Brazil Bocaiuva do Sul- Ribeirao Amarelo- PR -25.033 -48.826 LGEMA18403)
Piprites pileata (Brazil Bocaiuva do Sul- Ribeirao Amarelo- PR -25.043 -48.83 LGEMA18407)
Piprites pileata (Brazil Campos do Jordão- Fazenda Prana -22.739 -45.53 LGEMA19529)
Piprites pileata (Brazil Piquete- Estada vicinal- SP` -22.567 -45.223 LGEMA18419)
Piprites pileata (Brazil Piquete- Estada vicinal- SP` -22.546 -45.196 LGEMA18448)
Poospiza cabanisi (Brazil Pelotas- RS -31.628 -52.523 LGEMA18447)
Poospiza cabanisi (Brazil Santa Maria- RS -29.597 -53.701 LGEMA18459)
Poospiza cabanisi (Brazil Urubici- PN Sao Joaquim- SC -28.145 -49.632 LGEMA18670)
Poospiza cabanisi (Brazil Guarapuava- PR -25.394 -51.431 LGEMA18470)
Poospiza cabanisi (Brazil Quatro Barras- Morro do Capivari- PR -25.122 -48.825 LGEMA18514)
Poospiza cabanisi (Brazil Apiai- Estrada vicinal- SP -24.476 -48.897 LGEMA18421)
Poospiza lateralis (Brazil Cunha- Estrada vicinal- SP -23.149 -44.933 LGEMA18410)
Poospiza lateralis (Brazil Camanducaia- MG -22.811 -46.425 LGEMA18391)
Poospiza lateralis (Brazil Camanducaia- MG -22.784 -46.082 LGEMA18393)
Poospiza lateralis (Brazil Sao Jose do Barreiro - Serra da Bocaina - SP -22.694 -44.633 LGEMA18482)
Poospiza lateralis (Brazil Piquete- Estada vicinal- SP` -22.546 -45.196 LGEMA18395)
Poospiza lateralis (Brazil Piquete- Estada vicinal- SP` -22.567 -45.223 LGEMA18399)
Poospiza thoracica (Brazil Cambara Do Sul- RS -28.894 -50.03 PUC1077)
Poospiza thoracica (Brazil Sào José dos Ausentes- Fazenda Monte Negro- ca 30km NE- RS -28.597 -49.8 MPEGT6536)
Poospiza thoracica (Brazil Urubici- PN Sao Joaquim- SC -28.145 -49.632 LGEMA18680)
Poospiza thoracica (Brazil Urubici- Serra do Corvo Branco- SC -28.055 -49.374 LGEMA18863)
Poospiza thoracica (Brazil Rio Negrinho- Fazenda Serra Azul- SC -26.711 -49.583 FURB13205)
Poospiza thoracica (Brazil Quatro Barras- Morro do Capivari- PR -25.122 -48.825 LGEMA18137)
Poospiza thoracica (Brazil Paraty- PN Serra da Bocaina- RJ -23.168 -44.84 LGEMA18652)
Poospiza thoracica (Brazil Campos do Jordao- estrada para o Pico do Diamante - SP -22.785 -45.581 LGEMA19552)
Poospiza thoracica (Brazil Campos do Jordão- Fazenda Prana -22.739 -45.53 LGEMA2428)
Poospiza thoracica (Brazil Sao Jose do Barreiro - Serra da Bocaina - SP -22.694 -44.633 LGEMA18485)
Poospiza thoracica (Brazil Sao Jose do Barreiro - Serra da Bocaina - SP -22.692 -44.636 LGEMA18486)
Poospiza thoracica (Brazil Sao Jose do Barreiro- Entorno do PARNA da Serra da Bocaina- SP -22.698 -44.626 LGEMA18801)
Poospiza thoracica (Brazil Piquete- Estada vicinal- SP` -22.567 -45.223 LGEMA18627)
Poospiza thoracica (Brazil Piquete- Estada vicinal- SP` -22.567 -45.223 LGEMA18872)
Saltator maxillosus (Brazil Sapiranga- Sitio Ismael Franz- RS -29.55 -50.912 LGEMA18826)
Saltator maxillosus (Brazil Sapiranga- Sitio Ismael Franz- RS -29.53 -50.915 LGEMA18827)
Saltator maxillosus (Brazil Urubici- Morro do Antena- SC -28.001 -49.616 LGEMA18859)
Saltator maxillosus (Brazil Chapecó- SC -27.139 -52.714 FURB13885)
Saltator maxillosus (Brazil Ponte Serrada- Caratuva- Parque Nacional das Araucárias- SC -26.808 -51.975 FURB13862)
Saltator maxillosus (Brazil Campos do Jordão- Fazenda Prana -22.739 -45.53 LGEMA19540)
Saltator maxillosus (Brazil Campos do Jordao- Pico do Imbiri - SP -22.703 -45.589 LGEMA19543)
Saltator maxillosus (Brazil Sao Jose do Barreiro- Entorno do PARNA da Serra da Bocaina- SP -22.692 -44.636 LGEMA18775)
Saltator maxillosus (Brazil Sao Jose do Barreiro- Entorno do PARNA da Serra da Bocaina- SP -22.694 -44.633 LGEMA18776)
Saltator maxillosus (Brazil Marmelópolis- Estrada para pico dos Marins- MG -22.547 -45.196 LGEMA18814)
Stephanophorus diadematus (Brazil Pelotas- Arroio do Padre- SC -31.367 -52.383 LGEMA2137)
Stephanophorus diadematus (Brazil Jaquirana- Parque Estadual do Tainhas- RS -29.097 -50.354 PUC4061)
Stephanophorus diadematus (Brazil Ponte Serrada- Caratuva- Parque Nacional das Araucárias- SC -26.808 -51.975 FURB13843)
Stephanophorus diadematus (Brazil Quatro Barras- Morro do Capivari- PR -25.122 -48.825 LGEMA18524)
Stephanophorus diadematus (Brazil Campos do Jordao- SP -22.762 -45.591 LGEMA18761)
Stephanophorus diadematus (Brazil Sao Jose do Barreiro- Entorno do PARNA da Serra da Bocaina- SP -22.694 -44.633 LGEMA18770)
Stephanophorus diadematus (Brazil Sao Jose do Barreiro- Entorno do PARNA da Serra da Bocaina- SP -22.692 -44.636 LGEMA18771)
Stephanophorus diadematus (Brazil Sao Jose do Barreiro- Entorno do PARNA da Serra da Bocaina- SP -22.698 -44.626 LGEMA18793)
Stephanophorus diadematus (Brazil Piquete- Trilha do ataque- pista de voo- SP -22.569 -45.226 LGEMA18808)
Stephanoxis lalandi (Brazil Maquine- Serra do Umbu- RS -29.506 -50.324 LGEMA18142)

Stephanoxis lalandi (Brazil Maquine- Serra do Umbu- RS -29.503 -50.332 LGEMA18394)
Stephanoxis lalandi (Brazil Sao Bonifacio- Parque Estadual da Serra do Tabuleiro- SC -27.909 -48.873 LGEMA18483)
Stephanoxis lalandi (Brazil Doutor Pedrinho- Reserva Biológica Estadual do Sassafrás – REBIO- SC -26.713 -49.677 LGEMA13417)
Stephanoxis lalandi (Brazil Quatro Barras- Cerro Azul- PR -25.055 -49.101 LGEMA15766)
Stephanoxis lalandi (Brazil Quatro Barras- Cerro Azul- PR -25.341 -48.921 LGEMA15778)
Stephanoxis lalandi (Brazil Cunha- Estrada vicinal- SP -23.207 -45.008 LGEMA14716)
Stephanoxis lalandi (Brazil Cunha- Estrada vicinal- SP -23.207 -45.008 LGEMA15754)
Stephanoxis lalandi (Brazil Paraty- PN Serra da Bocaina- RJ -23.172 -44.836 LGEMA15780)
Stephanoxis lalandi (Brazil Campos do Jordão- Fazenda Prana -22.739 -45.53 LGEMA18672)
Stephanoxis lalandi (Brazil Sao Jose do Barreiro - Serra da Bocaina - SP -22.716 -44.632 LGEMA14717)
Stephanoxis lalandi (Brazil Piquete- Trilha do ataque- pista de voo- SP -22.569 -45.226 LGEMA17784)
Stephanoxis lalandi (Brazil Piquete- Trilha do ataque- pista de voo- SP -22.567 -45.224 LGEMA18138)
Stephanoxis lalandi (Brazil Piquete- Trilha do ataque- pista de voo - SP -22.584 -45.254 LGEMA18479)
Synallaxis cinerascens (Brazil Santa Cruz do Sul- RS -29.697 -52.38 LGEMA17991)
Synallaxis cinerascens (Brazil Sapiranga- Sitio Ismael Franz- RS -29.55 -50.912 LGEMA18828)
Synallaxis cinerascens (Brazil Sao Francisco de Paula- Parque Municipal da RONDA- RS -29.447 -50.544 LGEMA18836)
Synallaxis cinerascens (Brazil Caçador- Linha São Francisco- SC -26.755 -51.079 MPEGT14958)
Synallaxis cinerascens (Brazil Jambeiro- SP -23.234 -45.692 LGEMA13451)
Synallaxis cinerascens (Brazil Serra da Cantareira- Mairiporã -23.355 -46.515 LGEMA18528)
Synallaxis cinerascens (Brazil Sao Jose do Barreiro- Entorno do PARNA da Serra da Bocaina- SP -22.694 -44.633 LGEMA18792)
Synallaxis cinerascens (Brazil Piquete- Trilha do ataque- pista de voo- SP -22.569 -45.226 LGEMA18811)
Synallaxis cinerascens (Brazil Piquete- Trilha do ataque- pista de voo- SP -22.567 -45.224 LGEMA18812)
Synallaxis cinerascens (Brazil Piquete- Trilha do ataque- pista de voo - SP -22.584 -45.254 LGEMA19534)
Syndactyla rufosuperciliata (Brazil Pelotas- Arroio do Padre- SC -31.367 -52.383 LGEMA2129)
Syndactyla rufosuperciliata (Brazil Sao Francisco De Paula- Centro de Pesquisas e Conservação da Natureza Pró-Mata- RS -29.481 -50.175 PUC596)
Syndactyla rufosuperciliata (Brazil Sao Francisco de Paula- Parque Municipal da RONDA- RS -29.447 -50.544 LGEMA18831)
Syndactyla rufosuperciliata (Brazil Rancho Queimado- SC -27.737 -49.124 LGEMA2155)
Syndactyla rufosuperciliata (Brazil Caçador- Linha São Francisco- SC -26.755 -51.079 MPEGT14960)
Syndactyla rufosuperciliata Argentine Missiones- Paraje Maria Soledad- Departamento Gral Belgrano -25.858 -53.984 LGEMA10381)
Syndactyla rufosuperciliata (Brazil Quatro Barras- Morro do Capivari- PR -25.122 -48.825 LGEMA18523)
Syndactyla rufosuperciliata (Brazil Quatro Barras- Morro do Capivari- PR -25.116 -48.838 LGEMA18601)
Syndactyla rufosuperciliata (Brazil Serra da Cantareira- Mairiporã -23.355 -46.515 LGEMA18516)
Syndactyla rufosuperciliata (Brazil Campos do Jordão- Fazenda Prana -22.739 -45.53 LGEMA18767)
Syndactyla rufosuperciliata (Brazil Campos do Jordão- Fazenda Prana -22.696 -45.463 LGEMA18768)
Syndactyla rufosuperciliata (Brazil Sao Jose do Barreiro- Entorno do PARNA da Serra da Bocaina- SP -22.694 -44.633 LGEMA18772)
Syndactyla rufosuperciliata (Brazil Sao Jose do Barreiro- Entorno do PARNA da Serra da Bocaina- SP -22.692 -44.636 LGEMA18779)
Syndactyla rufosuperciliata (Brazil Sao Jose do Barreiro- Entorno do PARNA da Serra da Bocaina- SP -22.698 -44.626 LGEMA18791)
Syndactyla rufosuperciliata (Brazil Sao Jose do Barreiro- Entorno do PARNA da Serra da Bocaina- SP -22.699 -44.634 LGEMA18798)
Syndactyla rufosuperciliata (Brazil Delfim Moreira- Estrada para pico dos Marins- MG -22.547 -45.196 LGEMA18815)
Tangara desmaresti (Brazil Presidente Nereu- Agrião- PARNA Serra do Itajaí- SC -27.229 -49.289 FURB13928)
Tangara desmaresti (Brazil Doutor Pedrinho- Reserva Biológica Estadual do Sassafrás – REBIO- SC -26.713 -49.677 FURB13450)
Tangara desmaresti (Brazil Quatro Barras- Morro do Capivari- PR -25.122 -48.825 LGEMA18592)
Tangara desmaresti (Brazil Barra do Chapeu- SP -24.509 -48.994 LGEMA18423)
Tangara desmaresti (Brazil Barra do Chapeu- SP -24.528 -49.005 LGEMA18424)
Tangara desmaresti (Brazil Bananal- E.E. Bananal- SP -22.781 -44.387 LGEMA18755)
Tangara desmaresti (Brazil Campos do Jordao- SP -22.762 -45.591 LGEMA18764)
Tangara desmaresti (Brazil Sao Jose do Barreiro- Entorno do PARNA da Serra da Bocaina- SP -22.694 -44.633 LGEMA18781)
Tangara desmaresti (Brazil Sao Jose do Barreiro- Entorno do PARNA da Serra da Bocaina- SP -22.698 -44.626 LGEMA18784)
Tangara desmaresti (Brazil Piquete- Trilha do ataque- pista de voo- SP -22.569 -45.226 LGEMA18807)
Tangara desmaresti (Brazil Piquete- Trilha do ataque- pista de voo- SP -22.567 -45.224 LGEMA18816)
Tangara desmaresti (Brazil Piquete- Estada vicinal- SP` -22.567 -45.223 LGEMA18873)

Field-collected samples

Individuals were collected with mist nets and kept alive in ambient temperature for no more than 2 hours until the euthanazia. Tissue samples were fixed with absolute ethanol and kept at -20 Celsius degrees in the field . All tissue samples were stored at -80 Celsius degrees until DNA extraction.

Ethics oversight

All samples were collected under the ICMBio/SISBIO permits 30835-3 and 47205-1 and approved by the Animal Ethics Committee of the Universidade Federal de São Paulo (CEP 0069/12, CEUA 3139240719 and CEUA 6433170817).

Note that full information on the approval of the study protocol must also be provided in the manuscript.
